# Global, regional, and national burden of neurological disorders during 1990–2015: a systematic analysis for the Global Burden of Disease Study 2015

**DOI:** 10.1016/S1474-4422(17)30299-5

**Published:** 2017-11

**Authors:** GBD 2015 Neurological Disorders Collaborator Group, Valery L Feigin, Amanuel Alemu Abajobir, Kalkidan Hassen Abate, Foad Abd-Allah, Abdishakur M Abdulle, Semaw Ferede Abera, Gebre Yitayih Abyu, Muktar Beshir Ahmed, Amani Nidhal Aichour, Ibtihel Aichour, Miloud Taki Eddine Aichour, Rufus Olusola Akinyemi, Samer Alabed, Rajaa Al-Raddadi, Nelson Alvis-Guzman, Azmeraw T. Amare, Hossein Ansari, Palwasha Anwari, Johan Ärnlöv, Hamid Asayesh, Solomon Weldegebreal Asgedom, Tesfay Mehari Atey, Leticia Avila-Burgos, Euripide Frinel, G. Arthur Avokpaho, Aleksandra Barac, Miguel Barboza, Suzanne L Barker-Collo, Till Bärnighausen, Neeraj Bedi, Ettore Beghi, Derrick A Bennett, Isabela M Bensenor, Adugnaw Berhane, Balem Demtsu Betsu, Soumyadeep Bhaumik, Sait Mentes Birlik, Stan Biryukov, Dube Jara Boneya, Lemma Negesa Bulto Bulto, Hélène Carabin, Daniel Casey, Carlos A. Castañeda-Orjuela, Ferrán Catalá-López, Honglei Chen, Abdulaal A Chitheer, Rajiv Chowdhury, Hanne Christensen, Lalit Dandona, Rakhi Dandona, Gabrielle A de Veber, Samath D Dharmaratne, Huyen Phuc Do, Klara Dokova, E Ray Dorsey, Richard G Ellenbogen, Sharareh Eskandarieh, Maryam S Farvid, Seyed-Mohammad Fereshtehnejad, Florian Fischer, Kyle J Foreman, Johanna M Geleijnse, Richard F Gillum, Giorgia Giussani, Philimon N Gona, Alessandra Carvalho Goulart, Harish Chander Gugnani, Rahul Gupta, Rajeev Gupta, Randah Ribhi Hamadeh, Mitiku Hambisa, Graeme J Hankey, Habtamu Abera Hareri, Rasmus Havmoeller, Simon I Hay, Pouria Heydarpour, Peter J Hotez, Mihajlo (Michael) B Jakovljevic, Mehdi Javanbakht, Panniyammakal Jeemon, Jost B Jonas, Yogeshwar Kalkonde, Amit Kandel, André Karch, Amir Kasaeian, Anshul Kastor, Peter Njenga Keiyoro, Yousef Saleh Khader, Ibrahim A Khalil, Ejaz Ahmad Khan, Young-Ho Khang, Abdullah Tawfih, Abdullah Khoja, Jagdish Khubchandani, Daniel Kim, Yun Jin Kim, Mika Kivimaki, Yoshihiro Kokubo, Soewarta Kosen, Michael Kravchenko, Rita Vanmala Krishnamurthi, Barthelemy Kuate Defo, G Anil Kumar, Rashmi Kumar, Hmwe H Kyu, Anders Larsson, Pablo M Lavados, Yongmei Li, Xiaofeng Liang, Misgan Legesse Liben, Warren D Lo, Giancarlo Logroscino, Paulo A Lotufo, Clement T Loy, Mark T Mackay, Hassan Magdy Abd El Razek, Mohammed Magdy Abd El Razek, Azeem Majeed, Reza Malekzadeh, Treh Manhertz, Lorenzo G Mantovani, João Massano, Mohsen Mazidi, Colm McAlinden, Suresh Mehata, Man Mohan Mehndiratta, Ziad A Memish, Walter Mendoza, Mubarek Abera Mengistie, George A Mensah, Atte Meretoja, Haftay Berhane Mezgebe, Ted R Miller, Shiva Raj Mishra, Norlinah Mohamed Ibrahim, Alireza Mohammadi, Kedir Endris Mohammed, Shafiu Mohammed, Ali H Mokdad, Maziar Moradi-Lakeh, Ilais Moreno Velasquez, Kamarul Imran Musa, Mohsen Naghavi, Josephine Wanjiku Ngunjiri, Cuong Tat Nguyen, Grant Nguyen, Quyen Le Nguyen, Trang Huyen Nguyen, Emma Nichols, Dina Nur Anggraini Ningrum, Vuong Minh Nong, Bo Norrving, Jean Jacques N Noubiap, Felix Akpojene Ogbo, Mayowa O Owolabi, Jeyaraj D. Pandian, Max Petzold, Michael Robert Phillips, Michael A Piradov, Richie G. Poulton, Farshad Pourmalek, Mostafa Qorbani, Anwar Rafay, Mahfuzar Rahman, Mohammad HifzUr Rahman, Rajesh Kumar Rai, Sasa Rajsic, Annemarei Ranta, Salman Rawaf, Andre M.N. Renzaho, Mohammad Sadegh Rezai, Gholamreza Roshandel, Enrico Rubagotti, Perminder Sachdev, Saeid Safiri, Ramesh Sahathevan, Mohammad Ali Sahraian, Abdallah M. Samy, Paula Santalucia, Itamar S Santos, Benn Sartorius, Maheswar Satpathy, Monika Sawhney, Mete I Saylan, Sadaf G Sepanlou, Masood Ali Shaikh, Raad Shakir, Morteza Shamsizadeh, Kevin N Sheth, Mika Shigematsu, Haitham Shoman, Diego Augusto Santos Silva, Mari Smith, Eugene Sobngwi, Luciano A Sposato, Dan J Stein, Timothy J Steiner, Lars Jacob Stovner, Rizwan Suliankatchi Abdulkader, Cassandra EI Szoeke, Rafael Tabarés-Seisdedos, David Tanne, Alice M Theadom, Amanda G Thrift, David L Tirschwell, Roman Topor-Madry, Bach Xuan Tran, Thomas Truelsen, Kald Beshir Tuem, Kingsley Nnanna Ukwaja, Olalekan A Uthman, Yuri Y Varakin, Tommi Vasankari, Narayanaswamy Venketasubramanian, Vasiliy Victorovich Vlassov, Fiseha Wadilo, Tolassa Wakayo, Mitchell T Wallin, Elisabete Weiderpass, Ronny Westerman, Tissa Wijeratne, Charles Shey Wiysonge, Minyahil Alebachew Woldu, Charles D A Wolfe, Denis Xavier, Gelin Xu, Yuichiro Yano, Hassen Hamid Yimam, Naohiro Yonemoto, Chuanhua Yu, Zoubida Zaidi, Maysaa El Sayed Zaki, Joseph R Zunt, Christopher J L Murray, Theo Vos

## Abstract

**Background:**

Comparable data on the global and country-specific burden of neurological disorders and their trends are crucial for health-care planning and resource allocation. The Global Burden of Diseases, Injuries, and Risk Factors (GBD) Study provides such information but does not routinely aggregate results that are of interest to clinicians specialising in neurological conditions. In this systematic analysis, we quantified the global disease burden due to neurological disorders in 2015 and its relationship with country development level.

**Methods:**

We estimated global and country-specific prevalence, mortality, disability-adjusted life-years (DALYs), years of life lost (YLLs), and years lived with disability (YLDs) for various neurological disorders that in the GBD classification have been previously spread across multiple disease groupings. The more inclusive grouping of neurological disorders included stroke, meningitis, encephalitis, tetanus, Alzheimer's disease and other dementias, Parkinson's disease, epilepsy, multiple sclerosis, motor neuron disease, migraine, tension-type headache, medication overuse headache, brain and nervous system cancers, and a residual category of other neurological disorders. We also analysed results based on the Socio-demographic Index (SDI), a compound measure of income per capita, education, and fertility, to identify patterns associated with development and how countries fare against expected outcomes relative to their level of development.

**Findings:**

Neurological disorders ranked as the leading cause group of DALYs in 2015 (250·7 [95% uncertainty interval (UI) 229·1 to 274·7] million, comprising 10·2% of global DALYs) and the second-leading cause group of deaths (9·4 [9·1 to 9·7] million], comprising 16·8% of global deaths). The most prevalent neurological disorders were tension-type headache (1505·9 [UI 1337·3 to 1681·6 million cases]), migraine (958·8 [872·1 to 1055·6] million), medication overuse headache (58·5 [50·8 to 67·4 million]), and Alzheimer's disease and other dementias (46·0 [40·2 to 52·7 million]). Between 1990 and 2015, the number of deaths from neurological disorders increased by 36·7%, and the number of DALYs by 7·4%. These increases occurred despite decreases in age-standardised rates of death and DALYs of 26·1% and 29·7%, respectively; stroke and communicable neurological disorders were responsible for most of these decreases. Communicable neurological disorders were the largest cause of DALYs in countries with low SDI. Stroke rates were highest at middle levels of SDI and lowest at the highest SDI. Most of the changes in DALY rates of neurological disorders with development were driven by changes in YLLs.

**Interpretation:**

Neurological disorders are an important cause of disability and death worldwide. Globally, the burden of neurological disorders has increased substantially over the past 25 years because of expanding population numbers and ageing, despite substantial decreases in mortality rates from stroke and communicable neurological disorders. The number of patients who will need care by clinicians with expertise in neurological conditions will continue to grow in coming decades. Policy makers and health-care providers should be aware of these trends to provide adequate services.

**Funding:**

Bill & Melinda Gates Foundation.

## Introduction

In 2006, WHO emphasised the importance of neurological disorders (a group that at the time included epilepsy, Alzheimer's disease and other dementias, Parkinson's disease, multiple sclerosis, migraine, stroke, poliomyelitis, tetanus, meningitis, and Japanese encephalitis) for public health,^1^ and estimated that these disorders accounted for 6·3% of the global disability-adjusted life-years (DALYs). However, the WHO report was largely based on the Global Burden of Disease Study 2000 (GBD 2000)^2^ and did not reflect understanding of the comparative global epidemiology of neurological disorders. Increasing life expectancy and population growth worldwide in recent years mean that more people are now reaching ages where neurological disorders are most prevalent. Additionally, there have been changes in the treatment of some neurological disorders (such as the introduction of acute stroke units, thrombolysis, and thrombectomy), as well as changes in the risk factors that affect the burden of neurological disorders in the world.

**Research in context**

**Evidence before this study**

The Global Burden of Disease Study 2015 (GBD 2015) produced comprehensive and comparable estimates of the burden of various disorders (prevalence, deaths, and disability-adjusted life years [DALYs]) for 195 countries and territories from 1990 to 2015. However, aggregated estimates in the category of neurological disorders did not include data for stroke, brain and nervous system cancer, or communicable neurological disorders, which are all of great relevance to neurologists.

**Added value of this study**

For GBD 2015, the GBD 2013 search strategy was replicated to take into account all relevant epidemiological studies published between 2013 and 2015. In this systematic analysis, we produced estimates of the burden of neurological disorders (stroke, Alzheimer's disease and other dementias, Parkinson's disease, epilepsy, multiple sclerosis, migraine and tension-type headache, medication overuse headache, meningitis, tetanus, encephalitis, brain and nervous system cancers, motor neuron disease, and a residual category of other neurological disorders), separately and combined. For the first time, we included stroke, brain and nervous system cancer, and communicable neurological disorders. We quantified the global disease burden (as measured by prevalence, mortality, DALYs, years of life lost, and years lived with disability) and explored variation in the burden by neurological disorder type, age, sex, and overall country development level as measured by the Socio-demographic Index (SDI). The study showed that neurological disorders ranked as the leading cause of DALYs in 2015 and the second-leading cause of death. The most prevalent neurological disorders were headaches (tension-type headache, medication overuse headache, and migraine) and Alzheimer's disease and other dementias.

**Implications of all the available evidence**

Over the past 25 years, the burden of neurological disorders has increased substantially, and our expanded list of neurological disorders makes this the leading cause group of disability, and the second-leading cause group of mortality worldwide. Stroke remains the largest contributor to this burden globally. For all neurological disorders combined, there was a noticeable (by almost five times) gradual decrease in age-standardised DALY rates with increasing SDI, but these rates had large geographical variations. These findings could help to guide health systems and research activities to reduce the burden of neurological disorders.

To improve health-care planning and health outcomes of people with neurological disorders, we must understand not only the number and distribution of people with these disorders between countries, but also how these disorders affect population health (in terms of both mortality and disability) compared with other diseases and injuries.

The Global Burden of Disease Study 2015 (GBD 2015) GBD 2015^3^, ^4^ produced comprehensive and comparable estimates of the burden (prevalence, deaths, and DALYs) of 315 diseases and injuries for 195 countries and territories from 1990 to 2015. However, aggregated estimates in the category of neurological disorders did not include stroke, brain and nervous system cancer, or communicable neurological disorders, which are of great relevance to neurologists. Ischaemic and haemorrhagic stroke were classified as cardiovascular diseases; brain and nervous system cancer as malignant neoplasms; and meningitis, encephalitis, and tetanus as communicable diseases. In this systematic analysis, we quantify the disease burden (as measured by prevalence, mortality, DALYs, and years lived with disability [YLDs]) due to neurological disorders and its relationship with development level as measured by the Socio-demographic Index (SDI; an indicator based on lagged-distributed income per person, average educational attainment among individuals older than 15 years, and total fertility rate).

## Methods

### Categorisation of disorders

In an expanded category of neurological disorders we included stroke, Alzheimer's disease and other dementias, Parkinson's disease, epilepsy, multiple sclerosis, migraine, tension-type headache, medication overuse headache, meningitis, tetanus, encephalitis, brain and nervous system cancer, motor neuron disease, and a residual category of other neurological disorders that included diseases such as muscular dystrophy and Huntington's disease (appendix p 112).

### Non-fatal estimates

GBD 2015 non-fatal burden estimates were based on a systematic review of the literature to obtain all available epidemiological data on prevalence, incidence, risk of mortality, and severity. In the appendix we provide for each neurological disorder analysed the following: the list of International Classification of Diseases (ICD) codes used for mapping neurological causes of death (pp 118–21); a list of GBD sequelae, health states, health state lay descriptions, and disability weights for neurological disorders (pp 122–39); the total number of site-years by neurological cause and source type (p140); and the data representativeness index for each neurological disorder, the percentage of GBD 2015 geographies with any data by cause pertaining to the period before 2005, 2005–15, and all years of data (p 141). Reference case definitions were based on ICD-9 or ICD-10 criteria with the addition of Diagnostic and Statistical Manual of Mental Disorders (DSM)-III and DSM-IV criteria for dementia and the International Classification of Headache Disorders criteria for headaches.^5^, ^6^ Sources of information used to estimate the burden of neurological disorders are on the Global Health Data Exchange website.

Detailed GBD methods for calculating non-fatal estimates have been reported elsewhere.^4^ The epidemiological data were analysed with DisMod-MR 2.1,^7^ a Bayesian meta-regression tool that adjusts datapoints for variations in study methods between data sources and enforces consistency between data for different parameters, such as incidence and prevalence. For each neurological disorder, we defined a parsimonious set of sequelae that best described different aspects of the disabling consequences. Each non-fatal sequela was estimated separately. We calculated the YLDs caused by the residual category of other neurological disorders indirectly using a ratio of YLDs to years of life lost (YLLs). We calculated the ratio of YLDs to YLLs for Alzheimer's disease and other dementias, Parkinson's disease, multiple sclerosis, and motor neuron disease, and multiplied this ratio by the YLL estimates for other neurological disorders. Further details of the non-fatal estimates of each of the included neurological disorders are in the appendix (pp 5–113).

Disability weights for a set of 235 health states covering all sequelae of disease and injury estimated in GBD 2015 were estimated by pair-wise comparison methods presenting pairs of lay health state descriptions to respondents in surveys done in the general population in nine countries, and an open web-based survey.^8^ The sequelae of the neurological disorders included in this analysis each map to a unique health state with a corresponding disability weight (appendix pp 122–39). YLDs were computed by multiplying the prevalence of each sequela by a disability weight and aggregating estimates for all sequelae for a disease.

We categorised countries by their overall development status level as determined by the SDI, classifying them in high, high–medium, medium, medium–low, and low SDI quintiles on the basis of values across the 1980–2015 period (appendix pp 142–46), as described in detail elsewhere.^9^ The average expected relationships between DALY rates and death rates from neurological disorders (individually and as a group) and SDI over the entire study period (1990–2015) across all geographies in males and females were estimated using spline regression. We also categorised countries into 21 GBD regions (appendix p 155).

### Causes of death

GBD 2015 uses a database of 14 236 site-years of vital registration, verbal autopsy (a method of determining cause of death in countries that have no functional vital registration system), and maternal and child death surveillance data, covering the period from 1980 to 2015.^9^ Trained interviewers administer a structured questionnaire to relatives of a deceased person about their symptoms preceding death. The underlying cause of death is inferred by computer algorithms or physician review of the autopsy interview. We estimated deaths for all neurological disorders apart from headaches, to which no deaths are assigned as the underlying cause. For each neurological cause except dementia, we used the GBD Cause of Death Ensemble model (CODEm) strategy.^10^, ^11^ CODEm applies mixed effects or spatiotemporal Gaussian process regression models to mortality rates or cause fractions in varying combinations with predictive covariates. The ensemble of models with best credentials on out-of-sample predictive validity tests was selected for each cause of death. YLLs were calculated by multiplying the number of deaths at each age by the standard life expectancy at that age.^12^ Results from CODEm for each disease were scaled to fit all-cause mortality estimates derived from demographic sources by location, age, year, and sex.

We decided to use a natural history model for dementia because of a large inconsistency between the data for prevalence and mortality. For instance, in the USA, the rates of death from dementia increased five times between 1990 and 2014, whereas the available prevalence and incidence data showed no significant changes over the same period. Large increases in death rates assigned to dementia have also occurred in some other countries with high-quality vital registration systems. Furthermore, in GBD 2015, the prevalence of dementia varied among 187 countries by a factor of three, whereas dementia death rates varied by more than 20 times.^13^ The likely explanation was a change in coding practices between countries and within countries over time. To correct for this source of measurement bias, we assessed the most recent data from 23 high-income countries with high-quality vital registration systems and the highest ratio of registered dementia death rates to prevalent cases. This ratio is equivalent to the excess rate of mortality in cases of dementia. We derived a pooled estimate by age and sex using a linear regression of the log of these rates. We added these estimates as data in DisMod-MR 2.1 to derive estimates of cause-specific mortality rates that were consistent with prevalence data and the pooled estimate of excess mortality from the 23 countries that in their most recent year of vital registration were most willing to code a death to dementia as the underlying cause.^10^

### Compilation of results

DALYs were computed as the sum of YLLs and YLDs for each country, age, sex, and year with 95% uncertainty intervals (UIs) based on the 25th and 975th values of the ordered 1000 draws. Unless explicitly mentioned otherwise, all rates were age-standardised using the GBD standard.^10^ This study is compliant with the Guidelines for Accurate and Transparent Health Estimates Reporting (GATHER; appendix pp 114–17).^14^, ^15^

### Role of the funding source

The funder of the study had no role in study design, data collection, data analysis, data interpretation, or writing of the report. The corresponding authors had full access to all the data in the study and had final responsibility for the decision to submit for publication.

## Results

### Global burden

In 2015, the neurological disorders included in this analysis caused 250·692 (95% UI 229·080 to 274·654) million DALYs, comprising 10·2% of global DALYs, and 9·399 (9·095 to 9·714) million deaths, comprising 16·8% of global deaths. We were unable to report the combined prevalence. Classifying stroke under neurological disorders rather than cardiovascular diseases made neurological disorders the largest cause group of DALYs and the second-largest behind cardiovascular diseases in terms of global deaths. Among the neurological disorders, stroke, migraine, meningitis, Alzheimer's disease and other dementias, and epilepsy each caused more than 10 million DALYs. The most prevalent neurological disorders were the various types of headache and Alzheimer's disease and other dementias (table 1).

**Table 1:** Global absolute numbers and age-standardised rates per 100 000 people for neurological disorders and percentage changes between 1990 and 2015

	**All-age numbers (thousands)**		**Age-standardised rate (per 100 000)**	
	2015	Change from 1990 to 2015	2015	Change from 1990 to 2015
**Tetanus**				
DALYs	3510 (3002 to 4503)	−86·3% (−88·1 to −83·3)	47 (40 to 61)	−87·4% (−89·0 to −84·9)
Deaths	57 (48 to 80)	−83·4% (−85·5 to −78·8)	1 (1 to 1)	−85·7% (−87·5 to −82·4)
Prevalence	209 (205 to 215)	10·6% (5·1 to 15·2)	3 (3 to 3)	−15·4% (−19·6 to −12·3)
**Meningitis**				
DALYs	25 395 (21 653 to 30 649)	−31·9% (−42·6 to −10·1)	342 (292 to 413)	−39·8% (−48·8 to −22·0)
Deaths	379 (323 to 445)	−25·2% (−35·6 to −4·2)	5 (5 to 6)	−38·5% (−45·9 to −23·7)
Prevalence	8734 (8321 to 9107)	27·9% (24·3 to 31·7)	120 (114 to 125)	−10·6% (−13·2 to −8·0)
**Encephalitis**				
DALYs	8453 (7669 to 9412)	−14·0% (−25·8 to −0·1)	115 (104 to 128)	−29·0% (−38·4 to −18·7)
Deaths	150 (138 to 167)	−3·4% (−15·4 to 10·9)	2 (2 to 2)	−29·6% (−38·1 to −20·1)
Prevalence	4316 (3146 to 5876)	13·5% (9·3 to 19·2)	59 (43 to 80)	−23·7% (−26·9 to −19·2)
**Stroke**				
DALYs	118 627 (114 862 to 122 627)	21·7% (17·8 to 25·7)	1777 (1721 to 1835)	−32·3% (−34·4 to −30·0)
Deaths	6326 (6175 to 6493)	36·4% (32·4 to 40·8)	101 (99 to 104)	−30·0% (−32·0 to −27·7)
Prevalence	42 431 (42 068 to 42 767)	59·2% (58·4 to 59·9)	627 (621 to 631)	−9·8% (−10·3 to −9·4)
**Alzheimer's disease and other dementias**				
DALYs	23 779 (20 118 to 27 886)	98·4% (95·0 to 101·9)	396 (334 to 464)	−5·7% (−7·2 to −4·2)
Deaths	1908 (1587 to 2229)	114·9% (111·1 to 119·6)	32 (27 to 38)	−3·4% (−4·8 to −1·4)
Prevalence	45 956 (40 179 to 52 656)	111·7% (109·1 to 114·2)	761 (663 to 876)	2·4% (1·7 to 3·2)
**Parkinson's disease**				
DALYs	2059 (1832 to 2321)	111·2% (102·4 to 118·1)	33 (30 to 37)	10·8% (6·5 to 14·3)
Deaths	117 (114 to 121)	149·8% (135·0 to 161·4)	2 (2 to 2)	22·6% (15·7 to 28·4)
Prevalence	6193 (5726 to 6777)	117·8% (113·2 to 122·8)	98 (90 to 107)	15·7% (13·3 to 18·3)
**Epilepsy**				
DALYs	12 418 (10 438 to 14 479)	2·5% (−5·7 to 11·2)	168 (141 to 195)	−22·5% (−28·2 to −16·8)
Deaths	125 (119 to 131)	18·9% (6·4 to 32·1)	2 (2 to 2)	−15·6% (−23·0 to −8·0)
Prevalence	23 415 (21 550 to 25 419)	39·2% (33·4 to 45·2)	320 (295 to 347)	1·9% (−2·1 to 6·1)
**Multiple sclerosis**				
DALYs	1234 (1033 to 1437)	42·4% (31·8 to 57·3)	17 (14 to 20)	−16·0% (−22·1 to −7·6)
Deaths	19 (17 to 20)	39·9% (24·9 to 61·6)	0 (0 to 0)	−21·4% (−29·1 to −10·8)
Prevalence	2012 (1866 to 2167)	59·0% (55·5 to 62·6)	28 (26 to 30)	−4·6% (−6·7 to −2·5)
**Migraine**				
DALYs	32 899 (20 295 to 48 945)	49·5% (46·8 to 52·3)	439 (271 to 654)	0·5% (−0·5 to 1·5)
Deaths	··	··	··	··
Prevalence	958 789 (872 109 to 1 055 631)	49·6% (46·9 to 52·3)	12 799 (11 651 to 14 065)	0·1% (−0·9 to 1·1)
**Tension-type headache**				
DALYs	2261 (1055 to 4193)	49·2% (46·0 to 52·5)	30 (14 to 56)	0·2% (−1·0 to 1·4)
Deaths	··	··	··	··
Prevalence	1 505 892 (1 337 310 to 1 681 575)	49·2% (46·1 to 52·5)	20 121 (17 924 to 22 442)	0·0 (−1·1 to 1·2)
**Medication overuse headache**				
DALYs	9165 (6089 to 13081)	57·7% (52·5 to 63·3)	124 (82 to 177)	−0·9% (−3·9 to 2·2)
Deaths	··	··	··	··
Prevalence	58 455 (50 835 to 67 364)	57·8% (52·6 to 63·3)	790 (690 to 907)	−1·2% (−4·2 to 1·8)
**Motor neuron disease**				
DALYs	910 (872 to 959)	56·1% (34·6 to 69·6)	13 (13 to 14)	−0·9% (−12·4 to 4·0)
Deaths	35 (34 to 37)	97·3% (73·9 to 103·6)	1 (1 to 1)	12·7% (0·5 to 15·9)
Prevalence	202 (190 to 216)	72·4% (69·2 to 75·2)	3 (3 to 3)	3·1% (1·5 to 4·7)
**Brain and nervous system cancer**				
DALYs	7624 (6975 to 8219)	37·5% (16·9 to 54·1)	106 (97 to 114)	−9·0% (−21·1 to 0·8)
Deaths	229 (210 to 245)	65·8% (42·2 to 78·2)	3 (3 to 4)	−0·4% (−13·5 to 6·4)
Prevalence	1205 (1102 to 1323)	60·2% (31·7 to 87·2)	17 (16 to 19)	8·9% (−7·2 to 24·0)
Other neurological disorders*				
DALYs	2360 (2217 to 2565)	31·7% (19·1 to 40·5)	34 (31 to 36)	−9·4% (−15·8 to −5·7)
Deaths	54 (53 to 58)	53·1% (42·9 to 58·4)	1 (1 to 1)	−5·2% (−10·5 to −1·9)
**All neurological disorders**				
DALYs	250 692 (229 080 to 274 654)	7·4%†	3639 (3342 to 3964)	−29·7%†
Deaths	9399 (9095 to 9714)	36·7%†	150 (145 to 155)	−26·1%†

Data are n or % with 95% uncertainty intervals (UIs). Prevalence is an aggregate of all sequelae for a condition. DALYs=disability-adjusted life-years.

* Includes muscular dystrophy, Huntington's disease, and other less common neurological disorders (appendix p 112).

† UIs were not calculated for aggregated estimates of percentage change as this was not built into the Global Burden of Disease Study 2015 computational machinery and would have required a large computational effort competing with demands of completing the Global Burden of Disease Study 2016.

### Global burden by sex and age

**Figure 1:**
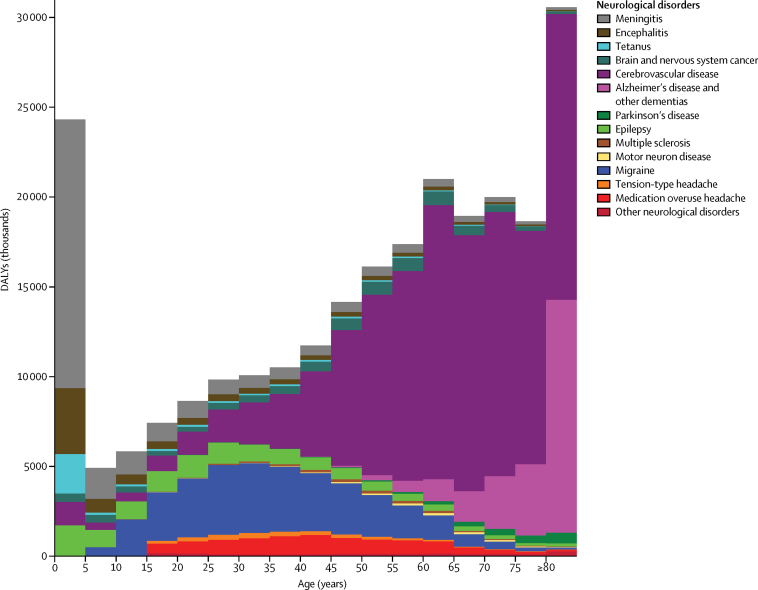
Global DALYs by age and neurological disorder in 2015 DALYs=disability-adjusted life-years.

Substantial sex differences in age-standardised death, DALY, and prevalence rates existed globally in 2015. The only neurological disorders with less than 10% difference between males and females were Alzheimer's disease and other dementias in terms of death and DALY rates, and meningitis and epilepsy in terms of prevalence rate. Apart from multiple sclerosis, Alzheimer's disease and other dementias, and the three headache types, DALY and prevalence rates were higher in males than females. All these more substantial sex differences were significant apart from those for meningitis, tetanus, and encephalitis death rates and the meningitis and encephalitis DALY rates (appendix pp 153–54). The age pattern varied between the various neurological disorders. The bulk of burden due to communicable neurological disorders occurred in young individuals, particularly the 0–5 years age group. Epilepsy caused the most burden in children and young adults. Headaches peaked at ages 25–49 years, whereas the burden of other neurological disorders increased with age (figure 1).

### Proportional contribution to the combined burden

**Figure 2:**
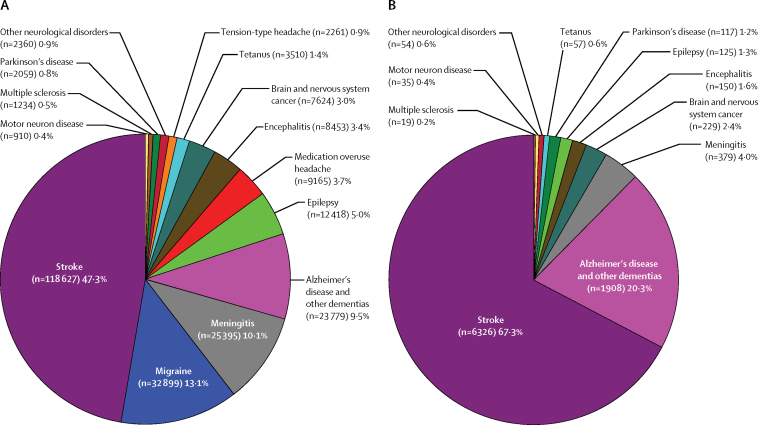
Contribution of various neurological disorders to the overall burden from neurological disorders in 2015 Estimates are for (A) disability-adjusted life-years and (B) deaths.

Stroke accounted for the largest proportion of total DALYs (47·3%) and deaths (67·3%) among all neurological disorders analysed (figure 2). Migraine, meningitis, and Alzheimer's disease and other dementias were the second, third, and fourth largest contributors of DALYs. The proportional contributions of the other neurological disorders analysed were less substantial and varied from 0·4% (for motor neuron disease) to 5·0% (epilepsy). The second largest contributor to deaths from neurological disorders was Alzheimer's disease and other dementias. The proportional contribution of deaths from other neurological disorders varied from 0·2% for (multiple sclerosis) to 4·0% (for meningitis; figure 2).

### Geographical variations in the burden

**Figure 3:**
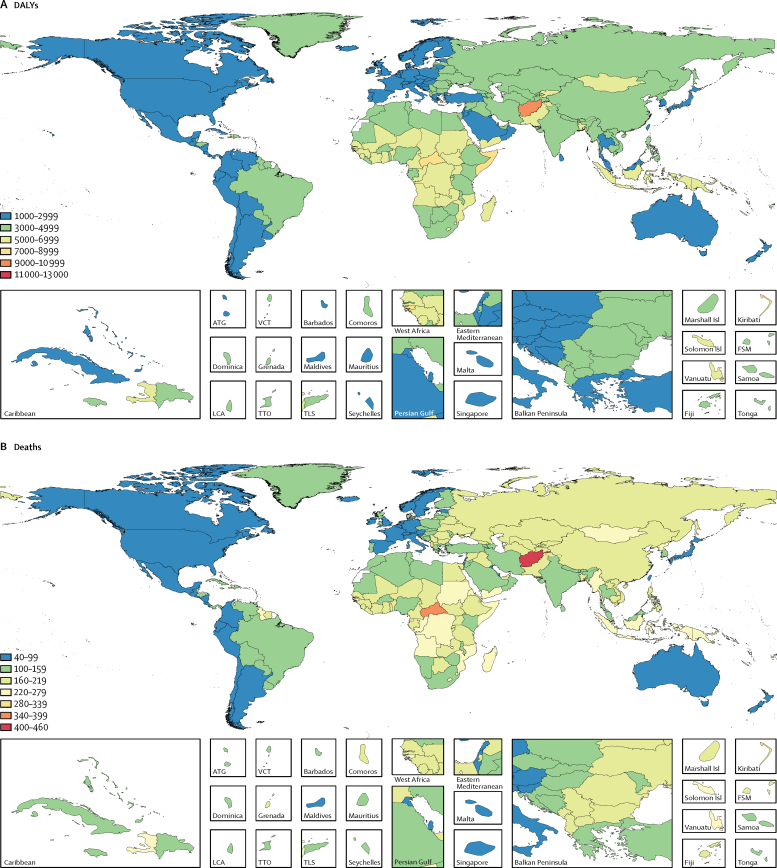
Age-standardised rates of (A) DALYs and (B) deaths per 100 000 people from all neurological disorders combined in 2015 Data are for both sexes. DALYs=disability-adjusted life-years. ATG=Antigua and Barbuda. VCT=Saint Vincent and the Grenadines. LCA=Saint Lucia. TTO=Trinidad and Tobago. TLS=Timor-Leste. Marshall Isl=Marshall Islands. Sol Isl=Solomon Islands. FSM=Federated States of Micronesia.

In 2015, the lowest age-standardised DALY rates (less than 3000 per 100 000 people) and death rates (less than 100 per 100 000 people) from neurological disorders were estimated for high-income regions and Latin America, whereas the highest DALY rates (more than 7000 per 100 000 people) were estimated for Afghanistan, Central African Republic, Guinea-Bissau, Kiribati, and Somalia. The highest death rates (more than 280 per 100 000 people) were estimated for Afghanistan and the Central African Republic (figure 3).

**Figure 4:**
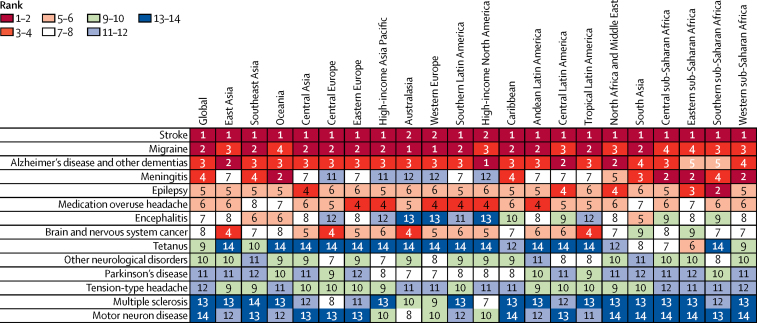
Ranking of age-standardised DALY rates for all neurological disorders by GBD region in 2015 Data are for both sexes. DALYs=disability-adjusted life-years.

Stroke was the leading cause of age-standardised DALY rates in 18 of 21 GBD regions, while migraine and Alzheimer's disease and other dementias were ranked among the top three causes in all regions except Oceania, south Asia, and the four sub-Saharan African regions, where epilepsy or meningitis ranked higher. Communicable neurological conditions ranked low in high-income regions and central Europe. Epilepsy and medication overuse headache ranked fourth, fifth, or sixth in almost all regions (figure 4).

A more detailed breakdown of geographical variation is shown as maps of age-standardised prevalence, death rates, and DALY rates for each neurological disorder (appendix pp 156–69). The prevalence of stroke was highest in eastern Europe, central Asia, Oceania, Indonesia, Myanmar, and sub-Saharan African countries. High death rates in Mongolia, Afghanistan, and Central African Republic were responsible for these countries also having the highest age-standardised DALY rates for stroke. Prevalence, death, and DALY rates for Alzheimer's and other dementias were highest in North America and north Africa and the Middle East, while the lowest rates were estimated for sub-Saharan Africa, southern Latin America, and Australasia. Prevalence and DALY rates for Parkinson's disease were highest in high-income regions and lowest in sub-Saharan Africa and eastern Europe. The strong positive relationship with latitude was apparent in the distribution of prevalence, death, and DALY rates for multiple sclerosis. High death rates due to epilepsy were the cause of high DALY rates in sub-Saharan Africa. Prevalence of epilepsy was highest in central Latin America, Chile, north Africa and the Middle East, and Bangladesh. Highest rates of motor neuron disease occurred in high-income regions.

The prevalence and DALY rates for the different types of headache varied by a factor of three to four between countries with the highest rates in high-income regions, north Africa and the Middle East, and tropical Latin America, while lowest rates were seen in sub-Saharan Africa and east Asia. Medication overuse headache was particularly common in eastern Europe and Iran. The highest death and DALY rates for brain and nervous system cancers were in central Europe and north Africa and the Middle East. Meningitis caused most disease burden in sub-Saharan Africa, especially in the meningitis belt across the Sahara. The largest burden from tetanus was estimated for a few countries in east Africa, particularly South Sudan and Somalia, which have low vaccination coverage owing to national conflict. India had the largest burden from encephalitis.

### Changes in the burden from 1990 to 2015

Time trends from 1990 to 2015 in prevalence, deaths, and DALYs due to neurological disorders varied across the disorders studied (table 1). Despite a significant drop in age-standardised stroke rates, population increase and ageing combined led to increases in absolute numbers of the three indicators. There was a large increase in the absolute numbers of DALYs, deaths, and prevalent cases of Alzheimer's disease and other dementias even though the changes in age-standardised rates were small. For Parkinson's disease, the numbers of DALYs, deaths, and prevalent cases increased substantially while increases in age-standardised rates were more modest. There was a significant decrease in epilepsy death and DALY rates while the age-standardised prevalence rate remained stable. DALYs, deaths, and prevalent cases of multiple sclerosis increased despite decreasing age-standardised rates. The absolute numbers of DALYs and prevalent cases of motor neuron disease, brain cancer, and headaches increased substantially while there was little change in age-standardised rates. The largest decreases were observed in communicable neurological disorders (tetanus, meningitis, and encephalitis). Although the rates of DALYs and deaths for all neurological disorders combined decreased by more than a quarter from 1990 to 2015, the absolute number of DALYs and deaths from all neurological disorders combined over that period increased by 7·4% (from 233·4 million to 250·7 million) and 36·7% (from 6·9 million to 9·4 million), respectively.

The decrease in DALY rates from 1990 to 2015 was estimated for most countries. The only countries with an increase in age-standardised DALY rates were the Philippines, Mongolia, Lesotho, Swaziland, and Zimbabwe, largely because these countries had an increase in DALYs from stroke over the 25-year period. The age-standardised DALY rates from neurological disorders dropped by more than half in Ethiopia, Equatorial Guinea, and South Korea (table 2).

**Table 2:** Global absolute number of DALYs and age-standardised rates per 100 000 people for all neurological disorders combined and percentage changes between 1990 and 2015 by country

		**All-age DALYs (thousands)**			**Age-standardised rate of DALYs (per 100 000)**		
		1990	2015	Change from 1990 to 2015*	1990	2015	Change from 1990 to 2015*
Afghanistan		1535 (1057 to 2053)	2071 (1667 to 2553)	34·9%	12 255 (9773 to 14 594)	10 669 (8798 to 12 830)	−12·9%
Albania		108 (97 to 120)	121 (108 to 135)	12·2%	4395 (4033 to 4804)	3691 (3287 to 4101)	−16·0%
Algeria		822 (707 to 961)	1107 (950 to 1267)	34·7%	4608 (4104 to 5162)	3462 (3021 to 3915)	−24·9%
Andorra		1 (1 to 2)	2 (2 to 3)	62·8%	2146 (1791 to 2536)	1766 (1435 to 2132)	−17·7%
Angola		871 (523 to 1284)	1067 (647 to 1956)	22·4%	8642 (4399 to 14 190)	6273 (3302 to 13 343)	−27·4%
Antigua and Barbuda		2 (2 to 2)	2 (2 to 3)	8·8%	4170 (3783 to 4612)	2866 (2516 to 3259)	−31·3%
Argentina		997 (908 to 1099)	977 (853 to 1118)	−2·0%	3310 (3029 to 3644)	2152 (1873 to 2470)	−35·0%
Armenia		108 (96 to 120)	108 (97 to 121)	0	3878 (3519 to 4253)	3101 (2782 to 3487)	−20·0%
Australia		448 (392 to 513)	572 (490 to 667)	27·6%	2529 (2209 to 2897)	1877 (1571 to 2237)	−25·8%
Austria		300 (270 to 336)	263 (227 to 303)	−12·5%	3022 (2661 to 3434)	2046 (1701 to 2431)	−32·3%
Azerbaijan		240 (219 to 266)	309 (274 to 350)	28·7%	4940 (4576 to 5348)	3619 (3242 to 4046)	−26·7%
Bahrain		10 (8 to 12)	21 (17 to 26)	110·8%	3231 (2800 to 3697)	2222 (1856 to 2627)	−31·2%
Bangladesh		6545 (5715 to 7383)	6416 (5551 to 7253)	−2·0%	7004 (6347 to 7682)	5344 (4667 to 5998)	−23·7%
Barbados		10 (9 to 11)	10 (9 to 11)	3·1%	3768 (3404 to 4168)	2760 (2411 to 3123)	−26·7%
Belarus		486 (444 to 529)	519 (474 to 572)	6·8%	4414 (4021 to 4831)	3906 (3536 to 4328)	−11·5%
Belgium		377 (341 to 420)	374 (328 to 426)	−0·6%	2954 (2624 to 3338)	2181 (1847 to 2562)	−26·2%
Belize		5 (4 to 5)	7 (6 to 9)	52·4%	3647 (3275 to 4045)	3055 (2681 to 3453)	−16·2%
Benin		330 (227 to 449)	431 (307 to 606)	30·7%	5984 (4947 to 7160)	5443 (3617 to 8060)	−9·0%
Bhutan		34 (25 to 44)	22 (18 to 26)	−34·8%	6145 (5110 to 7428)	3525 (2930 to 4167)	−42·6%
Bolivia		251 (217 to 299)	258 (219 to 302)	2·7%	4444 (3949 to 5024)	2941 (2514 to 3412)	−33·8%
Bosnia and Herzegovina		165 (148 to 183)	151 (135 to 169)	−8·8%	4323 (3931 to 4737)	2927 (2592 to 3313)	−32·3%
Botswana		33 (19 to 62)	61 (31 to 157)	84·3%	4495 (2344 to 8776)	4312 (2204 to 10667)	−4·1%
Brazil		4947 (4480 to 5482)	5874 (5149 to 6669)	18·7%	4988 (4619 to 5392)	3100 (2753 to 3480)	−37·9%
Brunei		5 (4 to 6)	8 (7 to 9)	57·6%	3414 (3080 to 3798)	2477 (2190 to 2796)	−27·4%
Bulgaria		570 (536 to 607)	458 (424 to 492)	−19·7%	5401 (5059 to 5782)	3870 (3531 to 4237)	−28·4%
Burkina Faso		647 (482 to 880)	750 (552 to 1010)	16·0%	5990 (4957 to 7341)	4633 (3380 to 6265)	−22·6%
Burundi		453 (337 to 573)	470 (341 to 644)	3·7%	10 962 (7640 to 14 124)	5627 (3938 to 7954)	−48·7%
Cambodia		536 (440 to 674)	468 (404 to 531)	−12·7%	7223 (6409 to 8176)	4512 (3938 to 5061)	−37·5%
Cameroon		595 (458 to 785)	865 (622 to 1200)	45·4%	5384 (4559 to 6359)	5148 (3530 to 7428)	−4·4%
Canada		724 (628 to 834)	984 (850 to 1130)	35·8%	2473 (2148 to 2844)	1999 (1685 to 2350)	−19·1%
Cape Verde		12 (10 to 14)	14 (12 to 16)	13·3%	4867 (4433 to 5327)	3577 (3086 to 4219)	−26·5%
Central African Republic		221 (173 to 269)	305 (197 to 439)	38·1%	9138 (7601 to 10697)	8838 (5307 to 13 092)	−3·3%
Chad		474 (349 to 634)	787 (555 to 1108)	66·2%	6416 (5233 to 7779)	6128 (4089 to 8876)	−4·5%
Chile		359 (318 to 406)	438 (382 to 501)	22·1%	3420 (3088 to 3784)	2223 (1926 to 2551)	−35·0%
China		45 845 (42 895 to 48 907)	49 486 (46 074 to 53 228)	7·9%	5529 (5208 to 5880)	3410 (3181 to 3658)	−38·3%
Colombia		826 (729 to 933)	949 (806 to 1112)	15·0%	3361 (3033 to 3710)	2244 (1928 to 2580)	−33·2%
Comoros		24 (18 to 31)	23 (17 to 30)	−5·0%	7384 (5104 to 10 202)	4378 (3200 to 5834)	−40·7%
Congo (Brazzaville)		117 (92 to 146)	166 (117 to 225)	41·4%	7457 (6021 to 9064)	5566 (3793 to 7795)	−25·4%
Costa Rica		59 (50 to 68)	93 (78 to 109)	58·6%	2594 (2272 to 2954)	1996 (1684 to 2335)	−23·1%
Cote d'Ivoire		594 (475 to 735)	848 (614 to 1130)	42·9%	5778 (4923 to 6704)	5377 (3750 to 7587)	−6·9%
Croatia		234 (218 to 253)	192 (175 to 210)	−18·0%	4309 (3986 to 4667)	2871 (2554 to 3217)	−33·4%
Cuba		324 (291 to 362)	379 (337 to 425)	16·7%	3295 (2976 to 3645)	2583 (2276 to 2944)	−21·6%
Cyprus		20 (18 to 22)	21 (18 to 25)	8·1%	2962 (2627 to 3321)	1922 (1593 to 2284)	−35·1%
Czech Republic		519 (483 to 561)	355 (315 to 400)	−31·7%	4376 (4039 to 4748)	2311 (2007 to 2657)	−47·2%
Democratic Republic of the Congo		1730 (1316 to 2183)	2976 (2197 to 3938)	72·0%	5934 (4390 to 7735)	5521 (3927 to 7317)	−7·0%
Denmark		204 (184 to 226)	184 (162 to 207)	−9·9%	3032 (2707 to 3425)	2286 (1967 to 2644)	−24·6%
Djibouti		27 (20 to 35)	34 (22 to 52)	26·7%	5845 (4301 to 7885)	5047 (3271 to 7902)	−13·7%
Dominica		2 (2 to 2)	2 (2 to 2)	7·1%	3479 (3103 to 3902)	3022 (2645 to 3436)	−13·1%
Dominican Republic		198 (174 to 222)	265 (231 to 301)	33·6%	3657 (3289 to 4039)	3038 (2691 to 3408)	−16·9%
Ecuador		246 (216 to 275)	326 (279 to 380)	32·8%	3266 (2917 to 3623)	2382 (2063 to 2734)	−27·1%
Egypt		2393 (2172 to 2607)	2696 (2395 to 3068)	12·6%	5267 (4828 to 5746)	3963 (3581 to 4494)	−24·8%
El Salvador		139 (124 to 156)	118 (99 to 139)	−15·0%	3516 (3166 to 3892)	2105 (1788 to 2461)	−40·1%
Equatorial Guinea		30 (17 to 47)	29 (18 to 55)	−3·5%	9305 (4456 to 15829)	4487 (2798 to 9079)	−51·8%
Eritrea		219 (161 to 281)	213 (146 to 314)	−3·0%	9041 (7575 to 10 688)	6369 (3831 to 9981)	−29·6%
Estonia		86 (79 to 92)	50 (44 to 56)	−42·1%	4813 (4451 to 5213)	2464 (2130 to 2836)	−48·8%
Ethiopia		4344 (2923 to 5858)	3387 (2546 to 4404)	−22·0%	9284 (7605 to 10967)	4637 (3343 to 6232)	−50·1%
Federated States of Micronesia		3 (3 to 4)	3 (2 to 4)	−15·6%	5884 (4146 to 7785)	4216 (3098 to 6093)	−28·4%
Fiji		24 (21 to 27)	28 (24 to 32)	15·7%	5429 (4749 to 6247)	3882 (3380 to 4409)	−28·5%
Finland		197 (178 to 219)	198 (175 to 224)	0·2%	3287 (2936 to 3678)	2309 (1980 to 2701)	−29·8%
France		1743 (1546 to 1966)	1901 (1658 to 2170)	9·1%	2493 (2169 to 2845)	1948 (1642 to 2305)	−21·8%
Gabon		44 (37 to 53)	53 (41 to 73)	20·4%	5763 (4886 to 6694)	4289 (3185 to 5984)	−25·6%
Georgia		289 (265 to 312)	222 (203 to 247)	−23·0%	5268 (4847 to 5707)	4236 (3825 to 4740)	−19·6%
Germany		3115 (2799 to 3455)	2748 (2400 to 3123)	−11·8%	2973 (2627 to 3343)	2041 (1711 to 2401)	−31·4%
Ghana		687 (549 to 853)	941 (644 to 1342)	36·9%	6309 (4812 to 8167)	5071 (3277 to 7649)	−19·6%
Greece		430 (394 to 470)	447 (403 to 496)	3·9%	3366 (3046 to 3735)	2365 (2058 to 2734)	−29·7%
Greenland		2 (2 to 2)	1 (1 to 2)	−20·7%	4929 (4464 to 5370)	3214 (2843 to 3616)	−34·8%
Grenada		4 (3 to 4)	3 (3 to 4)	−10·4%	4941 (4534 to 5365)	3714 (3350 to 4139)	−24·8%
Guatemala		191 (166 to 218)	304 (257 to 355)	59·6%	2874 (2531 to 3251)	2495 (2136 to 2865)	−13·2%
Guinea		558 (399 to 753)	550 (413 to 722)	−1·3%	7288 (5950 to 8810)	5719 (4215 to 7617)	−21·5%
Guinea–Bissau		87 (57 to 130)	121 (71 to 218)	40·0%	7587 (4479 to 13 190)	7463 (3972 to 15 833)	−1·6%
Guyana		32 (29 to 35)	27 (24 to 31)	−14·2%	7121 (6584 to 7631)	4805 (4309 to 5343)	−32·5%
Haiti		515 (441 to 624)	437 (358 to 525)	−15·0%	8584 (7695 to 9626)	5696 (4751 to 6682)	−33·6%
Honduras		224 (198 to 247)	210 (179 to 243)	−6·4%	4773 (4368 to 5208)	3530 (3022 to 4104)	−26·0%
Hungary		585 (545 to 626)	382 (343 to 424)	−34·7%	4697 (4357 to 5065)	2567 (2250 to 2906)	−45·3%
Iceland		7 (6 to 8)	8 (7 to 9)	16·4%	2607 (2286 to 2973)	1976 (1653 to 2345)	−24·2%
India		49 938 (45 989 to 54 561)	45 738 (40 949 to 51 065)	−8·4%	6590 (6135 to 7129)	4274 (3881 to 4706)	−35·2%
Indonesia		7770 (6393 to 9192)	9914 (8424 to 11268)	27·6%	5930 (5238 to 6639)	5115 (4375 to 5773)	−13·7%
Iran		1618 (1379 to 1855)	2033 (1664 to 2395)	25·7%	4596 (4042 to 5149)	3411 (2871 to 3950)	−25·8%
Iraq		640 (544 to 757)	1045 (875 to 1257)	63·2%	5733 (4939 to 6610)	4989 (4150 to 5944)	−13·0%
Ireland		101 (89 to 116)	111 (94 to 130)	9·3%	2811 (2446 to 3212)	2039 (1703 to 2412)	−27·4%
Israel		109 (96 to 125)	169 (143 to 199)	55·2%	2693 (2370 to 3069)	1968 (1642 to 2340)	−26·9%
Italy		2182 (1936 to 2447)	2378 (2058 to 2712)	9·0%	2991 (2611 to 3410)	2211 (1832 to 2637)	−26·1%
Jamaica		81 (73 to 89)	89 (79 to 101)	10·1%	4074 (3706 to 4489)	3249 (2873 to 3671)	−20·2%
Japan		3650 (3267 to 4087)	4489 (3999 to 5046)	23·0%	2587 (2302 to 2914)	1838 (1571 to 2163)	−29·0%
Jordan		76 (64 to 88)	129 (106 to 155)	69·9%	4125 (3579 to 4701)	2683 (2278 to 3104)	−35·0%
Kazakhstan		657 (605 to 720)	691 (623 to 765)	5·1%	5161 (4805 to 5562)	4518 (4102 to 4954)	−12·4%
Kenya		1143 (953 to 1509)	1427 (1222 to 1699)	24·9%	5323 (4608 to 6314)	4160 (3648 to 4684)	−21·9%
Kiribati		5 (4 to 6)	6 (5 to 7)	19·1%	8788 (7712 to 9853)	7420 (6390 to 8508)	−15·6%
Kuwait		30 (24 to 37)	59 (45 to 74)	94·5%	2447 (2078 to 2867)	2372 (1982 to 2802)	−3·1%
Kyrgyzstan		189 (174 to 205)	211 (190 to 234)	11·4%	6102 (5662 to 6542)	5062 (4661 to 5540)	−17·1%
Laos		327 (255 to 425)	221 (186 to 263)	−32·4%	8564 (7132 to 10059)	4735 (4058 to 5497)	−44·7%
Latvia		160 (150 to 171)	112 (103 to 122)	−29·9%	5136 (4784 to 5534)	3308 (2963 to 3682)	−35·6%
Lebanon		79 (67 to 91)	129 (104 to 155)	64·0%	3729 (3182 to 4297)	2421 (1969 to 2877)	−35·1%
Lesotho		45 (34 to 56)	79 (52 to 115)	74·2%	4296 (3155 to 5366)	6037 (3841 to 8956)	40·5%
Liberia		160 (115 to 224)	154 (115 to 212)	−4·0%	6601 (5212 to 8374)	4590 (3327 to 6043)	−30·5%
Libya		115 (99 to 131)	151 (128 to 176)	32·1%	3690 (3248 to 4146)	3324 (2880 to 3808)	−9·9%
Lithuania		142 (129 to 156)	132 (119 to 145)	−6·8%	3550 (3227 to 3926)	2843 (2522 to 3193)	−19·9%
Luxembourg		16 (15 to 18)	15 (13 to 18)	−4·8%	3480 (3113 to 3879)	2137 (1789 to 2551)	−38·6%
Macedonia		91 (84 to 99)	102 (93 to 112)	12·1%	5554 (5179 to 5963)	4160 (3765 to 4547)	−25·1%
Madagascar		648 (525 to 783)	925 (662 to 1286)	42·7%	7517 (6553 to 8475)	6186 (4189 to 8857)	−17·7%
Malawi		661 (472 to 950)	604 (452 to 810)	−8·6%	6512 (5245 to 8155)	4212 (3071 to 5691)	−35·3%
Malaysia		437 (389 to 484)	689 (592 to 789)	57·8%	4020 (3696 to 4359)	2925 (2555 to 3305)	−27·3%
Maldives		5 (4 to 6)	5 (4 to 6)	−0·2%	3712 (3308 to 4173)	2011 (1700 to 2359)	−45·8%
Mali		666 (498 to 885)	768 (570 to 1047)	15·4%	7318 (6215 to 8660)	5185 (3867 to 7169)	−29·2%
Malta		10 (9 to 11)	12 (10 to 13)	17·4%	2781 (2445 to 3159)	2007 (1686 to 2373)	−27·8%
Marshall Islands		2 (1 to 2)	2 (2 to 2)	28·2%	5588 (4953 to 6257)	4358 (3764 to 5009)	−22·0%
Mauritania		84 (63 to 112)	108 (81 to 140)	28·3%	5274 (4369 to 6368)	3664 (2624 to 4925)	−30·5%
Mauritius		34 (31 to 37)	36 (32 to 40)	5·8%	4922 (4593 to 5253)	2738 (2461 to 3056)	−44·4%
Mexico		1731 (1495 to 2002)	2414 (2048 to 2838)	39·5%	2772 (2441 to 3145)	2232 (1930 to 2577)	−19·5%
Moldova		201 (185 to 218)	165 (150 to 182)	−18·0%	5098 (4714 to 5483)	3592 (3263 to 3976)	−29·5%
Mongolia		96 (75 to 120)	136 (123 to 151)	40·9%	5738 (5020 to 6651)	6468 (5944 to 7197)	12·7%
Montenegro		28 (26 to 30)	32 (29 to 34)	12·6%	4875 (4486 to 5281)	3933 (3574 to 4319)	−19·3%
Morocco		962 (790 to 1143)	970 (796 to 1177)	0·8%	4956 (4362 to 5627)	3407 (2815 to 4100)	−31·3%
Mozambique		875 (690 to 1116)	1062 (746 to 1475)	21·4%	6921 (5890 to 8264)	5756 (3711 to 8423)	−16·8%
Myanmar		1912 (1439 to 2452)	2010 (1457 to 2662)	5·1%	6857 (4981 to 9046)	4991 (3657 to 6496)	−27·2%
Namibia		38 (31 to 44)	52 (38 to 72)	37·2%	4578 (3800 to 5351)	3426 (2455 to 4914)	−25·2%
Nepal		1702 (1339 to 2128)	904 (744 to 1066)	−46·9%	7684 (6469 to 8998)	3994 (3309 to 4713)	−48·0%
Netherlands		476 (423 to 536)	508 (444 to 577)	6·9%	2742 (2418 to 3106)	2131 (1821 to 2484)	−22·3%
New Zealand		92 (81 to 103)	108 (94 to 124)	17·4%	2625 (2331 to 2959)	1897 (1620 to 2215)	−27·8%
Nicaragua		94 (82 to 106)	109 (92 to 129)	16·7%	3076 (2723 to 3465)	2312 (1980 to 2655)	−24·8%
Niger		1259 (801 to 2018)	1267 (883 to 1848)	0·7%	9958 (7256 to 14427)	5869 (4344 to 7786)	−41·1%
Nigeria		5744 (4238 to 7818)	5694 (4390 to 7992)	−0·9%	5747 (4458 to 7261)	3618 (2864 to 4930)	−37·1%
North Korea		703 (558 to 876)	1178 (973 to 1412)	67·7%	4955 (3984 to 6118)	4679 (3888 to 5567)	−5·6%
Norway		161 (146 to 178)	141 (124 to 160)	−12·3%	2864 (2565 to 3209)	2017 (1723 to 2343)	−29·6%
Oman		35 (29 to 43)	71 (57 to 88)	99·7%	3029 (2471 to 3652)	2516 (2140 to 2905)	−16·9%
Pakistan		6419 (5315 to 7793)	7283 (6255 to 8567)	13·5%	6131 (5327 to 7083)	5005 (4380 to 5716)	−18·4%
Palestine		51 (43 to 61)	96 (80 to 115)	88·6%	4369 (3718 to 5160)	3722 (3109 to 4323)	−14·8%
Panama		57 (50 to 65)	86 (73 to 100)	51·0%	3230 (2889 to 3597)	2423 (2080 to 2798)	−25·0%
Papua New Guinea		205 (154 to 269)	308 (223 to 438)	49·9%	7826 (5720 to 10186)	6275 (4550 to 8789)	−19·8%
Paraguay		109 (97 to 123)	167 (145 to 194)	52·7%	3882 (3513 to 4288)	3298 (2898 to 3786)	−15·1%
Peru		610 (533 to 689)	572 (471 to 686)	−6·2%	3511 (3123 to 3921)	2066 (1721 to 2442)	−41·2%
Philippines		1617 (1439 to 1781)	2883 (2621 to 3173)	78·3%	3885 (3591 to 4190)	3968 (3642 to 4326)	2·1%
Poland		1442 (1315 to 1580)	1351 (1206 to 1503)	−6·3%	3743 (3420 to 4100)	2541 (2227 to 2876)	−32·1%
Portugal		541 (504 to 581)	401 (357 to 450)	−25·8%	4501 (4160 to 4877)	2370 (2052 to 2748)	−47·3%
Qatar		9 (7 to 10)	32 (24 to 41)	270·0%	2970 (2543 to 3480)	2220 (1826 to 2683)	−25·3%
Romania		1224 (1139 to 1313)	1124 (1044 to 1208)	−8·2%	5039 (4687 to 5412)	3817 (3491 to 4175)	−24·3%
Russia		8313 (7738 to 8910)	8507 (7857 to 9176)	2·3%	5380 (5009 to 5772)	4220 (3851 to 4600)	−21·6%
Rwanda		460 (360 to 585)	378 (288 to 494)	−17·9%	8493 (7054 to 10073)	4485 (3236 to 6286)	−47·2%
Saint Lucia		5 (5 to 6)	6 (5 to 7)	10·3%	4773 (4383 to 5192)	3237 (2879 to 3632)	−32·2%
St Vincent and the Grenadines		3 (3 to 4)	3 (3 to 4)	0·1%	4181 (3761 to 4616)	3483 (3122 to 3851)	−16·7%
Samoa		4 (4 to 5)	4 (3 to 5)	−4·0%	4534 (3770 to 5412)	3109 (2547 to 3657)	−31·4%
São Tomé and Príncipe		5 (4 to 5)	5 (4 to 8)	19·0%	4788 (4119 to 5510)	4610 (3123 to 6711)	−3·7%
Saudi Arabia		317 (263 to 379)	555 (444 to 686)	75·1%	3332 (2859 to 3808)	2705 (2302 to 3158)	−18·8%
Senegal		397 (326 to 494)	541 (383 to 748)	36·0%	5637 (4846 to 6563)	5226 (3419 to 7715)	−7·3%
Serbia		488 (449 to 529)	476 (440 to 515)	−2·5%	5146 (4755 to 5572)	3739 (3408 to 4110)	−27·3%
Seychelles		2 (2 to 2)	2 (2 to 3)	9·6%	4015 (3678 to 4382)	2636 (2311 to 3005)	−34·4%
Sierra Leone		304 (220 to 453)	308 (228 to 413)	1·3%	6568 (4982 to 8508)	6096 (4473 to 8013)	−7·2%
Singapore		61 (54 to 69)	76 (65 to 88)	24·8%	2864 (2613 to 3145)	1612 (1367 to 1896)	−43·7%
Slovakia		200 (182 to 220)	176 (156 to 199)	−12·0%	3830 (3487 to 4200)	2535 (2225 to 2903)	−33·8%
Slovenia		76 (69 to 84)	64 (57 to 73)	−15·9%	3553 (3228 to 3903)	2057 (1758 to 2390)	−42·1%
Solomon Islands		13 (10 to 17)	21 (14 to 29)	57·3%	7670 (5517 to 10124)	6077 (4252 to 8631)	−20·8%
Somalia		603 (401 to 896)	682 (428 to 1121)	13·1%	9690 (5059 to 16652)	7720 (3831 to 14872)	−20·3%
South Africa		1110 (1004 to 1226)	1458 (1291 to 1636)	31·4%	4340 (3955 to 4753)	3549 (3176 to 3953)	−18·2%
South Korea		1501 (1382 to 1640)	1371 (1216 to 1555)	−8·7%	5331 (5028 to 5669)	2185 (1926 to 2496)	−59·0%
South Sudan		508 (321 to 771)	691 (423 to 1222)	36·0%	8134 (4561 to 14144)	6548 (3545 to 13257)	−19·5%
Spain		1387 (1249 to 1548)	1477 (1276 to 1697)	6·5%	2998 (2671 to 3385)	2006 (1685 to 2378)	−33·1%
Sri Lanka		389 (344 to 439)	469 (389 to 566)	20·5%	2748 (2476 to 3062)	2384 (1994 to 2865)	−13·3%
Sudan		984 (790 to 1194)	1347 (1095 to 1647)	36·9%	6558 (5444 to 7867)	5257 (4240 to 6467)	−19·8%
Suriname		14 (13 to 15)	18 (16 to 20)	30·6%	4520 (4132 to 4920)	3913 (3462 to 4393)	−13·4%
Swaziland		23 (18 to 29)	40 (25 to 61)	75·5%	4819 (3679 to 6099)	5399 (3167 to 8410)	12·0%
Sweden		300 (271 to 333)	286 (254 to 326)	−4·4%	2458 (2176 to 2803)	1896 (1622 to 2227)	−22·9%
Switzerland		222 (197 to 249)	227 (196 to 262)	2·2%	2554 (2236 to 2913)	1875 (1572 to 2224)	−26·6%
Syria		472 (398 to 543)	454 (390 to 523)	−3·8%	6029 (5346 to 6815)	3733 (3253 to 4249)	−38·1%
Taiwan (Province of China)		509 (463 to 559)	533 (461 to 613)	4·8%	3439 (3176 to 3733)	1831 (1590 to 2109)	−46·8%
Tajikistan		189 (171 to 208)	237 (207 to 269)	25·1%	5248 (4808 to 5675)	4394 (3942 to 4849)	−16·3%
Tanzania		1186 (970 to 1421)	1740 (1290 to 2368)	46·7%	5600 (4737 to 6441)	4364 (3016 to 6521)	−22·1%
Thailand		1482 (1332 to 1652)	2033 (1751 to 2339)	37·2%	3723 (3397 to 4099)	2679 (2313 to 3084)	−28·0%
The Bahamas		7 (6 to 8)	11 (9 to 12)	48·5%	4025 (3668 to 4457)	2858 (2501 to 3232)	−29·0%
The Gambia		40 (31 to 51)	56 (43 to 71)	38·5%	5025 (3647 to 6938)	4166 (3164 to 5411)	−17·1%
Timor–Leste		38 (24 to 52)	30 (24 to 38)	−19·1%	6251 (4945 to 7614)	3643 (2976 to 4343)	−41·7%
Togo		171 (142 to 215)	235 (174 to 308)	37·3%	5474 (4660 to 6395)	4902 (3506 to 6685)	−10·4%
Tonga		3 (2 to 3)	3 (2 to 3)	0·3%	3983 (3479 to 4540)	3252 (2775 to 3750)	−18·3%
Trinidad and Tobago		40 (36 to 44)	42 (38 to 48)	5·6%	4459 (4103 to 4845)	3114 (2760 to 3496)	−30·2%
Tunisia		259 (227 to 294)	317 (265 to 373)	22·5%	4332 (3877 to 4801)	3088 (2577 to 3633)	−28·7%
Turkey		2225 (1854 to 2660)	1970 (1693 to 2271)	−11·5%	4564 (3926 to 5239)	2778 (2408 to 3176)	−39·1%
Turkmenistan		128 (117 to 141)	188 (171 to 207)	46·8%	5312 (4926 to 5798)	4786 (4427 to 5184)	−9·9%
Uganda		1254 (962 to 1677)	1558 (1143 to 2088)	24·3%	7413 (5838 to 8909)	5386 (3665 to 7530)	−27·3%
Ukraine		2900 (2698 to 3116)	2476 (2267 to 2671)	−14·6%	4853 (4494 to 5242)	3676 (3327 to 4045)	−24·3%
United Arab Emirates		46 (37 to 57)	212 (168 to 263)	359·0%	4712 (3949 to 5507)	3647 (3072 to 4316)	−22·6%
UK		2274 (2057 to 2522)	2018 (1777 to 2307)	−11·3%	3065 (2722 to 3466)	2207 (1875 to 2600)	−28·0%
	England	1865 (1686 to 2070)	1651 (1453 to 1886)	−11·5%	3001 (2668 to 3390)	2149 (1825 to 2530)	−28·4%
	Northern Ireland	55 (49 to 61)	55 (48 to 63)	0·8%	3025 (2675 to 3415)	2261 (1933 to 2631)	−25·3%
	Scotland	235 (210 to 263)	203 (177 to 235)	−13·3%	3679 (3236 to 4186)	2708 (2270 to 3232)	−26·4%
	Wales	120 (109 to 132)	109 (96 to 123)	−8·9%	3081 (2746 to 3452)	2297 (1977 to 2690)	−25·4%
USA		6812 (6031 to 7722)	8776 (7741 to 9939)	28·8%	2447 (2154 to 2790)	2109 (1831 to 2432)	−13·8%
Uruguay		116 (106 to 127)	110 (99 to 122)	−4·8%	3503 (3196 to 3849)	2509 (2232 to 2840)	−28·4%
Uzbekistan		680 (609 to 750)	919 (817 to 1028)	35·1%	5064 (4627 to 5483)	4126 (3747 to 4541)	−18·5%
Vanuatu		6 (5 to 8)	10 (7 to 14)	64·7%	7084 (5199 to 9626)	5797 (4304 to 7946)	−18·2%
Venezuela		435 (382 to 498)	649 (551 to 755)	49·2%	3378 (3051 to 3753)	2556 (2208 to 2935)	−24·3%
Vietnam		2310 (1974 to 2691)	3179 (2588 to 3745)	37·6%	4775 (4164 to 5511)	3862 (3134 to 4523)	−19·1%
Yemen		615 (436 to 791)	851 (629 to 1175)	38·3%	6756 (4862 to 9201)	5734 (4079 to 8182)	−15·1%
Zambia		557 (425 to 738)	636 (493 to 823)	14·3%	6691 (5443 to 7948)	5755 (4199 to 7711)	−14·0%
Zimbabwe		282 (237 to 336)	451 (339 to 595)	60·0%	3629 (2993 to 4329)	4009 (2809 to 5567)	10·5%

Data are n or % with 95% uncertainty intervals (UI). For more details on disorder, country, year, age, and sex, see the Global Burden of Disase compare website https://vizhub.healthdata.org/gbd-compare/. DALYs=disability-adjusted life-years.

* UIs cannot be calculated for aggregated estimates of percentage changes.

### Global burden by SDI

**Figure 5:**
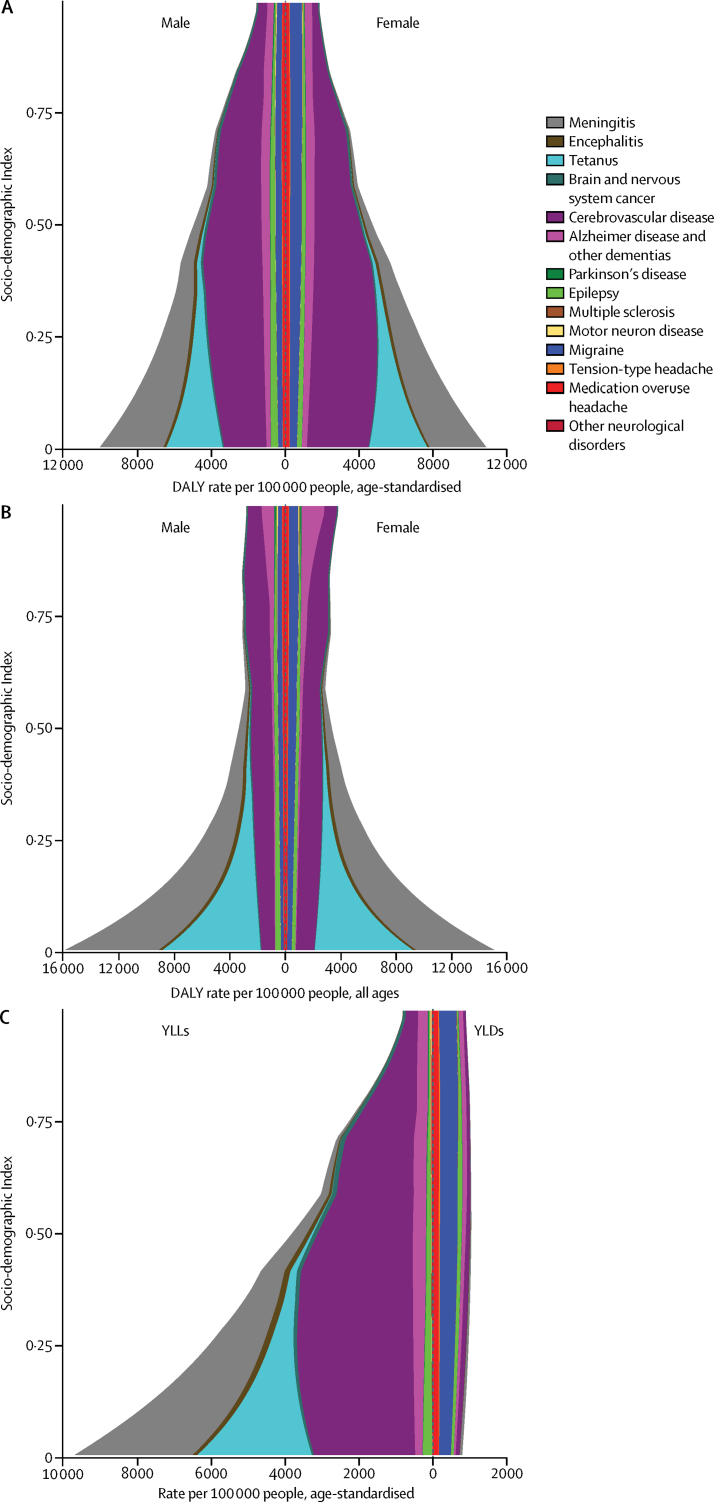
Expected relationship between the Socio-demographic Index and DALY rates for neurological disorders per 100 000 people between 1990 and 2015 Age-standardised disability-adjusted life-years (DALYs) per 100 000 people (A) by sex; (B) all-age DALY rate per 100 000 by sex; and (C) age-standardised rate per 100 000 by years of life lost (YLLs) and years lived with disability (YLDs).

The patterns of disease varied along the development spectrum as measured by the SDI (figure 5). Age-standardised DALY rates of communicable neurological disorders were the largest cause of DALYs at low levels of SDI. Stroke rates increased from low to middle levels of SDI and then decreased to their lowest values at the highest level of SDI. The headaches showed little change in rates with SDI. Rates of epilepsy gradually decreased with rising SDI (figure 5A). However, the all-age rates of combined neurological disorders increased from middle range to highest SDI values, particularly in females (figure 5B). Most of the changes in DALY rates of neurological disorders with development were driven by changes in YLLs. By comparison, the burden due to YLDs showed less variation over the range of SDI (figure 5C).

On the basis of SDI, Oceania, east and southeast Asia, eastern and central Europe, and central Asia had higher than expected age-standardised DALY rates for stroke for males over the entire estimation period. In females, the stroke DALY rates followed the same pattern but crossed the expected line in a few years of estimation. In females, the stroke DALY rates were initially higher than expected on the basis of SDI but fell to below expected levels during the study period. Latin America, apart from tropical Latin America in the 1990s, eastern and western sub-Saharan Africa, western Europe, Australasia, and high-income North America had lower DALY rates than expected based on SDI (appendix p 170). North Africa and the Middle East had higher than expected DALY rates for Alzheimer's disease and other dementias for males and females. Females in central sub-Saharan Africa, high-income North America, and tropical Latin America had DALY rates close to expected for their SDI values, while all other regions had much lower than expected rates (appendix p 171). Migraine DALY rates in males in south Asia were furthest above the expected line based on SDI. In females, migraine DALY rates in tropical Latin America, south Asia, Australasia, western Europe, and high-income North America were higher than expected. East Asia had much lower DALY rates of migraine than expected in males and females (appendix p 172). Epilepsy DALY rates were higher than expected in eastern, southern, and central sub-Saharan Africa and central Asia. Epilepsy rates were a bit lower than expected in Oceania and east Asia. The DALY rates for meningitis were higher than expected based on SDI in western sub-Saharan Africa. All other regions closely followed the expected pattern (appendix p 173). Plots of regional age-standardised DALY rates and SDI for the remaining neurological disorders are in the appendix (pp 174–83).

## Discussion

Neurological disorders including stroke, communicable neurological diseases, and brain cancer accounted for 10·2% of global DALYs and 16·8% of all deaths in 2015. DALYs from all neurological disorders combined exceeded those from all injuries (249·8 million), cardiovascular disease (228·9 million, excluding stroke), cancer (209·4 million), and mental and substance use disorders (162·4 million).^3^ Our study provides a comprehensive assessment of the extent, patterns, and trends of DALYs for the combined neurological disorders at global, regional, and national levels for 195 countries and territories with important implications for health policy, including priority-setting and financing of health services.

Despite an overall decrease in the age-standardised rates of DALYs, death, and YLDs of all neurological disorders combined between 1990 and 2015, the number of people dying from and affected by these disorders has increased substantially, contributing to higher health loss across the lifespan. Parkinson's disease was the only neurological disorder with increasing age-standardised rates of deaths, prevalence, and DALYs between 1990 and 2015. Although the burden of communicable neurological disorders has significantly decreased over this period, the burden of non-communicable neurological disorders has significantly increased. This finding is consistent with the overall global burden shift from communicable to non-communicable disorders.^16^ In terms of absolute number of people affected by neurological disorders, most of the increase in the burden was associated with ageing of the population and population growth.^9^ Increasing incidence of stroke in low-income and middle-income countries,^17^ increasing prevalence of multiple sclerosis,^18^, ^19^ increasing incidence of epilepsy in elderly people,^20^ increasing prevalence of tension-type headache,^21^ and increasing incidence of brain tumours in elderly people^22^ have been reported elsewhere. Findings from other studies have reported that it is difficult to assess trends in prevalence and incidence of Parkinson's disease because of changes in case definitions over time.^23^, ^24^ The evidence on secular trends in prevalence of migraine is mixed.^21^, ^25^

The conclusions from a systematic review^26^ of similar studies over time to examine trends in prevalence, incidence, and mortality for people with dementia were that there is some evidence that the incidence of dementia might be declining in high-income countries, but evidence on trends in the prevalence of dementia is inconsistent. Our study showed a modest increase in the prevalence of Alzheimer's disease and other dementias in high-income North America, high-income Asia Pacific, east Asia, south Asia, the Caribbean, and southern sub-Saharan Africa, and a modest decrease elsewhere. The small increase in the global age-standardised prevalence of Alzheimer's disease and other dementias is in contrast with a significant reduction from 1990 to 2015 in the prevalence of stroke, despite stroke sharing risks with, and contributing to, vascular dementia. For stroke, blood pressure control and smoking cessation have been important contributors to the reduction of its incidence in high-income and middle-income countries.^27^, ^28^, ^29^ Diverging trends of stroke and dementia incidence rates have been reported elsewhere.^30^ However, the most striking change has been the more than doubling of people in the world who die or are disabled from Alzheimer's disease and other dementias over the past 25 years. As ageing of the global population continues, our findings have important major health-service implications for the care of patients and adequate support for affected families.

Vaccinations have contributed to the favourable trends in the DALY rates of tetanus, meningitis, and encephalitis.^31^, ^32^, ^33^, ^34^, ^35^, ^36^, ^37^ Our estimate of 23·4 million cases of active epilepsy in 2015 is lower than the 32·7 million cases estimated in a meta-analysis of 65 prevalence studies, although that study did not specify a year of estimate.^38^ Similar to findings from this meta-analysis, we noted large geographical variations in the prevalence of epilepsy, with significantly greater rates in low-income and middle-income countries. The high prevalence of epilepsy in low-income and middle-income countries can partly be accounted for by the greater number of cases with communicable causes in these countries.^38^, ^39^ Our 2015 prevalence estimates are within the ranges reported for Parkinson's disease (51–177 per 100 000 people),^40^ motor neuron disease (1·9–3·9 per 100 000 people),^41^, ^42^ tension-type headache (21–27%),^43^, ^44^ medication overuse headache (0·5%–7·2%),^45^ and migraine (mean estimate 10%, range 1–25),^46^ but lower than that reported for multiple sclerosis (65–74 per 100 000 people),^47^ dementias (4·2–8·0% in people aged 60 years or older),^48^ and stroke (0·4–2·1% in low-income and middle-income countries).^49^ Differences in study populations (eg, age range and countries included) might account for some of the observed differences in prevalence rates.

Between countries, age-standardised rates of DALYs and deaths from neurological disorders as a group varied up to six times, with the highest rates in low-income to middle-income countries. These geographical patterns of the burden and distribution of individual neurological disorders are important for global and regional health-care planning and might inform further research to examine possible causes of the diseases. For example, the clear latitudinal gradient we noted in the prevalence of multiple sclerosis (about two times higher prevalence in countries at highest latitudes compared with those on the equator) corresponds to that observed in other studies^50^, ^51^ and is suggestive of the role of environmental factors (eg, vitamin D deficiency and infection).^52^, ^53^

Our findings of large geographical variations in the burden of stroke, dementias, Parkinson's disease, epilepsy, migraine, medication overuse headache, motor neuron disease, and brain and nervous system cancers concur with previous observations.^17^, ^24^, ^26^, ^38^, ^45^, ^46^, ^48^, ^54^, ^55^, ^56^, ^57^, ^58^ In non-communicable neurological disorders, the largest (greater than 20 times) variations in age-standardised DALYs were observed for stroke, motor neuron disease, and multiple sclerosis; in communicable neurological disorders, geographical differences ranged from 100 times for encephalitis to 10 000 times for tetanus. The greater geographical variation of communicable disorders was related to their overwhelming predominance in countries at low levels of socio-demographic development and emphasised the need for better prevention (including vaccination and sanitation measures), as well as better case management in these regions.

We noted significant sex differences in the burden of many neurological disorders analysed (higher prevalence of tetanus, stroke, Parkinson's disease, and motor neuron disease, but lower prevalence of multiple sclerosis and various types of headaches in males). The greater prevalence of stroke,^59^ Parkinson's disease,^24^ epilepsy,^60^ and motor neuron disease^61^ in males has also been reported by other studies. A greater prevalence in females has been reported elsewhere for migraine^62^ and multiple sclerosis.^50^ Our finding of 22% higher age-standardised prevalence of Alzheimer's disease and other dementias in women is in accordance with a finding from a meta-analysis of consistently higher estimates among 160 studies, although it was reported not to be significant.^48^ The higher age-standardised rate might be partly due to having a top age category of 80 years and older. As dementia is so highly prevalent at oldest ages, greater age detail in estimation of dementia prevalence might reduce the observed sex difference.

Our study findings have important health service implications. The large and increasing numbers of patients with neurological disorders necessitate careful planning by governments and other health-care providers to ensure adequate funding and staff for their treatment and rehabilitation services. However, a recent WHO–World Federation of Neurology survey^63^ of services and other resources for neurological disorders in 109 countries (90% of the world population) showed that there are large inequalities in access to neurological care across different populations, in particular for those living in low-income to middle-income countries. With a global shortage of neurologists, neurosurgeons, and rehabilitation professionals, improving neurological care will require innovative strategies within existing health systems. A good example of such innovative strategies is the use of nurses, nurse practitioners, and physician assistants trained in stroke care to care for patients with acute stroke and transient ischaemic attack in stroke units. A recently suggested implementation cycle for combating cardiovascular disease in low-income to middle-income countries^64^ provides a good template for similar interventional strategies for reducing the burden of neurological disorders in such countries. Although improving care and rehabilitation of people with neurological disorders is important for improving outcomes, there also are effective primary prevention strategies for communicable neurological disorders and stroke. However, proven effective preventive strategies are often underutilised.^65^, ^66^ More quality epidemiological research on risk factors, incidence, prevalence, and outcomes of neurological disorders in various countries is required to guide better prevention and management of these disorders, and our findings could help to prioritise such efforts. The sex, age, and regional and national differences and trends in the burden of neurological disorders necessitate the development, implementation, and prioritisation of treatments and preventive interventions that are specific for sex, age, and population to reduce the burden from these disorders.

Although ours was the most up-to-date overview of the global burden of major neurological disorders, this study was not free from some limitations in addition to overall GBD limitations.^3^, ^4^ First, we assumed that the excess mortality in Alzheimer's disease and other dementias implied by prevalence and mortality rates in countries that were most willing to code deaths to dementia in their vital registrations would apply to all other countries and periods. Although we realised that excess mortality was unlikely to be generalisable over location and time, we chose to make this assumption to address the much larger change we observed in deaths certified as Alzheimer's disease and other dementias between countries and over time than seen in prevalence or incidence studies. Second, estimates of cause-specific death rates in most low-income and middle-income countries rely on verbal autopsy data rather than physician-certified death records. Verbal autopsy instruments can identify deaths due to some neurological disorders, including stroke, meningitis, tetanus, and epilepsy, but are unable to capture other neurological disorders.^67^ Third, there are also many gaps in data availability by world region, and many low-income and middle-income regions do not have any epidemiological data. Epilepsy is the only neurological disorder with data sources for all 21 GBD regions. Fourth, heterogeneity in study methods and case definitions complicates the non-fatal estimation. Although we endeavoured to adjust for such methodological differences, this relied on generalising adjustment factors from few studies. Fifth, some categories of neurological disease were not included in this analysis because of an inability to aggregate cause-level and sequela-level data. For this reason, we were unable to include secondary epilepsy, the long-term neurological consequences of neonatal disorders, or traumatic brain injury and spinal cord injury, which are estimated in GBD as sequelae of injuries such as falls or road injuries. Adding currently missed neurological disorders would increase the health significance of the neurological disorders for public health systems and should be possible in a future GBD iteration. Sixth, sparse data on the severity of these neurological diseases did not allow us to differentiate severity by location or over time, with the exception of epilepsy (appendix pp 5–113). Relying on few studies, often from high-income countries only, meant we were unable to quantify any treatment effects on severity.

In conclusion, we have shown that neurological disorders are a large cause of disability and death worldwide. Globally, the burden of neurological disorders has increased substantially over the past 25 years because of population ageing, despite substantial decreases in mortality rates from stroke and communicable neurological disorders. Because low-income and middle-income countries still have a long way to go through the demographic transition of reductions in child mortality and population ageing, the number of patients who will need neurological care will continue to grow in the coming decades. It is important that policy makers and health-care providers are aware of these past trends to be able to provide adequate services for the growing numbers of patients with neurological disorders.

Correspondence to: Prof Valery L Feigin, National Institute for Stroke and Applied Neurosciences, School of Public Health and Psychosocial Studies, Faculty of Health and Environmental Studies, Auckland University of Technology, North Shore Campus, Auckland 1142, New Zealand **valery.feigin@aut.ac.nz**

and

Prof Theo Vos, Institute for Health Metrics and Evaluation, University of Washington, Seattle, WA 98121, USA **tvos@uw.edu**

For more on the **Global Health Data Exchange** see http://ghdx.healthdata.org/gbd-2015/data-input-sources
