# Supplementary material for: Global, regional, and national burden of neurological disorders during 1990–2015: a systematic analysis for the Global Burden of Disease Study 2015
Source: Lancet Neurol. 2017 Nov;16(11):877–97. doi: 10.1016/S1474-4422(17)30299-5 (PMC5641502; doi:10.1016/S1474-4422(17)30299-5)

THE LANCET
Neurology

Supplementary appendix

This appendix formed part of the original submission and has been peer reviewed. We post it as supplied by the authors.

Supplement to: GBD 2015 Neurological Disorders Collaborator Group. Global, regional, and national burden of neurological disorders during 1990–2015: a systematic analysis for the Global Burden of Disease Study 2015. *Lancet Neurol* 2017; published online Sept 17. http://dx.doi.org/10.1016/S1474-4422(17)30299-5.

Appendix: Global, regional, and national burden of neurological disorders during 1990–2015: a systematic analysis for the Global Burden of Disease study 2015

Preamble

This appendix provides further methodological detail and more detailed results for Global, regional, and national burden of neurological disorders during 1990-2015: a systematic analysis for the Global Burden of Disease study 2015. This study complies with the Guidelines for Accurate and Transparent Health Estimates Reporting (GATHER) recommendations. It includes detailed tables and information on data in an effort to maximize transparency in our estimation processes and provide a comprehensive description of analytical steps.

Table of Contents

Preamble 2

Fatal and non-fatal disease-specific estimation process 5

Tetanus estimation process 5

Meningitis estimation process 9

Encephalitis estimation process 18

Cerebrovascular disease estimation process 24

Alzheimer's disease and other dementias estimation process 32

Parkinson's disease estimation process 40

Epilepsy estimation process 49

Multiple sclerosis estimation process 55

Migraine estimation process 62

Tension-type headache estimation process 66

Medication overuse headache estimation process 70

Motor neuron disease estimation process 74

Cancer estimation process 83

Other neurological disorders estimation process 112

GATHER checklist of information that should be included in reports of global health estimates, with description of compliance and location of information for GBD 2015 paper on neurological disorders 114

Appendix Table 1: List of International Classification of Diseases (ICD) codes mapped to the Global Burden of Disease cause list for causes of death.......... 118

Appendix Table 2: GBD 2015 sequelae, health states, health state lay descriptions, and disability weights for neurological disorders 122

Appendix Table 3: Total number of site years of cause of death data by neurological cause and source type for 2015 140

Appendix Table 4: Data representativeness index (DRI), the percentage of GBD 2015 geographies with any data by cause, pertaining to periods before 2005–2015, and all years of data 141

Appendix Table 5: Socio-demographic Index quintiles for 195 countries and territories, based on their 2015 values 142

Appendix Table 6: Global all-age numbers and age-standardised rates in 2015 and percent change between 1990 and 2015 for DALYs, deaths, and prevalence by neurological disorder among females . 147

Appendix Table 7: Global all-age numbers and age-standardised rates in 2015 and percent change between 1990 and 2015 for DALYs, deaths, and prevalence by neurological disorder among males 150

Appendix Table S8. Global sex difference in age-standardised death, DALY, and prevalence rates by neurological disorder, 2015 153

Appendix Figure 1: Twenty-one GBD regions **155**

Appendix Figure 2: Geographical variations of the age-standardised prevalence rates per 100,000 from individual neurological disorders, both sexes, 2015.......... **156**

Appendix Figure 3: Geographical variations of the age-standardised DALY rates per 100,000 from individual neurological disorders, both sexes, 2015.......... **161**

Appendix Figure 4: Geographical variations of the age-standardised Death rates per 100,000 from individual neurological disorders, both sexes, 2015.......... **166**

Appendix Figure 5: Expected relationship between age-standardised DALY rates per 100,000 for individual neurological disorders by GBD regions, by sex, 1990–2015 .... **170**

Fatal Tetanus estimation process

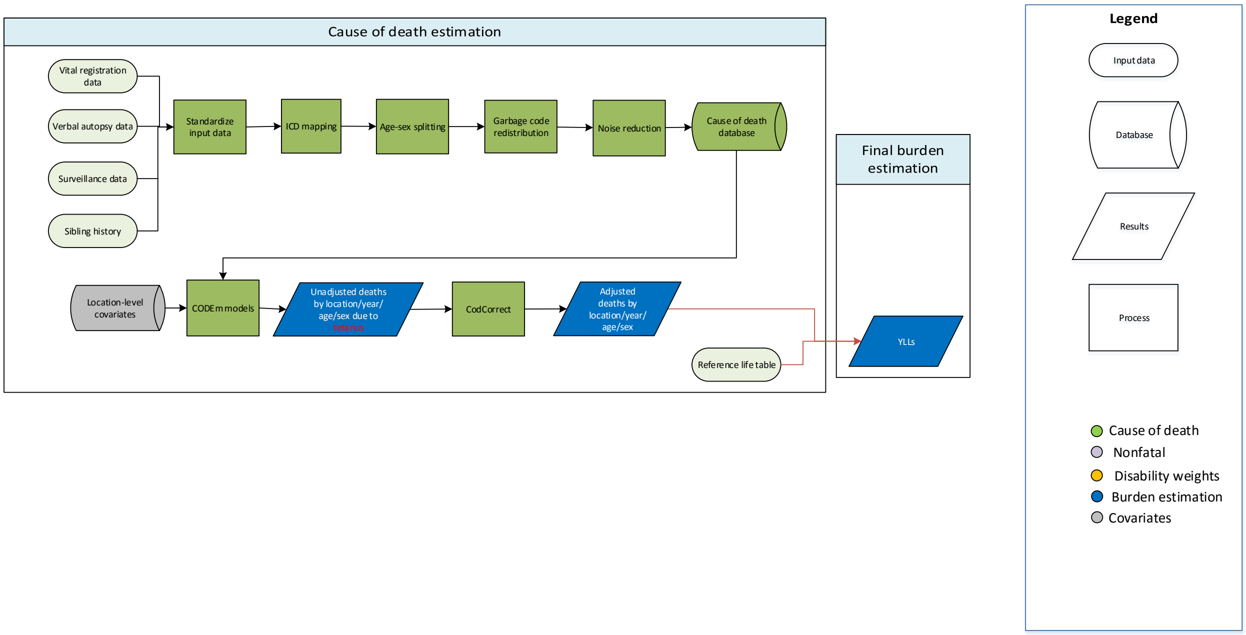

Input data

Vital registration, verbal autopsy, sibling history, and surveillance data were used. Data were outliered if they largely conflicted with the majority of data from other studies conducted either in the same or different countries with similar sociodemographic characteristics in the same region.

Modelling strategy

A general CODEm modelling strategy was used. We ran separate CODEm models for under 1 year and 1-80 years. There were no substantive changes from GBD 2013 in terms of modelling strategy.

Non-fatal Tetanus estimation process

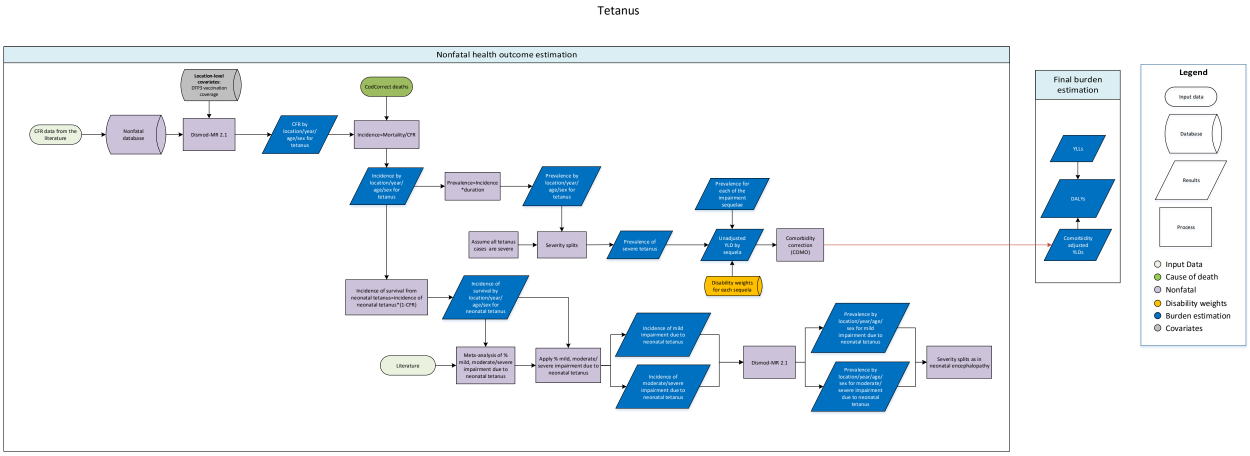

Case definition

Tetanus is a serious bacterial disease caused by the bacterium *Clostridium tetani.* For tetanus, the ICD 10 codes are A33-A35.0, Z23.5, and ICD 9 codes are 037-037.9,771.3,V03.7.

Input data

Model inputs

For GBD 2015, input data for the estimation of tetanus included case fatality data from the literature and IHME tetanus mortality estimates calculated with CODEm.

A systematic review was conducted for GBD 2013. The PubMed search terms were: (tetanus[Title/Abstract]) AND (case fatality[Title/Abstract]) AND ("2009"[Date - Publication] : "2013"[Date - Publication]).

Updates to systematic reviews are performed on an ongoing schedule across all GBD causes, and an update for tetanus will be performed in the next one to two iterations. The table below shows the number of literature studies included in GBD 2015 as well as the number of countries or subnational units and GBD world regions represented.

Table 1. Geographies

	Case fatality rate
Studies	49
Countries/subnationals	19

GBD world regions	12

Severity split & disability weight

We assume that all tetanus cases are severe episodes of acute infectious diseases. The lay descriptions and disability weights for tetanus derived from the GBD Disability Weights study are shown below.

Table 2. Severity splits, lay descriptions, and DWs

Severity level	Lay description	DW (95% CI)
Severe	Has a high fever and pain, and feels very weak, which causes great difficulty with daily activities.	0.133<br>(0.088–0.19)

Regarding the severity level of impairment due to neonatal tetanus, we assume the same distribution as in neonatal encephalopathy.

Modelling strategy

We used DisMod-MR 2.0 as a meta-regression tool to pool the case fatality data and generate location-year-age-sex-specific case fatality rate estimates. We used DTP3 coverage as a location-level covariate. Mortality was modelled using the standard CODEm tool on neonatal tetanus (ages 0–0.1) and non-neonatal tetanus (ages 1–80) separately for males and females. Incidence was then calculated as:

$$\text{incidence}=\text{mortality rate}/\text{case fatality rate}$$

Prevalence was then computed based on the estimated incidence and duration draws derived from literature review.

To estimate mild and moderate impairment due to neonatal tetanus, we first computed the incidence of survival from neonatal tetanus as:

$$\text{incidence of survival}= \text{incidence}*(1-\text{CFR})$$

We then conducted a meta-analysis of published studies to estimate the proportion of mild impairment due to neonatal tetanus and moderate-to-severe impairment due to neonatal tetanus. We applied these proportions to the estimated incidence of survival, to generate incidence of mild impairment due to neonatal tetanus and moderate-to-severe impairment due to neonatal tetanus, which were used as input data in DisMod 2.0. We ran two separate DisMod models (one for mild impairment due to neonatal

tetanus, and one for moderate-to-severe impairment due to neonatal tetanus) to generate age-sex-year-country-specific estimates.

The table below shows betas and exponentiated values for the covariates used in the estimation process (from the DisMod case-fatality model), which can be interpreted as an odds ratio.

Table 3. Beta and exponentiated beta values

Covariate	Parameter	Beta (95% CI)	Exponentiated beta (95% CI)
DTP3 coverage (proportion)	Case fatality	0.52 (-0.061–1.32)	1.68 (0.94–3.76)
Sex	Case fatality	-0.12 (-0.35–0.10)	0.89 (0.71–1.11)

No other significant changes were made to the modelling strategy for GBD 2015.

Fatal Meningitis estimation process

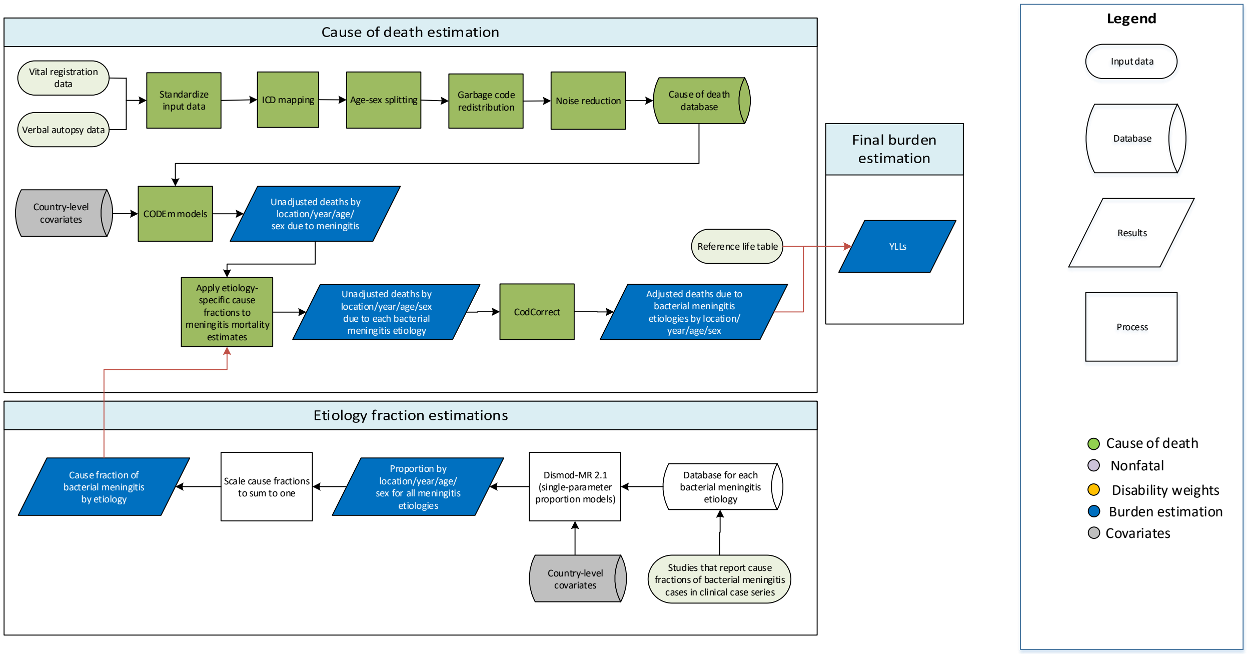

Input data

Input data for the all-meningitis model came from the cause of death database which includes vital registration and verbal autopsy data. We outliered data in instances where garbage code redistribution and noise reduction, in combination with small sample sizes, resulted in unreasonable cause fractions when compared to regional, super-regional, and global rates, and data that violated well-established time or age trends. Outliering methods were consistent across both vital registration and verbal autopsy data.

Input data that informed the aetiology splits came from a systematic review. In the GBD 2010 study, we conducted a systematic review of literature to capture studies of incidence for all four aetiologies of bacterial meningitis (meningococcal, pneumococcal, H influenza type B, and other bacterial meningitis). It was assumed that viral meningitis does not lead to mortality, therefore only deaths due to bacterial meningitis were considered. The inclusion criteria of the systematic review stipulated that (1) the publication year must be between 1980 and 2010; (2) “caseness” was based on diagnoses by antigen test, blood test, cerebrospinal fluid test, polymerase chain reaction test, or latex agglutination test; (3) sufficient information must be provided on study method and sample characteristics to assess the quality of the study; and (4) study samples must be representative of the general population. No limitation was set on the language of publication. For GBD 2013, the search strategy was replicated to capture epidemiological studies published between 2010 and 2013. This was repeated for GBD 2015 for studies published between 2013 and 2015, but only excess mortality was extracted. There were no bias corrections such as crosswalks or study-level covariates for the input data used for the aetiology splits.

Modelling strategy

We modelled deaths due to all bacterial meningitis with two CODEm models, separately for each sex and two age categories – under 5 and 5 years and above – because the mortality trends differ substantially between children and adults, and there are a significant number of data sources that only have data for under-5-year-olds. The two models used the same covariates and otherwise standard CODEm parameters. The final sex-specific models for deaths due to all bacterial meningitis were a hybridised model of separate global and data-rich models.

To obtain estimates for each of the four aetiologies of bacterial meningitis – meningococcal, pneumococcal, H influenza type B, and other bacterial – we ran four single-parameter proportion models using DisMod-MR 2.1. The meningococcal meningitis proportion model used two country-level covariates to inform the model – proportion of the population living within the meningitis belt, and proportion of the population covered by the meningococcal meningitis type A vaccine (an initiative called MenAfrivac). The pneumococcal meningitis model was informed by PCV3 coverage, and the H influenza type B meningitis model was informed by HIB3 coverage. The other bacterial meningitis proportion model did not use any country-level covariates. Since DisMod-MR 2.1 estimates in five-year intervals, the aetiological proportions for years between the intervals were interpolated at the draw level. Additionally, DisMod-MR 2.1 only produces estimates beginning in 1990, while cause of death estimates begin in 1980. Values at the draw level from 1990 were used for the years 1980–1989. The four proportion models were forced to sum to 1 at the draw level for each location, year, sex, and age combination. We applied these proportions to the all bacterial meningitis cause of death models to produce estimates for each of the four aetiologies assuming that the aetiological proportions derived from incidence studies apply equally to deaths.

Non-Fatal Meningitis estimation process

Meningitis

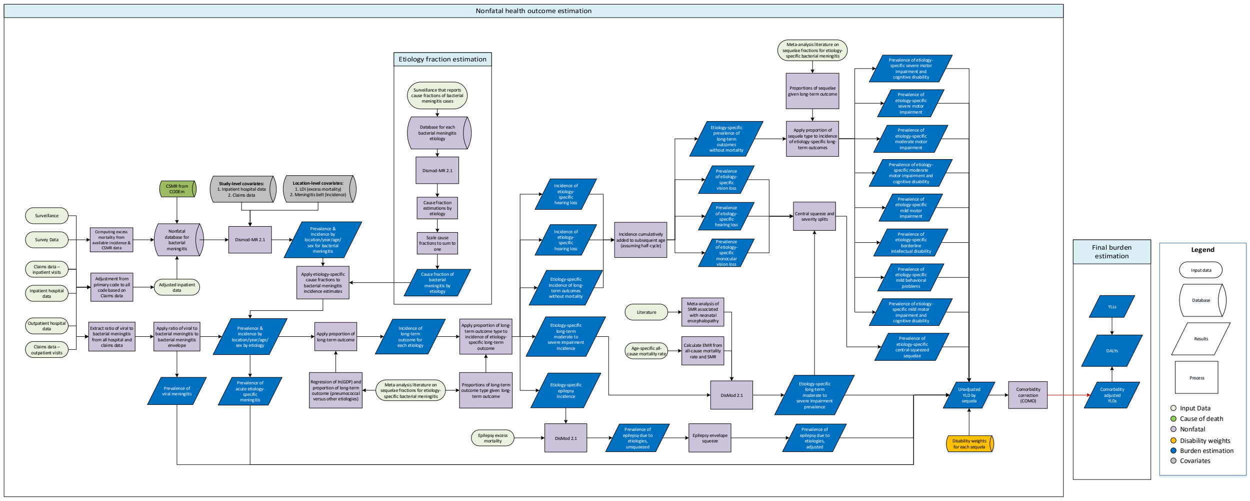

Case definition

Meningitis is a disease caused by inflammation of the meninges, the protective membrane surrounding the brain and spinal cord, and is typically caused by an infection in the cerebrospinal fluid. Symptoms include headache, fever, stiff neck, and sometimes seizures. Included in the GBD modelling were cases meeting ICD-10 diagnostic criteria for meningitis due to bacteria, viruses, or other causes (A39-A39.9, A87-A87.9, D86.81, G00.0-G00.8, G03-G03.8, Z20.811, and Z22.31) (1). In GBD 2015, meningitis encompasses viral meningitis and four bacterial aetiologies: pneumococcal, haemophilus influenza type B (HiB), meningococcal, and other.

Input data

Model inputs

In the GBD 2010 study, a systematic review of literature was conducted to capture studies of incidence and excess mortality rate for all bacterial meningitis cases. For each of the four aetiologies, literature included excess mortality rate, incidence, proportion, remission, and standardised mortality ratio. The inclusion criteria stipulated that (1) the publication year must be between 1980 and 2010; (2) “caseness” was based on diagnoses by antigen test, blood test, cerebrospinal fluid test, polymerase chain reaction test, or latex agglutination test; (3) sufficient information must be provided on study method and sample characteristics to assess the quality of the study; and (4) study samples must be representative of the general population. No limitation was set on the language of publication. For GBD 2013, the search strategy was replicated to capture epidemiological studies published between 2010 and 2013. The search strategy was repeated in 2015 only to capture excess mortality – updates to systematic reviews are

performed on an ongoing schedule across all GBD causes, and a complete update for meningitis will be performed in the next one to two iterations.

Additional sources we included in the acute bacterial meningitis model were inpatient-only hospital data and US claims data from 2000, 2010, and 2012, primary diagnosis and inpatient only. Sequelae and severity splits were informed by a meta-analysis, Edmond et al (2), while an internal meta-analysis informed mortality estimates for long-term moderate to severe impairments (3).

Data were outliered or excluded if we found them unreasonable when compared to regional, super-regional, and global rates.

The tables below show the number of studies included in GBD 2015, as well as the number of countries or subnational units and GBD world regions represented for the bacterial meningitis model and each model that informs the aetiology split.

Table 1a. Acute bacterial meningitis

	Prevalence	Incidence	Mortality risk
Studies	0	112	77
Countries/subnationals	0	189	182
GBD world regions	0	20	17

Table 1b. Pneumococcal meningitis proportion

	Proportion
Studies	67
Countries/subnationals	49
GBD world regions	18

Table 1c. Meningococcal meningitis proportion

	Proportion
Studies	62
Countries/subnationals	46
GBD world regions	17

Table 1d. H influenza type B meningitis proportion

	Proportion
Studies	68
Countries/subnationals	49
GBD world regions	18

Table 1e. Other bacterial meningitis proportion

	Proportion
Studies	60
Countries/subnationals	43

GBD world regions	16

Modelling strategy

Non-fatal outcomes were modelled using a combination of custom models and DisMod-MR 2.1, with minor changes from the GBD 2013 modelling process. First, the overall incidence and prevalence of bacterial meningitis were modelled to estimate the short-term morbidity due to acute infection. This DisMod model had a set duration (1/remission) of 4 weeks with a range ±2 weeks. We also imposed caps on excess mortality for neonates and elders based on the highest excess mortality estimates from GBD 2013. Hospital data were flagged with a covariate for inpatient hospital data, as were US claims data with year-specific covariates to be crosswalked to the reference data, which we extracted from literature. We used the function in DisMod-MR 2.1 to pull in cause-specific mortality rate (CSMR) data from our CODEm and CODcorrect analyses and match with prevalence data points for the same geography. We calculated excess mortality rate to estimate priors by dividing CSMR by prevalence, calculated from remission and incidence. To help inform trends where we lack data, we applied a country-level covariate for proportion of the population at the subnational and country levels that lives within the meningitis belt in sub-Saharan Africa. We forced a positive relationship, with a lower bound of 0 and an upper bound of 2. We also applied a lag-distributed income covariate to excess mortality, log transformed and forced negative with an upper bound of -0.1 and a lower bound of -1.

Incidence and prevalence of bacterial meningitis were split into four aetiologies (pneumococcal, meningococcal, *H. influenzae* type B, and other bacterial meningitis) using four proportion models run in DisMod-MR 2.1. Results from these models were squeezed to sum to 1 at the draw level for each location, year, age, and sex. We applied a Hib3 vaccine coverage covariate to the *H. influenzae* type B proportion model, the proportion of the population living in the meningitis belt covariate, and the proportion of the population living in areas covered by the MenAfrivac initiative (meningitis meningococcal type A) to the meningococcal meningitis proportion model, and a PCV3 coverage covariate to the pneumococcal meningitis model.

Data for viral meningitis were only available from hospitals or US claims data, and not from population studies, so incidence and prevalence of viral meningitis were extrapolated from bacterial meningitis incidence by applying age- and sex-specific ratios between bacterial and viral cases from a combination of hospital data and US claims data. In addition to short-term sequelae as a result of acute bacterial and viral meningitis, we also modelled the long-term outcomes from bacterial meningitis infection.

Sequelae splits

We first split the long-term sequelae among survivors of acute infection. We calculated the acute-phase survivors by applying the excess mortality (calculated by the acute meningitis DisMod model) to the incidence of each aetiology (excess mortality was converted to case fatality rate by $e^{(-\text{excess mortality} \times 1/(\text{excess mortality} + \text{remission}))}$. The survivors were then subject for long-term sequelae by applying the post-discharge proportions of health consequences calculated by a meta-analysis by Edmond et al (2). We calculated the

ratio of acute meningitis survivors that experience major long-term impairments for all aetiologies, and the ratio of minor impairments to major impairments for pneumococcal meningitis versus all other aetiologies (because pneumococcal meningitis showed significantly higher risk of morbidity than other aetiologies). This ratio was based off a regression of log-transformed GDP and ratio values from Edmonds et al. – this was different from last year, which used GNI. The regression is shown below:

$$y = -0.33590 \ln(GDP) + 1.15230$$

We used these two ratios to calculate the proportions of survivors who contract a long-term minor impairment and those who contract a long-term major impairment. The proportion with major impairments were further split (again using pooled proportions from Edmond et al) into specific major impairments, which were grouped into vision loss, hearing loss, moderate-to-severe cognitive impairments, and epilepsy.

The calculated incidence of long-term sequelae was then converted to prevalence by two different approaches. For the sequelae not associated with excess mortality, which were vision loss, hearing loss, intellectual disability, motor impairment, and behavioural problems, the incidence of each age was cumulatively added up to the subsequent age (assuming half-cycle) to construct prevalence at each age. If the sequela is associated with excess mortality (epilepsy and moderate-to-severe cognitive impairments), the calculated incidence was uploaded into DisMod together with the corresponding mortality parameters (excess mortality data from the epilepsy envelope DisMod model, and standardised mortality ratio data from a neonatal encephalopathy meta-analysis, converted to excess mortality using all-cause mortality estimates) to estimate the prevalence. Vision loss, hearing loss, and epilepsy estimates were squeezed and severity split centrally.

Disability weights

The basis of the GBD disability weight survey assessments are lay descriptions of sequelae highlighting major functional consequences and symptoms. The lay descriptions and disability weights for sequelae associated with each aetiology are shown below.

Table 5. Severity splits, lay descriptions, DWs

Severity split	Lay description	DW (95% CI)
Mild behaviour problems	This person is hyperactive and has difficulty concentrating, remembering things, and completing tasks.	0.045 (0.028–0.066)
Mild hearing loss	This person has great difficulty hearing and understanding another person talking in a noisy place (for example, on an urban street).	0.01 (0.004–0.019)

Mild hearing loss with ringing	This person is unable to hear and understand another person talking, even in a quiet place, is unable to take part in a phone conversation. Difficulties with communicating and relating to others cause emotional impact at times (for example worry or depression).	0.021 (0.012–0.036)
Moderate hearing loss	This person has great difficulty hearing and understanding another person talking in a noisy place (for example, on an urban street), and sometimes has annoying ringing in the ears.	0.027 (0.015–0.042)
Moderate hearing loss with ringing	This person is unable to hear and understand another person talking, even in a quiet place, is unable to take part in a phone conversation. Difficulties with communicating and relating to others often cause worry, depression, or loneliness.	0.074 (0.048–0.107)
Moderately severe hearing loss	Custom DW from hearing loss impairment envelope	
Severe hearing loss	This person is unable to hear and understand another person talking, even in a quiet place, and unable to take part in a phone conversation. Difficulties with communicating and relating to others cause emotional impact at times (for example worry or depression).	0.158 (0.105–0.227)
Profound hearing loss	This person is unable to hear and understand another person talking, even in a quiet place, is unable to take part in a phone conversation, and has great difficulty hearing anything in any other situation. Difficulties with communicating and relating to others often cause worry, depression, or loneliness.	0.204 (0.134–0.288)
Complete hearing loss	This person cannot hear at all in any situation, including even the loudest sounds, and cannot communicate verbally or use a phone. Difficulties with communicating and relating to others often cause worry, depression, or loneliness.	0.215 (0.144–0.307)

Severe hearing loss with ringing	This person is unable to hear and understand another person talking, even in a quiet place, is unable to take part in a phone conversation, and has annoying ringing in the ears for more than 5 minutes at a time, almost every day. Difficulties with communicating and relating to others cause emotional impact at times (for example worry or depression),	0.261 (0.175–0.36)
Profound hearing loss with ringing	This person is unable to hear and understand another person, even in a quiet place, is unable to take part in a phone conversation, has great difficulty hearing anything in any other situation, and has annoying ringing in the ears for more than 5 minutes at a time, several times a day. Difficulties with communicating and relating to others often cause worry, depression, or loneliness,	0.277 (0.182–0.387)
Complete hearing loss with ringing	This person cannot hear at all in any situation, including even the loudest sounds, and cannot communicate verbally or use a phone, and has very annoying ringing in the ears for more than half of the day. Difficulties with communicating and relating to others often cause worry, depression, or loneliness,	0.316 (0.212–0.435)
Moderate motor impairment	This person has some difficulty in moving around, and difficulty in lifting and holding objects, dressing and sitting upright, but is able to walk without help.	0.061 (0.04–0.089)
Moderate motor plus cognitive impairments	This person has some difficulty in moving around, holding objects, dressing and sitting upright, but can walk without help. This person has low intelligence and is slow in learning to speak and to do simple tasks.	0.203 (0.134–0.29)
Long-term mild motor impairment	This person has some difficulty in moving around but is able to walk without help.	0.01 (0.005–0.02)
Borderline intellectual disability	This person is slow in learning at school. As an adult, the person has some difficulty doing complex or unfamiliar tasks but otherwise functions independently.	0.011 (0.005–0.02)

Severe motor impairment	This person is unable to move around without help, and is not able to lift or hold objects, get dressed or sit upright.	0.402 (0.268–0.545)
Epilepsy	(combined DW)	NA
Blindness	Is completely blind, which causes great difficulty in some daily activities, worry and anxiety, and great difficulty going outside the home without assistance.	0.187 (0.124–0.26)
Severe acute episode of infectious disease	This person has a high fever and pain, and feels very weak, which causes great difficulty with daily activities.	0.133 (0.088–0.19)
Mild intellectual disability	This person has low intelligence and is slow in learning at school. As an adult, the person can live independently, but often needs help to raise children and can only work at simple supervised jobs.	0.043 (0.026–0.065)
Monocular distance vision loss	This person is blind in one eye and has difficulty judging distances	0.017 (0.009–0.029)
Mild motor plus cognitive impairments	This person has some difficulty in moving around but is able to walk without help. The person is slow in learning at school. As an adult, the person has some difficulty doing complex or unfamiliar tasks but otherwise functions independently.	0.031 (0.018–0.05)
Severe motor plus cognitive impairments	This person cannot move around without help, and cannot lift or hold objects, get dressed, or sit upright. The person also has very low intelligence, speaks few words, and needs constant supervision and help with all daily activities.	0.542 (0.37–0.702)

Fatal Encephalitis estimation process

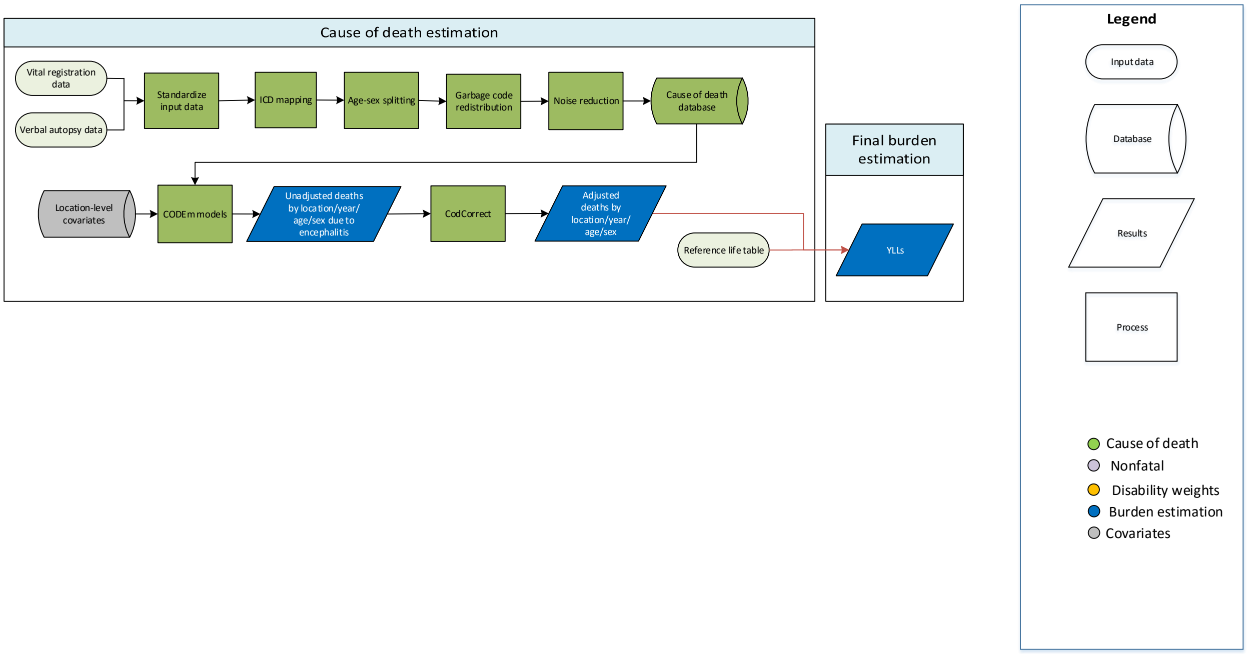

Input data

Vital registration and verbal autopsy data were used to model this cause. We outliered data in instances where garbage code redistribution and noise reduction, in combination with small sample sizes, resulted in unreasonable cause fractions when compared to regional, super-regional, and global rates, and data that violated well-established time or age trends. Outliering methods were consistent across both vital registration and verbal autopsy data.

Modelling strategy

We modelled deaths due to encephalitis with a standard CODEm model using the cause of death database and location-level covariates as inputs. We hybridised separate global and data-rich models to acquire unadjusted results, which were adjusted using CODCorrect to reach final years of life lost (YLLs) due to encephalitis.

In GBD 2013, the encephalitis model was modelled using two age categories – under 5 and 5 years and above – because the mortality trends differed substantially between children and adults and a significant number of data sources only had data for under-5-year-olds. With the addition of new data sources for GBD 2015, this modelling process was deemed unnecessary and the encephalitis model covered the entire age range. Another significant change was the addition of the Japanese encephalitis covariate, which is a binary covariate indicating if the location is known to be endemic for Japanese encephalitis. The covariate was modelled according to data from the Centers for Disease Control and Prevention.[1]

Reference

1 Centers for Disease Control (CDC). CDC health information for international travel 2016: the yellow book. New York City, United States: Oxford University Press, USA, 2016.

Non-fatal Encephalitis estimation process

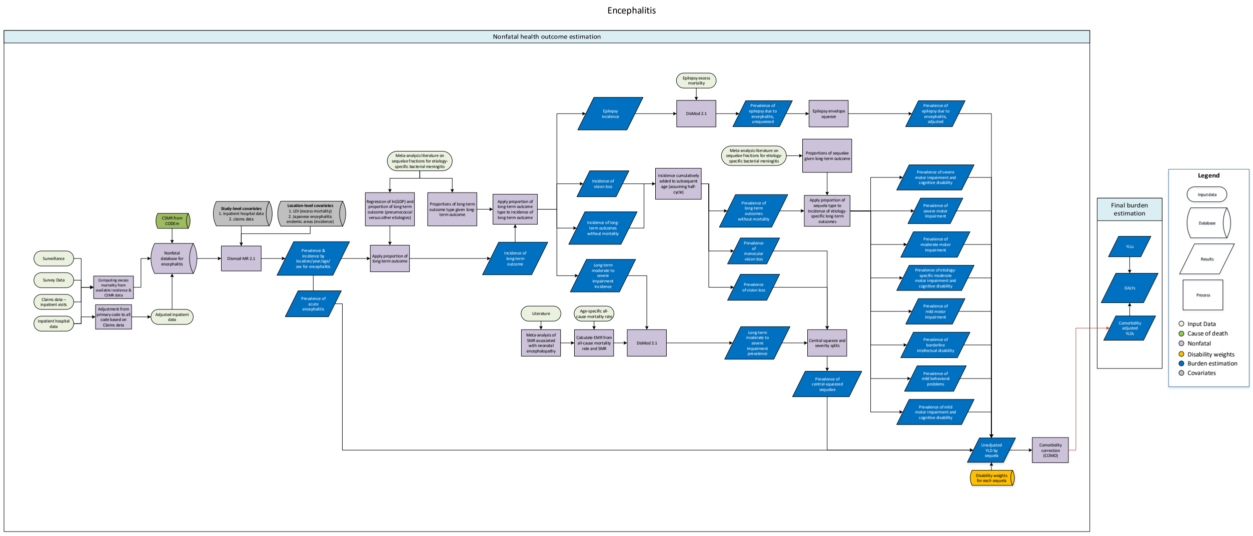

Case definition

Encephalitis is a disease caused by an acute inflammation of the brain. Symptoms of encephalitis can include flu-like symptoms like headache, fever, drowsiness, and fatigue, and at times, seizures, hallucinations, or stroke. Included in the GBD modelling were cases meeting ICD-10 diagnostic criteria for encephalitis (A83-A86.4, B94.1, F07.1, G04-G05.8) (1).

Input data

Model inputs

In the GBD 2010 study, a systematic review of literature was conducted to capture studies of incidence, excess mortality rate, remission, and standardised mortality ratio for encephalitis. These data sources included hospital data and literature. The inclusion criteria stipulated that (1) the publication year must be between 1980 and 2010; (2) sufficient information must be provided on study method and sample characteristics to assess the quality of the study; and (3) study samples must be representative of the general population. No limitation was set on the language of publication. For GBD 2015, the GBD 2010 search strategy was replicated to capture epidemiological studies published between 2010 and 2013.

Additional sources we included in the acute bacterial meningitis model were inpatient-only hospital data and US claims data from 2000, 2010, and 2012, primary diagnosis and inpatient only. Sequelae and severity splits were informed by a meta-analysis, Edmond et al (2), while an internal meta-analysis informed mortality estimates for long-term moderate-to-severe impairments (3).

Data were outliered or excluded if we found they differed significantly when compared to regional, super-regional, and global rates.

The tables below show the number studies included in GBD 2015, as well as the number of countries or subnational units and GBD world regions represented for the encephalitis.

Table 1. Acute encephalitis

	Prevalence	Incidence	Mortality risk
Studies	0	73	73
Countries/subnationals	0	154	154
GBD world regions	0	12	12

Modelling strategy

Non-fatal outcomes were modelled using a combination of custom models and DisMod-MR 2.1, with minor changes from the GBD 2013 modelling process. First, the overall incidence and prevalence of encephalitis were modelled to estimate the short-term morbidity due to acute infection. This DisMod model had a set duration (1/remission) of three weeks. We also imposed caps on excess mortality for ages 10–50. US claims data were flagged with year-specific covariates to be crosswalked to the reference data, which we extracted from literature and inpatient hospital data. We used the function in DisMod-MR 2.1 to pull in cause-specific mortality rate (CSMR) data from our CODEm and CODcorrect analyses and match with incidence data points for the same geography. We calculated excess mortality rate to estimate priors by dividing CSMR by prevalence, calculated from remission and incidence. To help inform trends where we lack data, we applied a binary country-level covariate at the subnational and country level that indicates if the location is in a Japanese Encephalitis endemic area (4). We forced a positive relationship, with a lower bound of 0 and an upper bound of 2. We also applied a lag-distributed income covariate to excess mortality, log transformed and forced negative with an upper bound of -0.1 and a lower bound of -1. Betas and exponentiated values (which can be interpreted as an odds ratio) are shown in the tables below for study-level covariates and country-level covariates.

Table 2. Study covariates

Study covariate	Parameter	beta	Exponentiated beta
Claims data – 2000	Incidence	-0.84 (-1.02 — -0.65)	0.43 (0.36 — 0.52)
Claims data – 2010	Incidence	-0.51 (-0.58 — -0.44)	0.60 (0.56 — 0.65)
Claims data – 2012	Incidence	-0.4 (-0.48 — -0.33)	0.67 (0.62 — 0.72)

Table 3. Country-level covariates

Country-level covariate	Parameter	beta	Exponentiated beta
Japanese Encephalitis endemic area	Incidence	0.92 (0.75 — 1.04)	2.51 (2.12 — 2.84)
LDI (log transformed)	Excess mortality	-0.18 (-0.22 — -0.088)	0.84 (0.80 — 0.92)

In addition to short-term sequelae as a result of acute encephalitis, we also modelled the long-term outcomes from encephalitis.

Sequelae splits

We first split the long-term sequelae among survivors of acute infection. We calculated the acute phase survivors by applying the excess mortality (calculated by the acute meningitis DisMod model) to the incidence of each aetiology (excess mortality was converted to case fatality rate by $e^{(-\text{excess mortality} \times 1/(\text{excess mortality} + \text{remission}))}$. The survivors were then subject to long-term sequelae by applying the post-discharge proportions of health consequences calculated by a meta-analysis by Edmond et al (2). We calculated the ratio of acute encephalitis survivors that result in a major long-term impairment, and the ratio of minor impairments to major impairments, based off a regression of log-transformed GDP and ratio values from Edmonds et al. This regression was done differently from last year, which previously used GNI. The regression is shown below:

$$y = -0.33590\ln(GDP) + 1.15230$$

We assumed a similar pattern of health outcomes for encephalitis infection survivors as with other bacterial meningitis survivors (except hearing loss, as we could not find evidence of hearing loss as a consequence of encephalitis infection). We used these two ratios to calculate the proportions of survivors who contract a long-term minor impairment and those who contract a long-term major impairment. The proportion with major impairments were further split (again using pooled proportions from Edmond et al) into specific major impairments, which were grouped into vision loss, moderate to severe cognitive impairments, and epilepsy.

The calculated incidence of long-term sequelae was then converted to prevalence by two different approaches. For the sequelae not associated with excess mortality, which were vision loss, intellectual disability, motor impairment, and behavioural problems, the incidence of each age was cumulatively added up to the subsequent age (assuming half-cycle) to construct prevalence at each age. If the sequela is associated with excess mortality (epilepsy and moderate-to-severe cognitive impairments), the calculated incidence was uploaded into DisMod together with the corresponding mortality parameters (excess mortality data from the epilepsy envelope DisMod model, and standardised mortality ratio data from a neonatal encephalopathy meta-analysis, converted to excess mortality using all-cause mortality estimates) to estimate the prevalence. Vision loss and epilepsy estimates were squeezed and severity split centrally.

Disability weights

The basis of the GBD disability weight survey assessments is lay descriptions of sequelae highlighting major functional consequences and symptoms. The lay descriptions and disability weights for sequelae associated with encephalitis are shown below.

Table 4. Severity splits, lay descriptions, and DWs

Severity split	Lay description	DW (95% CI)
Mild behaviour problems	This person is hyperactive and has difficulty concentrating, remembering things, and completing tasks.	0.045 (0.028–0.066)
Moderate motor impairment	This person has some difficulty in moving around, and difficulty in lifting and holding objects, dressing, and sitting upright, but is able to walk without help.	0.061 (0.04–0.089)
Moderate motor plus cognitive impairments	This person has some difficulty in moving around, holding objects, dressing and sitting upright, but can walk without help. This person has low intelligence and is slow in learning to speak and to do simple tasks.	0.203 (0.134–0.29)
Long-term mild motor impairment	This person has some difficulty in moving around but is able to walk without help.	0.01 (0.005–0.02)
Borderline intellectual disability	This person is slow in learning at school. As an adult, the person has some difficulty doing complex or unfamiliar tasks but otherwise functions independently.	0.011 (0.005–0.02)
Severe motor impairment	This person is unable to move around without help, and is not able to lift or hold objects, get dressed, or sit upright.	0.402 (0.268–0.545)
Epilepsy	(combined DW)	NA
Blindness	Is completely blind, which causes great difficulty in some daily activities, worry and anxiety, and great difficulty going outside the home without assistance.	0.187 (0.124–0.26)
Acute encephalitis	This person has a high fever and pain, and feels very weak, which causes great difficulty with daily activities.	0.133 (0.088–0.19)
Mild intellectual disability	This person has low intelligence and is slow in learning at school. As an adult, the person can live independently but often needs help to raise children and can only work at simple supervised jobs.	0.043 (0.026–0.065)

Monocular distance vision loss	This person is blind in one eye and has difficulty judging distances	0.017 (0.009–0.029)
Mild motor plus cognitive impairments	This person has some difficulty in moving around but is able to walk without help. The person is slow in learning at school. As an adult, the person has some difficulty doing complex or unfamiliar tasks but otherwise functions independently.	0.031 (0.018–0.05)
Severe motor plus cognitive impairments	This person cannot move around without help, and cannot lift or hold objects, get dressed or sit upright. The person also has very low intelligence, speaks few words, and needs constant supervision and help with all daily activities.	0.542 (0.37–0.702)

No other significant changes were made to the modelling process for GBD 2015.

Fatal Cerebrovascular Disease estimation process

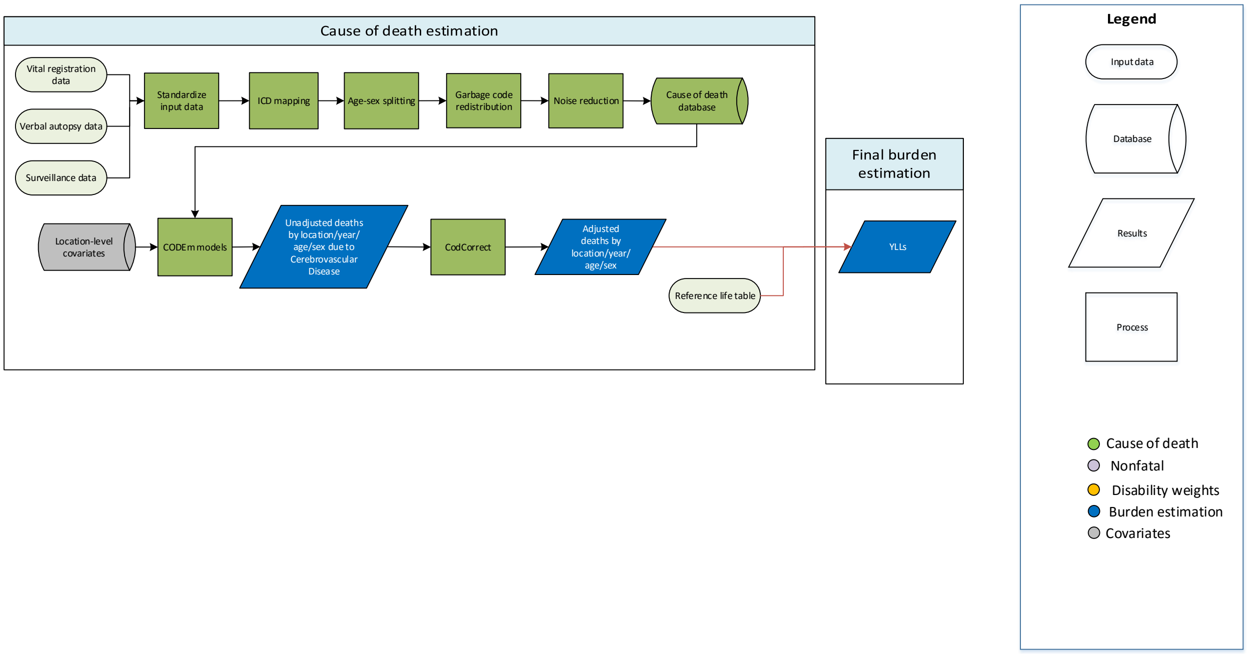

Input data

Verbal autopsy and vital registration data were used to model this cause. We outliered non-representative subnational verbal autopsy data points. We reassigned deaths from verbal autopsy reports for cerebrovascular disease to the parent cardiovascular disease for both sexes for those under 20 years of age. We also outliered ICD8, ICD9 BTL, and ICD10 Tabulated data points which were inconsistent with the rest of the data and created implausible time trends. Data points from sources which were implausibly low in all age groups and data points that were causing the regional estimates to be improbably high were outliered.

Modelling strategy

We used a standard CODEm approach to model deaths from cerebrovascular disease. We have included two new variables, Socio-demographic Index and the summary exposure value (SEV) scalar for cerebrovascular disease, as possible covariates for selection in the ensemble modelling process. Otherwise, there have been no substantive changes from the approach used in GBD 2013.

Non-fatal Cerebrovascular Disease, Ischemic Stroke, & Haemorrhagic Stroke estimation process

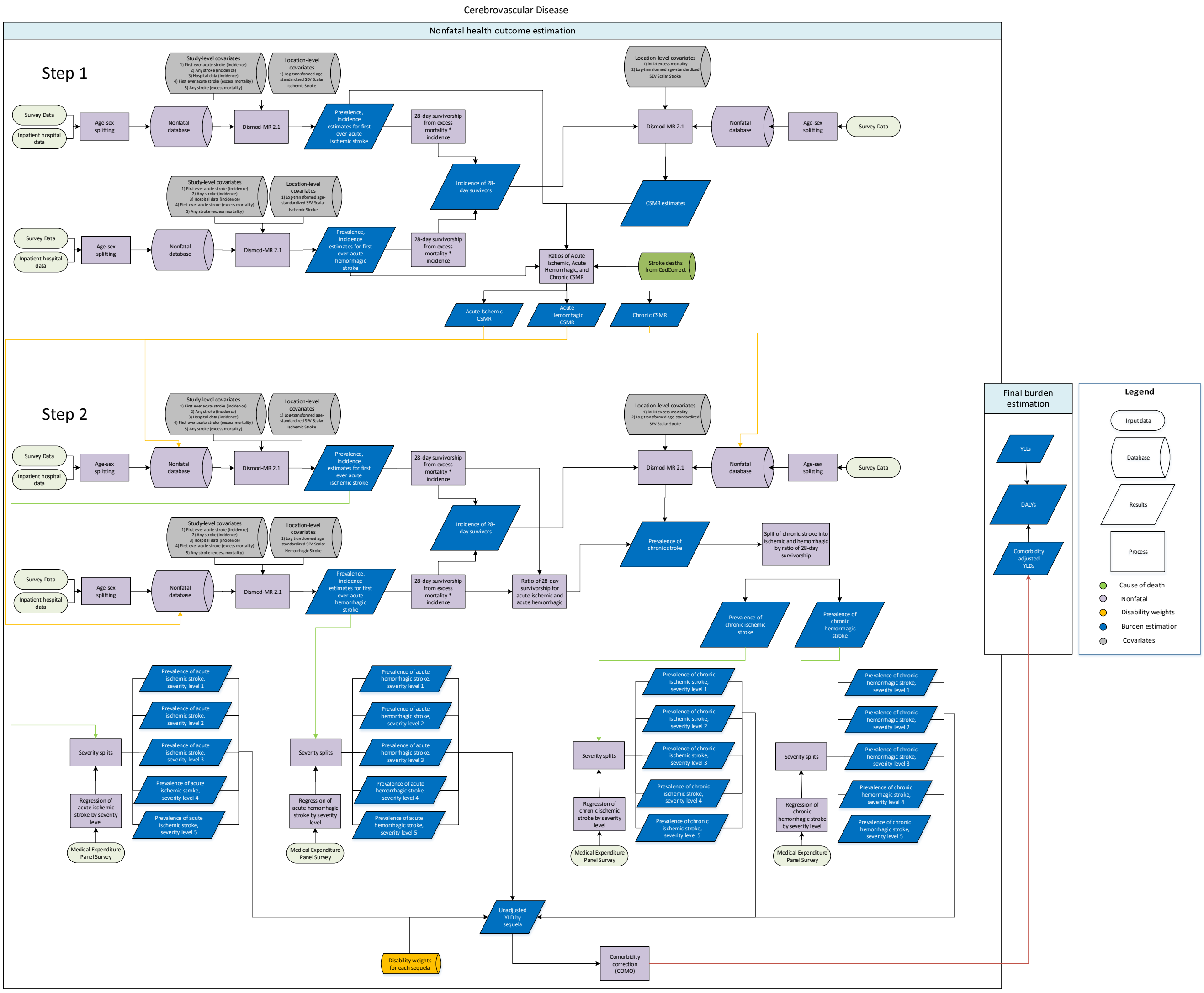

Input data and methodological summary

Case definition

Stroke was defined according to WHO criteria – rapidly developing clinical signs of focal (at times global) disturbance of cerebral function lasting more than 24 hours or leading to death with no apparent cause other than that of vascular origin. Data on transient ischemic attack (TIA) were not included.

Acute stroke: Stroke cases are considered acute from the data of incidence of a first-ever stroke through day 28 following the event.

Chronic stroke: **Stroke cases are considered chronic beginning 28 days following the occurrence of an event. Chronic stroke includes the sequelae of an acute stroke AND all recurrent stroke events. GBD 2015 adopts this broader definition of chronic stroke than prior iterations in order to model acute strokes using only first-ever incident events.**

Ischaemic stroke: Incident ischaemic stroke is defined as the occurrence of first-ever ischaemic stroke, based on clinical diagnosis by a physician using diagnostic imaging. Ischaemic strokes are considered to include all vascular events leading to limited blood flow to brain tissue, with resulting infarction, including atherosclerotic and thromboembolic strokes but excluding strokes in which the underlying cause is intracranial haemorrhage.

Haemorrhagic or other strokes: This cause includes all non-ischaemic strokes of a vascular cause including subarachnoid and stroke due to intracranial haemorrhage.

ICD codes used for inclusion of hospital data: G45-G46.8, I60-I61.9, I62.0-I62.03, I63-I63.9, I65-I66.9, I67.0-I67.3, I67.5-I67.7, I69.0-I69.198, I69.20-I69.398, I67.2, I69.3-I69.398, I67.0, I67.1, I67.7, I69.0-I69.198, I69.20-I69.298.

Input data

Model inputs

A systematic review was not performed for GBD 2015. Updates to systematic reviews are performed on an ongoing schedule across all GBD causes; an update for cerebrovascular disease will be performed in the next iteration.

A systematic review of the literature was performed in GBD 2013

- Search terms:
  - (stroke[Mesh]) AND (prevalence[Title/Abstract] OR incidence[Title/Abstract]) AND ("2010"[Date - Publication] : "3000"[Date - Publication]) AND (hasabstract[text] AND Humans[Mesh] AND middle age[MeSH]))
  - (hasabstract[text] AND Humans[Mesh] AND middle age[MeSH])) OR 21) AND ((hemorrhagic stroke/epidemiology[Mesh] OR hemorrhagic stroke/mortality[Mesh]) AND (prevalence[Title/Abstract] OR incidence[Title/Abstract]) AND ("2010"[Date - Publication] : "3000"[Date - Publication]) AND (hasabstract[text] AND Humans[Mesh] AND middle age[MeSH]))

The tables below indicates the number of literature studies included in GBD 2015, as well as the number of countries or subnational units and GBD world regions represented.

Cerebrovascular disease

	Prevalence	Incidence	Mortality risk
Studies	53	0	8

Countries/subnationals	50	0	4
GBD world regions	14	0	2

Ischaemic stroke

	Prevalence	Incidence	Mortality risk
Studies	0	71	45
Countries/subnationals	0	59	48
GBD world regions	0	12	17

Haemorrhagic or other stroke

	Prevalence	Incidence	Mortality risk
Studies	0	71	34
Countries/subnationals	0	59	43
GBD world regions	0	12	11

In addition to inpatient hospital data, we included unpublished stroke registry data for acute ischaemic and acute haemorrhagic strokes. We include survey data for chronic cerebrovascular disease. These surveys were identified based on expert opinion and review of major survey series focused on world health that included questions regarding self-reported history of stroke.

We included crosswalks to adjust data for first and recurrent strokes combined, using data for first strokes only as reference. We also included crosswalks for ischaemic and haemorrhagic strokes combined (all stroke), using as reference studies with subtype-specific information.

Severity split inputs

The standard GBD approach using MEPS data was used to determine severity splits for stroke. The table below illustrates the severity level, lay description, and disability weights for GBD 2015.

Severity level	Lay description	DW (95% CI)
Stroke, long-term consequences, mild	Has some difficulty in moving around and some weakness in one hand, but is able to walk without help.	0.019 (0.01–0.032)
Stroke, long-term consequences, moderate	Has some difficulty in moving around, and in using the hands for lifting and holding things, dressing, and grooming.	0.07 (0.046–0.099)
Stroke, long-term consequences, moderate plus cognition problems	Has some difficulty in moving around, in using the hands for lifting and holding things,	0.316 (0.206–0.437)

	dressing and grooming, and in speaking. The person is often forgetful and confused.	
Stroke, long-term consequences, severe	Is confined to bed or a wheelchair, has difficulty speaking and depends on others for feeding, toileting, and dressing.	0.552 (0.377–0.707)
Stroke, long-term consequences, severe plus cognition problems	Is confined to bed or a wheelchair, depends on others for feeding, toileting, and dressing, and has difficulty speaking, thinking clearly, and remembering things.	0.588 (0.411–0.744)

Modelling strategy

Three general approaches were employed for all of the components of the stroke modelling process, detailed in the table below.

- Data were crosswalked from nonstandard to standard case definitions using DisMod for all models. Coefficients for these crosswalks can be found in the tables for fixed effects located below.
- A GBD Standardised Exposure Variable for stroke and a covariate for country income were used as country-level covariates for all models. Coefficients for these covariates can be found in the tables for fixed effects located below.
- DisMod MR-2.1 was set with priors related to the coefficients of variation and heterogeneity for each model. Information for these parameters can be found in the tables of model parameters located below.

Step 1

- We generated estimates for first-ever acute ischaemic and first-ever acute haemorrhagic stroke using data collected on stroke incidence and excess mortality. We set value priors of 11 to 13 on remission for all ages to establish a one-month duration for these acute sequelae.
- We then calculated the incidence of surviving 28 days after an acute event for both ischaemic and haemorrhagic stroke using the modelled estimates of excess mortality and incidence.
- These survivor data were then uploaded into the chronic stroke, any type model as incidence.
- We then ran the chronic stroke model, using the survivor incidence data, prevalence data, and excess mortality data. We set a value prior of 0 on remission for all ages.
- Implausible or extreme outliers were dropped from these estimation results.
- From these three models, we generated the proportions of deaths for acute ischaemic, acute haemorrhagic, and chronic stroke, and split the post-CodCorrect stroke deaths generated from the GBD mortality estimates into these three parts. Thus, the proportion of deaths due to acute ischaemic, acute haemorrhagic, and chronic stroke are driven by all available data on incidence, prevalence, and excess mortality data for stroke. These CSMR estimates were then uploaded into the nonfatal database and used to estimates models for Step 2.

Step 2

- We re-ran the first-ever acute ischaemic and first-ever acute haemorrhagic models with CSMR as derived from CodCorrect and epidemiologic data as described above. Twenty-eight-day survivorship was recalculated from these models and uploaded into the chronic stroke, any type model with CSMR. As for acute models, this chronic model uses CSMR as derived from CodCorrect and epidemiologic data as described above.
- Implausible or extreme outliers were dropped from these estimation results.
- We then split the overall chronic stroke model into chronic haemorrhagic stroke and chronic ischaemic stroke based on the ratio of 28-day survivorship in the acute ischaemic and acute haemorrhagic models. The assumption built into this step is that the ratio of prevalent cases of chronic stroke matches that ratio of chronic stroke survivor cases at 29 days following an incident stroke.

Models were evaluated based on expert opinion, comparison with previous iterations, and model fit.

As described above, in GBD 2015 we are no longer directly estimating first and recurrent stroke combined. This decision was made in consultation with GBD stroke experts and reflects the fact that standard data reporting for stroke registries is for first-ever stroke. The majority of stroke incidence data available to GBD is for first-ever stroke.

The table below indicates the covariates used by cause in the estimation process, as well as the beta and exponentiated beta values.

Step 1:

Cause	Variable name	Measure	beta	Exponentiated beta
Chronic stroke; any type	Log-transformed age-standardised SEV scalar: Stroke	prevalence	.7833(.7512 to .8785)	2.189(2.12 to-2.407)
Chronic stroke; any type	LDI (I$ per capita)	excess mortality rate	-.1792(-.1819 to -.1769)	.836(.8337 to .8379)
First-ever acute haemorrhagic stroke	Hospital data	incidence	.5278(.5223 to .5298)	1.695(1.686 to 1.699)
First-ever acute haemorrhagic stroke	Any stroke	incidence	1.359(1.313 to 1.388)	3.892(3.717 to 4.007)
First-ever acute haemorrhagic stroke	First-ever acute stroke, ischaemic or haemorrhagic	incidence	.4925(.4163 to .5291)	1.636(1.516 to 1.697)
First-ever acute haemorrhagic stroke	Log-transformed age-standardised SEV scalar: haemorrhagic stroke	incidence	1.243(1.227 to 1.25)	3.468(3.411 to 3.49)
First-ever acute haemorrhagic stroke	Any stroke	excess mortality rate	-.4216(-.5741 to -.2617)	.656(.5632 to .7698)
First-ever acute haemorrhagic stroke	First-ever acute stroke, ischaemic or haemorrhagic	excess mortality rate	-.1409(-.3484 to .0613)	.8685(.7058 to 1.063)
First-ever acute ischaemic stroke	Hospital data	incidence	.002(4.3e-05 to .0067)	1.002(1 to 1.007)

First-ever acute ischaemic stroke	Any stroke	incidence	.4687(.4653 to .47)	1.598(1.592 to 1.6)
First-ever acute ischaemic stroke	First-ever acute stroke, ischaemic or haemorrhagic	incidence	.5142(.4772 to .5296)	1.672(1.612 to 1.698)
First-ever acute ischaemic stroke	Log-transformed age-standardised SEV scalar: ischaemic stroke	incidence	1.106(1.025 to 1.186)	3.021(2.787 to 3.274)

Step 2:

Cause	Variable name	Measure	beta	Exponentiated beta
Chronic stroke, any type with CSMR	Log-transformed age-standardised SEV scalar: Stroke	prevalence	.8185 (.7518 - .9986)	2.267 (2.121 - 2.714)
Chronic stroke, any type with CSMR	LDI (I$ per capita)	excess mortality rate	-.1879 (-.1917 - -.1845)	.8287 (.8256 - .8315)
First-ever acute haemorrhagic stroke with CSMR	Any stroke	incidence	1.401 (1.4 - 1.407)	4.06 (4.055 - 4.084)
First-ever acute haemorrhagic stroke with CSMR	First-ever acute stroke, ischaemic or haemorrhagic	incidence	9.8e-04 (2.2e-04 - .0049)	1.001 (1 - 1.005)
First-ever acute haemorrhagic stroke with CSMR	Log-transformed SEV scalar: haemorrhagic stroke	incidence	1.152 (1.031 - 1.243)	3.164 (2.804 - 3.466)
First-ever acute haemorrhagic stroke with CSMR	Any stroke	excess mortality rate	-.5999 (-.7527 - -.4538)	.5489 (.4711 - .6352)
First-ever acute haemorrhagic stroke with CSMR	First-ever acute stroke, ischaemic or haemorrhagic	excess mortality rate	-.2336 (-.512 - .0366)	.7917 (.5993 - 1.037)
First-ever acute ischaemic stroke with CSMR	Any stroke	incidence	.3452 (.3401 - .3575)	1.412 (1.405 - 1.43)
First-ever acute ischaemic stroke with CSMR	First-ever acute stroke, ischaemic or haemorrhagic	incidence	3.4e-04 (7.3e-05 - 9.8e-04)	1 (1 - 1.001)
First-ever acute ischaemic stroke with CSMR	Log-transformed age-standardised SEV scalar: Ischaemic stroke	incidence	1.248 (1.24 - 1.25)	3.483 (3.456 - 3.49)
First-ever acute ischaemic stroke with CSMR	Any stroke	excess mortality rate	-.6897 (-.8029 - -.5741)	.5017 (.448 - .5632)
First-ever acute ischaemic stroke with CSMR	First-ever acute stroke, ischaemic or haemorrhagic	excess mortality rate	-.869 (-.9992 - -.7466)	.4194 (.3682 - .474)

Fatal Alzheimer's Disease and Other Dementias estimation process

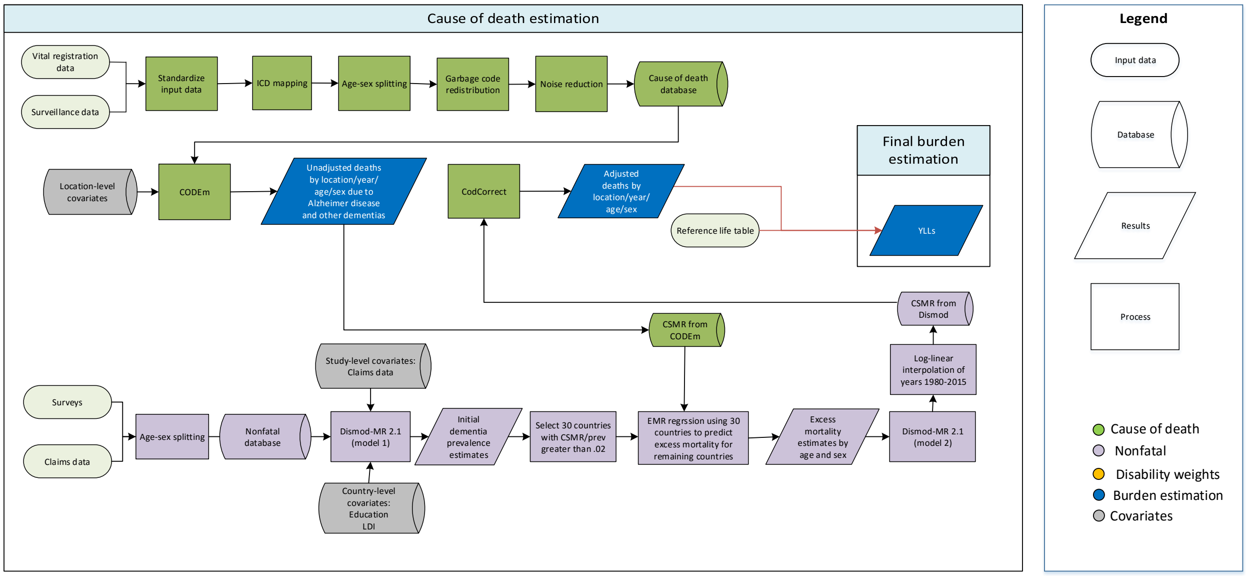

Input Data

In GBD 2015, data used to estimate deaths due to Alzheimer's disease and other dementias (dementias hereafter) included mortality data from vital registration systems and prevalence data from surveys and claims sources.

An updated systematic review was conducted from January 2013 to October 2015, and search terms[1] were set to capture studies for all dementia, including its sub-types. The search yielded 1,399 initial hits and 27 were marked for extraction. Inclusion criteria comprised studies that reported prevalence, incidence, remission rate, excess mortality rate, relative risk of mortality, standardised mortality ratio, or with-condition mortality rate. Studies with no clearly defined sample were excluded.

Modelling Strategy

Overview

Dementia mortality rates have increased more than five-fold since 1980 in high-quality vital registration systems such as in the US and Scandinavia. We have not seen an equivalent increase in prevalence and incidence data sources. If at all, there has been a modest decline in incidence and prevalence of dementia in studies in the UK and the US that used comparable survey methods. Also, the greater than 20-fold variation in mortality rates of dementia between countries is much greater the four-fold difference in prevalence and incidence between countries. As it is unlikely that case fatality from dementia has dramatically increased over the time period and that it would differ by a very large margin between

[1] ((dementia[Title/Abstract]) AND ((incidence[Title/Abstract]) or (prevalence[Title/Abstract])) AND ('2013'[Date - Publication] : '2015'[Date - Publication]))

countries, the hypothesis is that certifying and coding practices have changed over time and at a different pace between countries. To avoid spurious large trends over time in the fatal component of the burden of dementia, we decided for GBD 2013 to make dementia mortality rates consistent with the rates observed in 2015 relative to prevalence of countries that are most likely to certify or code dementia as an underlying cause of death. This approach was applied for GBD 2015, described further below.

Modelling steps

First, we ran a CODEm model for dementia and extracted the mortality rates by age, sex, and geography for 2015.

Second, we ran a DisMod-MR 2.1 model with all data on incidence, prevalence, and mortality risk (RR, SMR, or with-condition mortality rates) and a setting of zero remission and extracted 2015 prevalence by age, sex, and geography. The most substantial new source used for GBD 2015 was medical claims data, which provided data by age and sex for years 2000, 2010, and 2012 across all US states. To account for potential systematic differences between claims and survey data, we crosswalked for each year of claims data.

Third, we selected 30 countries with a cause-specific mortality rate to prevalence ratio greater than 0.02 (excluding small island nations and those without vital registration).

Fourth, we used a mixed effects regression with dummies on age group and sex to predict excess mortality (ie, the ratio of cause-specific mortality rate and prevalence) by age and sex, the results of which are found in the table below.

Table: Fixed effect coefficients of EMR regression. Outcome: ln(EMR)

Independent variables	Coef	Std. error	P value	95% Confidence Interval	
Female	-0.235	0.020	0.000	-0.274	-0.196
Age 60–64	0.707	0.034	0.000	0.640	0.774
Age 65–69	0.817	0.034	0.000	0.750	0.884
Age 70–74	1.120	0.034	0.000	1.053	1.188
Age 75– 80	1.471	0.034	0.000	1.404	1.539
Age 80+	2.198	0.034	0.000	2.131	2.266
Constant	-4.978	0.074	0.000	-5.122	-4.834
Random effect parameters					
Variance(constant)	0.143	0.038		0.085	0.240
Variance(residual)	0.034	0.003		0.029	0.040

We also fit a variation of the main EMR regression including the natural log of lagged distributed income (lnldi) as an additional covariate. The coefficient estimate and the corresponding confidence interval were then used to set a prior on the relationship between lnldi and EMR in DisMod-MR 2.1, where lnldi was a country-level covariate. This helped to capture location-specific variation in EMR for locations not included in the regression.

Fifth, these estimates were added to a second DisMod-MR 2.1 model as pertaining to the full 1990–2015 estimation period. For the 30 countries included in the regression, we retained their age- and sex-specific ratios and entered those also as pertaining to the full 1990–2015 estimation period.

Sixth, we took the predictions of cause-specific mortality by age, sex, geography, and year that DisMod-MR 2.1 calculated as being consistent with the data on incidence, prevalence, and the priors on excess mortality from step five. As mentioned, log lag distributed income per capita was used as a country-level covariate on EMR. The prior bounds of this latter selection were calculated using an iteration of the main EMR regression with log LDI as an additional covariate. We excluded data for standardised mortality ratio, with-condition mortality rate, relative risk as we wanted to estimate cause-specific mortality rates that were consistent with the level of excess mortality from the 30 chosen countries in 2015.

Seventh, because DisMod-MR 2.1 only produces estimates in five-year intervals from 1990 to 2015, we expanded the time series by log-linear interpolation. The trend from 1990–1995 was used to back-cast values for the 1980–1990 time period.

Non-fatal Alzheimer's Disease and Other Dementias estimation process

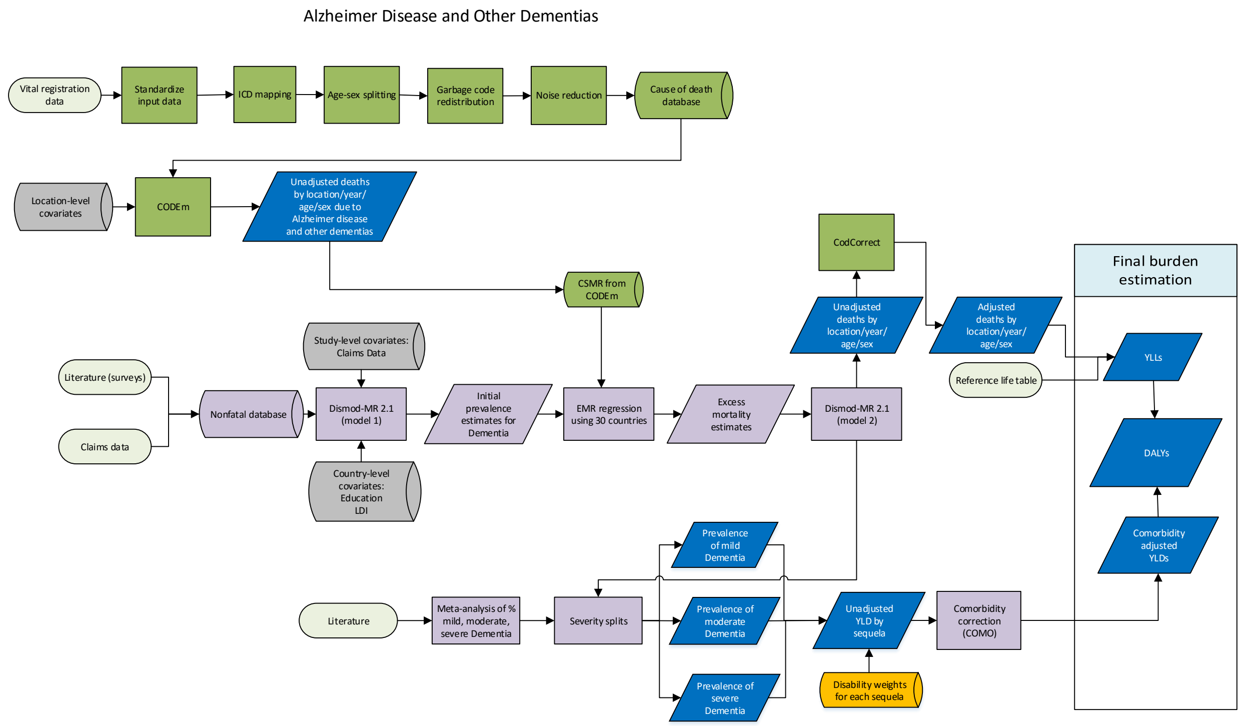

Input data and methodological summary

Case definition

Dementia is a progressive, degenerative, and chronic neurological disorder disease typified by memory impairment and other neurological dysfunctions. For the purposes of GBD 2015, we use the Diagnostic and Statistical Manual of Mental Disorders III or IV, or ICD case definitions as the reference. A wide array of diagnostic and screening instruments exists, including Clinical Dementia Rating scale (CDR), Mini Mental State Examination (MMSE), and the Geriatric Mental State (GMS). For severity rating purposes we use the CDR as the reference. The relevant ICD-10 codes for dementia are F00, F01, F02, F03, G30, and G31. The ICD-9 codes are 290, 291.2, 291.8, 294 and 331.

Unlike most causes in the Global Burden of Disease project, dementia mortality and morbidity estimates are modelled jointly. This is because of marked discrepancies between prevalence data and cause of death data. Specifically, prevalence data suggest little to no variation over time (eg, 1990–2015) whereas age-standardised mortality rates in vital registrations in high-income countries have increased multiple times over this same period. Additionally, prevalence variation between countries is much smaller than the variation in death rates assigned to dementia in vital registration. We attribute these discrepancies to changing coding practices rather than epidemiological change.

Because of this joint procedure, descriptions of the mortality estimation process are included where relevant.

Input data

Model inputs

To inform our estimates of burden due to dementia, we use mortality data from the vital registration systems, as well as prevalence data from surveys, and administrative data such as claims sources.

An update to earlier GBD systematic reviews was conducted from January 2013 to October 2015 with 1,399 initial hits and 27 marked for extraction. Inclusion criteria identified studies that reported prevalence, incidence, remission rate, excess mortality rate, relative risk of mortality, standardised mortality ratio, or with-condition mortality rate. Studies with no clearly defined sample, or on clinical samples were excluded.

A substantial new source used for GBD 2015 was medical claims data for the years 2000, 2010, and 2012 in all US states.

A table describing the density and distribution of the epidemiological data available for GBD 2015 is presented below:

	Prevalence	Incidence	Mortality risk	Severity
Studies	146	39	16	17
Countries/subnationals	96	13	19	15
GBD world regions	17	10	10	8

Severity splits

In GBD 2013 (and used in GBD 2015), we extracted data from studies reporting on mild, moderate, and severe dementia. As the data indicate an age pattern with greater proportions with more severe disease in the very old we restricted our analyses to studies reporting on severity <70, 70–79, and 80+ ages. Most of these studies reported severity based on the Clinical Dementia Rating scale (CDR): CDR=1 as mild, CDR=2 as moderate, and CDR=3 as severe dementia. Other studies report staging of dementia according to the Mini Mental State Examination (MMSE); DSM III criteria; the Functional capacity scale; the Cambridge Mental Disorders of the Elderly Examination (CAMDEX); the scale of Hughes and the Geriatric Mental State (GMS). We used a random effects meta-analysis to pool the data by severity level.

We multiplied estimations of prevalence (country-year-sex-age specific) by the fractions of mild, moderate, and severe dementia and estimated 95% uncertainty intervals at the 1,000 draw level.

Severity level	Lay description	DW (95% CI)	Severity distribution
Mild	The person has some trouble remembering recent events and finds it hard to concentrate and make decisions and plans.	0.069 (0.046–0.099)	<70: 79% (71–86%)<br>70-79: 71% (63–78%)<br>80+: 61% (53–68%)
Moderate	The person has memory problems and confusion, feels disoriented, at times hears voices that are not real, and needs help with some daily activities.	0.377 (0.252–0.508)	<70: 17% (11–23%)<br>70-79: 19% (14–24%)<br>80+: 26% (22–30%)
Severe	The person has complete memory loss, no longer recognises close family members, and requires help with all daily activities.	0.449 (0.304–0.595)	<70: 4% (2–7%)<br>70-79: 9% (5–13%)<br>80+: 12% (7–17%)

Modelling strategy

As mentioned above, the estimation of morbidity due to dementia occurs in conjunction with the mortality estimation.

First, we ran a CODEm model for dementia and extracted the cause-specific mortality rates by age, sex, and geography for 2015.

Second, we ran a DisMod-MR 2.1 model with all data on incidence, prevalence, and mortality risk (RR, SMR, or with-condition mortality rates) and a setting of zero remission and extracted 2015 prevalence by age, sex, and geography. To account for potential systematic differences between claims and survey data, we crosswalked for each year of claims data.

Third, we selected 30 countries with high-quality vital registration systems with a cause-specific mortality rate to prevalence ratio greater than 0.02 in 2015. These ratios are subsequently used in a regression to estimate general excess mortality – that is, to allow us to correct for the discrepancy between prevalence data and cause of death data described above.

Fourth, we used a mixed effects regression with dummies on age group and sex to predict excess mortality by age and sex, the results of which are found in the table below.

Table: Fixed effect coefficients of EMR regression. Outcome: ln(EMR)					
Independent variables	Coefficient	Standard error	P value	95% confidence interval	
Female	-0.235	0.020	0.000	-0.274	-0.196
Age 60–64	0.707	0.034	0.000	0.640	0.774
Age 65–69	0.817	0.034	0.000	0.750	0.884
Age 70–74	1.120	0.034	0.000	1.053	1.188
Age 75–80	1.471	0.034	0.000	1.404	1.539
Age 80+	2.198	0.034	0.000	2.131	2.266
Constant	-4.978	0.074	0.000	-5.122	-4.834

Random effect parameters					
Variance (constant)	0.143	0.038		0.085	0.240
Variance (residual)	0.034	0.003		0.029	0.040

We also fit a variation of the main EMR regression including the natural log of lagged distributed income (lnldi) as an additional covariate. The coefficient estimate and the corresponding confidence interval were then used to set a prior on the relationship between lnldi and EMR in DisMod-MR 2.1. This helped to capture location-specific variation in EMR for locations not included in the regression.

Fifth, these estimates were added to a second DisMod-MR 2.1 model as pertaining to the full 1990–2015 estimation period. For the 30 countries included in the regression, we retained their age- and sex-specific ratios and entered those also as pertaining to the full 1990–2015 estimation period. Thus, the model reflects the cause-specific mortality rate if all countries over time would have had the average propensity to code to dementia as an underlying cause of death similar to the selected 30 countries in 2015.

In this model, we assumed 0 remission as well as 0 excess mortality and incidence until age 40. Because of lack of consistency between prevalence and incidence data in locations where the underlying data, we excluded incidence data from the final model. In a few locations we found good consistency between prevalence and incidence and these were locations where incidence and prevalence were collected as part of the same study. In other locations (Beijing, Germany, Australia, Italy, North West England, Canada, various states in the US, Mexico, and Nigeria) we noted that DisMod-MR 2.1 was pushing the fit above the available prevalence data and below incidence – “averaging the difference”. In all cases the incidence and prevalence data were collected by different studies. We decided to drop the incidence estimates as

measuring incidence of dementia when symptoms are still mild is more prone to measurement bias than measuring prevalence when the diagnosis has become more obvious over time.

The table below provides additional information on the country covariates used in this model, as well as beta and exponentiated beta values.

Variable	Measure	Beta	Exponentiated Beta Value (CI)
LDI (I$ per capita)	excess mortality rate	-0.10	0.90 (0.90–0.90)
Mean years of education, age-standardised	prevalence	-0.04	0.96 (0.86–1.00)
US claims data 2000	prevalence	-0.69	0.50 (0.48–0 .53)
US claims data 2010	prevalence	-0.26	0.77 (0.74–0.82)
US claims data 2012	prevalence	-0.20	0.82 (0.79–0.86)

As described above, we used crosswalks to standardise the claims data relative to existing literature data and ln-LDI on EMR to capture location specific variation. Age-standardised education was used as a proxy for general brain health/use that may be protective of dementia – specifically Alzheimer's disease.

Fatal Parkinson's estimation process

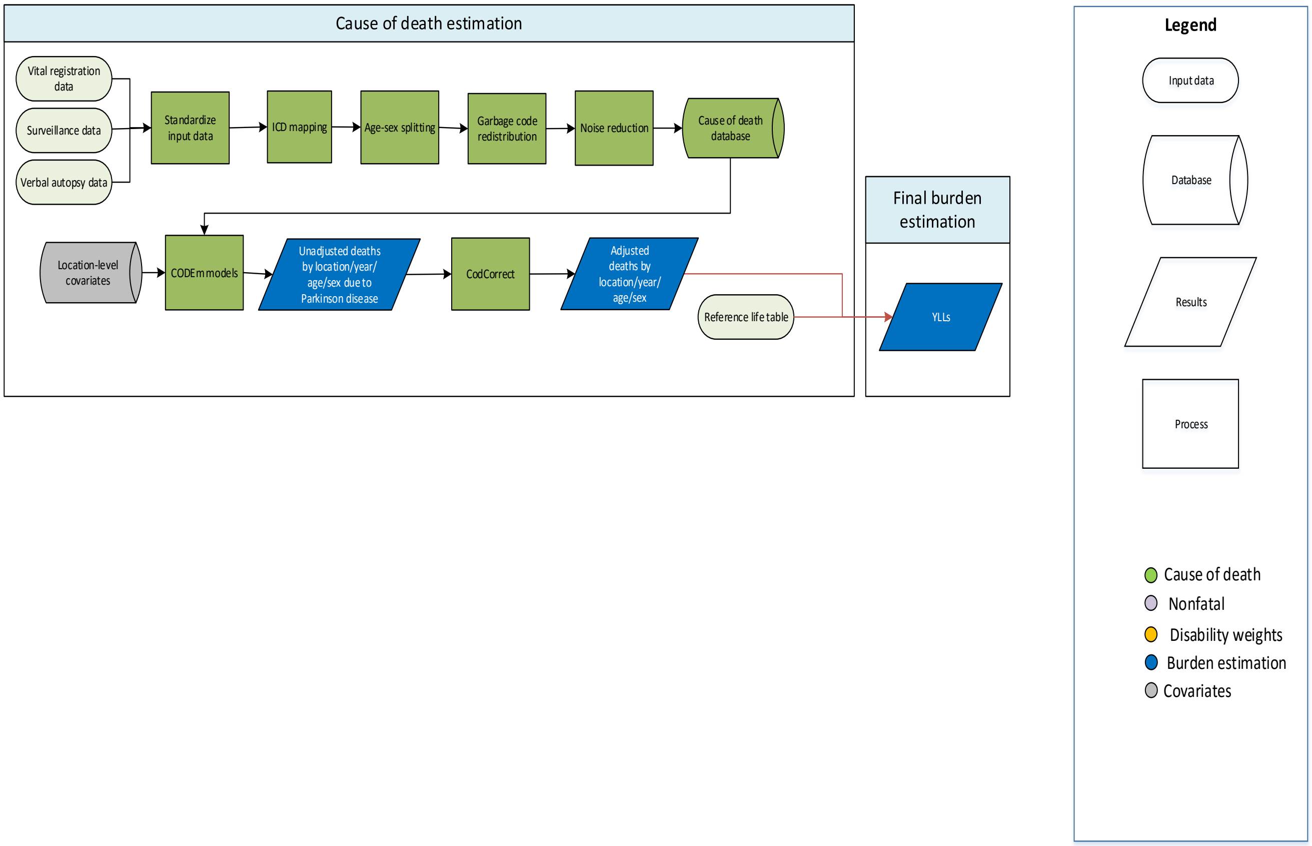

Input data

Data used to estimate Parkinson's disease included vital registration, surveillance, and verbal autopsy data from the cause of death (COD) database. Our outlier criteria excluded data points that (1) were implausibly high or low, (2) substantially conflicted with established age or temporal patterns, or (3) significantly conflicted with other data sources conducted from the same locations or locations with similar characteristics (ie, Socio-demographic Index).

Regarding data preparation, a change from GBD 2013 is that Parkinson's disease no longer receives garbage-coded deaths during the redistribution process. This change slightly reduces the number of uncorrected deaths but otherwise preserves the observed age and temporal patterns.

Modelling strategy

The standard CODEm modelling approach was applied to estimate deaths due to Parkinson's disease. Separate models were conducted for male and female mortality, and the age range for both models was 20–80+ years. There were no substantial changes from GBD 2013. The covariates used in GBD 2013 have been retained for this iteration, with the addition of the Socio-demographic Index (SDI) covariate.

Non-fatal Parkinson's Disease estimation process

Parkinson's Disease

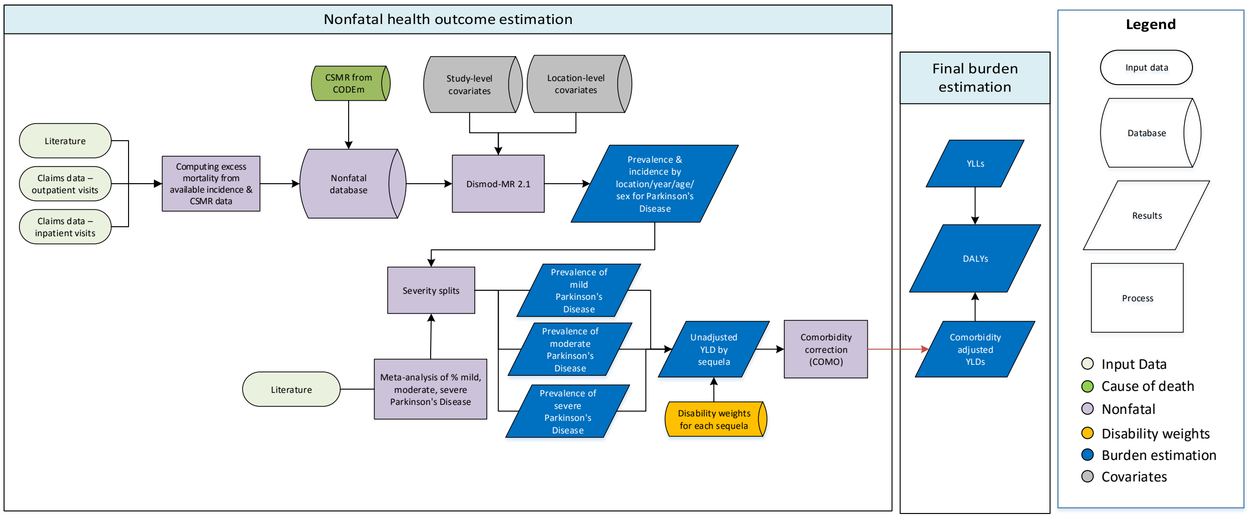

Case definition

Parkinson's disease

is a chronic, degenerative, and progressive neurological condition typified by the loss of motor mobility and control – most notably tremors. The corresponding ICD-10 codes are G20, G21, and G22. Our case definition for GBD is the presence of at least two of the four primary symptoms: (1) tremors/trembling, (2) bradykinesia, (3) stiffness of limbs and torso, and (4) posture instability.

Input data

Model inputs

For this iteration of GBD, we updated the systematic review for Parkinson's disease using the following search terms:

(((((Parkinson disease AND epidemiology) AND ("2011/01/01"[PDat] : "2015/12/31"[PDat]))) AND ((Parkinson disease AND epidemiology))))

This search term resulted in 1,433 initial hits with 17 sources marked for extraction. Studies with no clearly defined sample or that drew from specific clinic/patient organisations were excluded.

The data underpinning burden estimates due to Parkinson's are generally of two types. The first type, population-based studies, are part of the literature extraction and consist of cohort studies, surveys, and the like. The second are claims data from the United States for 2000, 2010, and 2012. Additional information on the source and preparation of these data is provided elsewhere.

The following table provides a description of the density and distribution of literature data informing the Parkinson's estimates:

	Prevalence	Incidence	Mortality risk
Studies	120	38	9
Subnational units	70	10	65
Countries	45	21	21
Regions	18	10	10

Beyond the exclusion of studies using non-representative populations, there are no substantial adjustment or outliering criteria for the Parkinson's model. Certain studies have been outliered on a case-by-case basis due to subsequent review and exclusion due to inappropriateness of the study design, and overly broad age and sex groups that conflict with existing gold-standard age-sex-specific data – where possible.

Severity splits

As in GBD 2013, we use Hoehn and Yahr stages to determine severity using the following cut points:

Severity	Stage
Mild	≤2.0
Moderate	2.5–4.0
Severe	>4

We continue to use the severity proportions generated for GBD 2013. In short, we conducted a meta-analysis of studies that reported prevalence of Parkinson's by Hoehn and Yahr stage. The analysis was stratified by high-income and low-middle-income status. The following figures show the results of this analysis:

Figure 1. Percentage of mild cases of Parkinson's disease in population-based studies

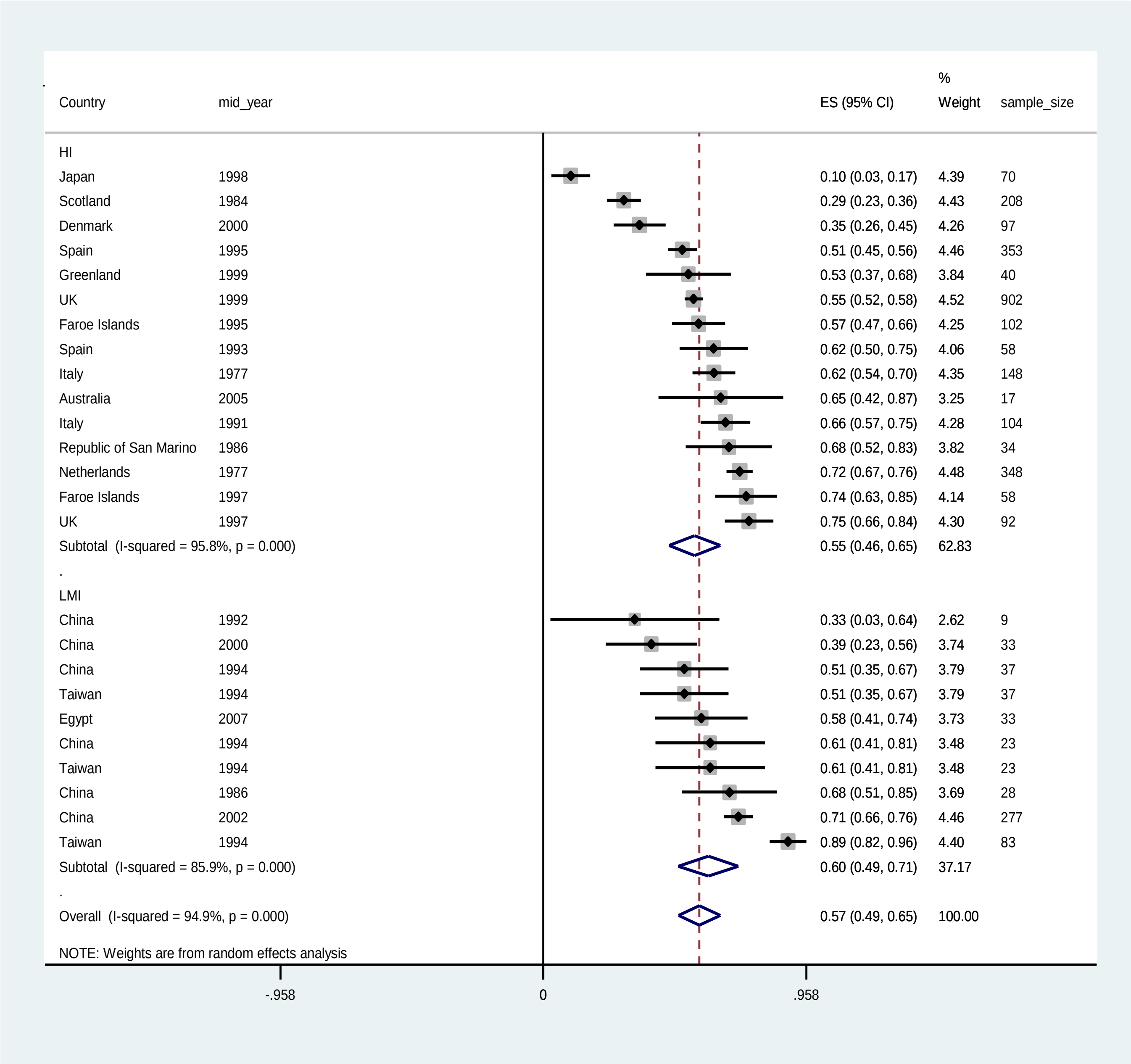

Figure 2. Percentage of moderate cases of Parkinson's disease in population-based studies

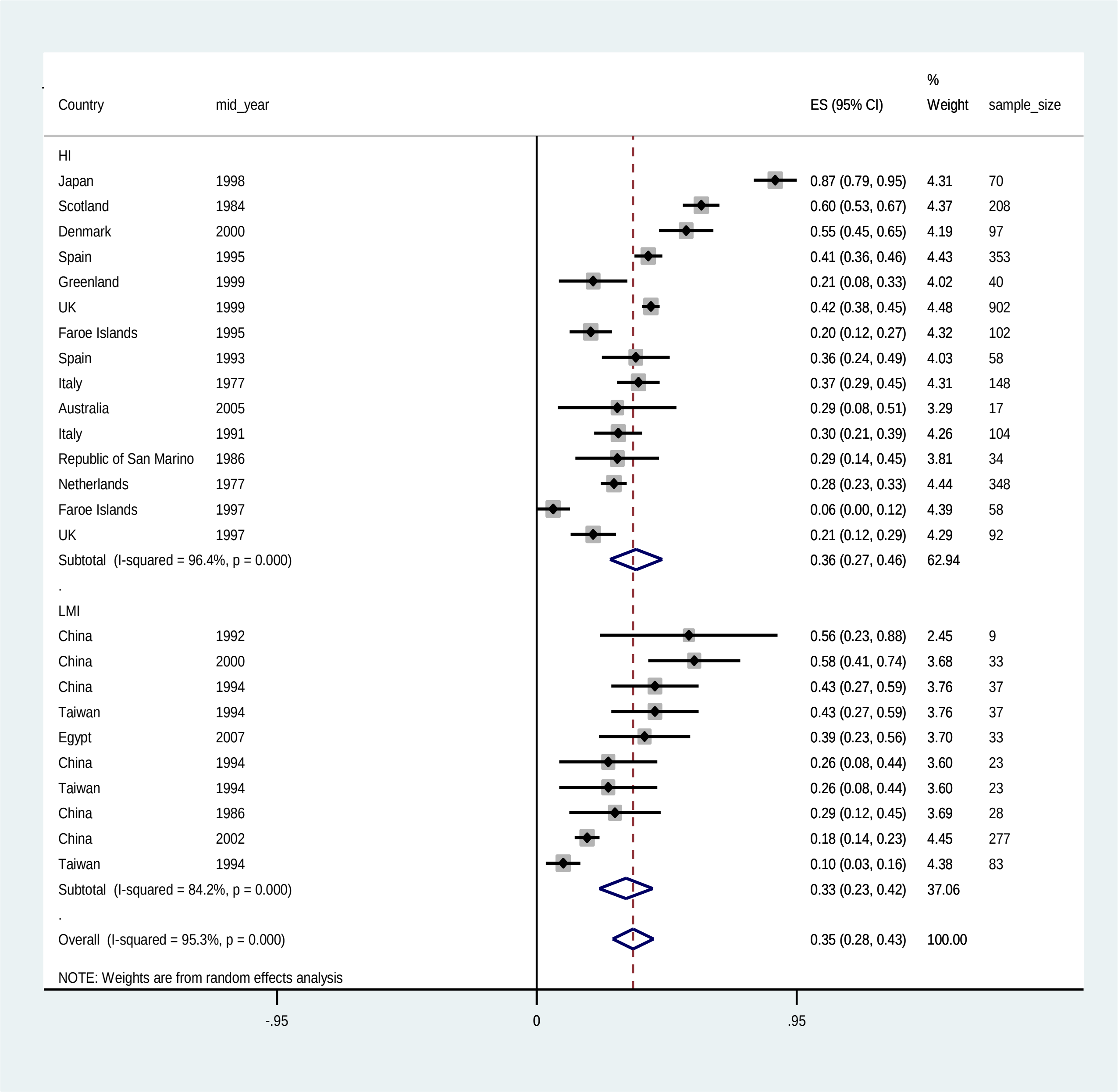

Figure 3. Percentage of severe cases of Parkinson's disease in population-based studies

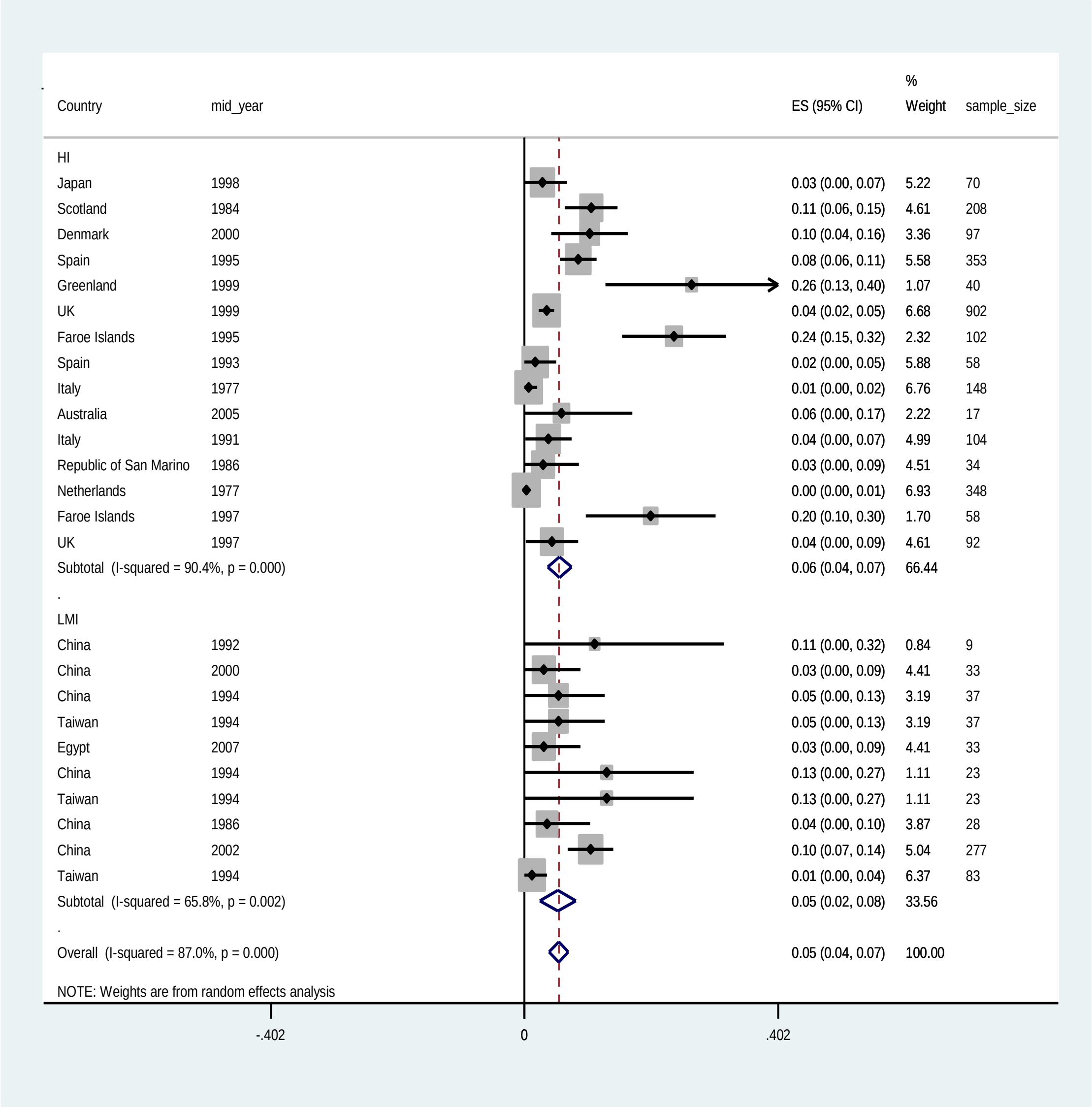

Severity estimates were generated by multiplying estimates of prevalence (country-year-sex-age-specific) by the fractions of mild, moderate, and severe PD and estimated 95% confidence intervals by taking 1,000 draws.

The following table provides the lay description and disability weights associated with Parkinson's disease.

Severity level	Lay description	DW (95% CI)
Mild	Has mild tremors and moves a little slowly, but is able to walk and do daily activities without assistance.	0.01<br>(0.005–0.019)

Moderate	Has moderate tremors and moves slowly, which causes some difficulty in walking and daily activities. The person has some trouble swallowing, talking, sleeping, and remembering things.	0.267 (0.181–0.372)
Severe	Has severe tremors and moves very slowly, which causes great difficulty in walking and daily activities. The person falls easily and has a lot of difficulty talking, swallowing, sleeping, and remembering things.	0.575 (0.396–0.73)

Modelling strategy

We use DisMod 2.1 as the main analytical tool for the Parkinson's disease estimation process. Prior settings are 0 remission among all ages, with no incidence or excess mortality for ages 0 to 20 years old. We ignore data on incidence, relative risk, standardised mortality ratio, and with-condition mortality as these were shown to be inconsistent with prevalence estimates. We also constrain the super-region random effects for prevalence and incidence to -0.5 and 0.5 to account for spurious inflation of regional differences. Similar to other causes, we use GBD estimates of cause-specific mortality rate (CSMR) and excess mortality rate (EMR) in this model.

We make two study-level crosswalks: Diagnostic Criteria and Ascertainment. Studies that ascertain cases on clinical record review rather than live diagnostic process are crosswalked to match the latter study design. Studies that do not use the gold-standard case definition of presence of at least two of the four main symptoms are crosswalked to meet this gold standard definition. The table below shows the effect of these crosswalks. Both result in an upward adjustment of non-standard data points.

Additionally, claims data for 2000 and 2010 are adjusted via study covariates to account for systematically low estimates relative to the 2012 claims data. Implicit in this adjustment is the assumption that variation between years of claims data is a function of data collection inconsistencies and noise.

For GBD 2015, we added a country-level crosswalk to assist DisMod in estimating global patterns. We use Socio-demographic Index as a proxy to capture possible social and cultural risk factors or modifiers of Parkinson's prevalence.

The following table provides an overview of the study-level and country covariates used in the Parkinson's model.

Covariate	Measure	Beta	Exponentiated
Socio-demographic Index	prevalence	.2302 (.0783 - .3789)	1.259 (1.081–1.461)

(Un)Filled diagnostic criteria	prevalence	-.2797<br>(-.4297 - -.1336)	.756<br>(.6507 - .8749)
All MarketScan, year 2010	prevalence	-.0237<br>(-.0514 - -.0024)	.9765<br>(.9499 - .9976)
All MarketScan, year 2000	prevalence	-.0929<br>(-.1321 - -.0561)	.9113<br>(.8762 - .9454)
Suboptimal Case Ascertainment	prevalence	-.3139<br>(-.4473 - -.1862)	.7306<br>(.6394 - .8301)

Although the foundation of the Parkinson's modelling strategy remains broadly similar, we do expect a few changes. First, the inclusion of SDI as a country covariate may slightly alter country and regional patterns. Second, unlike GBD 2013 we include Parkinson's cause-specific mortality estimates from earlier steps in GBD. We did this to ensure consistency between sets of estimates. However, there is evidence of the non-reliable pattern of death registries for this disease. It is likely that in the next iteration of GBD, we will move toward a single-step natural history model to estimate mortality and morbidity due to Parkinson's and therefore remove this limitation.

Fatal Epilepsy estimation process

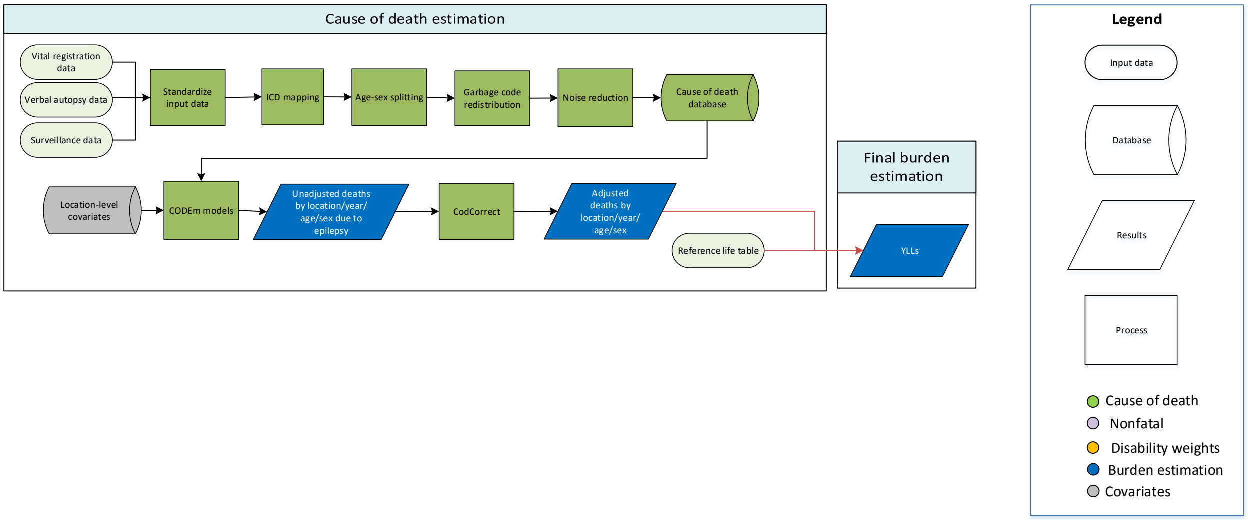

Input data

Data used to estimate epilepsy mortality included vital registration (VR), verbal autopsy, and China disease surveillance point data from the cause of death (COD) database. Our outlier criteria excluded data points that were implausibly high or low relative to global or regional patterns, substantially conflicted with established age or temporal patterns, or significantly conflicted with other data sources based from the same locations or locations with similar characteristics (ie, Socio-demographic Index).

Based on these criteria, we excluded ICD-9 BTL data for Sri Lanka, Fiji, and Kiribati as the estimates varied from year to year between zero and high values. We also outliered all VR data for Eastern Cape for ages 15–74 as this was a single province in South Africa for which the HIV correction (ie, removing excess deaths due to a cause during the HIV/AIDS epidemic compared to pre-epidemic years) was inadequate and caused this province to be an extremely high outlier globally.

Modelling strategy

The standard CODEm modelling approach was applied to estimate deaths due to epilepsy. We applied the same covariates used in GBD 2013 but added the Socio-demographic Index (SDI) variable created for this GBD cycle and the standardised exposure variable scalar (SEV-Scalar) for epilepsy. This covariate reflected the level of epilepsy burden attributed to alcohol (the only risk estimated for epilepsy). Otherwise, there were no changes from the GBD 2013 modelling strategy.

Non-fatal Epilepsy estimation process

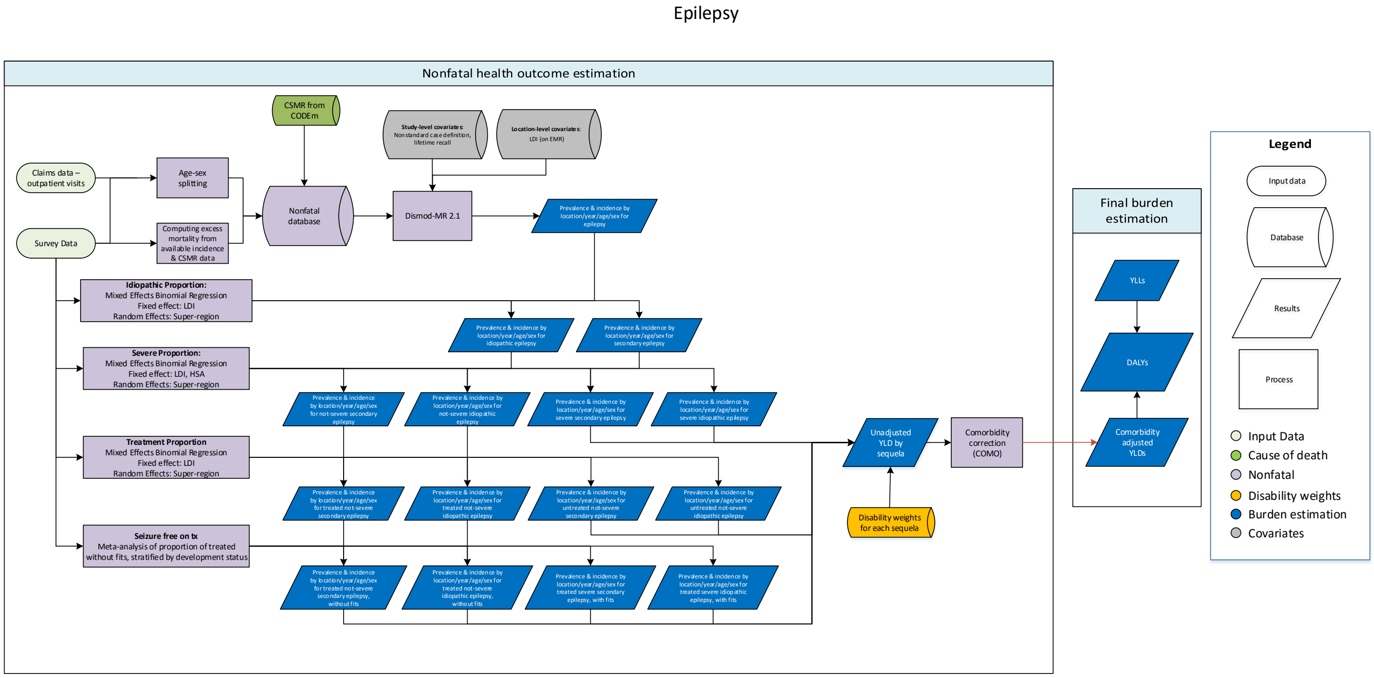

Case definition

For GBD 2013, we used the following definitions from the “Guidelines for Epidemiologic Studies on Epilepsy”: 1) Epilepsy: a condition characterised by recurrent (two or more) epileptic seizures, unprovoked by any immediate identified cause, and 2) “Active” epilepsy: a prevalent case of active epilepsy is defined as a person with epilepsy who has had at least one epileptic seizure in the previous five years, regardless of antiepileptic drug (AED) treatment.

For GBD 2015, we used the following ICD-10 codes for epilepsy: G40 (Neuro, epilepsy, total) and G41 (Neuro, epilepsy, status epilepticus). We defined severe epilepsy as having seizures one or more times per month.

Input data

Model inputs

For GBD 2013, we conducted a systematic review from 2009-2013 using the following search string:

("Epilepsy"[Mesh] OR "Epilepsy, Partial, Motor"[Mesh] OR "Epilepsy, Benign Neonatal"[Mesh] OR "Epilepsy, Reflex"[Mesh] OR "Myoclonic Epilepsy, Juvenile"[Mesh] OR "Epilepsy, Frontal Lobe"[Mesh] OR "Epilepsy, Complex Partial"[Mesh] OR "Epilepsy, Post-Traumatic"[Mesh] OR "Epilepsy, Temporal Lobe"[Mesh] OR "Epilepsy, Absence"[Mesh] OR "Epilepsy, Tonic-

Clonic"[Mesh] OR "Epilepsies, Myoclonic"[Mesh] OR "Epilepsies, Partial"[Mesh] OR (epilepsy[Title/Abstract]) AND (incidence[Title/Abstract] OR prevalence[Title/Abstract]) AND (“2009”[Date - Publication] : “2013”[Date - Publication]))

For GBD 2015, we conducted a systematic review searching from 1/1/2013 to 7/5/2015 using the following search string and extracted 19 relevant studies:

('Epilepsy'[Mesh] OR 'Epilepsy, Partial, Motor'[Mesh] OR 'Epilepsy, Benign Neonatal'[Mesh] OR 'Epilepsy, Reflex'[Mesh] OR 'Myoclonic Epilepsy, Juvenile'[Mesh] OR 'Epilepsy, Frontal Lobe'[Mesh] OR 'Epilepsy, Complex Partial'[Mesh] OR 'Epilepsy, Post-Traumatic'[Mesh] OR 'Epilepsy, Temporal Lobe'[Mesh] OR 'Epilepsy, Absence'[Mesh] OR 'Epilepsy, Tonic-Clonic'[Mesh] OR 'Epilepsies, Myoclonic'[Mesh] OR 'Epilepsies, Partial'[Mesh] OR (epilepsy[Title/Abstract]) AND (incidence[Title/Abstract] OR prevalence[Title/Abstract]) AND ('2013'[Date - Publication] : '2015'[Date - Publication])) Sort by: PublicationDate Filters: Humans

We included representative, population-based surveys that reported of prevalence, incidence, remission rate, excess mortality rate, relative risk of mortality, standardised mortality ratio, or with-condition mortality rate. We excluded studies with no clearly defined sample (eg, among clinic attenders or patient organization members with nonspecific or non-representative catchment area).

For GBD 2015, we also extracted Marketscan 2000, 2010, and 2012 data from inpatient and outpatient facilities. We did not use inpatient hospital data from other countries, as inpatient facility visits cannot be used to estimate prevalence. The tables below detail the model inputs used to estimate the epilepsy impairment.

	data_sources	subnational_coverage	country_coverage	region_coverage	super_region_coverage
prevalence	306	60	82	19	7
incidence	81	18	36	15	7
mortality	27	5	19	10	6

Severity splits & disability weights

The table below illustrates the severity levels, descriptions, and disability weights associated with epilepsy. These are calculated using regressions from literature (ie, frequency of seizures).

Severity level	Lay description	Disability weights (95% CI)
severe (seizures >= once per month)	This person has sudden seizures one or more times each month, with violent muscle contractions and stiffness, loss of	0.552<br>(0.375–0.71)

	consciousness, and loss of urine or bowel control. Between seizures the person has memory loss and difficulty concentrating.	
less severe (seizures < once per month)	This person has sudden seizures two to five times a year, with violent muscle contractions and stiffness, loss of consciousness, and loss of urine or bowel control.	0.263 (0.173–0.367)
Treated without fits	This person has a chronic disease that requires medication every day and causes some worry but minimal interference with daily activities.	0.049 (0.031–0.072)

Modelling strategy

We modelled the prevalence of epilepsy in two steps: first, we created an epilepsy impairment envelope. Second, we split the envelope into primary (or idiopathic) and secondary epilepsies. Each of these were subdivided into "severe" (on average 1 or more fits per month) and "non-severe." Non-severe cases were sub-divided into "treated" and "un-treated." Finally, "treated" cases were divided into "treated cases with fits" (between 1 and 11 fits on average in preceding year) and "treated cases without fits" (no fits reported in preceding year).

In the first step, we used the DisMod-MR tool for the epilepsy impairment envelope to model a consistent fit between incidence, prevalence, remission, and SMR data while using meta-regression to correct data points with non-reference study quality characteristics. We assumed a non-zero prevalence at birth to account for neonatal and congenital causes of epilepsy. We found no systematic bias for the covariate "non-standard case definition" indicating studies that did not define "active epilepsy" and added this covariate as a "z-cov" to the model which means a multiplier is applied to the standard error and thus is given less weight in the analysis than the "reference" data points. Unlike in GBD 2013, we included data of lifetime prevalence and therefore added a covariate on lifetime prevalence data points. We did not use sampling strategy as a z-cov because it did not have a significant effect. We included cause-specific mortality rate (CSMR) results from the epilepsy mortality model as input data to the DisMod model. Where age-specific prevalence data were available, we calculated excess mortality rate (EMR) from prevalence and CSMR. We included the log of the lag distributed income (LDI) as a covariate on EMR to account for lower mortality in developed countries. We included Bayesian priors on remission to account for the scarcity of remission data. We set bounds on remission from 0 to 0.25 from age 0–60 and 0 to

0.05 from age 61–100. The table below indicates the covariates used in the estimation process, as well as parameters, betas, and exponentiated betas.

Cause	Measure	Variable_Name	Beta	Exponentiated
Epilepsy impairment envelope	prevalence	Recall Lifetime	0.25 (0.20 - 0.30)	1.28 (1.22 - 1.35)
Epilepsy impairment envelope	prevalence	All MarketScan, year 2000	-0.76 (-0.94 - -0.69)	0.47 (0.39 - 0.50)
Epilepsy impairment envelope	prevalence	All MarketScan, year 2010	-0.26 (-0.42 - -0.19)	0.77 (0.66 - 0.83)
Epilepsy impairment envelope	prevalence	All MarketScan, year 2012	-0.18 (-0.35 - -0.11)	0.83 (0.71 - 0.90)
Epilepsy impairment envelope	incidence	Nonstandard case definition	0.14 (0.00 - 0.53)	1.15 (1.00 - 1.69)
Epilepsy impairment envelope	excess mortality rate	LDI (I$ per capita)	-0.30 (-0.30 - -0.30)	0.74 (0.74 - 0.74)

In the second step, we used a mixed-effects generalised linear model (binomial family) to predict the proportion of idiopathic epilepsy. We used a fixed effect on LDI, a lagged transformation of GDP per capita and super-region random effects in the final model. We also tested health system access as well as region and country effects in different models, but they did not improve the model. We used a similar model to predict the proportion of severe epilepsy and treatment gap based on the reported proportions extracted from the systematic review. We used fixed effects on health system access and LDI and super-region random effects in the final model for severe epilepsy. We also tested region and country effects in different models, but they did not improve the model. For estimating the treatment gap, we used fixed effects on LDI and health system access and super-region random effects in the final model. We tested region and country effects in different models, but they did not improve the model. We generated 1,000 draws of country-specific estimates for each year between 1980 and 2015 for each of the models.

Regression	covariate	beta	SE
Idiopathic	LDI	-0.58	0.03
Severe	LDI	0.49	0.07
Severe	HSA	-0.78	0.07
Treatment	LDI	-0.65	0.03
Treatment	HSA	-0.19	0.05

Seizure-free treated epilepsy

There were too few data points to use a mixed effects model. Instead, we used meta-analysis to generate two different pooled estimates for proportion of seizure free treated epilepsy in developing and developed countries.

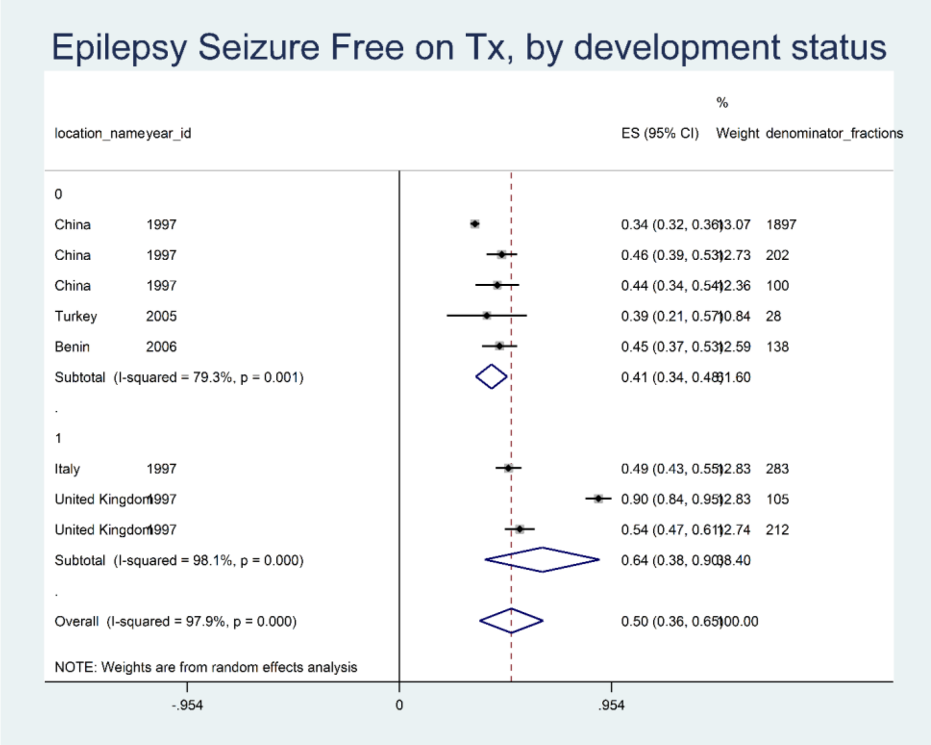

No additional changes were made to the modelling strategy for GBD 2015.

Fatal Multiple Sclerosis estimation process

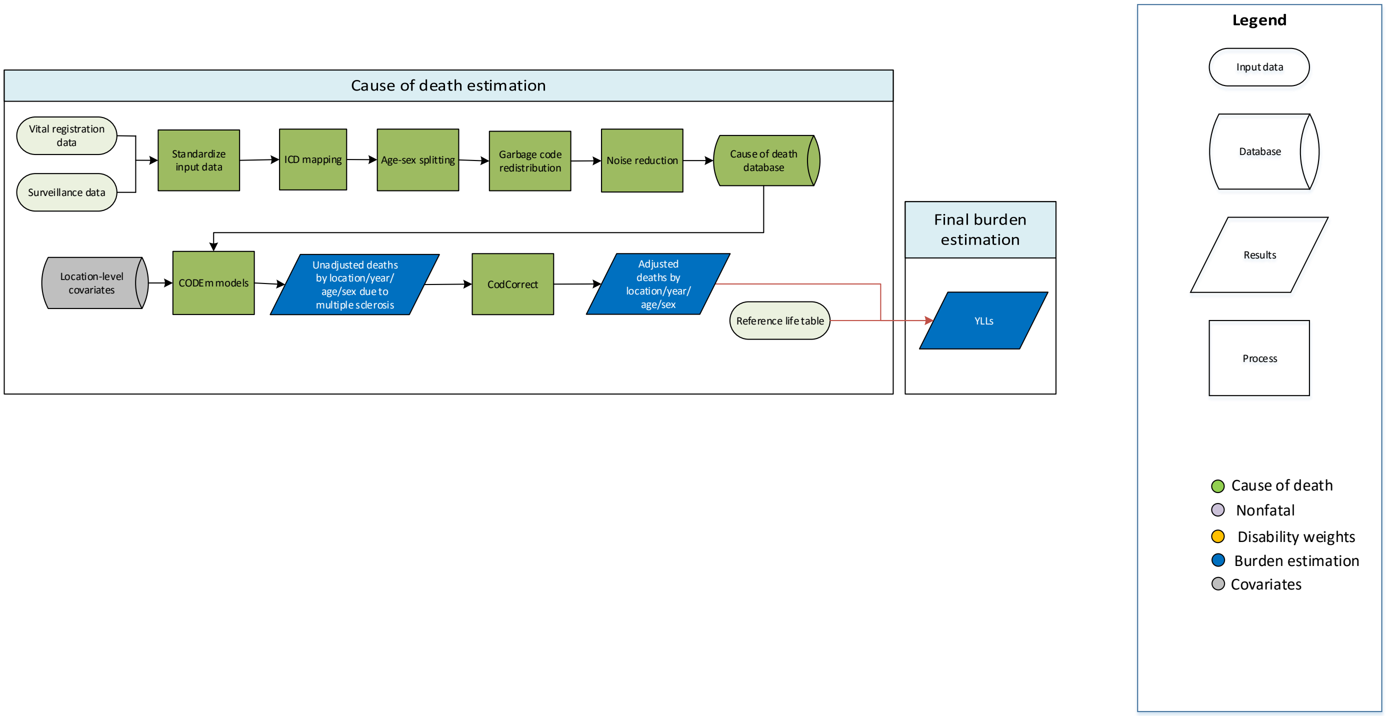

Input data

Data used to estimate multiple sclerosis included vital registration and surveillance data from the cause of death (COD) database. Our outlier criteria excluded data points that (1) were implausibly high or low, (2) substantially conflicted with established age or temporal patterns, or (3) significantly conflicted with other data sources conducted from the same locations or locations with similar characteristics (ie, Socio-demographic Index).

Modelling strategy

The standard CODEm modelling approach was used to estimate deaths due to multiple sclerosis. Separate models were conducted for male and female mortality, and the age range for both models was 20–80+ years. There were no substantial changes from GBD 2013. The covariates used in GBD 2013 have been retained for this iteration, with the addition of the Socio-demographic Index (SDI) covariate.

Non-fatal Multiple Sclerosis estimation process

Multiple Sclerosis

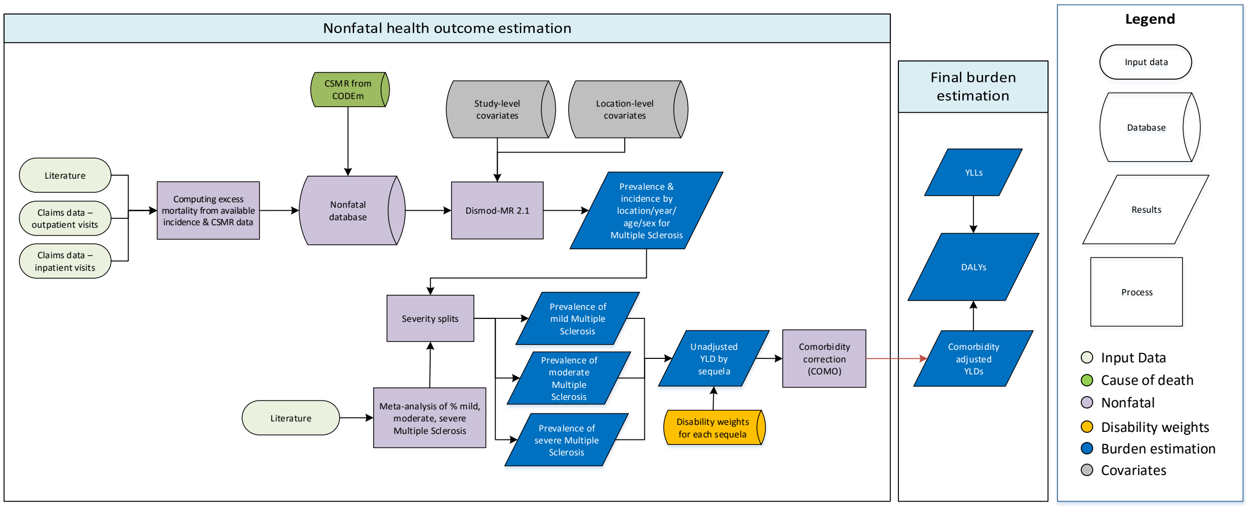

Input data and methodological summary

Case definition

Multiple Sclerosis is a chronic, degenerative, and progressive neurological condition typified by the damaging of the myelin sheaths. For GBD, the McDonald's criteria for diagnosis is considered the gold standard, but other definitions such as Poser Committee's criteria and self-report of a doctor's diagnosis are also included. The ICD-10 code for MS is G35.

Input data

A systematic review was conducted for MS for this iteration of GBD. A review was not done for GBD 2013. The search using (multiple sclerosis AND epidemiology AND ("2011/01/01"[PDat] : "2015/12/31"[PDat])) from 1/1/2011-7/15/15 yielded 1756 hits with 28 sources marked for extraction.

The data underpinning estimates of burden due to MS are generally of two types. The first, are representative, population-based surveys. This includes retrospective case/hospital report analysis, nationally representative health studies and the like. Studies with no clearly defined sample or that draw from specific clinic/patient organizations were excluded during the systematic review phase. The second type are claims data from the United States from 2000, 2010 and 2012. Additional information on the source and preparation of these data is provided elsewhere.

The following table provides a description of the density and distribution of literature data informing the MS estimates:

	Prevalence	Incidence	Mortality risk
Studies	147	59	15
Countries/subnationals	44/69	25/6	20/58
GBD world regions	12	8	8

Beyond the exclusion of studies using non-representative populations, there are no substantial adjustment or outliering criteria for the MS model. Certain studies have been outliered on a case by case basis due to: (1) subsequent review and exclusion due to inappropriate of the study design, and overly broad age and sex groups that conflict with existing gold standard age-sex specific data – where possible.

Severity splits

As in GBD 2013, we use Kurtzke's Expanded Disability Status Scale (EDSS) to determine severity splits for MS. They broke down to:

Mild: EDSS ≤ 3.5
Moderate: 3.5 < EDSS ≤ 6.5
Severe: 6.5 < EDSS ≤ 9.5

The table below illustrates the severity level, lay description and DW.

Severity level	Lay description	DW (95% CI)
Mild	Has mild loss of feeling in one hand, is a little unsteady while walking, has slight loss of vision in one eye, and often needs to urinate urgently.	0.183 (0.124–0.253)
Moderate	Needs help walking, has difficulty with writing and arm coordination, has loss of vision in one eye and cannot control urinating.	0.463 (0.313–0.613)
Severe	Has slurred speech and difficulty swallowing. The person has weak arms and hands, very limited and stiff leg movement, has loss of vision in both eyes and cannot control urinating.	0.719 (0.534–0.858)

To generate fractions for population level assignment, we re-use the meta-analysis conducted as part of GBD 2013. In short, we conducted a meta-analysis of all eligible studies that reported both prevalence and EDSS with separate results for high-income and low- and middle-income countries. The following figures provide the result of the meta-analysis.

Figure 1. Mild cases of MS

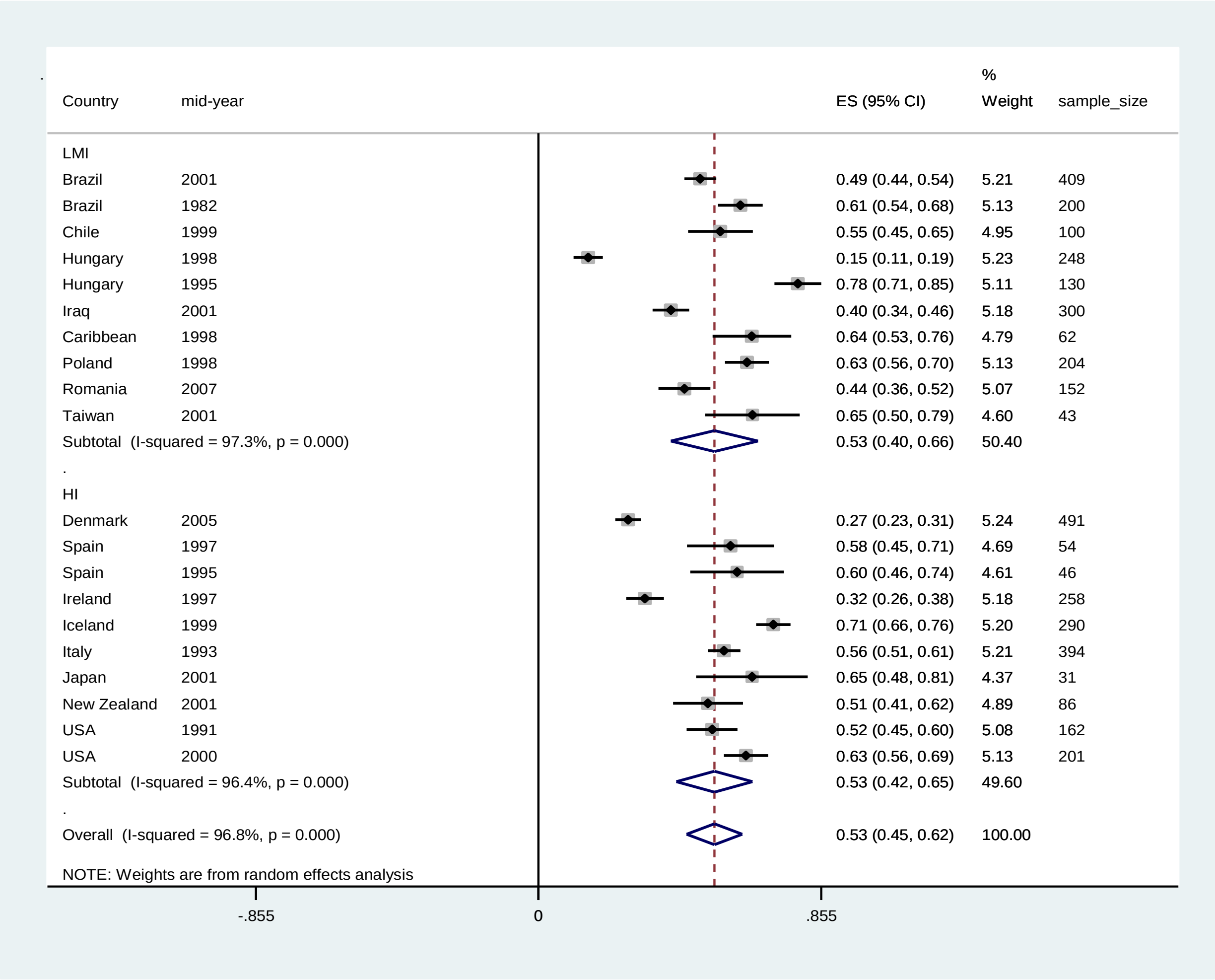

Figure 2. Moderate cases of MS

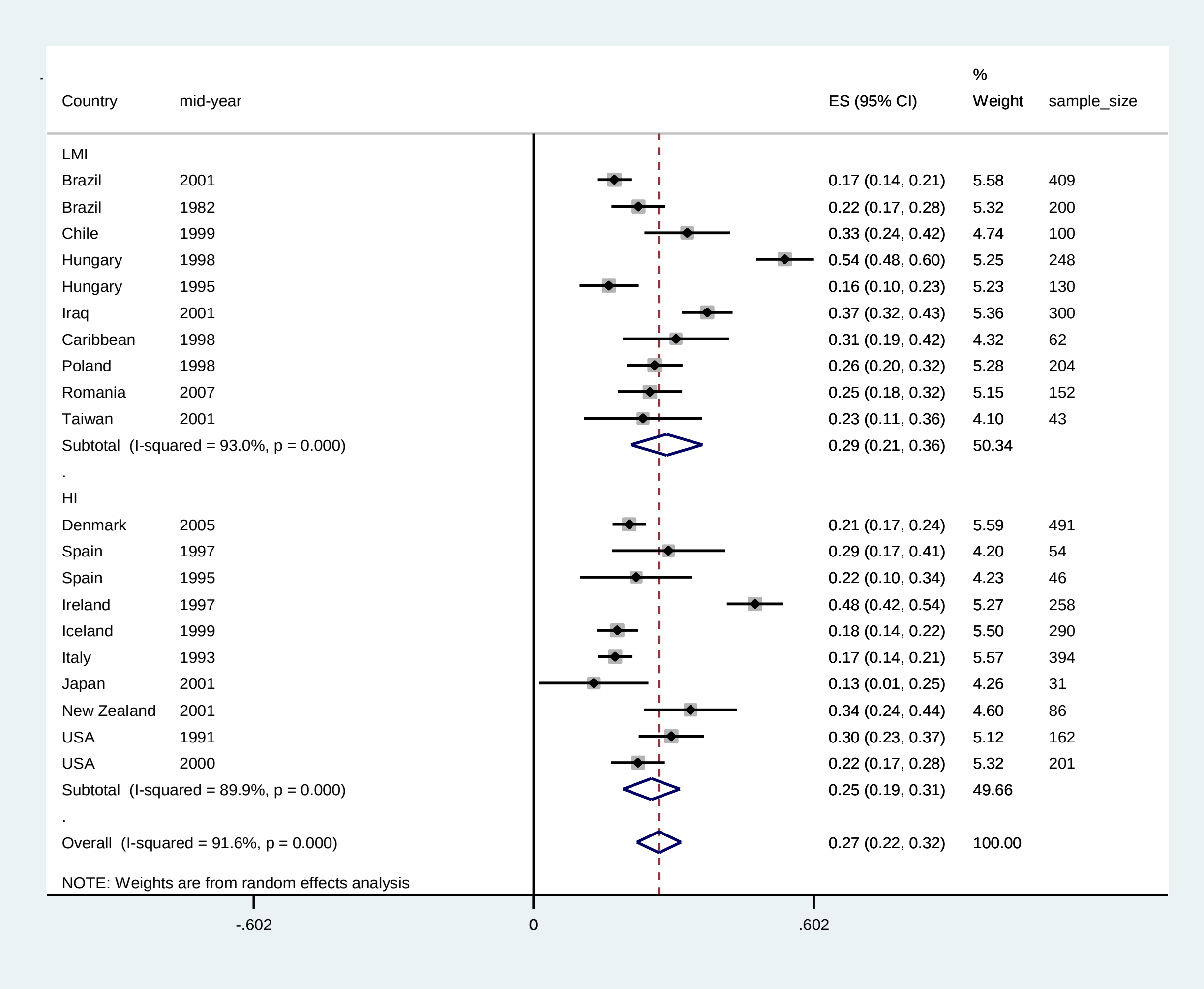

Figure 3. Severe cases of MS

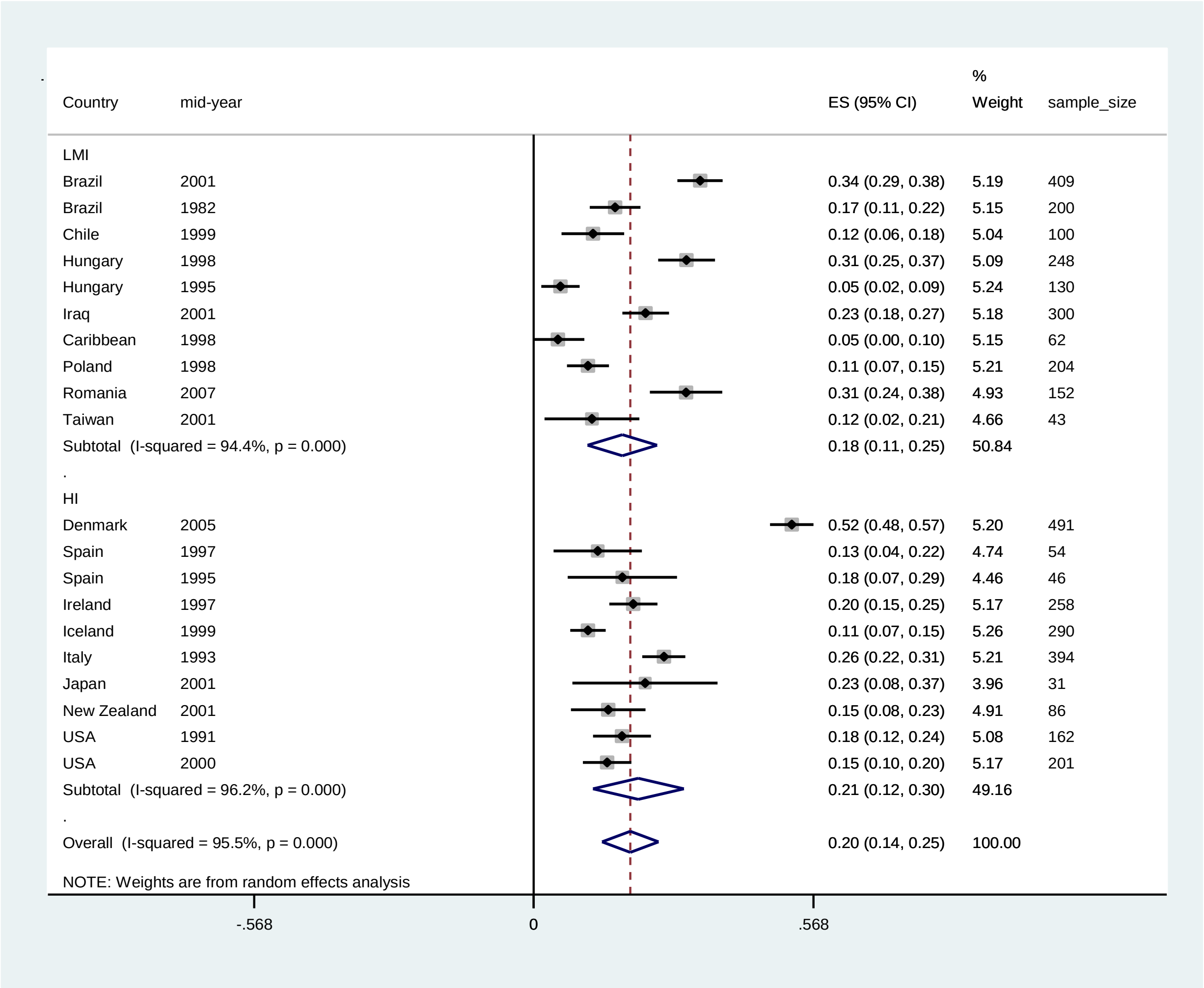

Modelling strategy

We use DisMod 2.1 as the main analytical tool for the MS estimation process. Prior settings include 0 remission for all ages, and no incidence or excess mortality for persons under 4 years old. We also constrain the super region random effects for prevalence and incidence to -.5 and .5 to account for spurious inflation of regional differences.

Unlike GBD 2013, we do not use study covariates to inflate the variance of data points with non-optimal case ascertainment and diagnostic criteria for model parsimony, as they did not meaningfully contribute the modelling process in this cycle.

Claims data for 2000 and 2010 are adjusted via study covariates to account for systematically low estimates relative to the 2012 claims data. Implicit in this adjustment is the assumption that variation between years of claims data is a function of data collection inconsistencies and noise.

Similar to other cases we use GBD estimates of cause-specific mortality rate (CSMR) and excess mortality rate (EMR) in this model.

To assist the estimation process, we use several country-level covariates. These effects plus those of the study covariates are presented below.

Covariate	Measure	Beta	Exponentiated	Parameter Type
Absolute value of average latitude	prevalence	.0377 (.0362 - .0393)	1.038 (1.037 - 1.04)	Country-level
Absolute value of average latitude	incidence	.0229 (.0165 - .0294)	1.023 (1.017 - 1.03)	Country-level
All MarketScan, year 2000	prevalence	-.3526 (-.3799 - -.325)	.7029 (.6839 - .7225)	x-cov
All MarketScan, year 2010	prevalence	-.0111 (-.0281 - -.0016)	.989 (.9723 - .9984)	x-cov
LDI (I$ per capita)	excess mortality rate	-.5187 (-.5358 - -.5013)	.5953 (.5852 - .6057)	Country-level
Socio-demographic Index	prevalence	-2 (-2 - -2)	.1353 (.1353 - .1353)	Country-level

As described in the literature, extreme latitude is associated with higher prevalence and incidence of MS. While the pathway of how this affects MS is not fully understood, our results suggest a sizable relationship. Our operationalization of latitude is created by a population-weighted average of latitude by country and taking the absolute value. The underlying population distribution rasters are part of the Gridded Population of the World dataset.

Although there are no known cures for MS, we expect disease management to differ globally – largely as a function of available resources. To capture this, we use the natural log of lagged distributed income per capita as a proxy to capture this relationship in the estimation of excess mortality.

To capture possible social and cultural risk factors or modifiers of MS prevalence, we include Socio-demographic Index as a covariate.

In general, we expect little change in the overall patterns of MS relative to GBD 2013. The main data types are consistent with previous iterations of GBD and new data are generally within the bounds of the existing dataset. We also used a substantially similar modelling strategy to GBD 2013.

Non-fatal Migraine estimation process

Migraine

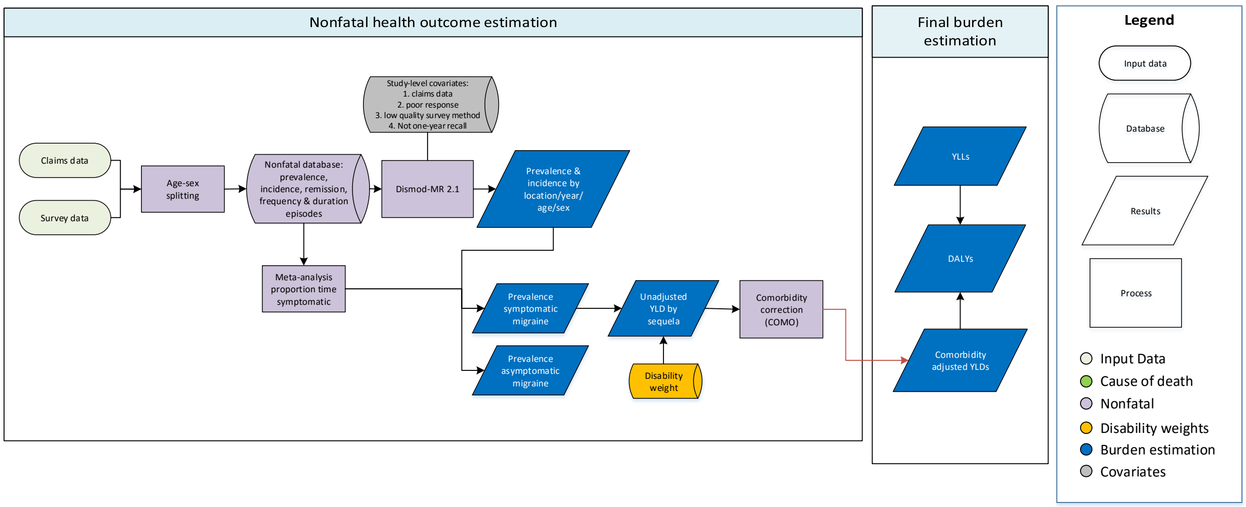

Input data and methodological summary

Case definition

Migraine is a class of disabling primary headache disorders, characterised by recurrent unilateral pulsatile headaches. The two major subtypes are common migraine (without aura) and classic migraine (with aura or neurological symptoms). In GBD we do not distinguish subtypes as most epidemiological studies report on overall migraine only. The ICD-10 code for migraine is G43 and ICD-9 code is 346.

Input data

Model inputs

A systematic review was conducted for GBD 2010 and updated for GBD 2013. For GBD 2015, three new representative surveys conducted by GBD collaborators in Norway; Karnataka, India; and Nepal were added. Inclusion criteria of the systematic reviews were:

- Representative, population-based surveys
- Reporting of prevalence of migraine headache

The table below illustrates the geographic distribution of data points.

	Prevalence	Incidence	Remission	Frequency and duration episodes
Studies	116	3	1	16
Countries/subnational locations	113	3	1	16
GBD world regions	16	2	1	9

In addition, data from US claims data for 2000, 2010, and 2012 by US state were included.

Severity splits

The basis of the GBD disability weight (DW) survey assessments are lay descriptions of sequelae highlighting major functional consequences and symptoms. The lay description and disability weight for migraine are shown below.

Severity level	Lay description	DW (95% CI)
Mild	This person has severe, throbbing head pain and nausea that cause great difficulty in daily activities and sometimes confine the person to bed. Moving around, light, and noise make it worse.	0.434 (0.285–0.603)

To determine the proportion of time spent over a year spent in an episode of migraine headache, 16 studies providing data on the frequency of episodes and the average duration of episodes were meta-analysed. As these studies reported frequency and duration of episodes by disparate categories, an assumption was made that the mean represented each category. For each study the estimated proportion of time symptomatic is 0.085 (0.058–0.112).

Modelling strategy

We used a list of binary covariates which are modified version of quality indicators of epidemiological studies on headache and shown in the table below.

**Study covariate**	**Notation**	
	**Less desirable (1)**	**Reference (zero)**
**Other than one-year recall period**	Point prevalence	One-year prevalence

Not representative	Selected population	General population or community-based sample from whole country OR general population or community-based sample from defined region within a country, or school-based (for children)
Low-quality sampling method	Not stated OR no (or failed) attempt to secure representativeness	Total defined population, or random sample corrected for population demographics OR random sample uncorrected for population demographics
Poor response	Not stated, or <70%	70–100%
Low-quality survey method and type of interviewer	Not stated OR self-administered (unsupervised) questionnaire OR telephone or face-to-face interview by untrained or unspecified interviewer(s)	Face-to-face interview with headache expert
Low-quality validation of diagnostic instrument	Instrument not specified or not validated OR validated, but sensitivity and/or specificity <70% OR validated only in screen-positive sub-sample, or in clinic or unspecified sample, but sensitivity and specificity ≥70%	Validated in target population or similar, and sensitivity and specificity ≥70%, or all diagnoses made in face-to-face or telephone interviews by headache expert
Low-quality diagnostic criteria	Not stated OR stated, other than ICHD OR ICHD (or reasonable modification), but uncertain or inappropriate analysis of "probable" diagnoses	ICHD (or reasonable modification) with clear exposition regarding "probable" diagnoses

We added separate covariates for the three years of claims data from MarketScan (2000, 2010, and 2012).

Prior settings in the DisMod model included setting incidence to 0 before age 5 based on expert advice. We also assume no excess mortality due to migraine.

All study covariates were initially evaluated as x-cov (which means that data points are adjusted to the reference value if a systematic bias is detected); those that did not have a significant coefficient, were entered as z-cov (which means that a multiplier is applied to the standard error of such data points to indicate they are less certain values because they did not meet the reference criteria for study quality). The table below shows the fixed effect values of the x-covs which are in log space (as DisMod uses an offset lognormal model) as well as the exponentiated values which for an x-cov can be interpreted as an odds ratio.

The covariates for low-quality sampling method, low-quality diagnostic criteria, and low-quality validation of diagnostic instrument and not representative studies had non-significant coefficients as an x-cov and were subsequently used as z-covs.

Study covariate	Parameter	beta	Exponentiated beta
Low-quality survey method and type of interviewer	Prevalence	0.18	1.20 (1.07–1.35)
Other than one-year recall period	Prevalence	-0.38	0.68 (0.61–0.76)
Poor response	Prevalence	-0.13	0.88 (0.81–0.95)
Claims data US 2000	Prevalence	-2.35	0.096 (0.071–0.12)
Claims data US 2010	Prevalence	-1.96	0.14 (0.11–0.17)
Claims data US 2012	Prevalence	-1.86	0.16 (0.12–0.19)

No other significant changes were made to the modelling strategy from GBD 2013.

Non-fatal Tension-type Headache estimation process

Tension-type headache

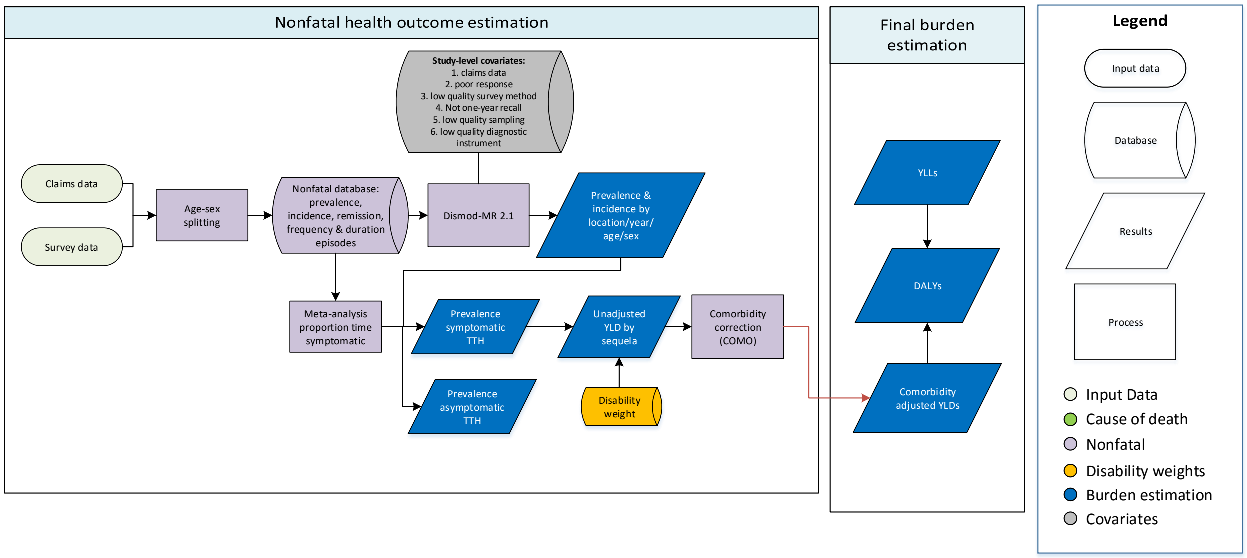

Case Definition

Tension-type headache is characterised by a dull, non-pulsatile, diffuse, band-like (or vice-like) pain of mild to moderate intensity in the head, scalp, or neck. The ICD-10 code for migraine is G44.2 and ICD-9 code is 339.1.

Input data

Model inputs

A systematic review was conducted for GBD 2010 and updated for GBD 2013. For GBD 2015 three new representative surveys conducted by GBD collaborators in Norway; Karnataka, India; and Nepal were added. Inclusion criteria of the systematic reviews were:

- Representative, population-based surveys
- Reporting of prevalence of TTH headache

	Prevalence	Incidence	Remission	Frequency and duration episodes
Studies	84	1	0	9

Countries/subnational locations	103	1	0	7
GBD world regions	15	1	0	6

In addition, data from US claims data for 2000, 2010 and 2012 by US state were included.

Severity splits

The basis of the GBD disability weight survey assessments are lay descriptions of sequelae highlighting major functional consequences and symptoms. The lay description and disability weight for migraine are shown below.

Severity level	Lay description	DW (95% CI)
Mild	This person has a moderate headache that also affects the neck, which causes difficulty in daily activities	0.036 (0.023–0.053)

To determine the proportion of time spent over a year spent in an episode of TTH headache, nine studies providing data on the frequency of episodes and the average duration of episodes were meta-analysed. As these studies reported frequency and duration of episodes by disparate categories, an assumption was made that the mean represented each category. The estimated proportion of time symptomatic is 0.058 (0.023–0.092).

Modelling strategy

We used a list of binary covariates which are modified version of quality indicators of epidemiological studies on headache (add ref: Steiner TJ, Stovner LJ et al (2013). Improving quality in population surveys of headache prevalence, burden and cost: key methodological considerations. J Headache Pain, 14: 87) and shown in the table below.

**Study covariate**	**Notation**	
	**Less desirable (1)**	**Reference (zero)**
**Other than one-year recall period**	Point prevalence	One-year prevalence
**Not representative**	selected population	general population or community-based sample from whole country OR general population or community-based sample from

		defined region within a country, or school-based (for children)
**Low-quality sampling method**	not stated OR no (or failed) attempt to secure representativeness	total defined population, or random sample corrected for population demographics OR random sample uncorrected for population demographics
**Poor response**	not stated, or <70%	70–100%
**Low-quality survey method and type of interviewer**	not stated OR self-administered (unsupervised) questionnaire OR telephone or face-to-face interview by untrained or unspecified interviewer(s)	face-to-face interview with headache expert
**Low-quality validation of diagnostic instrument**	instrument not specified or not validated OR validated, but sensitivity and/or specificity <70% OR validated only in screen-positive sub-sample, or in clinic or unspecified sample, but sensitivity and specificity ≥70%	validated in target population or similar, and sensitivity and specificity ≥70%, or all diagnoses made in face-to-face or telephone interviews by headache expert
**Low-quality diagnostic criteria**	not stated OR stated, other than ICHD OR ICHD (or reasonable modification), but uncertain or inappropriate analysis of "probable" diagnoses	ICHD (or reasonable modification) with clear exposition regarding "probable" diagnoses

We added separate covariates for the three years of claims data from MarketScan (2000, 2010, and 2012).

Prior settings in the DisMod model included setting incidence to 0 before age 5 based on expert advice. We also assume no excess mortality due to TTH. In the absence of any data on remission we set bounds between 0 and 0.5, ie, ensuring an average duration of at least two years.

All study covariates were initially evaluated as x-cov (which means that data points are adjusted to the reference value if a systematic bias is detected); those that did not have a significant coefficient were entered as z-cov (which means that a multiplier is applied to the standard error of such data points to indicate they are less certain values because they did not meet the reference criteria for study quality). The table below shows the fixed effect values of the x-covs which are in log space (as DisMod uses an offset lognormal model) as well as the exponentiated values which for an x-cov can be interpreted as an odds ratio.

The covariate for low-quality diagnostic criteria s had non-significant coefficients as an x-cov and was subsequently used as a z-cov.

Study covariate	Parameter	beta	Exponentiated beta
Low-quality survey method and type of interviewer	Prevalence	-0.43	0.65 (0.53–0.79)
Low-quality sampling method	Prevalence	1.00	2.72 (2.17–3.40)
Low-quality validation diagnostic instrument	Prevalence	0.63	1.88 (1.72–2.07)
Other than one-year recall period	Prevalence	-0.20	0.82 (0.70–0.98)
Poor response	Prevalence	-0.38	0.69 (0.60–0.79)
Claims data US 2000	Prevalence	-4.39	0.012 (0.012–0.013)
Claims data US 2010	Prevalence	-3.99	0.018 (0.018–0.019)
Claims data US 2012	Prevalence	-3.89	0.020 (0.020–0.021)

The very low coefficients in claims data mean that few cases of TTH are included in claims data. Data points were crosswalked up by a factor 50 or more. We decided to include the data with such large crosswalks as we had no other data for the states of the US and the crosswalks estimated by DisMod were within range of the data from three US studies in Massachusetts, Maryland, and Kentucky

Non-fatal Medication Overuse Headache estimation process

Medication overuse headache

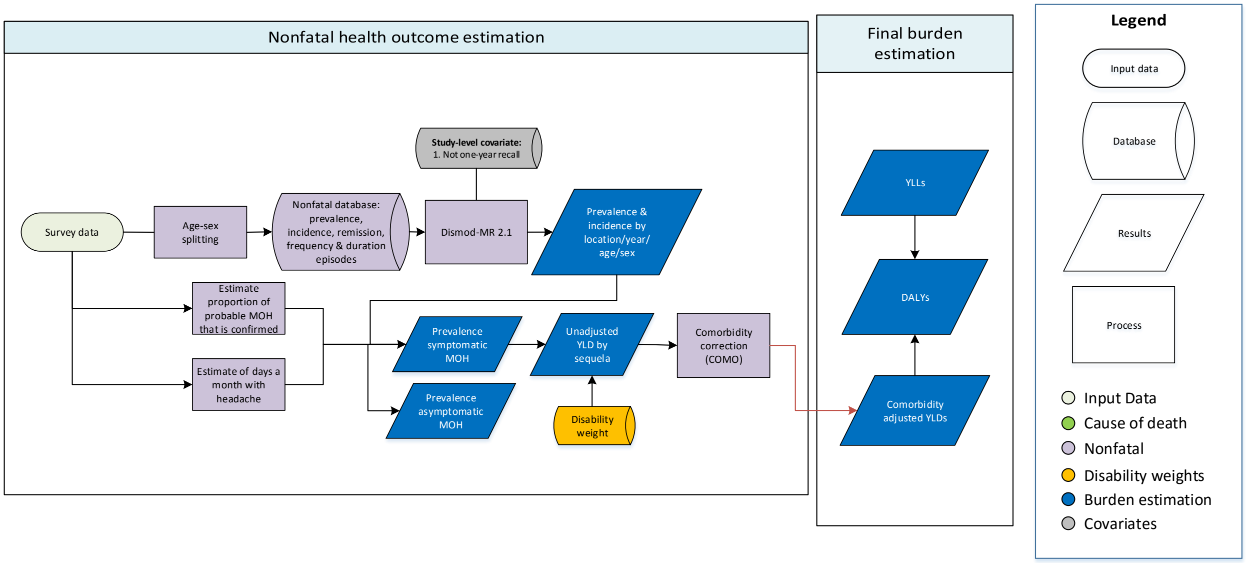

Case Definition

The diagnostic criteria (The International Classification of Headache Disorders, 3rd edition - beta version) for MOH are:

A. Headache present on ≥15 days/month fulfilling criteria C and D

B. Regular overuse (ie, >2 days per week) for ≥3 months of one or more drugs that can be taken for acute and/or symptomatic treatment of headache

C. Headache has developed or markedly worsened during medication overuse

D. Headache resolves or reverts to its previous pattern within 2 months after discontinuation of overused medication

It also explicitly states that if a person qualifies for chronic migraine or chronic TTH as well as MOH, both diagnoses should be given. However, our headache GBD collaborators, Steiner and Stovner, say that in survey practice, a screening question on chronic headache is used first, followed by questions to determine if medication overuse headache is probable (ie, fitting all criteria but criterion D).

Only one study was able to meet criterion D making a final diagnosis after a trial of detoxification. Of 25 cases with probably MOH, 15 were confirmed as MOH.

The headache survey in Russia reports an average frequency of 23.1 (SD 6.7; calculated SE 0.46) days with headache per month in people with chronic headache and report that over two-thirds of these are MOH.

Input data

Model inputs

A systematic review was conducted for GBD 2010 and updated for GBD 2013. For GBD 2015 three new representative surveys conducted by GBD collaborators in Norway; Karnataka, India; and Nepal were added. Inclusion criteria of the systematic reviews were:

- Representative, population-based surveys
- Reporting of prevalence of medication overuse headache

	Prevalence	Incidence	Remission
Studies	23	0	0
Countries/subnational locations	19	0	0
GBD world regions	7	0	0

Sequelae splits

The headache survey in Russia (Ayzenberg 2012) reports an average frequency of 23.1 (SD 6.7; calculated SE 0.46) days with headache per month in people with chronic headache and report that over two-thirds of these are MOH.

	Lay description	DW (95% CI)
Medication overuse headache	This person has daily headaches, felt as dull pain and often lasting all day, with poor sleep, nausea, and fatigue. The person takes medicine for the headaches, which provides little relief but is needed to avoid having worse symptoms.	0.217 (0.138–0.311)

Modelling Strategy

We used a list of binary covariates which are modified version of quality indicators of epidemiological studies on headache (ref: Steiner TJ, Stovner LJ et al (2013). Improving quality in population surveys of headache prevalence, burden and cost: key methodological considerations. J Headache Pain, 14: 87) and shown in the table below.

Study covariate	Notation	
	Less desirable (1)	Reference (zero)
Other than one-year recall period	Point prevalence	One-year prevalence
Not representative	selected population	general population or community-based sample from whole country OR general population or community-based sample from defined region within a country, or school-based (for children)
Low-quality sampling method	not stated OR no (or failed) attempt to secure representativeness	total defined population, or random sample corrected for population demographics OR random sample uncorrected for population demographics
Poor response	not stated, or <70%	70–100%
Low-quality survey method and type of interviewer	not stated OR self-administered (unsupervised) questionnaire OR telephone or face-to-face interview by untrained or unspecified interviewer(s)	face-to-face interview with headache expert
Low-quality validation of diagnostic instrument	instrument not specified or not validated OR validated, but sensitivity and/or specificity <70% OR validated only in screen-positive sub-sample, or in clinic or unspecified sample, but sensitivity and specificity ≥70%	validated in target population or similar, and sensitivity and specificity ≥70%, or all diagnoses made in face-to-face or telephone interviews by headache expert
Low-quality diagnostic criteria	not stated OR stated, other than ICHD OR ICHD (or reasonable modification), but uncertain or inappropriate analysis of "probable" diagnoses	ICHD (or reasonable modification) with clear exposition regarding "probable" diagnoses

Prior settings in the DisMod model included setting incidence to 0 before age 5 based on expert advice. We also assume no excess mortality due to TTH. In the absence of data on remission we set bounds between 0 and 1, thus ensuring that the average duration is at least one year.

All study covariates were initially evaluated as x-cov (which means that data points are adjusted to the reference value if a systematic bias is detected); those that did not have a significant coefficient, were entered as z-cov (which means that a multiplier is applied to the standard error of such data points to indicate they are less certain values because they did not meet the reference criteria for study quality). The table below shows the fixed effect values of the x-covs which are in log space (as DisMod uses an

offset lognormal model) as well as the exponentiated values which for a x-cov can be interpreted as an odds ratio.

The covariate for recall period was the only one with a significant coefficient x-cov. The others were subsequently used as a z-cov.

Study covariate	Parameter	beta	Exponentiated beta
Other than one-year recall period	Prevalence	-0.16	0.85 (0.62–1.23)

To the prevalence output from DisMod we first apply the finding from da Silva (2010) that 60% (40.8–79.2%) of “probable” cases were confirmed cases of MOH. Second, we estimate the proportion of time “symptomatic”, ie, with headache from the Ayzenberg (2012) estimate of 23.1 days a month with headache and multiply estimates by another 75.9% (72.9–78.8%).

Fatal Motor Neuron Disease estimation process

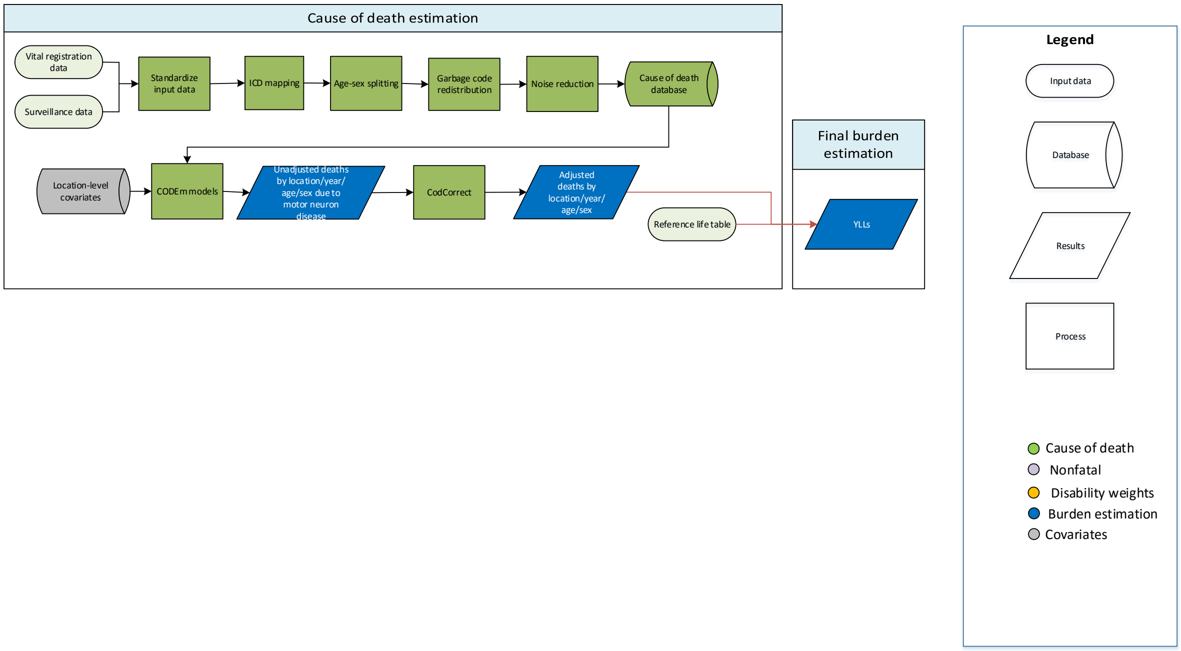

Input data

Data used to estimate motor neuron disease mortality included vital registration and surveillance data from the cause of death (COD) database. Our outlier criteria excluded data points that (1) were implausibly high or low, (2) substantially conflicted with established age or temporal patterns, or (3) significantly conflicted with other data sources conducted from the same locations or locations with similar characteristics (ie, Socio-demographic Index).

Modelling strategy

Previously, deaths due to motor neuron disease (MND) were classified and modelled under Other Neurological Disorders. For GBD 2015, we elevated MND deaths to their own cause. We used the standard CODEm modelling approach to generate estimates of deaths due to MND for ages 0 days–80+ years. The covariate structure of the MND model is very similar to other neurological causes and takes into account geographic factors (eg, latitude), infrastructure (water and sanitation, health systems access), and socioeconomic variables (eg, lag distributed income).

Non-fatal Motor Neuron estimation process

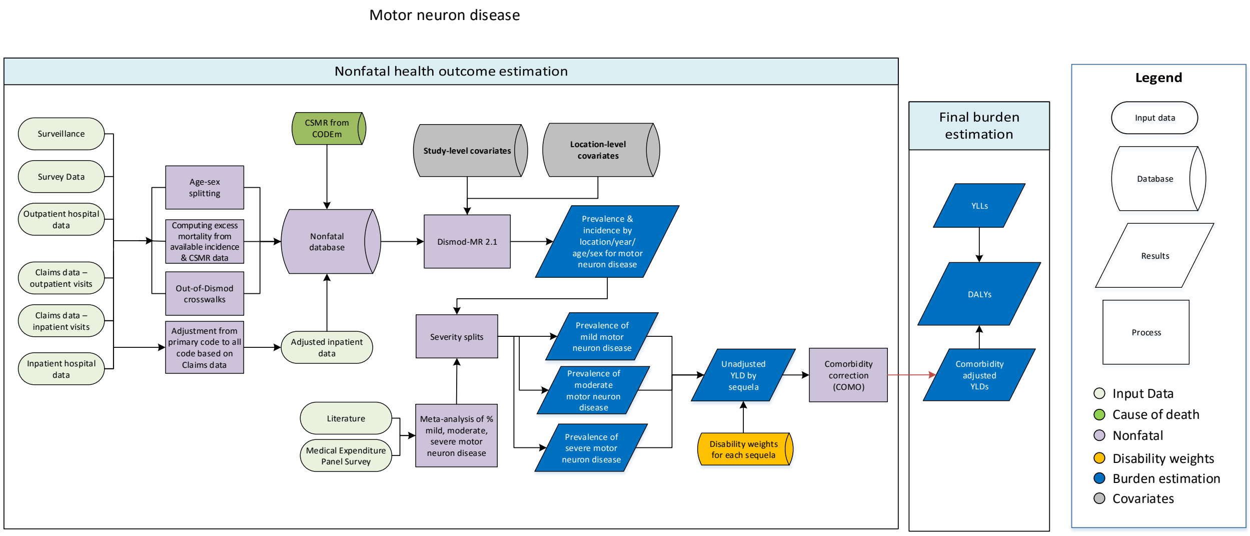

Case definition

Motor neuron diseases (MND) are a set of chronic, degenerative, and progressive neurological conditions typified by the destruction of motor neurons and the subsequent deterioration of voluntary muscle activity. The most common MND is Amyotrophic Lateral Sclerosis. This is a new cause for GBD 2015, with the corresponding ICD-10 code of G12. Our gold standard diagnostic criteria are the El Escorial Criteria, with other similar criteria (eg, the original set from World Federation of Neurology) if necessary.

Input data

As MND is a new cause in the Global Burden of Disease project, we conducted a full systematic review. The following search string guided our search, which resulted in 3,146 hits with 58 sources meeting extraction criteria: (1) the study is a representative population-based study, (2) reports on prevalence, incidence, remission, excess mortality, relative risk of mortality, standardised mortality ratio, or with-condition mortality rate. Studies with no clearly defined sample were excluded.

(('motor neuron disease'[MeSH Terms] OR ('motor'[All Fields] AND 'neuron'[All Fields] AND 'disease'[All Fields]) OR 'motor neuron disease'[All Fields] OR ('motor'[All Fields] AND 'neuron'[All Fields] AND 'diseases'[All Fields]) OR 'motor neuron diseases'[All Fields]) OR ('amyotrophic lateral sclerosis'[MeSH Terms] OR ('amyotrophic'[All Fields] AND 'lateral'[All Fields] AND 'sclerosis'[All Fields]) OR 'amyotrophic lateral sclerosis'[All Fields]) OR ALS[All Fields] OR ('motor neuron disease'[MeSH Terms] OR ('motor'[All Fields] AND 'neuron'[All Fields] AND 'disease'[All Fields]) OR 'motor neuron disease'[All Fields] OR ('primary'[All Fields] AND 'lateral'[All Fields] AND 'sclerosis'[All Fields]) OR 'primary lateral sclerosis'[All Fields]) OR ('Politics Life Sci'[Journal] OR 'pls'[All Fields]) OR ('muscular atrophy, spinal'[MeSH Terms] OR ('muscular'[All Fields] AND 'atrophy'[All Fields] AND 'spinal'[All Fields]) OR 'spinal muscular atrophy'[All Fields] OR ('progressive'[All Fields] AND 'muscular'[All Fields] AND 'atrophy'[All Fields]) OR 'progressive muscular atrophy'[All Fields]) OR PBP[All Fields] OR ('pseudobulbar palsy'[MeSH Terms] OR

('pseudobulbar'[All Fields] AND 'palsy'[All Fields]) OR 'pseudobulbar palsy'[All Fields])) AND (('epidemiology'[Subheading] OR 'epidemiology'[All Fields] OR 'epidemiology'[MeSH Terms]) OR population-based[All Fields])

The following table provides an overview of the density and distribution of the data extracted from the literature.

	Prevalence	Incidence	Mortality risk
Studies	11	47	5
Subnational units	53	9	53
Countries	6	17	7
Regions	5	7	5

Beyond the literature data, we also make use of claims data from the United States for 2000, 2010, and 2012. Descriptions of the source and preparation of this data are provided elsewhere.

Except for excluding studies using non-representative populations, there are no substantial adjustments or outliering criteria for the MND model. Certain studies have been outliered on a case-by-case basis due to (1) subsequent review and exclusion due to inappropriateness of the study design, and (2) overly broad age and sex groups that conflict with existing gold standard age-sex-specific data – where possible.

Severity splits

To calculate severity and disability due to MND we analysed a dataset from Pooled Resource Open-access ALS Clinical Trials (PRO-ACT). This dataset contains the largest ALS clinical trials dataset, with a total of 8,635 ALS patient records from multiple completed clinical trials. Among these, we conducted the final analysis with n=4,838 (56%) of the patients with complete ALS Function Rating Score (ALSFRS) with average follow-up time of 184 days (min: -22, max: 648), in which 2,999 (62%) received experimental (medication) treatments and 1,301 (27%) received placebo (in these trials, the medications tested were found to be no better than placebo with respect to their effects on ALS progressions).

The ALSFRS is an instrument for evaluating the functional status of patients with Amyotrophic Lateral Sclerosis. It can be used to monitor functional changes in a patient over time. It measures (1) speech, (2) salivation, (3) swallowing, (4) handwriting, (5) cutting food and handling utensils (with or without gastrostomy), (6) dressing and hygiene, (7) turning in bed and adjusting bed clothes, (8) walking, (9) climbing stairs, and (10) breathing. Each task is rated on a 5-point scale from 0 = can't do, to 4 = normal ability. Individual item scores are summed to produce a reported total score of between 0 and 40 (worst to best). ALSFRS has been revised to ALSFRS-R, which includes 12 questions (ALSFRS Q10 changes to (10)

Dyspnoea, (11) Orthopnoea, and (12) Respiratory insufficiency), with individual item scores summed to a score between 0 and 48.

In order to eliminate any bias from the treatment effects on the ALSFRS, only the first observation at the time of trial is selected. If the first observation is missing at the time of trial (or prior), the next non-missing observation is selected to be included in the final analysis.

We subsequently mapped ALSFRS scores into GBD severities, and sequelae into different combinations of speech problems, chronic obstructive pulmonary disease, and motor impairment using the following logic:

Motor impairment

The ALSFRS assess motor function of the legs through questions on walking (Q8) and stair climbing (Q9).

Combined score	Severity level
8	None
5–7	Mild
2–4	Moderate
0–1	Severe

The ALSFRS also assesses motor impairment through questions on handwriting (Q4), cutting food and handling utensils (Q5), and dressing and hygiene (Q6).

Combined score	Severity level
12	None
9–11	Mild
3–8	Moderate
0–2	Severe

After determining case severity on these two separate metrics, we aggregate by taking the most severe ranking (eg, severe + mild = a severe case).

Respiratory problems:

Question 10 of the ALSFRS describes breathing difficulty as a function of MND.

ALSFRS score	Description	Severity level
4	Normal	None
3	Shortness of breath with minimal exertion	Mild
2	Shortness of breath at rest	Moderate
0–1	Intermittent ventilator assistance required/ventilator-dependent	Severe

Speech problems

Speech impairment due to MND is derived from ALSFRS question 1, which describes speech impediments. A score of 4 on this question denotes no impairment, while all other values suggest some impairment.

Creating sequelae

After determining the severity status of each case for the three symptom umbrellas, we subsequently estimated the relative proportion of each combination of symptom class and their respective severities. Those without any symptoms (eg, no severity) were categorised as having worry about the diagnosis for disability estimation. The following table displays the various sequelae and their associated proportions.

Sequelae	Proportion (Mean)	Proportion (Lower)	Proportion (Upper)
Mild motor impairment, mild respiratory problems, and speech problems due to motor neuron disease	0.01779	0.01658	0.01909
Mild motor impairment, moderate respiratory problems, and speech problems due to motor neuron disease	0.00270	0.00225	0.00324
Mild motor impairment, severe respiratory problems, and speech problems due to motor neuron disease	0.00082	0.00059	0.00113
Mild motor impairment, and speech problems due to motor neuron disease	0.02052	0.01922	0.02190
Moderate motor impairment, mild respiratory problems, and speech problems due to motor neuron disease	0.03377	0.03210	0.03552

Moderate motor impairment, moderate respiratory problems, and speech problems due to motor neuron disease	0.00715	0.00640	0.00799
Moderate motor impairment, severe respiratory problems, and speech problems due to motor neuron disease	0.00286	0.00240	0.00342
Moderate motor impairment, and speech problems due to motor neuron disease	0.03041	0.02883	0.03208
Severe motor impairment, mild respiratory problems, and speech problems due to motor neuron disease	0.05242	0.05035	0.05457
Severe motor impairment, moderate respiratory problems, and speech problems due to motor neuron disease	0.02247	0.02111	0.02392
Severe motor impairment, severe respiratory problems, and speech problems due to motor neuron disease	0.01365	0.01259	0.01479
Severe motor impairment and speech problems due to motor neuron disease	0.04765	0.04567	0.04970
Mild respiratory problems and speech problems due to motor neuron disease	0.01157	0.01060	0.01263
Moderate respiratory problems and speech problems due to motor neuron disease	0.00142	0.00111	0.00182
Severe respiratory problems and speech problems due to motor neuron disease	0.00023	0.00013	0.00043
Speech problems due to motor neuron disease	0.02457	0.02315	0.02608
Mild motor impairment and mild respiratory problems due to motor neuron disease	0.02245	0.02109	0.02389
Mild motor impairment and moderate respiratory problems due to motor neuron disease	0.00275	0.00230	0.00329
Mild motor impairment and severe respiratory problems due to motor neuron disease	0.00068	0.00047	0.00097
Mild motor impairment due to motor neuron disease	0.10388	0.10103	0.10681
Moderate motor impairment and mild respiratory problems due to motor neuron disease	0.06744	0.06511	0.06985

Moderate motor impairment and moderate respiratory problems due to motor neuron disease	0.01302	0.01199	0.01413
Moderate motor impairment and severe respiratory problems due to motor neuron disease	0.00412	0.00356	0.00477
Moderate motor impairment due to motor neuron disease	0.20136	0.19760	0.20518
Severe motor impairment and mild respiratory problems due to motor neuron disease	0.06902	0.06666	0.07146
Severe motor impairment and moderate respiratory problems due to motor neuron disease	0.02000	0.01872	0.02137
Severe motor impairment and severe respiratory problems due to motor neuron disease	0.01062	0.00969	0.01163
Severe motor impairment due to motor neuron disease	0.15037	0.14702	0.15378
Mild respiratory problems due to motor neuron disease	0.00643	0.00571	0.00723
Moderate respiratory problems due to motor neuron disease	0.00044	0.00028	0.00069
Severe respiratory problems due to motor neuron disease	0.00005	0.00001	0.00017
Asymptomatic, but worry about diagnosis due to motor neuron disease	0.03738	0.03562	0.03921

To determine disability due to these sequelae, we use the standard multiplicative aggregation formula as described in the main text. The following table provides description and disability weight assigned to the sequelae as appropriate.

Symptom group	Severity level	Lay description	DW (95%)
Respiratory problems	Asymptomatic		
Respiratory problems	Mild	Has cough and shortness of breath after heavy physical activity, but is able to walk long distances and climb stairs.	0.019 (0.011–0.033)
Respiratory problems	Moderate	Has cough, wheezing, and shortness of breath, even after light physical activity. The person feels tired and can walk only	0.225 (0.153–0.31)

		short distances or climb only a few stairs.	
Respiratory problems	Severe	Has cough, wheezing, and shortness of breath all the time. The person has great difficulty walking even short distances or climbing any stairs, feels tired when at rest, and is anxious.	0.408 (0.273–0.556)
Motor impairment	Asymptomatic		
Motor impairment	Mild	Has some difficulty in moving around but is able to walk without help.	0.01 (0.005–0.019)
Motor impairment	Moderate	Has some difficulty in moving around and difficulty in lifting and holding objects, dressing, and sitting upright, but is able to walk without help.	0.061 (0.04–0.089)
Motor impairment	Severe	Is unable to move around without help, and is not able to lift or hold objects, get dressed, or sit upright.	0.402 (0.268–0.545)
Speech problems	No		
Speech problems	Yes	Has difficulty speaking, and others find it difficult to understand.	0.051 (0.032–0.078)
Asymptomatic, but worry	Yes	Has a disease diagnosis that causes some worry but minimal interference with daily activities.	0.012 (0.006–0.023)

Modelling strategy

We use DisMod 2.1 as the main analytical tool for MND estimation. Prior settings are limited to 0 remission at all ages. We also constrain the super-region random effects for prevalence and incidence to -0.5 and 0.5 to account for spurious inflation of regional differences.

Claims data for 2000 and 2010 are adjusted via study covariates to account for systematically low estimates relative to the 2012 claims data. Implicit in this adjustment is the assumption that variation between years of claims data is a function of data collection inconsistencies and noise.

Similar to other cases we use GBD estimates of cause-specific mortality rate (CSMR) and excess mortality rate (EMR) in this model. The source and estimation of these rates are discussed elsewhere.

To assist the estimation process we use several country-level covariates.

Covariate	Measure	Beta	Exponentiated
Socio-demographic Index	prevalence	1.626<br>(1.148 - 1.971)	5.086<br>(3.152 - 7.178)
All MarketScan, year 2010	prevalence	-.1082<br>(-.1511 - -.0627)	.8974<br>(.8598 - .9392)
Absolute value of average latitude	prevalence	.0016<br>(7.5e-05 - .0041)	1.002<br>(1 - 1.004)
LDI (I$ per capita)	excess mortality rate	-.4999<br>(-.5 - -.4998)	.6066<br>(.6065 - .6067)
Mean BMI	prevalence	-.0922<br>(-.1336 - -.0581)	.912<br>(.8749 - .9435)
All MarketScan, year 2000	prevalence	-.1146<br>(-.1612 - -.0621)	.8918<br>(.8511 - .9398)

As described in the literature, BMI may be protective of MND. Accordingly, we have included mean BMI as a covariate to assist the estimation of prevalence within the disease model. As expected, the coefficient of BMI on MND prevalence is negative.

Although there are no known cures for MND, we expect disease management to differ globally – largely as a function of available resources. To capture this, we use the natural log of lagged distributed income per capita as a proxy to capture this relationship in the estimation of excess mortality.

To capture possible social and cultural risk factors or modifiers of MS prevalence, we include Socio-demographic Index as a covariate.

Because MND is a new cause for GBD 2015, we have no reference point relative to other GBD iterations.

Fatal Cancer estimation process

Input data and methodological summary for all cancers except for liver cancer and non-melanoma skin cancer

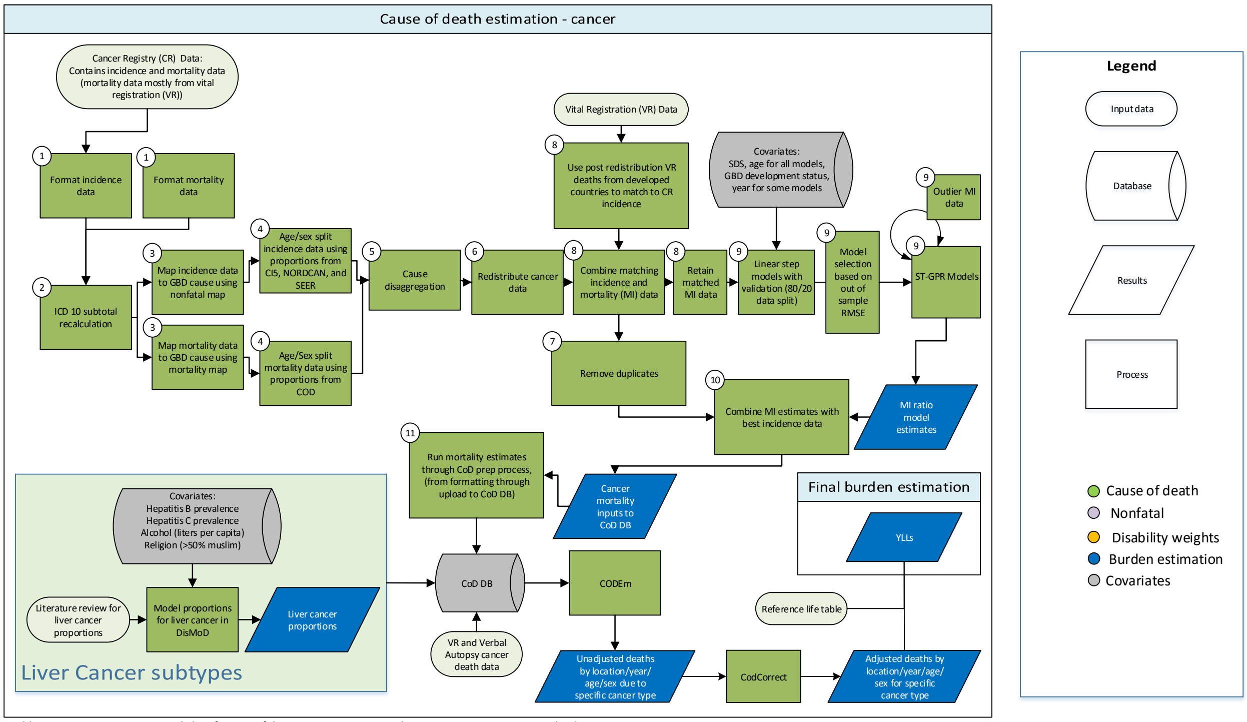

Abbreviations: ICD: International classification of diseases; CI5: Cancer Incidence in Five Continents, DB: database

Data

The Cause of Death (COD) database contains multiple sources of cancer mortality data. These sources include vital registration, verbal autopsy, and cancer registry data. The cancer registry mortality estimates that are uploaded into the COD database stem from cancer registry incidence data that have been transformed to mortality estimates through the use of mortality-to-incidence (MI) ratios.

Data-seeking processes

Cancer mortality data in the cause of death database other than cancer registry data

Sources for cancer mortality data other than cancer registry data are described in the COD database description (Part 2).

Cancer registry data

All cancer registry data used for GBD 2010 were also included for GBD 2013, and the majority of these data were also used for GBD 2015 unless superseded by newer data (see step 7 in flow chart and below). Most new data were added based on availability and collaborator recommendation. Some new data were acquired and approved for GBD 2013 but were received after the deadline for adding new data to GBD

2013. More than half (56%) of the final incidence data and 35% of the final MI model input data came from the Cancer Incidence in Five Continents series (CI5).[1–10]

Cancer registry data were most often downloaded from a publicly available webpage or provided by collaborators. Most cancer registries only report cancer incidence. However, if a cancer registry also reported cancer mortality, mortality data were also extracted from the source to be used in the mortality-to-incidence estimation.

Inclusion and exclusion criteria

Only population-based cancer registries were included, and only those that included all cancers (no specialty registries), data for all age groups, and data for both sexes. Pathology-based cancer registries were included if they had a defined population. Hospital-based cancer registries were not included.

Cancer registry data were excluded from either the final incidence data input or the MI model input if a more detailed source (eg, providing more detailed age or diagnostic groups) was available for the same population. Preference was given to registries with national coverage over those with only local coverage, except those from countries where the GBD study provides subnational estimates; thus some data were excluded because newly acquired national registry data could replace a regionally representative predecessor.

Data were excluded from the final incidence data input if the coverage population was unknown.

Bias of categories of input data

Cancer registry data can be biased in multiple ways. A high proportion of ill-defined cancer cases in the registry data requires redistribution of these cases to other cancers, which introduces a potential for bias. Changes between coding systems can lead to artificial differences in disease estimates; however, we adjust for this bias by mapping the different coding systems to the GBD causes. Underreporting of cancers that require advanced diagnostic techniques (eg, leukaemia and brain, pancreatic, and liver cancer) can be an issue in cancer registries from low-income countries. On the other hand, misclassification of metastatic sites as primary cancer can lead to overestimation of cancer sites that are common sites for metastases like brain or liver. Since many cancer registries are located in urban areas, the representativeness of the registry for the general population can also be problematic. The accuracy of mortality data reported in cancer registries usually depends on the quality of the vital registration system. If the vital registration system is incomplete or of poor quality, the mortality-to-incidence ratio can be biased to lower ratios.

Methods

Overall methodological process

See cancer flowchart.

Steps of analysis and data transformation processes

Cancer registry data went through multiple processing steps before integration with the COD database. First, the original data were transformed into standardised files, which included standardization of format, categorization, and registry names (#1 in flowchart).

Second, some cancer registries report individual codes as well as aggregated totals [eg, C18, C19, and C20 are reported individually but the aggregated group of C18-C20 (colorectal cancer) is also reported in the registry data]. The data processing step "subtotal recalculation" (#2 in flowchart) verifies these totals and subtracts the values of any individual codes from the aggregates.

In the third step (#3 in the flowchart), cancer registry incidence data and cancer registry mortality data are mapped to GBD causes. A different map is used for incidence and for mortality data because of the assumption that there are no deaths for certain cancers. One example is basal cell carcinoma of the skin. In the cancer registry incidence data, basal cell carcinoma is mapped to non-melanoma skin cancer (basal cell carcinoma). However, if basal cell skin cancer is recorded in the cancer registry mortality data, the deaths are instead mapped to non-melanoma skin cancer (squamous cell carcinoma) under the assumption that they were indeed misclassified squamous cell skin cancers. Other examples are benign or in situ neoplasms. Benign or in situ neoplasms found in the cancer registry incidence dataset were simply dropped from that dataset. The same neoplasms reported in a cancer registry mortality dataset were mapped to the respective invasive cancer (eg, melanoma in situ in the cancer registry incidence dataset was dropped from the dataset; melanoma in situ in the cancer registry mortality dataset was mapped to melanoma).

In the fourth data processing step (#4 in the flowchart) cancer registry data were standardised to the GBD age groups. Age-specific incidence rates were generated using CI5, SEER, and NORDCAN data, while age-specific mortality rates were generated from the CoD data through a method described in Part 2. Age-specific weights were then generated by applying the age-specific rates to a given registry population that required age-splitting to produce the expected number of cases/deaths for that registry by age. The expected number of cases/deaths for each sex, age, and cancer were then normalised to 1, creating final, age-specific proportions. These proportions were then applied to the total number of cases/deaths by sex and cancer to get the age-specific number of cases/deaths.

In the rare case that the cancer registry only contained data for both sexes combined, the now-age-specific cases/deaths were split and re-assigned to separate sexes using the same weights that are used for the age-splitting process. Starting from the expected number of deaths, proportions were generated by sex for each age (eg, if for ages 15 to 19 years old there are six expected deaths for males and four expected deaths for females, then 60% of the combined-sex deaths for ages 15–19 years would be assigned to males and the remaining 40% would be assigned to females).

In the fifth step (#5 in the flowchart) data for cause entries that are aggregates of GBD causes were redistributed. Examples of these aggregated causes include some registries reporting ICD10 codes C00-C14 together as, "lip, oral cavity, and pharyngeal cancer." These groups were broken down into sub-

causes that could be mapped to single GBD causes. In this example, those include lip and oral cavity cancer (C00-C08), nasopharyngeal cancer (C11), cancer of other parts of the pharynx (C09-C10, C12-C13), and "Malignant neoplasm of other and ill-defined sites in the lip, oral cavity, and pharynx" (C14). To redistribute the data, weights were created using the same "rate-applied-to-population" method employed in age-sex splitting (see step four above). For the undefined code (C14 in the example) an "average all cancer" weight was used, which was generated by adding all cases from SEER/NORDCAN/CI5 and dividing the total by the combined population. Then, proportions were generated by sub-cause for each aggregate cause as in the sex-splitting example above (see step four). The total number of cases from the aggregated group (C00-C14) was then recalculated for each subgroup and the undefined code (C14). C14 was then redistributed as a "garbage code" in step six. Distinct proportions were used for C44 (non-melanoma skin cancer) and C46 (Kaposi's sarcoma). Population data were not used to redistribute data for these ICD codes. Non-melanoma skin cancer processing is described under section "Input data and methodological summary for non-melanoma skin cancer (squamous-cell carcinoma)." C46 entries were redistributed as "other cancer," HIV, and C80 (other and unknown cancers) using proportions described in Part 2.

In the sixth step (#6 in the flowchart) unspecified codes ("garbage codes") were redistributed. Redistribution of cancer registry incidence and mortality data mirrored the process of the redistribution used in the cause of death database (Part 2).

In the seventh step (#7 in the flowchart) duplicate or redundant sources were removed from the processed cancer registry dataset. Duplicate sources were present if, for example, the cancer registry was part of the CI5 dataset but we also had data from the registry directly. Redundancies occurred and were removed as described in "Inclusion and Exclusion Criteria," where more detailed data were available, or when national registry data could replace regionally representative data. From here, two parallel selection processes were run to generate input data for the MI models and to generate incidence for final mortality estimation. Higher priority was given to registry data from the most standardised source when creating the final incidence input (generally CI5 data), whereas preference was given to registry data from sources with matching mortality and incidence for the MI model input (in order to reduce confounding due to oppositional input biases when matching the two data types).

In the eighth step (#8 in the flowchart) the processed incidence and mortality data from cancer registries were matched by cancer, age, sex, year, and location to generate MI ratios. Because some cancer registries do not report mortality data – even though high-quality vital registration system data are available to the registry's coverage area – processed vital registration mortality data from the CoD database were matched to the registry's incidence data for some countries. This was the case for certain registries in the following countries: Australia, Austria, Belgium, Bulgaria, Denmark, Estonia, Finland, Hungary, Iceland, Ireland, New Zealand, Norway, South Korea, and Switzerland.

The ninth step involved creating and selecting the MI models. All models were run separately by cancer, and the best model was selected from the following list (see Table below).

1. $\text{logit}\left(MI\,ratio_{c,a,s,t}\right) = \alpha + \beta_1 SDS_{c,t} + \sum_a^A \beta_2 I_a + \beta_3 I_s + \theta_c + \epsilon_{c,a,s,t}$
2. $\text{logit}\left(MI\,ratio_{c,a,s,t}\right) = \alpha + \beta_1 SDS_{c,t} + \sum_a^A \beta_2 I_a + \beta_3 I_s + \beta_4 t + \theta_c + \epsilon_{c,a,s,t}$
3. $\text{logit}\left(MI\,ratio_{c,a,s,t}\right) = \alpha + \beta_1 SDS_{c,t} + \sum_a^A \beta_2 I_a + \beta_3 I_s + \beta_4 DS + \theta_c + \epsilon_{c,a,s,t}$
4. $\text{logit}\left(MI\,ratio_{c,a,s,t}\right) = \alpha + \beta_1 SDS_{c,t} + \sum_a^A \beta_2 I_a + \beta_3 I_s + \beta_4 DS + \beta_5 t + \theta_c + \epsilon_{c,a,s,t}$
5. $\text{logit}\left(MI\,ratio_{c,a,s,t}\right) = \alpha + \beta_1 SDS_{c,t} + \sum_a^A \beta_2 I_a + \beta_3 I_s + \theta_c + \lambda_{SR}(SDS_{c,t}) + \beta_4 t + \epsilon_{c,a,s,t}$
6. $\text{logit}\left(MI\,ratio_{c,a,s,t}\right) = \alpha + \beta_1 SDS_{c,t} + \sum_a^A \beta_2 I_a + \beta_3 I_s + \theta_c + \lambda_{SR}(SDS_{c,t}) + \epsilon_{c,a,s,t}$
7. $\text{logit}\left(MI\,ratio_{c,a,s,t}\right) = \alpha + \beta_1 SDS_{c,t} + \sum_a^A \beta_2 I_a + \beta_3 I_s + \theta_c + \lambda_{SR}(SDS_{c,t}) + \beta_4 DS + \epsilon_{c,a,s,t}$
8. $\text{logit}\left(MI\,ratio_{c,a,s,t}\right) = \alpha + \beta_1 SDS_{c,t} + \sum_a^A \beta_2 I_a + \beta_3 I_s + \theta_c + \lambda_{SR}(SDS_{c,t}) +$
   $\beta_4 t + \beta_5 DS + \epsilon_{c,a,s,t}$

c: country; a: age group; t: time (years); s: sex
I: indicator variable
DS: binary variable for development status
θ_c: random effect by country (intercept)
$\lambda_{SR}(SDSc, t)$: random effect modifier between SDI and super-region (slope)
$\epsilon_{c,a,s,t}$: error term

Table: MI models

All models were tested at multiple stages before creating the final model output. Models were initiated with an SDI covariate (Socio-demographic Index) and first tested using the complete input dataset (Part 4). If after that initial test the SDI covariate's coefficient was negative (as expected), the next step was to outlier any data point for which the residual from the prediction was greater than three times the MAD from the mean residual. Next, data were marked as outliers due to a random effect criterion: if the country-level random effect for a lower-income country was lower than the random effect for the USA, all data points for that country were marked as outliers. This process was run iteratively until all lower-income countries had country-level random effects greater than that of the USA. All data points marked outliers were dropped from the final dataset, and that dataset was used to create the final model predictions.

If the SDI coefficient was found to be positive (unexpected) after the initial SDI test, it was assumed to indicate an excess of unrealistic data in the input dataset. To remove these unrealistic data, SDI was temporarily removed from the model formula. The model proceeded as above without SDI until all unrealistic data points were removed and the SDI coefficient was found to be negative. Unrealistic data were marked as outliers using the same residual MAD and random effect methodology described above. Once SDI was established as negative (expected) the model proceeded as usual.

To select the best model formula, the initial model results were tested by comparing mean MI predictions and the mean root-mean-squared error (RMSE) values of 10 random samples of 80%/20% splits from the input dataset. Mean MI predictions were compared between developing and developed countries. Models were eliminated if the mean MI for developing countries was lower than the mean MI ratio for developed countries. For RMSE testing, the dataset was split into an 80% dataset for model development

and a 20% dataset for model testing. The process was repeated 10 times. The best model for each cancer was selected based on the lowest mean out-of-sample RMSE from those models remaining after checking the mean MI. The table below contains the final models selected for each cancer.

Cancer	Final model number (see numbering above)
Ovarian cancer	1
Uterine cancer	1
Gallbladder cancer	1
Kidney cancer	1
Larynx cancer	1
Acute lymphoid leukaemia	1
Chronic myeloid leukaemia	1
Lip and oral cavity cancer	1
Pancreatic cancer	1
Hodgkin’s lymphoma	2
Acute myeloid leukaemia	2
Chronic lymphoid leukaemia	2
Malignant skin melanoma	2
Bladder cancer	3
Brain and nervous system cancer	3
Oesophageal cancer	3
Tracheal, bronchus, and lung cancer	3
Mesothelioma	3
Multiple myeloma	3

Other cancer	3
Prostate cancer	4
Testicular cancer	4
Breast cancer	4
Colorectal cancer	4
Leukaemia	4
Liver cancer	4
Non-Hodgkin lymphoma	4
Non-melanoma skin cancer (squamous cell carcinoma)	4
Stomach cancer	4
Nasopharynx cancer	6
Cervical cancer	7
Other pharynx cancer	8
Thyroid cancer	8

Table: Final model selections

Once the best models were selected, data points were manually outliered based on the results of the first run of the model algorithm. Data points were outliered if they clearly influenced the model in an unrealistic way. For example, a data point was marked as an outlier if it created a single-year, single-age-group spike in model predictions. This was mainly the case in countries with a small number of cases or deaths, or in age groups with small numbers of cases or deaths. Manual outliers were removed from the input dataset prior to initiating the second run of the model algorithm.

After best models were selected, all final outliers were dropped from the data input, and final linear predictions were created, the final linear predictions and residuals were used as input for space-time smoothing. Space-time smoothing is a spatiotemporal regression to smooth residuals over space, time, and age.[11–13] The weighted residuals were added to the linear model predictions and used as priors for the third stage, a Gaussian process regression (GPR) implementing a Matern covariance function.[13–18] GPR is a nonparametric technique for interpolating non-linear trends that has been used extensively in the estimation of time series data. Final MI ratio predictions with 95% uncertainty intervals were obtained by back-transforming 1,000 draws from the posterior distribution.

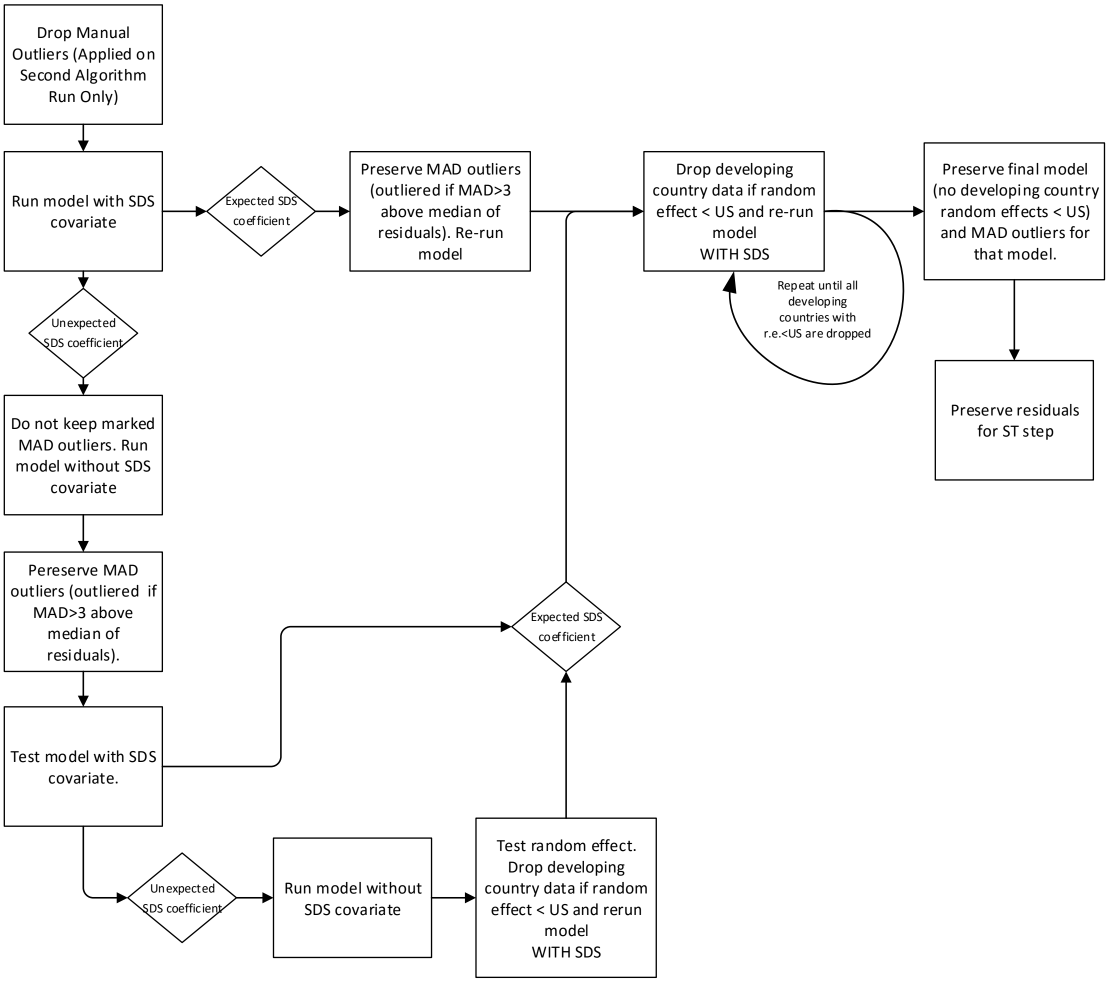

Figure 1: MI model estimation algorithm

Step 9 has undergone a revision compared to GBD 2010 and GBD 2013. In GBD 2010 and GBD 2013 only one model was used to predict all MI ratios, whereas for GBD 2015 we generated multiple models and chose a best model based on out-of-sample validation. Another major difference is that LDI (lagged distributed income) was used as a covariate in previous versions and was replaced by SDI for GBD 2015.

Final MI ratios were matched with the cancer registry incidence dataset in the ninth step (#10 in the flowchart) to generate mortality estimates (Incidence * Mortality/Incidence = Mortality). The final mortality estimates were then uploaded into the COD database (#11 in the flowchart).

After transforming cancer registry incidence data to mortality estimates, the modelling strategy followed the general CODEm process as described in Part 3.

Results

Interpretation of results

Cancer mortality estimates for GBD 2015 can differ from the GBD 2013 results for multiple reasons. First, compared to GBD 2013 more cancer mortality data were added to the cause of death database. Second, we added sources for cancer registry data, which were transformed into mortality estimates by using the MI ratio. Third, mapping of cancer ICD codes to the GBD cancer causes was updated slightly based on collaborator comments. One example is that mapping for the ICD10 code D46 (myelodysplastic syndrome) was changed from "other cancer" to "undefined cancer" for later redistribution to non-Hodgkin lymphoma and leukaemia. The one major mapping change was the addition of subtypes for leukaemia and non-melanoma skin cancer. Fourth, the method to redistribute undefined causes of death or undefined cancers changed compared to GBD 2013. Models for redistribution are now performed regionally rather than by super-region. Fifth, we updated and refined the mortality-to-incidence ratio estimation compared to GBD 2013. Whereas for GBD 2010 and GBD 2013 a single model was used to estimate the MI ratios for each location, by cancer, sex, and age, we developed multiple plausible models for GBD 2015 and chose the best model based on out-of-sample validation. Sixth, we reviewed the covariate inputs for the CODEm models and changed covariates when updated or improved covariates were available. Seventh, many covariates used in CODEm models were updated for GBD 2015 (Part 4).

The other group producing country-level cancer mortality estimates is the International Agency for Research on Cancer (IARC) with their GLOBOCAN database. Significantly different methods between the GBD study and GLOBOCAN can lead to differences in results. Whereas estimates in GLOBOCAN are based on the assumption that there are "In theory, […] as many methods as countries,"[19] the cancer estimation process for the GBD study follows a coherent, well-documented method for all cancers, which allows cross-validation of models as well as determination of uncertainty. Another major difference is the ability in the GBD study to adjust single cause estimates to the all-cause mortality, which is being determined independently. This also allows us to adjust individual causes of death to the all-cause mortality envelope which permits us to correct for the underdiagnosis of cancer in countries with inadequate diagnostic resources. Redistribution of a fraction of undefined causes of death to certain cancers is another methodical advantage the GBD study has over GLOBOCAN, and estimates for cancer mortality can therefore differ substantially in countries with a large proportion of undefined causes of deaths in their vital registration data or a large proportion of undefined cancer cases in their cancer registry data.

Limitations

There are certain limitations to consider when interpreting the GBD mortality cancer estimates. First, even though every effort is made to include the most recently available data for each country, data-seeking resources are not limitless and new data cannot always be accessed as soon as they are made available. It is therefore possible that the GBD study does not include all available data sources for cancer incidence or cancer mortality. Second, different redistribution methods can potentially change the cancer estimates substantially if the data sources used for the estimated location contain a large number of undefined causes; however, neglecting to account for these undefined deaths would likely introduce an

even greater bias in the disease estimates. Third, using mortality-to-incidence ratios to transform cancer registry incidence data to mortality estimates requires accurate MI ratios. For GBD 2015 the methodology to estimate MI ratios was improved with development of multiple different models and implementation of model cross-validation, but the method is still sensitive to underdiagnosis of cancer cases or underascertainment of cancer deaths. However, given that the majority of data used for the cancer mortality estimation come from vital registration data and not cancer registry data this is not a major limitation.

Non-fatal Cancer estimation process

All cancers except for non-melanoma skin cancer (basal cell carcinoma and squamous cell carcinoma)

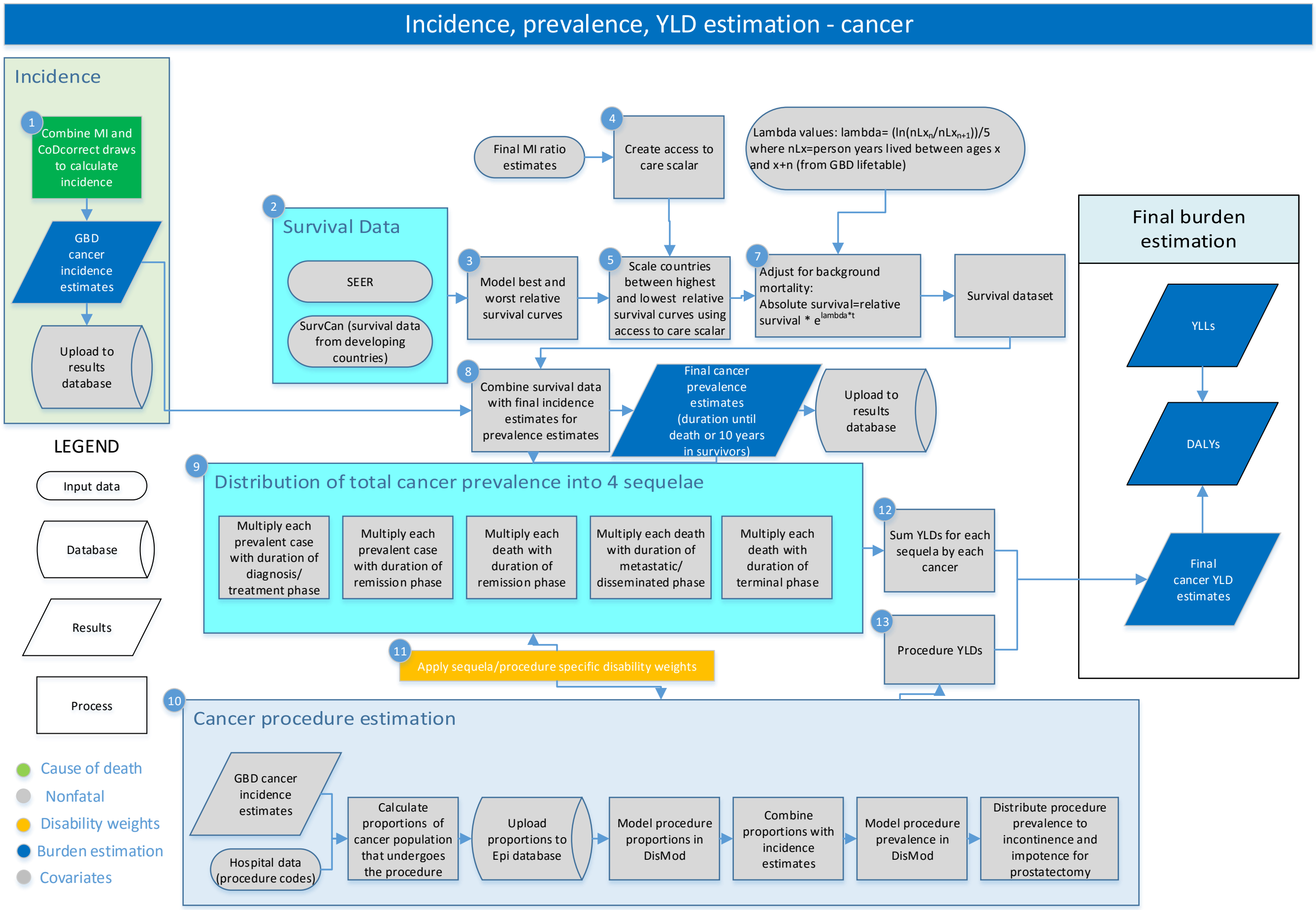

Input data and methodological appendix

Case definition

For GBD 2015, the incidence, prevalence, and disability are estimated for all cancers as defined in ICD-10 (C00-C96). Prevalence for all cancers is estimated for a maximum of 10 years after incidence as in GBD 2013. Prevalence extending beyond the 10-year period is only estimated for permanent sequelae from procedures.

To estimate disability for each cancer, total prevalence is split into four sequelae: 1. diagnosis and primary therapy; 2. controlled phase; 3. metastatic phase; and 4. terminal phase. Diagnosis and primary therapy

are defined as the time from symptoms onset to end of treatment. Controlled phase is defined as the time after finishing primary treatment and either cure (defined as survival after 10 years) or metastatic phase. Metastatic phase is defined as the time period of intensive treatment for metastatic disease, terminal phase is defined as the one month period prior to death. Each of these sequelae has a separate disability weight. Additional disability is estimated for breast cancer (disability due to mastectomy), larynx cancer (disability due to laryngectomy), colon and rectum cancer (disability due to stoma), bladder cancer (disability due to incontinence), and prostatectomy (disability due to incontinence and impotence). The associated ICD codes for neoplasms estimated for GBD 2015 are listed below.

Table 1. GBD cancer causes with respective ICD codes

GBD cause	ICD9	ICD10
Bladder cancer	188.0-188.9	C67-C67.9
Brain and nervous system cancer	191.0-192.9	C70-C72.9
Breast cancer	174.0-175.9	C50-C50.929
Cervical cancer	180.0-180.9	C53-C53.9, D26.0
Colon and rectum cancer	153.0-154.9, 209.1-209.17	C18-C21.9
Oesophageal cancer	150.0-150.9	C15-C15.9
Gallbladder and biliary tract cancer	156.0-156.9, 209.25-209.27	C23-C24.9
Hodgkin's disease	201.0-201.98	C81-C81.99
Kidney cancer	189.0-189.1, 209.24	C64-C65.9
Larynx cancer	161.0-161.9	C32-C32.9
Leukaemia	208.0-208.92	C94.1, C94.7-C95.92
Acute lymphoid leukaemia ALL	204.0-204.02	C91.0-C91.02
Acute myeloid leukaemia AML	205.0-205.02, 205.3-205.32, 206.0-206.02, 207.0	C92.0-C92.02, C92.3-C92.62, C93.0-C93.02, C94.0-C94.02, C94.2-C94.22, C94.4-C94.5
Chronic lymphoid leukaemia CLL	204.1-204.12	C91.1-C91.12
Chronic myeloid leukaemia CML	205.1-205.12, 206.1-206.12, 207.1	C92.1-C92.12
lymphoid leukaemia	204.0, 204.2-204.92	C91, C91.2-C91.92
myeloid leukaemia	205.0, 205.2-205.22, 205.8-206.0, 206.2-207.9	C92, C92.2-C92.22, C92.7-C93, C93.1-C94, C94.3-C94.32, C94.6
Liver cancer	155.0-155.9	C22-C22.9

Lung, bronchus, and trachea cancer	162.0-162.9, 209.21	C33-C34.92
Non-Hodgkin lymphoma	200.0-200.9, 202.0-202.98	C82-C86.6, C96-C96.9
Malignant skin melanoma	172.0-172.9	C43-C43.9
Mesothelioma	158.9, 163.0-163.9	C45-C45.9
Oral and lip cancer	140.0-145.9	C0-C08.9
Multiple myeloma and immunoproliferative diseases	203.0-203.9	C88-C90.9
Nasopharynx cancer	147.0-147.9	C11-C11.9
Non-melanoma skin cancer basal cell carcinoma	173.0-173.01, 173.09-173.11, 173.19-173.21, 173.29-173.31, 173.39-173.41, 173.49-173.51, 173.59-173.61, 173.69-173.71, 173.79-173.81, 173.89-173.91, 173.99, 216.0-216.9, 232.0-232.9, 238.2	C44.0-C44.01, C44.09-C44.119, C44.19-C44.219, C44.29-C44.319, C44.39-C44.41, C44.49-C44.519, C44.59-C44.619, C44.69-C44.719, C44.79-C44.80, C44.82-C44.91, C44.99
Non-melanoma skin cancer squamous-cell carcinoma	173.02, 173.12, 173.22, 173.32, 173.42, 173.52, 173.62, 173.72, 173.82, 173.92	C44.02, C44.12-C44.129, C44.22-C44.229, C44.32-C44.329, C44.42, C44.52-C44.529, C44.62-C44.629, C44.72-C44.729, C44.81, C44.92, D04-D04.9, D49.2
Other neoplasms	158.0-158.8, 209.4-209.57, 209.61, 209.63-209.67, 210.0-211.8, 212.0-212.8, 213.0-215.9, 217.0-221.8, 222.0-	D00.00-D00.2, D01.0-D01.3, D02.0-D02.3, D03-D03.9, D05-D06.9, D07.0-D07.2, D07.4-D07.5, D09.0, D09.2-D09.3, D09.8,

	222.8, 223.0-223.89, 224.0-229.0, 229.8, 230.1-230.8, 231.0-231.2, 233.0-233.2, 233.31-233.32, 233.4-233.5, 233.7, 234.0-234.8, 235.0, 235.4, 235.6-235.8, 236.1-236.2, 236.4-236.5, 236.7, 236.91-237.3, 237.5-238.1, 238.3-238.5, 239.2-239.4, 239.6	D10.0-D10.7, D11-D12.9, D13.0-D13.7, D14.0-D14.32, D15-D24.9, D27-D27.9, D28.0-D28.7, D29.0-D29.8, D30.0-D30.8, D31-D36.7, D37.01-D37.5, D38.0-D38.5, D39.1-D39.2, D39.8, D40.0-D40.8, D41.0-D41.8, D42-D43.9, D44.0-D44.8, D45-D45.9, D47-D47.0, D47.2-D47.9, D48.0-D48.7, D49.3-D49.4, D49.6
Other cancers	152.0-152.9, 160.0-160.9, 164.0-164.9, 170.0-171.9, 181.0-181.9, 182.9, 183.2-183.8, 184.0-184.4, 184.8, 187.1-187.8, 189.2-189.8, 190.0-190.9, 194.0-194.8, 209.0-209.03, 209.22, 209.31-209.36	C17-C17.9, C3-C31.9, C37-C38.8, C4-C41.9, C47-C5, C51-C52.9, C57-C57.8, C58-C58.0, C60-C60.9, C63-C63.8, C66-C66.9, C68.0-C68.8, C69-C7, C74-C75.8, D49.81
Other pharynx cancer	146.0-146.9, 148.0-148.9	C09-C10.9, C12-C13.9
Ovarian cancer	183.0	C56-C56.9
Pancreatic cancer	157.0-157.9	C25-C25.9
Prostate cancer	185.0-185.9	C61-C61.9
Stomach cancer	151.0-151.9, 209.23	C16-C16.9
Testicular cancer	186.0-186.9	C62-C62.92
Thyroid cancer	193.0-193.9	C73-C73.9
Uterine cancer	182.0-182.8	C54-C54.9

Garbage code	149.0-149.9, 159.0-159.9, 165.0-165.9, 169.0, 173.0, 176.0-179.9, 183.9-184.0, 184.5, 184.9, 187.0, 187.9, 189.0, 189.9, 194.9-199.9, 209.0, 209.2, 209.29-209.3, 209.6, 209.62, 209.69-210.0, 211.0, 211.9-212.0, 212.9, 221.0, 221.9-222.0, 222.9-223.0, 223.9, 229.0-229.1, 229.9-230.0, 230.9-231.0, 231.8-231.9, 233.0, 233.3, 233.39, 233.6, 233.9-234.0, 234.9-235.3, 235.5, 235.9-236.0, 236.3, 236.6, 236.9, 237.4, 238.0, 238.6-239.1, 239.5, 239.7-239.9	C14-C14.9, C26-C29, C35-C36, C39-C39.9, C42, C44, C46-C46.9, C55-C55.9, C57.9, C59-C6, C63.9, C68, C68.9, C75.9-C80.9, C87, C97-D00.0, D01, D01.4-D02, D02.4-D02.9, D07, D07.3-D07.39, D07.6-D09, D09.1-D09.19, D09.7, D09.9-D10, D10.9, D13, D13.9-D14, D14.4, D28, D28.9-D29, D29.9-D30, D30.9, D36.9-D37.0, D37.6-D38, D38.6-D39.0, D39.7, D39.9-D40, D40.9-D41, D41.9, D44, D44.9, D46-D46.9, D47.1, D48, D48.9-D49.1, D49.5, D49.7-D49.8, D49.89-D49.9

Input data

Cancer incidence is directly estimated from cancer mortality using mortality to incidence ratios. Data sources for cancer mortality are described in detail elsewhere.[1] Data sources to scale countries between a hypothetical best- and a hypothetical worst-case survival remained the same as in GBD 2013 where we used SEER 2010 data for the "best case" survival and a combination of the 1950 US Mortality Files with "Cancer Survival in Africa, Asia, the Caribbean, and Central America" (SurvCan) data for the worst case survival.[2–4] For mesothelioma, gallbladder cancer, and the leukaemia subtypes SEER 1973 survival data for the lower boundary was used since these cancers are not included in the US Mortality Files from 1950. To estimate the proportion of cancer patients undergoing procedures we used SEER data form 1983 to 2008[2], Canada Hospital Data from 1994 to 2009[5], and Mexico Hospital Data from 2001 to 2009[6]. Data sources used to adjust procedure sequelae will be listed below.

Modelling strategy

Estimation of cancer mortality and mortality to incidence ratio estimation has been described in the GBD 2015 Mortality and Causes of Death capstone paper.[1] The final GBD cancer mortality estimates are being

transformed to incidence estimate by using the separately estimated MI ratios. To summarise the mortality to incidence ratio estimation process, processed incidence and mortality data from cancer registries were matched by cancer, age, sex, year, and location to generate MI ratios. Multiple MI models with different sets of covariates were created. All models were run separately by cancer, and the best model was selected from the following list:

1. $\text{logit}\left(MI\ ratio_{c,a,s,t}\right) = \alpha + \beta_1 SDI_{c,t} + \sum_a^A \beta_2 I_a + \beta_3 I_s + \theta_c + \epsilon_{c,a,s,t}$
2. $\text{logit}\left(MI\ ratio_{c,a,s,t}\right) = \alpha + \beta_1 SDI_{c,t} + \sum_a^A \beta_2 I_a + \beta_3 I_s + \beta_4 t + \theta_c + \epsilon_{c,a,s,t}$
3. $\text{logit}\left(MI\ ratio_{c,a,s,t}\right) = \alpha + \beta_1 SDI_{c,t} + \sum_a^A \beta_2 I_a + \beta_3 I_s + \beta_4 DS + \theta_c + \epsilon_{c,a,s,t}$
4. $\text{logit}\left(MI\ ratio_{c,a,s,t}\right) = \alpha + \beta_1 SDI_{c,t} + \sum_a^A \beta_2 I_a + \beta_3 I_s + \beta_4 DS + \beta_5 t + \theta_c + \epsilon_{c,a,s,t}$
5. $\text{logit}\left(MI\ ratio_{c,a,s,t}\right) = \alpha + \beta_1 SDI_{c,t} + \sum_a^A \beta_2 I_a + \beta_3 I_s + \theta_c + \lambda_{SR}(SDS_{c,t}) + \beta_4 t + \epsilon_{c,a,s,t}$
6. $\text{logit}\left(MI\ ratio_{c,a,s,t}\right) = \alpha + \beta_1 SDI_{c,t} + \sum_a^A \beta_2 I_a + \beta_3 I_s + \theta_c + \lambda_{SR}(SDS_{c,t}) + \epsilon_{c,a,s,t}$
7. $\text{logit}\left(MI\ ratio_{c,a,s,t}\right) = \alpha + \beta_1 SDI_{c,t} + \sum_a^A \beta_2 I_a + \beta_3 I_s + \theta_c + \lambda_{SR}(SDS_{c,t}) + \beta_4 DS + \epsilon_{c,a,s,t}$
8. $\text{logit}\left(MI\ ratio_{c,a,s,t}\right) = \alpha + \beta_1 SDI_{c,t} + \sum_a^A \beta_2 I_a + \beta_3 I_s + \theta_c + \lambda_{SR}(SDS_{c,t}) +$
   $\beta_4 t + \beta_5 DS + \epsilon_{c,a,s,t}$

c: country, a: age group, t: time (years); s: sex
SDI: Socio-demographic Index (index using log lag dependent income per capita (LDI), average educational attainment in the population over age 15, and total fertility rate (TFR))
I: indicator variable
DS: binary variable for development status
θ_c: random effect by country (intercept)
$\lambda_{SR}(SDIc, t)$: random effect modifier between SDI and superregion (slope)
$\epsilon_{c,a,s,t}$: error term
All models were tested at multiple stages before generating final predictions. Models were initiated with SDI (Socio-demographic Index) as covariate and first tested using the complete input dataset. If after that initial test the SDI covariate's coefficient was negative (as expected), the next step was to outlier any data point for which the residual from the prediction was greater than three times the MAD from the mean residual. Next, data were marked as outliers due to a random effect criterion: if the country-level random effect for a developing country was lower than the random effect for the USA, all data points for that country were marked as outliers. This process was run iteratively until all developing countries had country-level random effects greater than that of the USA. All data points marked outliers were dropped from the final dataset, and that dataset was used to create the final model predictions.

If the SDI coefficient was found to be positive (unexpected) after the initial SDI test, it was assumed to indicate an excess of unrealistic data in the input dataset. To remove these unrealistic data, SDI was temporarily removed from the model formula. The model proceeded as above without SDI until all unrealistic data points were removed and the SDI coefficient was found to be negative. Unrealistic data were marked as outliers using the same residual MAD and random effect methodology described above. Once SDI was established as negative (expected) the model proceeded as usual.

To select the best model formula, the initial model results were tested by comparing mean MI predictions and the mean root-mean-squared error (RMSE) values of ten random samples of 80%/20% splits from the

input dataset. Mean MI predictions were compared between developing and developed countries. Models were eliminated if the mean MI for developed countries was higher than the mean MI ratio for developing countries. For RMSE testing, the dataset was split into an 80% dataset for model development and a 20% dataset for model testing. The process was repeated ten times. The best model for each cancer was selected based on the lowest mean out-of-sample RMSE from those models remaining after checking the mean MI. Table 2 contains the final models selected for each cancer.

**Table 2. Final MI ratio model selection**	
Cancer	Final model number (for model numbers, see text)
Ovarian cancer	1
Uterine cancer	1
Gallbladder cancer	1
Kidney cancer	1
Larynx cancer	1
Acute lymphoid leukaemia	1
Chronic myeloid leukaemia	1
Lip and oral cavity cancer	1
Pancreatic cancer	1
Hodgkin's lymphoma	2
Acute myeloid leukaemia	2
Chronic lymphoid leukaemia	2
Malignant skin melanoma	2
Bladder cancer	3
Brain and nervous system cancer	3

Oesophageal cancer	3
Tracheal, bronchus, and lung cancer	3
Mesothelioma	3
Multiple myeloma	3
Other cancer	3
Prostate cancer	4
Testicular cancer	4
Breast cancer	4
Colorectal cancer	4
Leukaemia	4
Liver cancer	4
Non-Hodgkin lymphoma	4
Non-melanoma skin cancer (squamous cell carcinoma)	4
Stomach cancer	4
Nasopharynx cancer	6
Cervical cancer	7
Other pharynx cancer	8
Thyroid cancer	8

Once the best models were selected, data points were manually outliered based on the results of the first run of the model algorithm. Data points were outliered if they clearly influenced the model in an unrealistic way. For example, a data point was marked as an outlier if it created a single-year, single age group spike in model predictions. This was mainly the case in countries with a small number of cases or deaths, or in age groups with small numbers of cases or deaths. Manual outliers were removed from the input dataset prior to initiating the second run of the model algorithm.

After best models were selected, all final outliers were dropped from the data input, and final linear predictions were created, the final linear predictions and residuals were used as input for space-time smoothing. The weighted residuals were added to the linear model predictions and used as priors for the third stage, a Gaussian process regression (GPR) implementing a Matern covariance function. Final MI ratio predictions with 95% uncertainty intervals were obtained by back-transforming 1,000 draws from the posterior distribution. The MI ratio estimation has undergone a revision compared to the GBD 2010 and GBD 2013. Whereas in GBD 2010 and GBD 2013 only one model was used to predict all MI ratios, for GBD 2015 we generated multiple models and chose a best model based on out-of-sample validation. Another major difference is that LDI (lagged distributed income) was used as a covariate, which was replaced by SDI (Socio-demographic Index) for GBD 2015. Final MI ratio estimates at the 1,000 draw level were combined with final mortality estimates (as well at the 1,000 draw level) to generate incidence estimates. It was assumed that uncertainty in the MI ratio is independent of uncertainty in the estimated mortality.

After transforming the final GBD cancer mortality estimates to incidence estimates using the 1,000 mortality draws and the 1,000 MI ratio draws (step 1 in the flowchart), incidence was combined with the relative yearly survival estimates up to 10 years (step 8 in the flowchart). To estimate cancer prevalence, relative cancer survival was estimated by scaling cancer-specific survival between the "best case" and "worst case" survival, using the survival data sources listed above (steps 2, 3, and 5 in the flowchart). To transform relative to absolute survival (adjusting for background mortality), GBD 2015 lifetables were used (steps 6 and 7 in the flowchart) to calculate lambda values: lambda= (ln(nLxn/nLxn+1))/5 where nLx=person years lived between ages x and x+n (from GBD lifetable). Absolute survival was then calculated using an exponential survival function (absolute survival = relative survival * $e^{lambda*t}$).

The access to cancer care variable to scale countries between the best and worst case survival was estimated using the same method as for GBD 2013 (step 4 in the flowchart)[7]:

$$Access\ to\ care = 1 - \frac{Age\ standardized\ MI\ ratio_{cys} - Age\ standardized\ MI\ ratio_{min}}{Age\ standardized\ MI\ ratio_{max} - Age\ standardized\ MI\ ratio_{min}}$$

c=country; y=year; s=sex; Age-standardised MI $ratio_{min}$=lowest MI ratio for all countries and years; Age standardised MI $ratio_{max}$=highest MI ratio for all countries and years

Survivors beyond 10 years were considered cured. The survivor population prevalence was divided into two sequelae (1. diagnosis and primary therapy; 2. controlled phase). The yearly prevalence of the population that did not survive beyond 10 years was then divided into the four sequelae by assigning the fixed durations for the diagnosis and primary therapy phase, metastatic phase, and terminal phase and assigning the remaining prevalence to the controlled phase (step 9 in the flowchart). Duration of the treatment sequelae (1. diagnosis and primary therapy; 2. controlled phases; 3. metastatic phase; 4. terminal phase) remained the same as for GBD 2013.[7] Table 3 lists the duration including sources used.

Table 3. Duration of four prevalence sequelae by cancer					
	Diagnosis/ Treatment (months)	Remission	Disseminated/metastatic (months)	Note	Terminal (months)
Oesophageal cancer	5[8]	Calculated based on remainder of time after attributing other sequelae.	4.6[9]	SEER Summary Stage 1997 (Distant site/node involved) 1995–2000	1 month
Stomach cancer	5.2[8]		3.88[9]	SEER Summary Stage 1997 (Distant site/node involved) 1995–2000	
Liver cancer	4		2.51[9]	SEER Summary Stage 1997 (Distant site/node involved) 1995–2000	
Larynx cancer	5.3[8]		8.84[9]	SEER Stage IVc	
Lung cancer	3.3[10]		4.51[9]	SEER Summary Stage 1997 (Distant site/node involved) 1995–2000	
Breast cancer	3[10]		17.7[9]	SEER Summary Stage 1997 (Distant site/node involved) 1995–2000	
Cervical cancer	4.8[8]		9.21[9]	SEER Summary Stage 1997 (Distant site/node involved) 1995–2000	
Uterine cancer	4.6[8]		11.6[9]	SEER Summary Stage 1997 (Distant site/node involved) 1995–2000	
Prostate cancer	4[10]		30.35[9]	SEER Summary Stage 1997 (Distant site/node involved) 1995–2000	
Colorectal cancer	4[10]		9.69[9]	SEER Summary Stage 1997 (Distant site/node involved) 1995–2000	
Oral cancer	5.3[8]		9.33[9]	SEER Stage IVc	
Nasopharyngeal cancer	5.3[8]		13.19[9]	SEER Stage IVc	
Cancer of other part of pharynx	5.3[8]		7.91[9]	SEER Stage IVc	
Gallbladder cancer	4		3.47[9]	SEER Summary Stage 1997 (Distant site/node involved) 1995–2000	
Pancreas cancer	4.1[8]		2.54[9]	SEER Summary Stage 1997 (Distant site/node involved) 1995–2000	

Melanoma	2.9[11]		7.18[9]	SEER Summary Stage 1997 (Distant site/node involved) 1995–2000
NMSC (squamous cell carcinoma)	2.9[11]		17[12]	
Ovarian cancer	3.2[10]		25.6[9]	SEER Summary Stage 1997 (Distant site/node involved) 1995–2000
Testicular cancer	3.7[8]		19.47[9]	SEER Stage III
Kidney cancer	5.3[8]		5.38[9]	SEER Summary Stage 1997 (Distant site/node involved) 1995–2000
Bladder cancer	5.1[8]		5.8[9]	SEER Summary Stage 1997 (Distant site/node involved) 1995–2000
Brain cancer	5		6.93[9]	SEER Median age standardised survival all patients, all years
Thyroid cancer	3		19.39[9]	SEER Stage IVc
Mesothelioma	4		7.75[9]	SEER Summary Stage 1997 (Distant site/node involved) 1995–2000
Hodgkin's lymphoma	3.7[10]		26[13]	
Non-Hodgkin lymphoma	3.7[10]		7.7[13]	
Multiple myeloma	7[8]		36.82[9]	SEER Median age standardised survival all patients, all years
Leukaemia[8]	5		43.67[9]	SEER Median age standardised survival all patients, all years
ALL	12		7.02[9]	SEER Median age standardised survival all patients, all years
AML	6		4.6[9]	SEER Median age standardised survival all patients, all years
CLL	6		48[14]	
CML	6		4.6[9]	SEER Median age standardised survival for AML (patients with CML die in blast crisis, which is

				treated like AML) all patients, all years	
Other	4.4 (mean of other cancer durations)		15.81[9]	SEER Median age standardised survival all patients, all years	

For cancer-specific procedure sequelae, hospital data were used to estimate the number of cancer patients undergoing mastectomy, laryngectomy, stoma, prostatectomy, and cystectomy (step 10 in the flowchart). These proportions remained the same as in GBD 2013.[7] Proportions were generated by dividing the rate of procedures generated from the diagnostic codes in the hospital dataset and the coverage population by the GBD age-, and sex-specific disease incidence rates for that country. Diagnostic codes used are listed in table 4:

Table 4. Procedure codes used to estimate cancer procedure proportions

Procedure	Cancer	Procedure code (ICD-9_CM)
Mastectomy	Breast cancer	854, 8541, 8542, 8543, 8544, 8545,<br><br>8546, 8547, 8548
Laryngectomy	Larynx cancer	301, 303, 304, 3029
Stoma	Colon and rectum cancer	461, 4610, 4611, 4613, 4862
Cystectomy	Bladder cancer	5771, 5779
Prostatectomy	Prostate cancer	603, 604, 605, 606, 6062

To estimate procedure-related disability for certain cancers, the procedure proportions (proportion of cancer population that undergoes procedures) from hospital data was used as input for a proportion model in DisMod-MR 2.1 in order to estimate the proportions for all locations, by age, and by sex. Since colostomy or ileostomy procedures are done for reasons other than cancer a literature review was done to determine the proportion of ostomies due to colorectal cancer. The “all-cause” colostomy proportions were multiplied by 0.58 based on the results of the literature review showing that on average 58% of ostomies are done for colorectal cancer.[15–17] The final procedure proportions were applied to the incidence cases of the respective cancers to determine the incident cases of the cancer population that underwent procedures. These incident cases were used again as an input for DisMod-MR 2.1 with a remission specification of zero and the cause-specific mortality of the specific cancer to obtain prevalence of the sequela. By using cause-specific mortality, the simplifying assumption was made that survival for cancer patients undergoing procedures is the same as for cancer patients who do not need a procedure. Since disability associated with prostatectomy comes from impotence and incontinence and not from the

prostatectomy itself, 18% of the prostatectomy prevalence was assumed to be incontinent and 55% was assumed to be impotent based on a literature review done for GBD 2013.[18–25]

Since all sequelae for a cause need to be mutually exclusive, the controlled phase for the cancers with additional procedure-related disability was adjusted to only include the population without procedure-related disability (= controlled phases prevalence of the total population – controlled phase prevalence of the proportion that experienced procedure related disability) (step 11 in the flowchart). The disability weight for the prevalence of the population that experiences additional disability was adjusted to reflect the combined disability of the controlled phase as well as the procedure.

Lastly, the procedure sequelae prevalence and general sequelae prevalence were multiplied with disability weights (Table 5) for the procedures to obtain the number of YLDs (steps 11, 12, 13 in the flowchart). The sum of these YLDs is the final YLD estimate associated with each cancer.

Table 5. Lay description and disability weights

Health state	Lay description	Estimate	Uncertainty interval	
Cancer, diagnosis and primary therapy (cancer_diagnosis)	This person has pain, nausea, fatigue, weight loss, and high anxiety.	0.288	0.193	0.399
Cancer, controlled phase (generic_medication)	This person has a chronic disease that requires medication every day and causes some worry but minimal interference with daily activities.	0.049	0.031	0.072
Cancer, metastatic (cancer_metastatic)	This person has severe pain, extreme fatigue, weight loss, and high anxiety.	0.451	0.307	0.600
Terminal phase, with medication (cancer_terminal_treat)	This person has lost a lot of weight and regularly uses strong medication to avoid constant pain. The person has no appetite, feels nauseated, and needs to spend most of the day in bed.	0.540	0.377	0.687
Mastectomy (cancer_mastectomy)	This person had one of her breasts removed and sometimes has pain or swelling in the arms.	0.036	0.020	0.057

Stoma (cancer_stoma)	This person has a pouch attached to an opening in the belly to collect and empty stools.	0.095	0.063	0.131
Laryngectomy (speech_problems)	This person has difficulty speaking, and others find it difficult to understand.	0.051	0.032	0.078
Urinary incontinence (incontinence)	This person cannot control urinating.	0.139	0.094	0.198
Impotence	This person has difficulty in obtaining or maintaining an erection.	0.017	0.009	0.030

Estimating non-melanoma skin cancer (squamous and basal cell carcinoma)

Mortality due to squamous cell skin cancer was estimated in the same way that all other cancers were estimated using the same methods as in GBD 2013. Cancer registry data were used as input data into the COD database. CODEm models were run to generate estimates for all countries, years, and age groups by sex.
We estimated squamous cell skin cancer incidence by using cancer registry as well as primary literature data for incidence. Only cancer registries that were listed in CI5 VIII as registering squamous cell carcinoma were included in the analysis.[26] For cancer registry data reported at the three-digit level (C44: Other and unspecified malignant neoplasm of skin), proportions from Karagas et al were used to split C44 into squamous cell carcinoma and basal cell carcinoma.[27] A systematic literature review was done to update any newly published articles between 2013 and 2015. The search was done on 8/15/2015 and yielded 619 initial results of which five were included as sources for incidence. DisMod-MR 2.1 was used to model incidence. To estimate prevalence the incidence estimates were combined with the yearly survival estimates up to 10 years. Relative survival from squamous cell skin cancer was estimated by scaling cancer specific survival between a “best case” survival of 100% and a “worst case” survival. For the “worst case” survival data for melanoma from the 1950 US Mortality Files was compared to Cancer Survival in Africa, Asia, the Caribbean, and Central America (SurvCan) data and whichever survival was the lowest was used.

As in GBD 2013 for basal cell carcinoma of the skin (BCC) we did not estimate any mortality given that this is a very rare event. Incidence estimates for BCC were generated using the same methods as for GBD 2013. Cancer registry data as well as literature were used for incidence data. A systematic literature review was done to update any newly published articles between 2013 and 2015. The search was done on 8/15/2015 and yielded 619 initial results, of which 14 were included as sources for incidence. DisMod-MR 2.1 was used to model incidence and prevalence. Since prevalence and duration of basal cell skin cancer are not generally reported, we calculated the prevalence of BCC as function of two extreme scenarios (duration 1 versus 5 years). Country, age, sex and year specific duration was estimated using a country-age-sex-year specific relative access-to-care-score.

The access to care score was based on the melanoma mortality to incidence ratio:

$$Access\ to\ cancer\ care = 1 - \frac{Age\ standardized\ MI\ ratio_{cys} - Age\ standardized\ MI\ ratio_{min}}{Age\ standardized\ MI\ ratio_{max} - Age\ standardized\ MI\ ratio_{min}}$$

c=country; y=year; s=sex; Age-standardized MI $ratio_{min}$=lowest MI ratio for all countries and years; Age-standardized MI $ratio_{max}$=highest MI ratio for all countries and years

Remission was calculated as the inverse of the duration estimates and used as additional input for DisMod-MR 2.1.

There are no other significant changes to the GBD 2015 neoplasms modelling process.

References

1 GBD Mortality and Causes of Death Collaborators. Global, regional, and national life expectancy, all-cause and cause specific mortality for 249 causes of death, 1980–2015: a systematic analysis for the Global Burden of Disease Study 2015. *Lancet* 2016; published online Aug.

2 National Cancer Institute (United States). United States SEER Cancer Data 1973-2010. Bethesda, United States: National Cancer Institute (United States). .

3 Sankaranarayanan R, Swaminathan R, World Health Organization, International Agency for Research on Cancer, editors. Cancer survival in Africa, Asia, the Caribbean and Central America. Lyon, France: International Agency for Research on Cancer, World Health Organization, 2011.

4 National Center for Health Statistics, Centers for Disease Control and, Prevention. US Mortality Files. 61-Year Trends in U.S. Cancer Death Rates. http://seer.cancer.gov/archive/csr/1975_2010/results_merged/topic_historical_mort_trends.pdf.

5 Canadian Institute for Health Information (CIHI). Canada Discharge Abstract Database 1994-2009. Ottawa, Canada: Canadian Institute for Health Information (CIHI). .

6 Ministry of Health (Mexico). Mexico Ministry of Health Hospital Discharges 2000-2012. Mexico City, México: Ministry of Health (Mexico). .

7 Fitzmaurice C, Dicker D, Pain A, *et al.* The Global Burden of Cancer 2013. *JAMA Oncol* 2015; published online May 28. DOI:10.1001/jamaoncol.2015.0735.

8 Neal RD, Din NU, Hamilton W, *et al.* Comparison of cancer diagnostic intervals before and after implementation of NICE guidelines: analysis of data from the UK General Practice Research Database. *Br J Cancer* 2014; **110**: 584–92.

9 Surveillance, Epidemiology, and End Results (SEER) Program (www.seer.cancer.gov) SEER*Stat Database: Incidence - SEER 18 Regs Research Data + Hurricane Katrina Impacted Louisiana Cases, Nov 2012 Sub (1973-2010 varying) - Linked To County Attributes - Total U.S., 1969-2011 Counties, National Cancer Institute, DCCPS, Surveillance Research Program, Surveillance Systems Branch, released April 2013, based on the November 2012 submission. .

10 Allgar VL, Neal RD. Delays in the diagnosis of six cancers: analysis of data from the National Survey of NHS Patients: Cancer. *Br J Cancer* 2005; **92**: 1959–70.

11 Neal RD, Cannings-John R, Hood K, *et al.* Excision of malignant melanomas in North Wales: effect of location and surgeon on time to diagnosis and quality of excision. *Fam Pract* 2008; **25**: 221–7.

12 Nolan RC, Chan MT-L, Heenan PJ. A clinicopathologic review of lethal nonmelanoma skin cancers in Western Australia. *J Am Acad Dermatol* 2005; **52**: 101–8.

13 Kewalramani T, Nimer SD, Zelenetz AD, *et al.* Progressive disease following autologous transplantation in patients with chemosensitive relapsed or primary refractory Hodgkin's disease or aggressive non-Hodgkin's lymphoma. *Bone Marrow Transplant* 2003; **32**: 673–9.

14 Esteban D, Tovar N, Jiménez R, *et al.* Patients with relapsed/refractory chronic lymphocytic leukaemia may benefit from inclusion in clinical trials irrespective of the therapy received: a case-control retrospective analsysis. *Blood Cancer J* 2015; **5**: e356.

15 Canova C, Giorato E, Roveron G, Turrini P, Zanotti R. Validation of a stoma-specific quality of life questionnaire in a sample of patients with colostomy or ileostomy. *Colorectal Dis Off J Assoc Coloproctology G B Irel* 2013; **15**: e692-698.

16 Caricato M, Ausania F, Ripetti V, Bartolozzi F, Campoli G, Coppola R. Retrospective analysis of long-term defunctioning stoma complications after colorectal surgery. *Colorectal Dis Off J Assoc Coloproctology G B Irel* 2007; **9**: 559–61.

17 Erwin-Toth P, Thompson SJ, Davis JS. Factors impacting the quality of life of people with an ostomy in North America: results from the Dialogue Study. *J Wound Ostomy Cont Nurs Off Publ Wound Ostomy Cont Nurses Soc WOCN* 2012; **39**: 417-422-424.

18 Catalona WJ, Carvalhal GF, Mager DE, Smith DS. Potency, continence and complication rates in 1,870 consecutive radical retropubic prostatectomies. *J Urol* 1999; **162**: 433–8.

19 Donnellan SM, Duncan HJ, MacGregor RJ, Russell JM. Prospective assessment of incontinence after radical retropubic prostatectomy: objective and subjective analysis. *Urology* 1997; **49**: 225–30.

20 Eastham JA, Kattan MW, Rogers E, *et al.* Risk factors for urinary incontinence after radical prostatectomy. *J Urol* 1996; **156**: 1707–13.

21 Kundu SD, Roehl KA, Eggener SE, Antenor JAV, Han M, Catalona WJ. Potency, continence and complications in 3,477 consecutive radical retropubic prostatectomies. *J Urol* 2004; **172**: 2227–31.

22 Potosky AL, Davis WW, Hoffman RM, *et al.* Five-Year Outcomes After Prostatectomy or Radiotherapy for Prostate Cancer: The Prostate Cancer Outcomes Study. *JNCI J Natl Cancer Inst* 2004; **96**: 1358–67.

23 Sacco E, Prayer-Galetti T, Pinto F, *et al.* Urinary incontinence after radical prostatectomy: incidence by definition, risk factors and temporal trend in a large series with a long-term follow-up. *BJU Int* 2006; **97**: 1234–41.

24 Stanford JL, Feng Z, Hamilton AS, *et al.* Urinary and sexual function after radical prostatectomy for clinically localized prostate cancer: the Prostate Cancer Outcomes Study. *JAMA* 2000; **283**: 354–60.

25 Walsh PC, Marschke P, Ricker D, Burnett AL. Patient-reported urinary continence and sexual function after anatomic radical prostatectomy. *Urology* 2000; **55**: 58–61.

26 Parkin D, Whelan S, Ferlay J, Teppo L, Thomas D. Cancer Incidence in Five Continents VIII. Lyon: IARC, 2002.

27 Karagas MR, Greenberg ER, Spencer SK, Stukel TA, Mott LA. Increase in incidence rates of basal cell and squamous cell skin cancer in New Hampshire, USA. New Hampshire Skin Cancer Study Group. *Int J Cancer J Int Cancer* 1999; **81**: 555–9.

Fatal Other Neurological Disorders estimation process

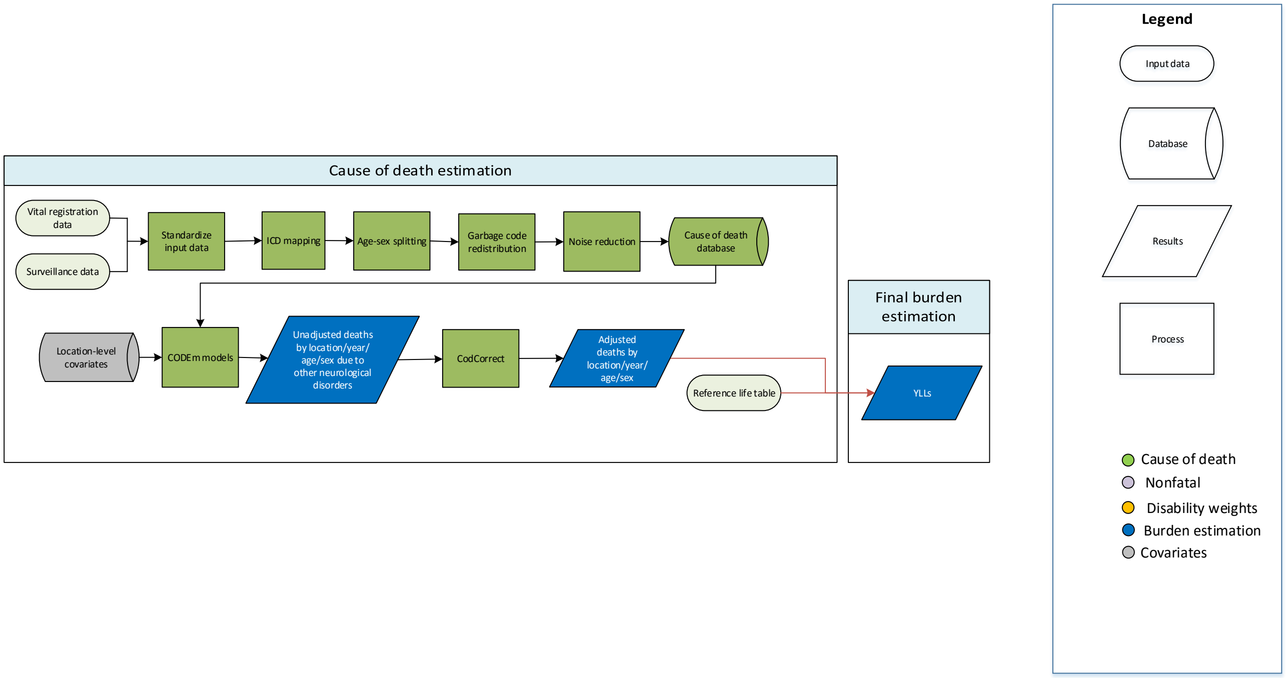

Input data

Data used to estimate other neurological disorders included vital registration and surveillance data from the cause of death (COD) database. Our outlier criteria excluded data points that (1) were implausibly high or low, (2) substantially conflicted with established age or temporal patterns, or (3) significantly conflicted with other data sources conducted from the same locations or locations with similar characteristics (ie, Socio-demographic Index).

Relative to GBD 2013, the main data-related change to other neurological disorders is the removal of deaths due to motor neuron disease (MND) – a function of MND becoming a separate cause. This resulted in a reduction of our mortality estimates for other neurological disorders.

Modelling strategy

The standard CODEm modelling approach was applied to estimate deaths due to other neurological disorders. Male and female CODEm models were run for deaths occurring between ages 28 days to 80+ years.

Although the covariate list remains essentially unchanged from GBD 2013, a number of covariates have received substantial updates (all received some updates). Metabolic values (eg, BMI), smoking-based covariates, health systems access, and meat-based dietary energy consumption received the greatest overhaul of the covariates informing this model. The Socio-demographic Index (SDI) covariate was added to the GBD 2015 covariate set.

Non-fatal Other Neurological Disorders estimation process

In addition to the neurological disorders described above, there are many diverse types of neurological disorders with a range of severities and associated sequelae. Because these neurological disorders are diverse in their underlying causes and risk factors as well as in their associated health outcomes, modelling them together in a DisMod-MR model would not produce reliable estimates of prevalence or excess mortality. Instead, we calculated the YLDs caused by neurological disorders directly using a YLD/YLL ratio.

We calculated the ratio of YLDs to YLLs across the specified neurological disorders for which nonfatal outcomes were modelled, using YLL estimates from the GBD 2015 cause of death (CoD) analysis. We then multiplied this YLD/YLL ratio by the YLL estimates for other neurological disorders from the GBD 2015 CoD analysis, providing us with an estimate of the YLDs associated with other neurological disorders.

GATHER checklist of information that should be included in reports of global health estimates, with description of compliance and location of information for GBD 2015 paper on Neurological disorders

#	GATHER checklist item	Description of compliance	Reference
**Objectives and funding**			
1	Define the indicators, populations, and time periods for which estimates were made.	Narrative provided in paper and appendix describing indicators, definitions, and populations.	Manuscript; Methods Appendix, Section 1. GBD Overview
2	List the funding sources for the work.	Funding sources listed in paper.	Funding of GBD by Bill & Melinda Gates Foundation acknowledged
**Data Inputs**			
For all data inputs from multiple sources that are synthesized as part of the study:			
3	Describe how the data were identified and how the data were accessed.	Narrative description of data seeking methodology provided.	• Main text, Methods<br>• Appendix, description of methods of estimating fatal and non-fatal outcomes of neurological disorders
4	Specify the inclusion and exclusion criteria. Identify all ad-hoc exclusions.	Narrative about inclusion and exclusion criteria provided.	• Main text, Methods<br>• Appendix, description of methods of estimating fatal and non-fatal outcomes of neurological disorders
5	Provide information on all included data sources and their main characteristics. For each data source used, report reference information or contact name/institution, population represented, data collection method, year(s) of data collection, sex and age range, diagnostic criteria or measurement method, and sample size, as relevant.	Interactive, online data source tool that provides metadata for data sources by component, geography, cause, risk, or impairment has been developed.	http://ghdx.healthdata.org/gbd-2015/data-input-sources

6	Identify and describe any categories of input data that have potentially important biases (e.g., based on characteristics listed in item 5).	Summary of known biases by cause included in appendix.	• Main text, Methods and Discussion<br>• Appendix, description of methods of estimating fatal and non-fatal outcomes of neurological disorders
For data inputs that contribute to the analysis but were not synthesized as part of the study:			
7	Describe and give sources for any other data inputs.	Included in online data source tool.	http://ghdx.healthdata.org/gbd-2015/data-input-sources
For all data inputs:			
8	Provide all data inputs in a file format from which data can be efficiently extracted (e.g., a spreadsheet as opposed to a PDF), including all relevant meta-data listed in item 5. For any data inputs that cannot be shared due to ethical or legal reasons, such as third-party ownership, provide a contact name or the name of the institution that retains the right to the data.	Downloads of input data available through online tools, including data visualization tools and data query tools. Input data not available in tools will be made available upon request.	Online data tools<br>http://ghdx.healthdata.org/gbd-2015/data-input-sources<br>http://www.healthdata.org/results/data-visualizations;<br>http://ghdx.healthdata.org/;<br>http://ghdx.healthdata.org/gbd-data-tool
**Data analysis**			
9	Provide a conceptual overview of the data analysis method. A diagram may be helpful.	Flow diagrams and narrative of methodological processes have been provided.	• Main text, methods<br>• Appendix description of methods of estimating fatal and non-fatal outcomes of neurological disorders
10	Provide a detailed description of all steps of the analysis, including mathematical formulae. This description should cover, as relevant, data cleaning, data pre-processing, data adjustments and weighting of data sources, and mathematical or statistical model(s).	Flow diagrams and narrative of methodological processes have been provided.	• Main text, methods<br>• Appendix description of methods of estimating fatal and non-fatal outcomes of neurological disorders

11	Describe how candidate models were evaluated and how the final model(s) were selected.	Provided in the methodological write-ups.	• Appendix description of methods of estimating fatal and non-fatal outcomes of neurological disorders
12	Provide the results of an evaluation of model performance, if done, as well as the results of any relevant sensitivity analysis.	Provided in the methodological write-ups.	Appendix description of methods of estimating fatal and non-fatal outcomes of neurological disorders
13	Describe methods for calculating uncertainty of the estimates. State which sources of uncertainty were, and were not, accounted for in the uncertainty analysis.	Provided in the methodological write-ups.	• Main text, methods<br>• Appendix description of methods of estimating fatal and non-fatal outcomes of neurological disorders
14	State how analytic or statistical source code used to generate estimates can be accessed.	Access statement provided.	http://ghdx.healthdata.org/gbd-2015-code
**Results and Discussion**			
15	Provide published estimates in a file format from which data can be efficiently extracted.	GBD 2015 results are available through online data visualization tools, the Global Health Data Exchange, and the online data query tool	Online data tools http://www.healthdata.org/results/data-visualizations; http://ghdx.healthdata.org/; http://ghdx.healthdata.org/gbd-data-tool
16	Report a quantitative measure of the uncertainty of the estimates (e.g. uncertainty intervals).	Uncertainty intervals are provided with all results.	Main text and online data tools (to go live with GBD 2015 at publication) http://www.healthdata.org/results/data-visualizations; http://ghdx.healthdata.org/; http://ghdx.healthdata.org/gbd-data-tool

17	Interpret results in light of existing evidence. If updating a previous set of estimates, describe the reasons for changes in estimates.	Discussion of methodological changes between GBD rounds provided in the narrative of the paper.	Main text, Discussion
18	Discuss limitations of the estimates. Include a discussion of any modelling assumptions or data limitations that affect interpretation of the estimates.	Discussion of limitations provided in the narrative of the main paper as well as in the methodological write-ups in the appendix.	• Main text, Discussion<br>• Appendix description of methods of estimating fatal and non-fatal outcomes of neurological disorders

Appendix Table 1: List of International Classification of Diseases (ICD) codes mapped to the Global Burden of Disease cause list for causes of death

**Cause**	**ICD10**	**ICD9**
Meningitis	A39-A39.4, A39.8-A39.9, A87-A87.9, D86.81, G00.0-G00.8, G03-G03.8	036-036.40, 036.5, 036.8-036.9, 047-049.9, 320.0-320.89, 321-322.9
Pneumococcal meningitis	G00.1	320.1
H. influenzae type B meningitis	G00.0	320.0
Meningococcal meningitis	A39-A39.4, A39.8-A39.9	036-036.40, 036.5, 036.8-036.9
Other meningitis	A87-A87.9, D86.81, G00.2-G00.8, G03-G03.8	047-049.9, 320.2-320.89, 321-322.9
Encephalitis	A83-A86.4, B94.1, F07.1, G04-G05.8	062-064.9, 139.0, 323, 323.4-323.9
Tetanus	A33-A35.0	037-037.9, 771.3
Brain and nervous system cancer	C70-C72.9	191-192.9
Cerebrovascular disease	G45-G46.8, I60-I61.9, I62.0-I62.03, I63-I63.9, I65-I66.9, I67.0-I67.3, I67.5-I67.6, I68.1-I68.2, I69.0-I69.398	430-435.9, 437.0-437.2, 437.5-437.8
Ischaemic stroke	G45-G46.8, I63-I63.9, I65-I66.9, I67.2-I67.3, I67.5-I67.6, I69.3-I69.398	433-435.9, 437.0-437.1, 437.5-437.8
Haemorrhagic stroke	I60-I61.9, I62.0-I62.03, I67.0-I67.1, I68.1-I68.2, I69.0-I69.298	430-432.9, 437.2

Alzheimer's disease and other dementias	F00-F03.91, G30-G31.1, G31.8-G31.9	290-290.9, 294.1-294.9, 331-331.2, 331.6-331.7, 331.8-331.9
Parkinson's disease	G20-G21.0, G21.2-G22.0	332-332.9
Epilepsy	G40-G41.9	345-345.91
Multiple sclerosis	G35-G35.9	340-340.9
Motor neuron disease	G12.2-G12.9	335-335.29, 335.8-335.9
Other neurological disorders*	G10-G12.1, G13-G13.8, G23-G24, G24.1-G25.0, G25.2-G25.3, G25.5, G25.8-G26.0, G36-G37.9, G61-G61.9, G70-G72, G72.2-G73.7, G90-G90.9, G95-G95.9, M33-M33.99	330-330.9, 331.3-331.5, 333-334.9, 335.3, 336-337.9, 341-341.9, 348-356, 358-359, 710.3

***List of other neurological disorders in ICD 10 and ICD 9:**

**ICD 10**	**Neurological disorder**
G10	Huntington's disease
G11	Hereditary ataxia
G12	Spinal muscular atrophy and related syndromes
G13	Systemic atrophies primarily affecting central nervous system in diseases classified elsewhere
G23	Other degenerative diseases of basal ganglia
G24	Dystonia
G25	Other extrapyramidal and movement disorders
G26	Extrapyramidal and movement disorders in diseases classified elsewhere

G36	Other acute disseminated demyelination
G37	Other demyelinating diseases of central nervous system
G61	Inflammatory polyneuropathy
G70	Myasthenia gravis and other myoneural disorders
G71	Primary disorders of muscles (including muscular dystrophy and myopathies)
G72	Other and unspecified myopathies
G73	Disorders of myoneural junction and muscle in diseases classified elsewhere
G90	Disorders of autonomic nervous system
G95	Other and unspecified diseases of spinal cord
M33	Systemic connective tissue disorders

**ICD 9**	**Neurological disorder**
330	Cerebral degenerations usually manifest in childhood
331.3-5	Hydrocephalus
333	Other extrapyramidal disease and abnormal movement disorders
334	Spinocerebellar disease
335	Anterior horn disease (not 335.2 motor-neuron disease)
336	Other diseases of spinal cord

337	Disorders of autonomic nervous system
341	Other demyelinating diseases of central nervous system
348	Other conditions of the brain
349	Other and unspecified disorders of the nervous system
350	Trigeminal nerve disorders
351	Facial nerve disorders
352	Disorders of other cranial nerves
353	Nerve root and plexus disorders
354	Mononeuritis of upper limb and mononeuritis multiplex
355	Mononeuritis of lower limb and unspecified site
356	Hereditary and idiopathic peripheral neuropathy
358	Myoneural disorders
359	Muscular dystrophies and other myopathies
710.3	Dermatomyositis

*****Table modified from GBD 2015 Mortality and Causes of Death Collaborators. Global, regional, and national life expectancy, all-cause mortality, and cause-specific mortality for 249 causes of death, 1980-2015: a systematic analysis for the Global Burden of Disease Study 2015. *Lancet* 2016; 388: 1459–544

Appendix Table 2. GBD 2015 sequelae, health states, health state lay descriptions, and disability weights for neurological disorders

Sequela	Health state	Lay description	Disability weight
Acute meningitis	Infectious disease, acute episode, severe	has a high fever and pain, and feels very weak, which causes great difficulty with daily activities.	0.133 (0.088–0.19)
Mild behavioural problems due to meningitis	Attention deficit hyperactivity disorder	is hyperactive and has difficulty concentrating, remembering things, and completing tasks.	0.045 (0.028–0.066)
Mild motor impairment due to long term due to meningitis	Motor impairment, mild	has some difficulty in moving around but is able to walk without help.	0.01 (0.005–0.019)
Mild motor plus cognitive impairments due to meningitis	Motor plus cognitive impairments, mild	has some difficulty in moving around but is able to walk without help. The person is slow in learning at school. As an adult, the person has some difficulty doing complex or unfamiliar tasks but otherwise functions independently.	0.031 (0.018–0.05)
Borderline intellectual disability due to meningitis	Borderline intellectual functioning	is slow in learning at school. As an adult, the person has some difficulty doing complex or unfamiliar tasks but otherwise functions independently.	0.011 (0.005–0.02)
Monocular distance vision loss due to meningitis	Distance vision, monocular	is blind in one eye and has difficulty judging distances	0.017 (0.009-0.029)
Mild intellectual disability due to meningitis	Intellectual disability/mental retardation, mild	has low intelligence and is slow in learning at school. As an adult, the person can live independently, but often needs help to raise children and can only work at simple supervised jobs.	0.043 (0.026–0.064)
Moderate motor impairment due to meningitis	Motor impairment, moderate	has some difficulty in moving around, and difficulty in lifting and holding objects, dressing, and sitting upright, but is able to walk without help.	0.061 (0.04–0.089)

Sequela	Health state	Lay description	Disability weight
Severe motor impairment due to meningitis	Motor impairment, severe	is unable to move around without help, and is not able to lift or hold objects, get dressed, or sit upright.	0.402 (0.268–0.545)
Moderate motor plus cognitive impairments due to meningitis	Motor plus cognitive impairments, moderate	has some difficulty in moving around, holding objects, dressing and sitting upright, but can walk without help. The person has low intelligence and is slow in learning to speak and to do simple tasks.	0.203 (0.134–0.29)
Severe motor plus cognitive impairments due to meningitis	Motor plus cognitive impairments, severe	cannot move around without help, and cannot lift or hold objects, get dressed, or sit upright. The person also has very low intelligence, speaks few words, and needs constant supervision and help with all daily activities.	0.542 (0.374–0.702)
Epilepsy due to meningitis	Epilepsy	(combined DW)	--
Blindness due to meningitis	Distance vision blindness	is completely blind, which causes great difficulty in some daily activities, worry and anxiety, and great difficulty going outside the home without assistance.	0.187 (0.124–0.26)
Mild hearing loss due to meningitis	Hearing loss, mild	has great difficulty hearing and understanding another person talking in a noisy place (for example, on an urban street).	0.01 (0.004-0.019)
Mild hearing loss with ringing due to meningitis	Hearing loss, mild, with ringing	has great difficulty hearing and understanding another person talking in a noisy place (for example, on an urban street), and sometimes has annoying ringing in the ears.	0.021 (0.012–0.036)
Moderate hearing loss due to meningitis	Hearing loss, moderate	is unable to hear and understand another person talking in a noisy place (for example, on an urban street), and has difficulty hearing another person talking even in a quiet place or on the phone.	0.027 (0.015–0.042)

Sequela	Health state	Lay description	Disability weight
Moderate hearing loss with ringing due to meningitis	Hearing loss, moderate, with ringing	is unable to hear and understand another person talking in a noisy place (for example, on an urban street), has difficulty hearing another person talking even in a quiet place or on the phone, and has annoying ringing in the ears for 5 minutes at a time, almost every day.	0.074 (0.049–0.107)
Moderately severe hearing loss due to meningitis	Hearing loss, moderately severe	(custom DW: average of moderate and severe hearing loss impairment)	--
Moderately severe hearing loss with ringing due to meningitis	Hearing loss, moderately severe, with ringing	(custom DW: average of moderate and severe hearing loss impairment)	--
Severe hearing loss due to meningitis	Hearing loss, severe	is unable to hear and understand another person talking, even in a quiet place, and unable to take part in a phone conversation. Difficulties with communicating and relating to others cause emotional impact at times (for example worry or depression).	0.158 (0.105–0.227)
Severe hearing loss with ringing due to meningitis	Hearing loss, severe, with ringing	is unable to hear and understand another person talking, even in a quiet place, is unable to take part in a phone conversation, and has annoying ringing in the ears for more than 5 minutes at a time, almost every day. Difficulties with communicating and relating to others cause emotional impact at times (for example worry or depression).	0.261 (0.175–0.36)
Profound hearing loss due to meningitis	Hearing loss, profound	is unable to hear and understand another person talking, even in a quiet place, is unable to take part in a phone conversation, and has great difficulty hearing anything in any other situation. Difficulties with communicating and relating to others often cause worry, depression or loneliness.	0.204 (0.134–0.288)

Sequela	Health state	Lay description	Disability weight
Profound hearing loss with ringing due to meningitis	Hearing loss, profound, with ringing	is unable to hear and understand another person talking, even in a quiet place, is unable to take part in a phone conversation, has great difficulty hearing anything in any other situation, and has annoying ringing in the ears for more than 5 minutes at a time, several times a day. Difficulties with communicating and relating to others often cause worry, depression, or loneliness.	0.277 (0.182–0.387)
Complete hearing loss due to meningitis	Hearing loss, complete	cannot hear at all in any situation, including even the loudest sounds, and cannot communicate verbally or use a phone. Difficulties with communicating and relating to others often cause worry, depression or loneliness.	0.215 (0.144–0.307)
Complete hearing loss with ringing due to meningitis	Hearing loss, complete, with ringing	cannot hear at all in any situation, including even the loudest sounds, and cannot communicate verbally or use a phone, and has very annoying ringing in the ears for more than half of the day. Difficulties with communicating and relating to others often cause worry, depression or loneliness.	0.316 (0.212–0.435)
Moderate vision impairment due to meningitis	Distance vision, moderate impairment	has vision problems that make it difficult to recognise faces or objects across a room.	0.031 (0.019–0.049)
Severe vision impairment due to meningitis	Distance vision, severe impairment	has severe vision loss, which causes difficulty in daily activities, some emotional impact (for example worry), and some difficulty going outside the home without assistance.	0.184 (0.125–0.258)
Acute encephalitis	Infectious disease, acute episode, severe	has a high fever and pain, and feels very weak, which causes great difficulty with daily activities.	0.133 (0.088–0.19)

Sequela	Health state	Lay description	Disability weight
Mild behavioural problems due to encephalitis	Attention deficit hyperactivity disorder	is hyperactive and has difficulty concentrating, remembering things, and completing tasks.	0.045 (0.028–0.066)
Mild motor impairment due to long term due to encephalitis	Motor impairment, mild	has some difficulty in moving around but is able to walk without help.	0.01 (0.005–0.019)
Mild motor plus cognitive impairments due to encephalitis	Motor plus cognitive impairments, mild	has some difficulty in moving around but is able to walk without help. The person is slow in learning at school. As an adult, the person has some difficulty doing complex or unfamiliar tasks but otherwise functions independently.	0.031 (0.018–0.05)
Borderline intellectual disability due to encephalitis	Borderline intellectual functioning	is slow in learning at school. As an adult, the person has some difficulty doing complex or unfamiliar tasks but otherwise functions independently.	0.011 (0.005–0.02)
Monocular distance vision loss due to encephalitis	Distance vision, monocular	is blind in one eye and has difficulty judging distances	0.017 (0.009–0.029)
Mild intellectual disability due to encephalitis	Intellectual disability/mental retardation, mild	has low intelligence and is slow in learning at school. As an adult, the person can live independently, but often needs help to raise children and can only work at simple supervised jobs.	0.043 (0.026–0.064)
Moderate motor impairment due to encephalitis	Motor impairment, moderate	has some difficulty in moving around, and difficulty in lifting and holding objects, dressing and sitting upright, but is able to walk without help.	0.061 (0.04–0.089)
Severe motor impairment due to encephalitis	Motor impairment, severe	is unable to move around without help, and is not able to lift or hold objects, get dressed or sit upright.	0.402 (0.268–0.545)
Moderate motor plus cognitive impairments due to encephalitis	Motor plus cognitive impairments, moderate	has some difficulty in moving around, holding objects, dressing and sitting upright, but can walk without help.	0.203 (0.134–0.29)

Sequela	Health state	Lay description	Disability weight
		The person has low intelligence and is slow in learning to speak and to do simple tasks.	
Severe motor plus cognitive impairments due to encephalitis	Motor plus cognitive impairments, severe	cannot move around without help, and cannot lift or hold objects, get dressed or sit upright. The person also has very low intelligence, speaks few words, and needs constant supervision and help with all daily activities.	0.542 (0.374–0.702)
Epilepsy due to encephalitis	Epilepsy	(combined DW)	--
Blindness due to encephalitis	Distance vision blindness	is completely blind, which causes great difficulty in some daily activities, worry and anxiety, and great difficulty going outside the home without assistance.	0.187 (0.124–0.26)
Moderate vision impairment due to encephalitis	Distance vision, moderate impairment	has vision problems that make it difficult to recognise faces or objects across a room.	0.031 (0.019–0.049)
Severe vision impairment due to encephalitis	Distance vision, severe impairment	has severe vision loss, which causes difficulty in daily activities, some emotional impact (for example worry), and some difficulty going outside the home without assistance.	0.184 (0.125–0.258)
Severe tetanus	Infectious disease, acute episode, severe	has a high fever and pain, and feels very weak, which causes great difficulty with daily activities.	0.133 (0.088–0.19)
Mild motor impairment due to neonatal tetanus	Motor impairment, mild	has some difficulty in moving around but is able to walk without help.	0.01 (0.005–0.019)
Mild motor plus cognitive impairments due to neonatal tetanus	Motor plus cognitive impairments, mild	has some difficulty in moving around but is able to walk without help. The person is slow in learning at school. As an adult, the person has some difficulty doing	0.031 (0.018–0.05)

Sequela	Health state	Lay description	Disability weight
		complex or unfamiliar tasks but otherwise functions independently.	
Moderate motor impairment due to neonatal tetanus	Motor impairment, moderate	has some difficulty in moving around, and difficulty in lifting and holding objects, dressing and sitting upright, but is able to walk without help.	0.061 (0.04–0.089)
Moderate motor impairment with blindness due to neonatal tetanus	Moderate motor impairment with blindness	(combined DW)	--
Moderate motor impairment with epilepsy due to neonatal tetanus	Moderate motor impairment with epilepsy	(combined DW)	--
Moderate motor impairment with blindness and epilepsy due to neonatal tetanus	Moderate motor impairment with blindness and epilepsy	(combined DW)	--
Moderate motor plus cognitive impairment with blindness due to neonatal tetanus	Moderate motor plus cognitive impairment with blindness	(combined DW)	--
Moderate motor plus cognitive impairment with epilepsy due to neonatal tetanus	Moderate motor plus cognitive impairment with epilepsy	(combined DW)	--
Moderate motor plus cognitive impairment with blindness and epilepsy due to neonatal tetanus	Moderate motor plus cognitive impairment with blindness and epilepsy	(combined DW)	--
Severe motor impairment due to neonatal tetanus	Motor impairment, severe	is unable to move around without help, and is not able to lift or hold objects, get dressed, or sit upright.	0.402 (0.268–0.545)
Severe motor impairment with blindness due to neonatal tetanus	Severe motor impairment with blindness	(combined DW)	--

Sequela	Health state	Lay description	Disability weight
Severe motor impairment with epilepsy due to neonatal tetanus	Severe motor impairment with epilepsy	(combined DW)	--
Severe motor impairment with blindness and epilepsy due to neonatal tetanus	Severe motor impairment with blindness and epilepsy	(combined DW)	--
Severe motor plus cognitive impairment with blindness due to neonatal tetanus	Severe motor plus cognitive impairment with blindness	(combined DW)	--
Severe motor plus cognitive impairment with epilepsy due to neonatal tetanus	Severe motor plus cognitive impairment with epilepsy	(combined DW)	--
Severe motor plus cognitive impairment with blindness and epilepsy due to neonatal tetanus	Severe motor plus cognitive impairment with blindness and epilepsy	(combined DW)	--
Diagnosis and primary therapy phase of brain and nervous system cancers	Cancer, diagnosis and primary therapy	has pain, nausea, fatigue, weight loss, and high anxiety.	0.288 (0.193–0.399)
Controlled phase of brain and nervous system cancers	Generic uncomplicated disease: worry and daily medication	has a chronic disease that requires medication every day and causes some worry but minimal interference with daily activities.	0.049 (0.031–0.072)
Metastatic phase of brain and nervous system cancers	Cancer, metastatic	has severe pain, extreme fatigue, weight loss, and high anxiety.	0.451 (0.307–0.6)
Terminal phase of brain and nervous system cancers	Terminal phase, with medication (for cancers, end-stage kidney/liver disease)	has lost a lot of weight and regularly uses strong medication to avoid constant pain. The person has no appetite, feels nauseated, and needs to spend most of the day in bed.	0.54 (0.377–0.687)

Sequela	Health state	Lay description	Disability weight
Chronic ischaemic stroke severity level 1	Stroke, long-term consequences, mild	has some difficulty in moving around and some weakness in one hand, but is able to walk without help.	0.019 (0.01–0.032)
Chronic ischaemic stroke severity level 2	Stroke, long-term consequences, moderate	has some difficulty in moving around, and in using the hands for lifting and holding things, dressing and grooming.	0.07 (0.046–0.099)
Chronic ischaemic stroke severity level 3	Stroke, long-term consequences, moderate plus cognition problems	has some difficulty in moving around, in using the hands for lifting and holding things, dressing and grooming, and in speaking. The person is often forgetful and confused.	0.316 (0.206–0.437)
Chronic ischaemic stroke severity level 4	Stroke, long-term consequences, severe	is confined to bed or a wheelchair, has difficulty speaking and depends on others for feeding, toileting, and dressing.	0.552 (0.377–0.707)
Chronic ischaemic stroke severity level 5	Stroke, long-term consequences, severe plus cognition problems	is confined to bed or a wheelchair, depends on others for feeding, toileting and dressing, and has difficulty speaking, thinking clearly and remembering things.	0.588 (0.411–0.744)
Acute ischaemic stroke severity level 1	Stroke, long-term consequences, mild	has some difficulty in moving around and some weakness in one hand, but is able to walk without help.	0.019 (0.01–0.032)
Acute ischaemic stroke severity level 2	Stroke, long-term consequences, moderate	has some difficulty in moving around, and in using the hands for lifting and holding things, dressing, and grooming.	0.07 (0.046–0.099)
Acute ischaemic stroke severity level 3	Stroke, long-term consequences, moderate plus cognition problems	has some difficulty in moving around, in using the hands for lifting and holding things, dressing and grooming, and in speaking. The person is often forgetful and confused.	0.316 (0.206–0.437)

Sequela	Health state	Lay description	Disability weight
Acute ischaemic stroke severity level 4	Stroke, long-term consequences, severe	is confined to bed or a wheelchair, has difficulty speaking and depends on others for feeding, toileting and dressing.	0.552 (0.377–0.707)
Acute ischaemic stroke severity level 5	Stroke, long-term consequences, severe plus cognition problems	is confined to bed or a wheelchair, depends on others for feeding, toileting and dressing, and has difficulty speaking, thinking clearly and remembering things.	0.588 (0.411–0.744)
Asymptomatic chronic haemorrhagic stroke	--	--	--
Chronic haemorrhagic stroke severity level 1	Stroke, long-term consequences, mild	has some difficulty in moving around and some weakness in one hand, but is able to walk without help.	0.019 (0.01–0.032)
Chronic haemorrhagic stroke severity level 2	Stroke, long-term consequences, moderate	has some difficulty in moving around, and in using the hands for lifting and holding things, dressing and grooming.	0.07 (0.046–0.099)
Chronic haemorrhagic stroke severity level 3	Stroke, long-term consequences, moderate plus cognition problems	has some difficulty in moving around, in using the hands for lifting and holding things, dressing and grooming, and in speaking. The person is often forgetful and confused.	0.316 (0.206–0.437)
Chronic haemorrhagic stroke severity level 4	Stroke, long-term consequences, severe	is confined to bed or a wheelchair, has difficulty speaking and depends on others for feeding, toileting and dressing.	0.552 (0.377–0.707)
Chronic haemorrhagic stroke severity level 5	Stroke, long-term consequences, severe plus cognition problems	is confined to bed or a wheelchair, depends on others for feeding, toileting and dressing, and has difficulty speaking, thinking clearly and remembering things.	0.588 (0.411–0.744)
Acute haemorrhagic stroke severity level 1	Stroke, long-term consequences, mild	has some difficulty in moving around and some weakness in one hand, but is able to walk without help.	0.019 (0.01–0.032)

**Sequela**	**Health state**	**Lay description**	**Disability weight**
Acute haemorrhagic stroke severity level 2	Stroke, long-term consequences, moderate	has some difficulty in moving around, and in using the hands for lifting and holding things, dressing and grooming.	0.07 (0.046–0.099)
Acute haemorrhagic stroke severity level 3	Stroke, long-term consequences, moderate plus cognition problems	has some difficulty in moving around, in using the hands for lifting and holding things, dressing and grooming, and in speaking. The person is often forgetful and confused.	0.316 (0.206–0.437)
Acute haemorrhagic stroke severity level 4	Stroke, long-term consequences, severe	is confined to bed or a wheelchair, has difficulty speaking and depends on others for feeding, toileting and dressing.	0.552 (0.377–0.707)
Acute haemorrhagic stroke severity level 5	Stroke, long-term consequences, severe plus cognition problems	is confined to bed or a wheelchair, depends on others for feeding, toileting and dressing, and has difficulty speaking, thinking clearly and remembering things.	0.588 (0.411–0.744)
Mild Alzheimer’s disease and other dementias	Dementia, mild	has some trouble remembering recent events, and finds it hard to concentrate and make decisions and plans.	0.069 (0.046–0.099)
Moderate Alzheimer’s disease and other dementias	Dementia, moderate	has memory problems and confusion, feels disoriented, at times hears voices that are not real, and needs help with some daily activities.	0.377 (0.252–0.508)
Severe Alzheimer’s disease and other dementias	Dementia, severe	has complete memory loss; no longer recognises close family members; and requires help with all daily activities.	0.449 (0.304–0.595)
Mild Parkinson’s disease	Parkinson’s disease, mild	has mild tremors and moves a little slowly, but is able to walk and do daily activities without assistance.	0.01 (0.005–0.019)
Moderate Parkinson’s disease	Parkinson’s disease, moderate	has moderate tremors and moves slowly, which causes some difficulty in walking and daily activities.	0.267 (0.181–0.372)

Sequela	Health state	Lay description	Disability weight
		The person has some trouble swallowing, talking, sleeping, and remembering things.	
Severe Parkinson’s disease	Parkinson’s disease, severe	has severe tremors and moves very slowly, which causes great difficulty in walking and daily activities. The person falls easily and has a lot of difficulty talking, swallowing, sleeping, and remembering things.	0.575 (0.396–0.73)
Seizure-free, treated epilepsy	Generic uncomplicated disease: worry and daily medication	has a chronic disease that requires medication every day and causes some worry but minimal interference with daily activities.	0.049 (0.031–0.072)
Less severe epilepsy	Epilepsy, less severe (seizures < once per month)	has sudden seizures two to five times a year, with violent muscle contractions and stiffness, loss of consciousness, and loss of urine or bowel control.	0.263 (0.173–0.367)
Severe epilepsy	Epilepsy, severe (seizures >= once per month)	has sudden seizures one or more times each month, with violent muscle contractions and stiffness, loss of consciousness, and loss of urine or bowel control. Between seizures the person has memory loss and difficulty concentrating.	0.552 (0.375–0.71)
Mild multiple sclerosis	Multiple sclerosis, mild	has mild loss of feeling in one hand, is a little unsteady while walking, has slight loss of vision in one eye, and often needs to urinate urgently.	0.183 (0.124–0.253)
Moderate multiple sclerosis	Multiple sclerosis, moderate	needs help walking, has difficulty with writing and arm coordination, has loss of vision in one eye and cannot control urinating.	0.463 (0.313–0.613)
Severe multiple sclerosis	Multiple sclerosis, severe	has slurred speech and difficulty swallowing. The person has weak arms and hands, very limited and stiff	0.719 (0.534–0.858)

Sequela	Health state	Lay description	Disability weight
		leg movement, has loss of vision in both eyes and cannot control urinating.	
Asymptomatic, but worry about diagnosis of motor neuron disease	Generic uncomplicated disease: anxiety about diagnosis	has a disease diagnosis that causes some worry but minimal interference with daily activities.	0.012 (0.006–0.023)
Mild motor impairment and mild respiratory problems due to motor neuron disease	Mild motor impairment and mild COPD	(combined DW)	--
Mild motor impairment and severe respiratory problems due to motor neuron disease	Mild motor impairment and severe COPD	(combined DW)	--
Mild motor impairment and speech problems due to motor neuron disease	Mild motor impairment and speech problems	(combined DW)	--
Mild motor impairment due to motor neuron disease	Motor impairment, mild	has some difficulty in moving around but is able to walk without help.	0.01 (0.005–0.019)
Mild motor impairment, mild respiratory problems, and speech problems due to motor neuron disease	Mild motor impairment, mild COPD and speech problems	(combined DW)	--
Mild motor impairment, moderate respiratory problems, and speech problems due to motor neuron disease	Mild motor impairment, moderate COPD and speech problems	(combined DW)	--

Sequela	Health state	Lay description	Disability weight
Mild motor impairment, severe respiratory problems and speech problems due to motor neuron disease	Mild motor impairment, severe COPD and speech problems	(combined DW)	--
Mild respiratory problems and speech problems due to motor neuron disease	Mild COPD and speech problems	(combined DW)	--
Mild respiratory problems due to motor neuron disease	COPD and other chronic respiratory problems, mild	has cough and shortness of breath after heavy physical activity, but is able to walk long distances and climb stairs.	0.019 (0.011–0.033)
Moderate motor impairment and mild respiratory problems due to motor neuron disease	Moderate motor impairment and mild COPD	(combined DW)	--
Moderate motor impairment and moderate respiratory problems due to motor neuron disease	Moderate motor impairment and moderate COPD	(combined DW)	--
Moderate motor impairment and severe respiratory problems due to motor neuron disease	Moderate motor impairment and severe COPD	(combined DW)	--
Moderate motor impairment and speech problems due to motor neuron disease	Moderate motor impairment and speech problems	(combined DW)	--
Moderate motor impairment due to motor neuron disease	Motor impairment, moderate	has some difficulty in moving around, and difficulty in lifting and holding objects, dressing, and sitting upright, but is able to walk without help.	0.061 (0.04–0.089)

Sequela	Health state	Lay description	Disability weight
Moderate motor impairment, mild respiratory problems, and speech problems due to motor neuron disease	Moderate motor impairment, mild COPD and speech problems	(combined DW)	--
Moderate motor impairment, moderate respiratory problems, and speech problems due to motor neuron disease	Moderate motor impairment, moderate COPD and speech problems	(combined DW)	--
Moderate motor impairment, severe respiratory problems, and speech problems due to motor neuron disease	Moderate motor impairment, severe COPD and speech problems	(combined DW)	--
Moderate respiratory problems and speech problems due to motor neuron disease	Moderate COPD and speech problems	(combined DW)	--
Moderate respiratory problems due to motor neuron disease	COPD and other chronic respiratory problems, moderate	has cough, wheezing, and shortness of breath, even after light physical activity. The person feels tired and can walk only short distances or climb only a few stairs.	0.225 (0.153–0.31)
Severe motor impairment and mild respiratory problems due to motor neuron disease	Severe motor impairment and mild COPD	(combined DW)	--
Severe motor impairment and moderate respiratory problems due to motor neuron disease	Severe motor impairment and moderate COPD	(combined DW)	--

Sequela	Health state	Lay description	Disability weight
Severe motor impairment and severe respiratory problems due to motor neuron disease	Severe motor impairment and severe COPD	(combined DW)	--
Severe motor impairment and speech problems due to motor neuron disease	Severe motor impairment and speech problems	(combined DW)	--
Severe motor impairment due to motor neuron disease	Motor impairment, severe	is unable to move around without help, and is not able to lift or hold objects, get dressed, or sit upright.	0.402 (0.268–0.545)
Severe motor impairment, mild respiratory problems, and speech problems due to motor neuron disease	Severe motor impairment, mild COPD and speech problems	(combined DW)	--
Severe motor impairment, moderate respiratory problems, and speech problems due to motor neuron disease	Severe motor impairment, moderate COPD and speech problems	(combined DW)	--
Severe motor impairment, severe respiratory problems, and speech problems due to motor neuron disease	Severe motor impairment, severe COPD and speech problems	(combined DW)	--
Severe respiratory problems and speech problems due to motor neuron disease	Severe COPD and speech problems	(combined DW)	--
Severe respiratory problems due to motor neuron disease	Severe COPD	has cough, wheezing, and shortness of breath all the time. The person has great difficulty walking even short	0.408 (0.273–0.556)

Sequela	Health state	Lay description	Disability weight
		distances or climbing any stairs, feels tired when at rest, and is anxious.	
Speech problems due to motor neuron disease	Speech problems	has difficulty speaking, and others find it difficult to understand.	0.051 (0.032–0.078)
Asymptomatic migraine	--	--	--
Symptomatic migraine	Headache, migraine	has severe, throbbing head pain and nausea that cause great difficulty in daily activities and sometimes confine the person to bed. Moving around, light, and noise make it worse.	0.441 (0.294–0.588)
Asymptomatic tension-type headache	--	--	--
Symptomatic tension-type headache	Headache, tension-type	has a moderate headache that also affects the neck, which causes difficulty in daily activities.	0.037 (0.022–0.057)
Asymptomatic medication overuse headache	--	--	--
Symptomatic medication overuse headache	Headache, medication overuse	has daily headaches, felt as dull pain and often lasting all day, with poor sleep, nausea and fatigue. The person takes medicine for the headaches, which provides little relief but is needed to avoid having worse symptoms.	0.217 (0.138–0.311)
Other neurological disorders	--	--	--
Guillain-Barré syndrome due to other neurological disorders	Spinal cord lesion below neck level (treated)	is paralyzed from the waist down, cannot feel or move the legs, and has difficulties with urine and bowel control. The person uses a wheelchair to move around.	0.296 (0.198–0.414)

*Table modified from GBD 2015 Disease and Injury Incidence and Prevalence Collaborators. Global, regional, and national incidence, prevalence, and years lived with disability for 310 diseases and injuries, 1990-2015: a systematic analysis for the Global Burden of Disease Study 2015. *Lancet* 2016; **388**: 1545–602

Appendix Table 3: Total number of site years of cause of death data by neurological cause and source type for 2015

Cause	Vital registration	Verbal autopsy	Surveillance	Cancer registry
Meningitis	10,658	633	546	
Encephalitis	10,150	128		
Tetanus	10,578	621	393	
Brain and nervous system cancer	9,801	1		2,418
Cerebrovascular disease	10,660	691	1	
Ischaemic stroke	9,207			
Haemorrhagic stroke	9,211			
Neurological disorders	10,406	416	553	
Alzheimer's disease and other dementias	9,922	1		
Parkinson's disease	8,969			
Epilepsy	10,282	324		
Multiple sclerosis	7,663			
Motor neuron disease	8,122			
Other neurological disorders	8,159			

*****Table modified from GBD 2015 Mortality and Causes of Death Collaborators. Global, regional, and national life expectancy, all-cause mortality, and cause-specific mortality for 249 causes of death, 1980-2015: a systematic analysis for the Global Burden of Disease Study 2015. *Lancet* 2016; 388: 1459–544

Appendix Table 4. Data representativeness Index (DRI), the percentage of GBD 2015 geographies with any data by cause, pertaining to periods before 2005, 2005–2015, and all years of data

Disorder	<2005	2005-2015	Total
Meningitis	45.6%	33.0%	50.0%
Encephalitis	28.6%	19.4%	31.6%
Tetanus	65.0%	59.7%	68.0%
Brain and nervous system cancer	52.4%	44.2%	52.9%
Ischaemic stroke	62.6%	60.7%	68.0%
Haemorrhagic stroke	62.6%	60.7%	68.0%
Alzheimer’s disease and other dementias	20.4%	16.0%	23.3%
Parkinson’s disease	19.4%	12.6%	24.8%
Epilepsy	23.8%	6.3%	25.2%
Multiple sclerosis	25.2%	10.7%	27.2%
Motor neuron disease	10.2%	5.3%	10.7%
Migraine	19.4%	14.1%	23.3%
Tension-type headache	11.2%	13.6%	18.4%
Medication overuse headache	4.9%	10.2%	11.7%
Other neurological disorders	8.3%	1.0%	8.3%

The percentage is calculated out of a total of 195 countries and territories. GBD = Global Burden of Disease

*Table modified from GBD 2015 Disease and Injury Incidence and Prevalence Collaborators. Global, regional, and national incidence, prevalence, and years lived with disability for 310 diseases and injuries, 1990-2015: a systematic analysis for the Global Burden of Disease Study 2015. *Lancet* 2016; **388**: 1545–602

Appendix Table 5. Socio-demographic Index quintiles for 195 countries and territories, based on their 2015 values

Location	SDI level
Andorra	High SDI
Antigua and Barbuda	High SDI
Australia	High SDI
Austria	High SDI
Belarus	High SDI
Belgium	High SDI
Bermuda	High SDI
Brunei	High SDI
Canada	High SDI
Cyprus	High SDI
Czech Republic	High SDI
Denmark	High SDI
Estonia	High SDI
Finland	High SDI
France	High SDI
Germany	High SDI
Guam	High SDI
Hungary	High SDI
Iceland	High SDI
Ireland	High SDI
Israel	High SDI
Italy	High SDI
Japan	High SDI
Kuwait	High SDI
Latvia	High SDI
Lithuania	High SDI
Luxembourg	High SDI
Netherlands	High SDI
New Zealand	High SDI
Northern Mariana Islands	High SDI
Norway	High SDI
Poland	High SDI
Puerto Rico	High SDI
Russia	High SDI
Singapore	High SDI
Slovakia	High SDI
Slovenia	High SDI
South Korea	High SDI
Sweden	High SDI

Location	SDI level
Switzerland	High SDI
Taiwan (Province of China)	High SDI
The Bahamas	High SDI
Trinidad and Tobago	High SDI
United Arab Emirates	High SDI
United Kingdom	High SDI
United States	High SDI
Virgin Islands, U.S.	High SDI
Albania	High-middle SDI
American Samoa	High-middle SDI
Argentina	High-middle SDI
Armenia	High-middle SDI
Azerbaijan	High-middle SDI
Bahrain	High-middle SDI
Barbados	High-middle SDI
Bosnia and Herzegovina	High-middle SDI
Bulgaria	High-middle SDI
Chile	High-middle SDI
Colombia	High-middle SDI
Costa Rica	High-middle SDI
Croatia	High-middle SDI
Cuba	High-middle SDI
Dominica	High-middle SDI
Dominican Republic	High-middle SDI
Ecuador	High-middle SDI
Fiji	High-middle SDI
Georgia	High-middle SDI
Greece	High-middle SDI
Greenland	High-middle SDI
Grenada	High-middle SDI
Iran	High-middle SDI
Jamaica	High-middle SDI
Jordan	High-middle SDI
Kazakhstan	High-middle SDI
Lebanon	High-middle SDI
Macedonia	High-middle SDI
Malaysia	High-middle SDI
Malta	High-middle SDI
Mauritius	High-middle SDI
Mexico	High-middle SDI
Moldova	High-middle SDI
Mongolia	High-middle SDI

Location	SDI level
Montenegro	High-middle SDI
Oman	High-middle SDI
Panama	High-middle SDI
Peru	High-middle SDI
Portugal	High-middle SDI
Qatar	High-middle SDI
Romania	High-middle SDI
Saint Lucia	High-middle SDI
Saint Vincent and the Grenadines	High-middle SDI
Saudi Arabia	High-middle SDI
Serbia	High-middle SDI
Seychelles	High-middle SDI
South Africa	High-middle SDI
Spain	High-middle SDI
Sri Lanka	High-middle SDI
Suriname	High-middle SDI
Thailand	High-middle SDI
Turkey	High-middle SDI
Turkmenistan	High-middle SDI
Ukraine	High-middle SDI
Uruguay	High-middle SDI
Uzbekistan	High-middle SDI
Venezuela	High-middle SDI
Algeria	Middle SDI
Belize	Middle SDI
Bolivia	Middle SDI
Botswana	Middle SDI
Brazil	Middle SDI
China	Middle SDI
Egypt	Middle SDI
El Salvador	Middle SDI
Equatorial Guinea	Middle SDI
Federated States of Micronesia	Middle SDI
Gabon	Middle SDI
Guyana	Middle SDI
Honduras	Middle SDI
India	Middle SDI
Indonesia	Middle SDI
Iraq	Middle SDI
Kyrgyzstan	Middle SDI
Libya	Middle SDI
Maldives	Middle SDI

Location	SDI level
Marshall Islands	Middle SDI
Namibia	Middle SDI
Nicaragua	Middle SDI
North Korea	Middle SDI
Palestine	Middle SDI
Paraguay	Middle SDI
Philippines	Middle SDI
Samoa	Middle SDI
Swaziland	Middle SDI
Syria	Middle SDI
Tajikistan	Middle SDI
Tonga	Middle SDI
Tunisia	Middle SDI
Vietnam	Middle SDI
Angola	Low-middle SDI
Bangladesh	Low-middle SDI
Bhutan	Low-middle SDI
Cambodia	Low-middle SDI
Cameroon	Low-middle SDI
Cape Verde	Low-middle SDI
Congo (Brazzaville)	Low-middle SDI
Djibouti	Low-middle SDI
Ghana	Low-middle SDI
Guatemala	Low-middle SDI
Haiti	Low-middle SDI
Kenya	Low-middle SDI
Kiribati	Low-middle SDI
Laos	Low-middle SDI
Lesotho	Low-middle SDI
Morocco	Low-middle SDI
Myanmar	Low-middle SDI
Nepal	Low-middle SDI
Nigeria	Low-middle SDI
Pakistan	Low-middle SDI
Papua New Guinea	Low-middle SDI
Sao Tome and Principe	Low-middle SDI
Solomon Islands	Low-middle SDI
Sudan	Low-middle SDI
Tanzania	Low-middle SDI
Timor-Leste	Low-middle SDI
Vanuatu	Low-middle SDI
Yemen	Low-middle SDI

Location	SDI level
Zambia	Low-middle SDI
Zimbabwe	Low-middle SDI
Afghanistan	Low SDI
Benin	Low SDI
Burkina Faso	Low SDI
Burundi	Low SDI
Central African Republic	Low SDI
Chad	Low SDI
Comoros	Low SDI
Cote d'Ivoire	Low SDI
Democratic Republic of the Congo	Low SDI
Eritrea	Low SDI
Ethiopia	Low SDI
Guinea	Low SDI
Guinea-Bissau	Low SDI
Liberia	Low SDI
Madagascar	Low SDI
Malawi	Low SDI
Mali	Low SDI
Mauritania	Low SDI
Mozambique	Low SDI
Niger	Low SDI
Rwanda	Low SDI
Senegal	Low SDI
Sierra Leone	Low SDI
Somalia	Low SDI
South Sudan	Low SDI
The Gambia	Low SDI
Togo	Low SDI
Uganda	Low SDI

*****Table modified from GBD 2015 Mortality and Causes of Death Collaborators. Global, regional, and national life expectancy, all-cause mortality, and cause-specific mortality for 249 causes of death, 1980-2015: a systematic analysis for the Global Burden of Disease Study 2015. *Lancet* 2016; 388: 1459–544

Appendix Table 6. Global all-age numbers and age-standardised rates in 2015 and percent change between 1990 and 2015 for DALYs, deaths, and prevalence by neurological disorder among females

Table 6. Global all-age numbers and age-standardised rates in 2015 and percent change between 1990 and 2015 for DALYs, deaths, and prevalence by neurological disorder among females					
**Cause Name**	**Metric**	**All age numbers (thousands)**		**Age-standardised rate (per 100,000)**	
		**2015**	**Percent change from 1990 to 2015**	**2015**	**Percent change from 1990 to 2015**
Tetanus	DALYs	1,405 (1,128 - 1,834)	-88.2 (-90.3 - -84.9)	39 (31 - 51)	-89.1 (-91.0 - -86.2)
	Deaths	23 (18 - 33)	-85.9 (-88.2 - -81.4)	1 (1 - 1)	-87.8 (-89.9 - -84.3)
	Prevalence	86 (84 - 88)	8.3 (0.7 - 13.9)	2 (2 - 2)	-16.6 (-22.6 - -12.7)
Meningitis	DALYs	11,367 (9,500 - 14,530)	-36.4 (-48.4 - -5.2)	315 (263 - 402)	-43.5 (-53.7 - -16.6)
	Deaths	169 (143 - 208)	-31.1 (-42.2 - -2.9)	5 (4 - 6)	-43.3 (-51.4 - -21.8)
	Prevalence	4,272 (4,078 - 4,468)	26.8 (22.4 - 31.8)	117 (112 - 123)	-10.7 (-13.9 - -7.2)
Encephalitis	DALYs	3,854 (3,307 - 4,618)	-18.7 (-37.2 - 8.4)	107 (92 - 128)	-31.6 (-46.9 - -10.7)
	Deaths	68 (60 - 84)	-8.2 (-29.5 - 23.1)	2 (2 - 2)	-31.8 (-47.7 - -10.0)
	Prevalence	1,969 (1,439 - 2,673)	10.2 (5.8 - 16.4)	54 (39 - 73)	-25.2 (-28.7 - -20.5)
Cerebrovascular disease	DALYs	51,291 (49,263 - 53,567)	7.7 (2.9 - 12.4)	1,452 (1,395 - 1,516)	-39.1 (-41.7 - -36.5)
	Deaths	3,095 (3,001 - 3,201)	24.2 (19.2 - 28.8)	88 (86 - 91)	-35.0 (-37.6 - -32.6)
	Prevalence	20,257 (20,083 - 20,422)	52.6 (51.7 - 53.7)	573 (568 - 577)	-11.8 (-12.3 - -11.1)

Alzheimer's disease and other dementias	DALYs	13,965 (11,801 - 16,448)	90.5 (86.8 - 94.5)	400 (338 - 471)	-5.1 (-6.9 - -3.2)
	Deaths	1,176 (974 - 1,377)	105.9 (101.7 - 111.3)	33 (28 - 39)	-2.5 (-4.4 - -0.3)
	Prevalence	28,614 (24,998 - 32,823)	105.8 (103.3 - 108.1)	823 (718 - 945)	4.1 (3.2 - 5.0)
Parkinson's disease	DALYs	865 (766 - 984)	93.7 (86.5 - 99.4)	25 (22 - 28)	4.5 (0.5 - 7.6)
	Deaths	51 (49 - 53)	129.2 (115.4 - 140.2)	2 (1 - 2)	16.0 (9.0 - 21.5)
	Prevalence	2,849 (2,633 - 3,110)	107.9 (103.0 - 113.2)	82 (76 - 89)	13.0 (10.2 - 15.8)
Epilepsy	DALYs	5,207 (4,265 - 6,166)	-0.1 (-11.9 - 9.4)	143 (117 - 169)	-24.2 (-32.5 - -17.5)
	Deaths	47 (46 - 51)	18.6 (-6.5 - 31.1)	1 (1 - 1)	-16.6 (-32.1 - -8.3)
	Prevalence	11,029 (10,156 - 11,990)	39.0 (33.2 - 44.9)	303 (279 - 330)	1.8 (-2.2 - 6.1)
Multiple sclerosis	DALYs	767 (639 - 899)	44.8 (31.2 - 58.0)	21 (17 - 24)	-13.9 (-21.8 - -6.5)
	Deaths	11 (10 - 12)	43.8 (24.3 - 61.0)	0 (0 - 0)	-18.6 (-29.0 - -10.0)
	Prevalence	1,330 (1,231 - 1,436)	60.1 (56.0 - 64.3)	36 (33 - 39)	-3.3 (-5.7 - -0.8)
Migraine	DALYs	21,343 (13,177 - 31,691)	48.9 (46.1 - 51.8)	575 (355 - 854)	0.0 (-1.4 - 1.2)
	Deaths*				
	Prevalence	624,294 (570,018 - 684,027)	49.0 (46.0 - 51.9)	16,818 (15,366 - 18,428)	-0.4 (-1.6 - 0.8)
Tension-type headache	DALYs	1,335 (624 - 2,483)	46.5 (43.3 - 50.1)	36 (17 - 67)	-0.9 (-2.3 - 0.6)

	Deaths*				
	Prevalence	891,602 (796,087 - 989,527)	46.5 (43.3 - 50.1)	24,045 (21,473 - 26,675)	-1.1 (-2.5 - 0.3)
Medication overuse headache	DALYs	5,364 (3,587 - 7,624)	56.2 (49.2 - 63.3)	145 (97 - 206)	-1.6 (-5.7 - 2.7)
	Deaths*				
	Prevalence	34,352 (29,883 - 39,398)	56.2 (49.3 - 63.1)	928 (808 - 1,064)	-1.9 (-5.9 - 2.4)
Motor neuron disease	DALYs	378 (353 - 402)	43.7 (5.3 - 64.1)	11 (10 - 11)	-7.2 (-28.9 - 0.6)
	Deaths	16 (15 - 16)	86.4 (44.9 - 97.4)	0 (0 - 1)	7.9 (-13.6 - 13.5)
	Prevalence	89 (84 - 96)	66.3 (63.3 - 69.2)	3 (2 - 3)	0.3 (-1.3 - 1.7)
Brain and nervous system cancer	DALYs	3,280 (3,079 - 3,452)	37.9 (21.7 - 66.3)	90 (85 - 95)	-8.4 (-18.3 - 8.2)
	Deaths	102 (96 - 106)	65.7 (50.0 - 87.7)	3 (3 - 3)	0.2 (-8.7 - 11.4)
	Prevalence	557 (503 - 618)	63.7 (36.9 - 96.9)	16 (14 - 17)	10.5 (-5.9 - 29.7)
Other neurological disorders	DALYs	1,007 (917 - 1,185)	38.5 (17.2 - 49.6)	28 (26 - 33)	-8.3 (-15.9 - -2.3)
	Deaths	24 (23 - 27)	54.8 (41.7 - 62.8)	1 (1 - 1)	-7.0 (-12.7 - -2.7)

*No deaths assigned to headaches.
Prevalence is an aggregate of all sequelae for a condition.

Appendix Table 7. Global all-age numbers and age-standardised rates in 2015 and percent change between 1990 and 2015 for DALYs, deaths, and prevalence by neurological disorder among males

Table 7. Global all-age numbers and age-standardised rates in 2015 and percent change between 1990 and 2015 for DALYs, deaths, and prevalence by neurological disorder among males

Cause Name	Metric	All age numbers (thousands)		Age-standardised rate (per 100,000)	
		2015	Percent change from 1990 to 2015	2015	Percent change from 1990 to 2015
Tetanus	DALYs	2,105 (1,701 - 2,869)	-84·6 (-87·4 - -80·0)	55 (45 - 76)	-85·9 (-88·3 - -82·0)
	Deaths	34 (27 - 52)	-81·4 (-84·6 - -74·2)	1 (1 - 1)	-83·9 (-86·5 - -78·7)
	Prevalence	123 (120 - 127)	12·3 (6·8 - 17·1)	3 (3 - 3)	-14·8 (-19·5 - -11·1)
Meningitis	DALYs	14,028 (11,355 - 17,261)	-27·8 (-41·1 - -2·3)	370 (300 - 453)	-36·4 (-47·4 - -15·4)
	Deaths	210 (167 - 255)	-19·9 (-32·6 - 4·8)	6 (5 - 7)	-34·1 (-43·9 - -16·3)
	Prevalence	4,461 (4,262 - 4,669)	29·1 (24·2 - 34·3)	122 (117 - 128)	-10·9 (-14·2 - -7·4)
Encephalitis	DALYs	4,599 (4,199 - 5,007)	-9·6 (-21·4 - 3·1)	123 (112 - 134)	-26·8 (-36·2 - -17·6)
	Deaths	82 (75 - 89)	1·7 (-13·2 - 13·5)	2 (2 - 3)	-28·2 (-39·3 - -20·4)
	Prevalence	2,347 (1,708 - 3,194)	16·4 (12·3 - 21·8)	64 (46 - 87)	-22·8 (-25·9 - -18·3)
Cerebrovascular disease	DALYs	67,335 (64,770 - 69,982)	35·1 (29·2 - 41·5)	2,133 (2,057 - 2,215)	-26·3 (-29·4 - -22·9)
	Deaths	3,231 (3,129 - 3,346)	50·8 (45·0 - 58·0)	116 (112 - 120)	-24·8 (-27·5 - -21·4)
	Prevalence	22,174 (21,993 - 22,339)	65·6 (64·6 - 66·7)	686 (679 - 691)	-8·3 (-8·9 - -7·8)
Alzheimer’s disease and other dementias	DALYs	9,815 (8,276 - 11,442)	110·8 (106·3 - 115·7)	388 (328 - 455)	-6·2 (-7·8 - -4·4)
	Deaths	732 (610 - 853)	131·1 (125·4 - 136·9)	31 (26 - 36)	-4·1 (-5·8 - -2·1)
	Prevalence	17,342 (15,100 - 19,902)	122·4 (118·9 - 126·3)	673 (586 - 775)	1·7 (1·0 - 2·4)
Parkinson’s disease	DALYs	1,194 (1,070 - 1,337)	126·1 (113·2 - 136·9)	44 (39 - 49)	12·9 (6·6 - 18·2)
	Deaths	67 (64 - 70)	168·0 (145·9 - 186·8)	3 (3 - 3)	23·7 (13·7 - 32·5)
	Prevalence	3,344 (3,086 - 3,662)	127·0 (122·4 - 132·3)	117 (108 - 129)	16·2 (13·9 - 18·8)

Epilepsy	DALYs	7,211<br>(6,138 - 8,291)	4·5<br>(-3·2 - 14·9)	192<br>(163 - 221)	-21·4<br>(-26·5 - -14·7)
	Deaths	78<br>(73 - 82)	20·1<br>(10·1 - 38·5)	2<br>(2 - 2)	-14·3<br>(-20·9 - -4·8)
	Prevalence	12,385<br>(11,358 - 13,473)	39·3<br>(33·2 - 45·8)	337<br>(310 - 365)	1·9<br>(-2·4 - 6·4)
Multiple sclerosis	DALYs	466<br>(395 - 537)	38·7<br>(29·8 - 57·5)	13<br>(11 - 15)	-19·0<br>(-24·2 - -8·5)
	Deaths	8<br>(7 - 8)	34·9<br>(22·5 - 65·2)	0<br>(0 - 0)	-25·4<br>(-31·9 - -10·2)
	Prevalence	682<br>(635 - 733)	56·9<br>(53·8 - 60·4)	19<br>(18 - 20)	-6·1<br>(-7·8 - -4·3)
Migraine	DALYs	11,556<br>(7,097 - 17,287)	50·7<br>(47·3 - 53·9)	305<br>(188 - 456)	2·0<br>(0·5 - 3·4)
	Deaths*				
	Prevalence	334,495<br>(301,368 - 373,703)	50·8<br>(47·6 - 53·9)	8,836<br>(7,982 - 9,843)	1·7<br>(0·2 - 3·0)
Tension-type headache	DALYs	925<br>(432 - 1,705)	53·2<br>(49·7 - 57·0)	24<br>(11 - 45)	2·3<br>(0·7 - 3·9)
	Deaths*				
	Prevalence	614,290<br>(539,874 - 691,464)	53·2<br>(49·9 - 57·2)	16,240<br>(14,330 - 18,264)	2·1<br>(0·6 - 3·6)
Medication overuse headache	DALYs	3,800<br>(2,497 - 5,417)	60·0<br>(52·9 - 67·5)	103<br>(68 - 146)	0·5<br>(-3·6 - 4·7)
	Deaths*				
	Prevalence	24,103<br>(20,774 - 27,856)	60·1<br>(53·2 - 67·8)	652<br>(563 - 749)	0·2<br>(-3·8 - 4·4)
Motor neuron disease	DALYs	532<br>(503 - 566)	66·3<br>(50·7 - 77·0)	16<br>(15 - 17)	3·7<br>(-6·7 - 8·4)
	Deaths	20<br>(19 - 21)	107·0<br>(81·8 - 114·6)	1<br>(1 - 1)	15·6<br>(1·2 - 19·8)
	Prevalence	113<br>(106 - 121)	77·4<br>(73·9 - 80·9)	3<br>(3 - 4)	4·7<br>(3·1 - 6·6)
Brain and nervous system cancer	DALYs	4,345<br>(3,683 - 4,899)	37·2<br>(3·5 - 69·2)	122<br>(103 - 136)	-9·7<br>(-29·5 - 8·5)
	Deaths	127<br>(108 - 141)	65·8<br>(27·9 - 90·6)	4<br>(3 - 4)	-1·4<br>(-22·3 - 11·3)
	Prevalence	648<br>(551 - 738)	57·2<br>(10·5 - 101·3)	19<br>(16 - 21)	7·2<br>(-19·8 - 32·1)
Other neurological disorders	DALYs	1,353<br>(1,279 - 1,427)	27·1<br>(7·7 - 40·5)	39<br>(37 - 42)	-10·4<br>(-21·7 - -4·6)
	Deaths	30<br>(29 - 32)	52·4<br>(33·4 - 59·2)	1<br>(1-1)	-3·9<br>(-12·9 - 0·8)

*No deaths assigned to headaches. Prevalence is an aggregate of all sequelae for a condition.

Appendix Table 8. Global sex difference in age-standardised death, DALY and prevalence rates by neurological disorder, 2015

Cause Name	Metric	Male minus female age-standardised rate per 100,000
Tetanus	DALYs	16.5 (1.6, 37.8)
	Deaths	0.4 (-0.2, 1.1)
	Prevalence	0.9 (0.8, 1)
Meningitis	DALYs	55 (-21.6, 130.2)
	Deaths	1 (0, 2.3)
	Prevalence	4.6 (0, 9.3)
Encephalitis	DALYs	16 (-2.4, 32.3)
	Deaths	0.2 (-0.3, 1)
	Prevalence	9.9 (6.5, 14.4)
Cerebrovascular disease	DALYs	680.8 (595.6, 766)
	Deaths	24.8 (21.4, 28.3)
	Prevalence	115 (111, 119.4)
Alzheimer's disease and other dementias	DALYs	-31.9 (-64, -0.3)
	Deaths	0.4 (0, 0.9)
	Prevalence	-411.9 (-465.4, -362.7)
Parkinson's disease	DALYs	28.3 (25.3, 31.5)
	Deaths	0.6 (0.5, 0.8)
	Prevalence	54 (49.6, 60.1)
Epilepsy	DALYs	49.3 (40.8, 57.6)
	Deaths	0.7 (0.6, 0.9)
	Prevalence	33.4 (27.3, 40.4)
Multiple sclerosis	DALYs	-8.6 (-10.6, -6.6)
	Deaths	-0.1 (-0.2, 0)
	Prevalence	-18.9 (-20.6, -17.3)

Cause Name	Metric	Male minus female age-standardised rate per 100,000
Migraine	DALYs	-296.8 (-438.5, -182.1)
	Prevalence	-8772.5 (-9612.5, -8047.1)
Tension-type headache	DALYs	-12.7 (-23.3, -6)
	Prevalence	-8578 (-9387.4, -7800.2)
Medication overuse headache	DALYs	-46.5 (-65.6, -30.7)
	Prevalence	-303.8 (-361.5, -254.5)
Motor neuron disease	DALYs	5.1 (4.1, 6)
	Deaths	0.1 (0.1, 0.1)
	Prevalence	0.9 (0.8, 1)
Brain and nervous system cancer	DALYs	31.8 (11.5, 46.2)
	Deaths	1 (0.5, 1.6)
	Prevalence	3.3 (0, 6.4)
Other neurological disorders	DALYs	11.2 (6.7, 13.7)
	Deaths	0.2 (0, 0.3)

Appendix Figure S1. Twenty-one GBD regions

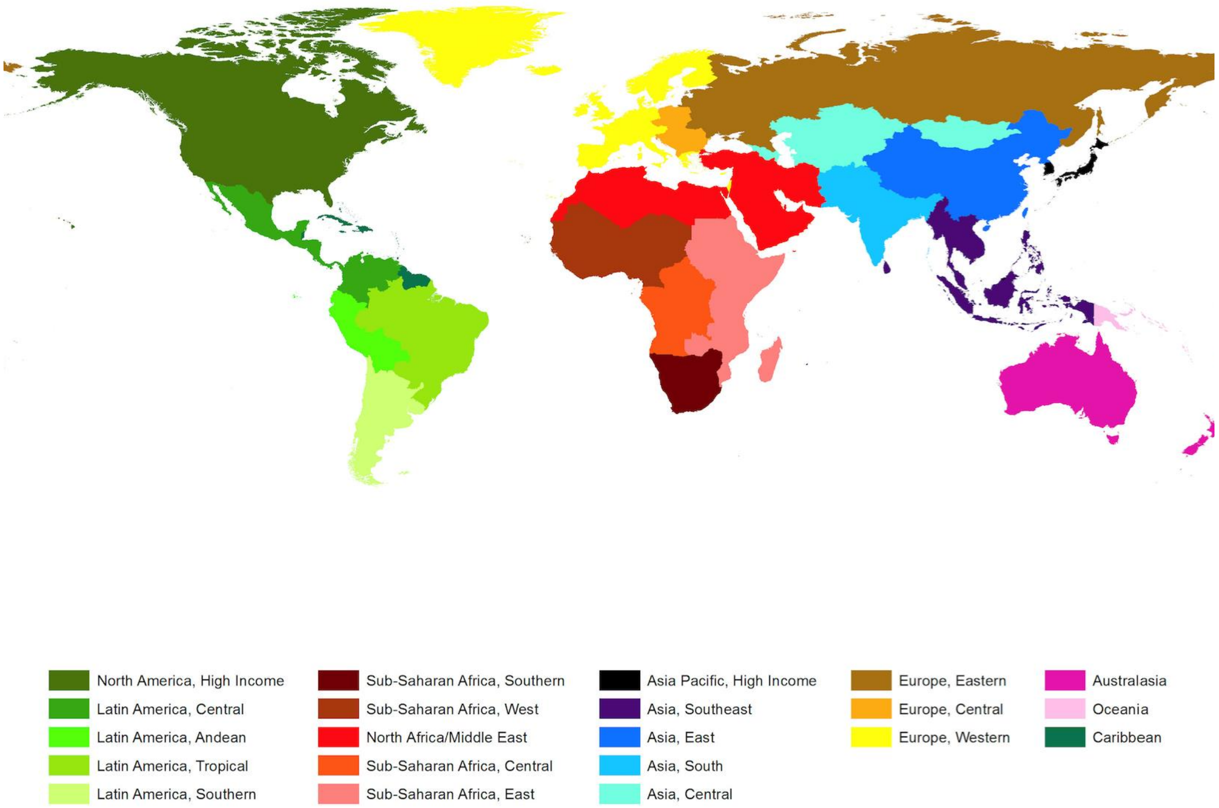

Appendix Figure 2. Geographical variations of the age-standardised prevalence rates per 100,000 from individual neurological disorders both sexes, 2015

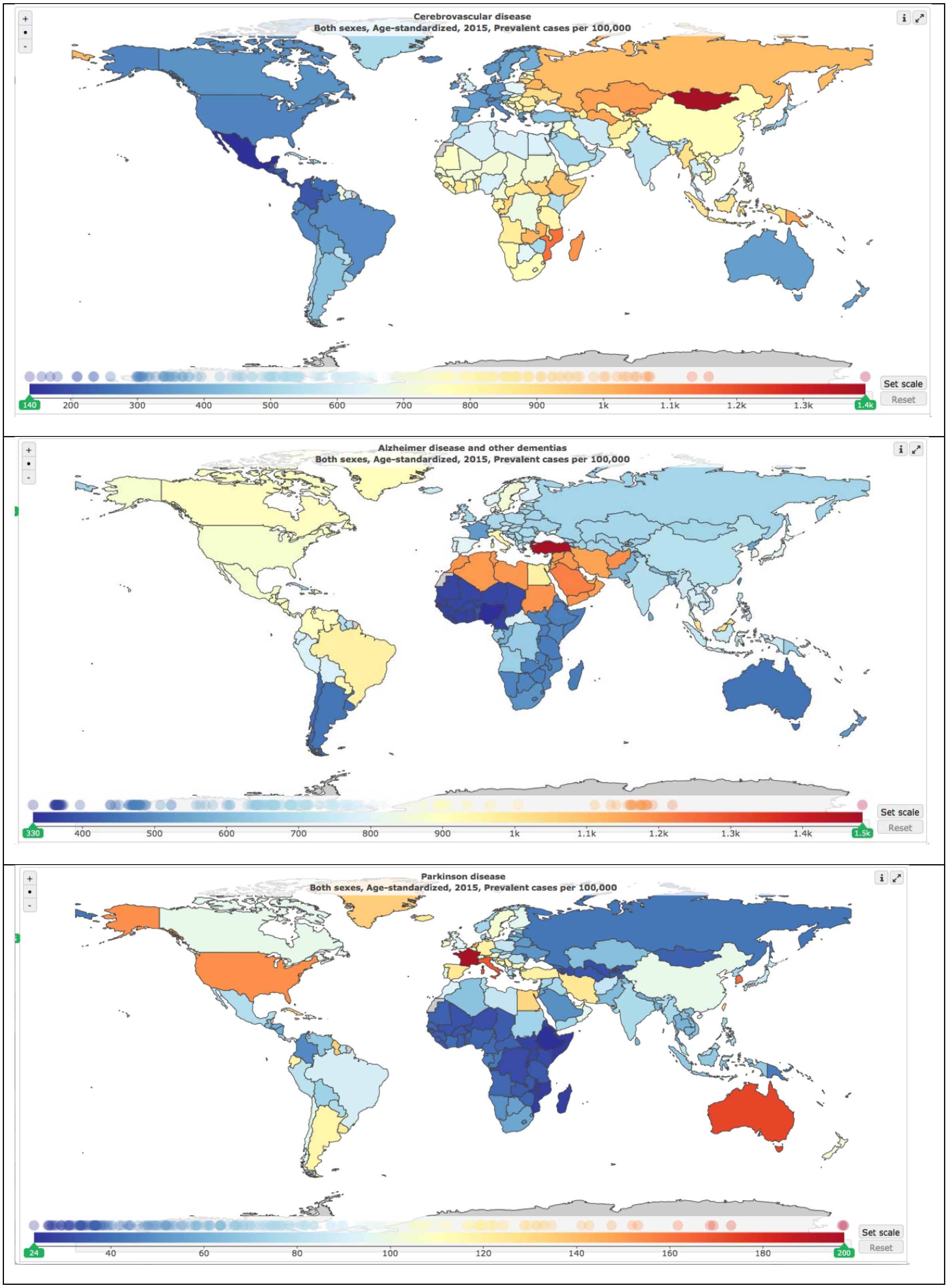

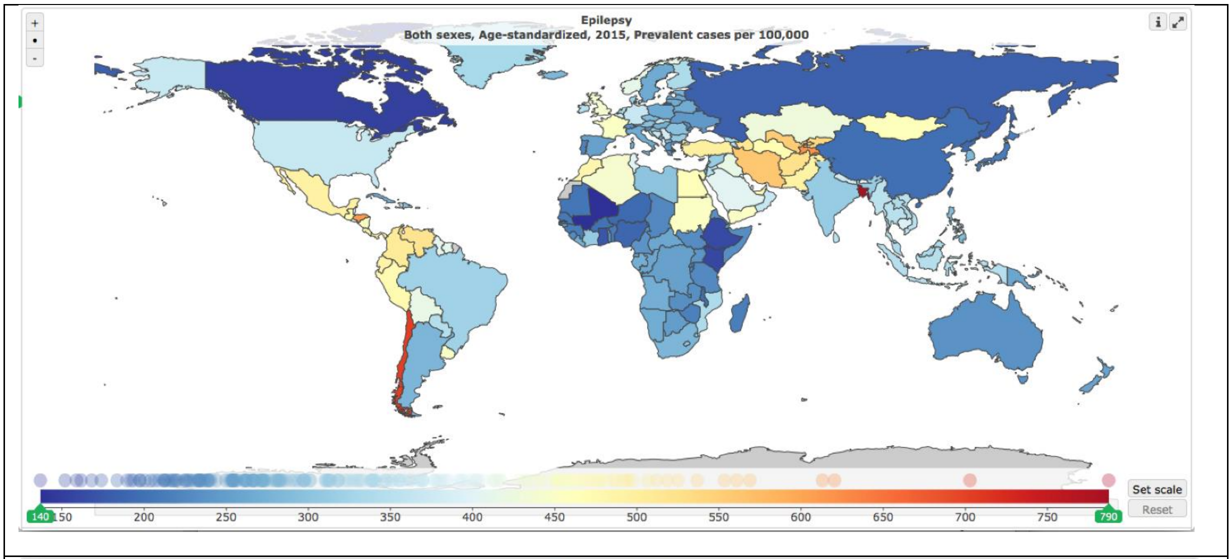
Epilepsy
Both sexes, Age-standardized, 2015, Prevalent cases per 100,000
140 150 200 250 300 350 400 450 500 550 600 650 700 750 790
Set scale
Reset

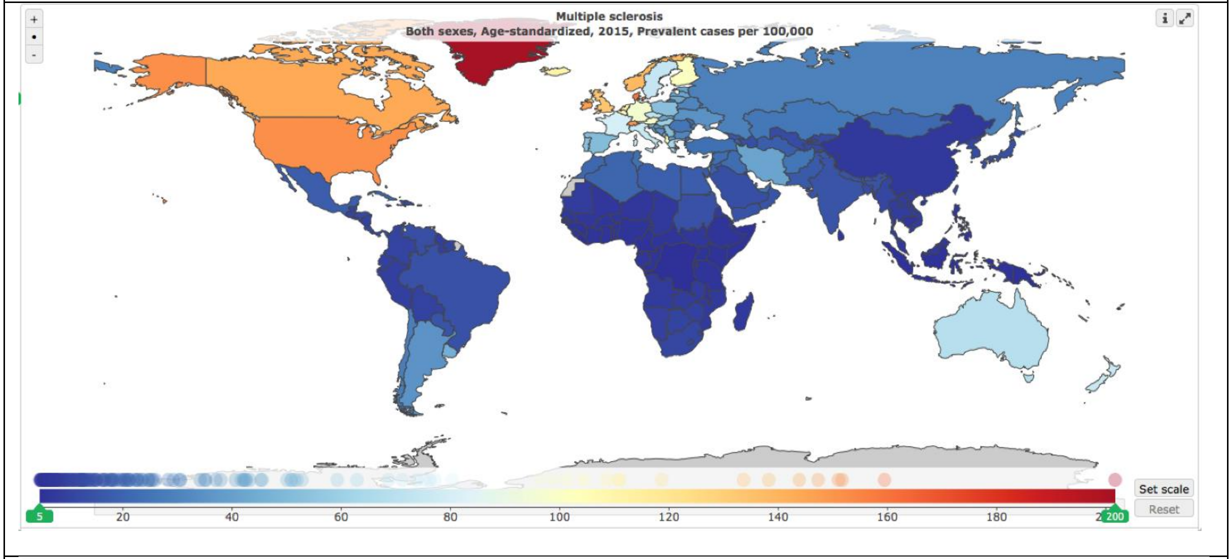
Multiple sclerosis
Both sexes, Age-standardized, 2015, Prevalent cases per 100,000
5 20 40 60 80 100 120 140 160 180 200
Set scale
Reset

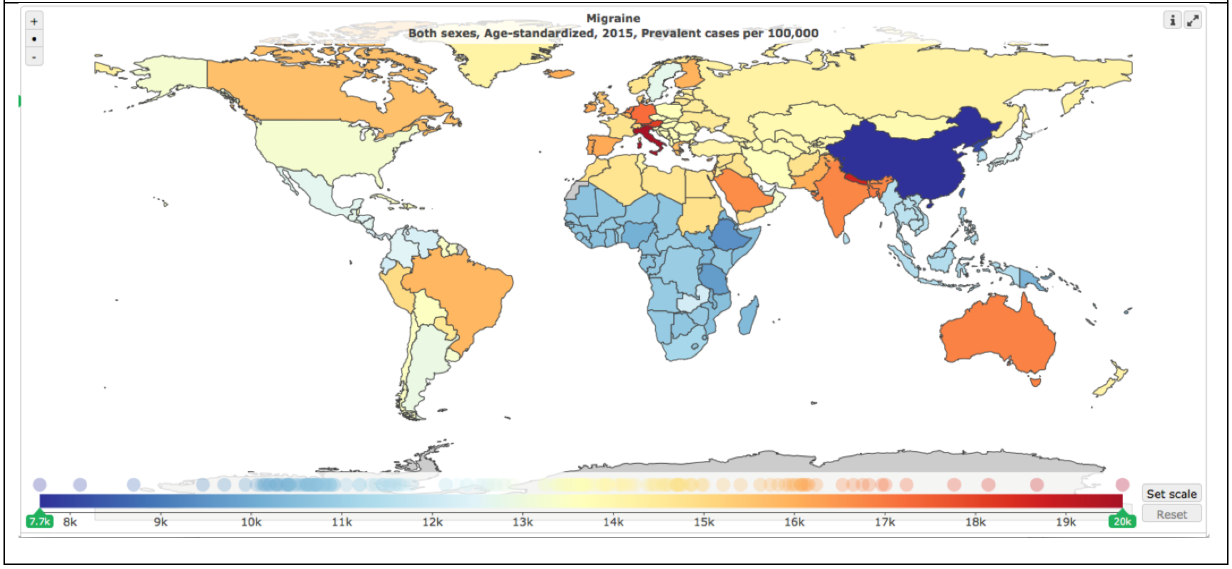
Migraine
Both sexes, Age-standardized, 2015, Prevalent cases per 100,000
7.7k 8k 9k 10k 11k 12k 13k 14k 15k 16k 17k 18k 19k 20k
Set scale
Reset

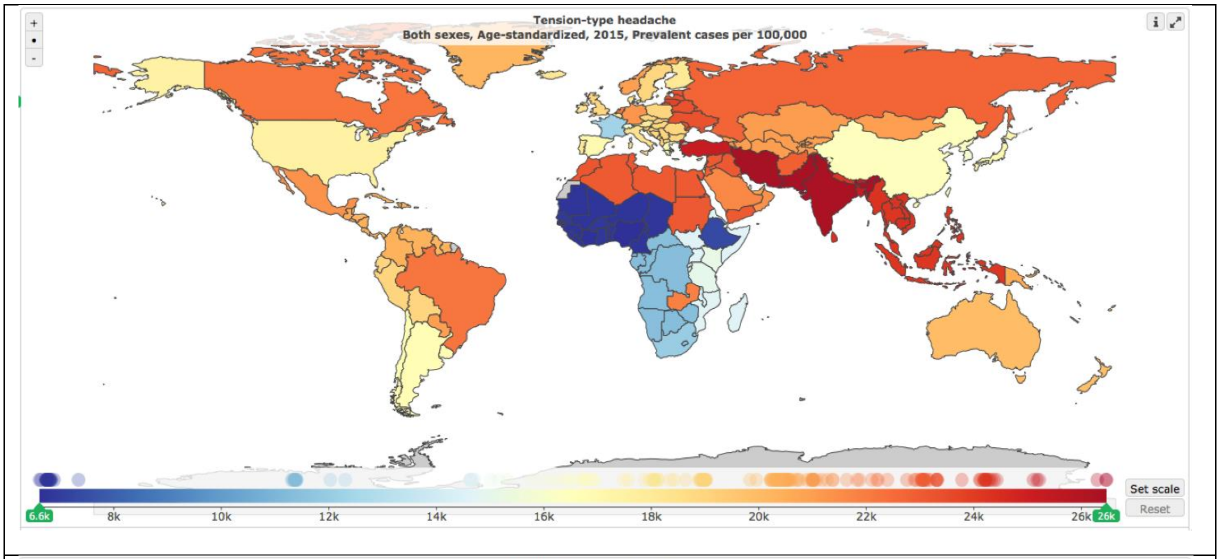
Tension-type headache
Both sexes, Age-standardized, 2015, Prevalent cases per 100,000
Set scale
Reset
6.6k 8k 10k 12k 14k 16k 18k 20k 22k 24k 26k 26k

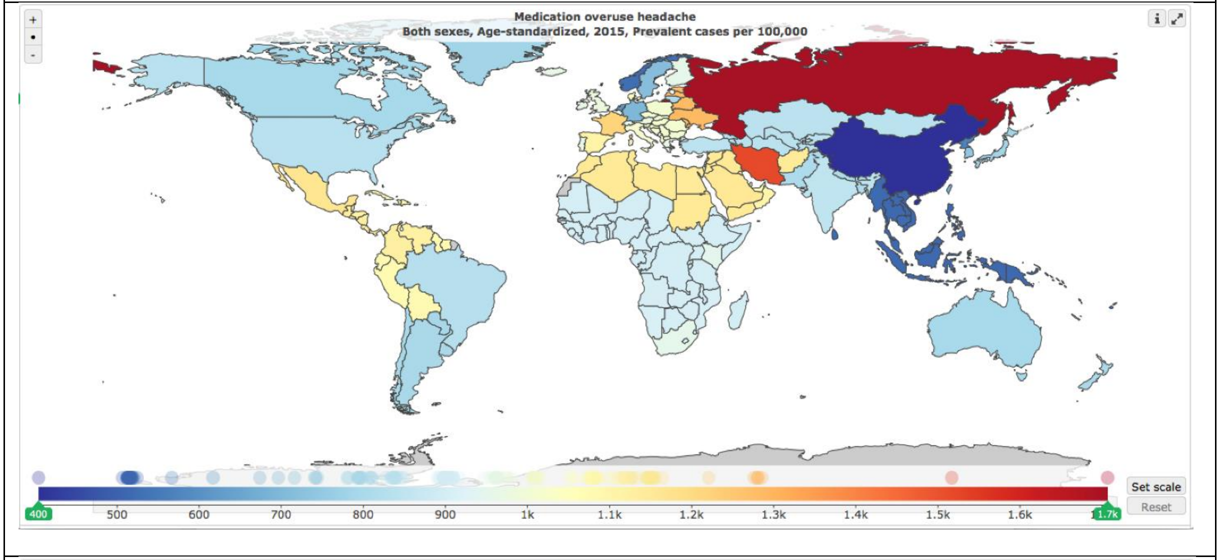
Medication overuse headache
Both sexes, Age-standardized, 2015, Prevalent cases per 100,000
Set scale
Reset
400 500 600 700 800 900 1k 1.1k 1.2k 1.3k 1.4k 1.5k 1.6k 1.7k

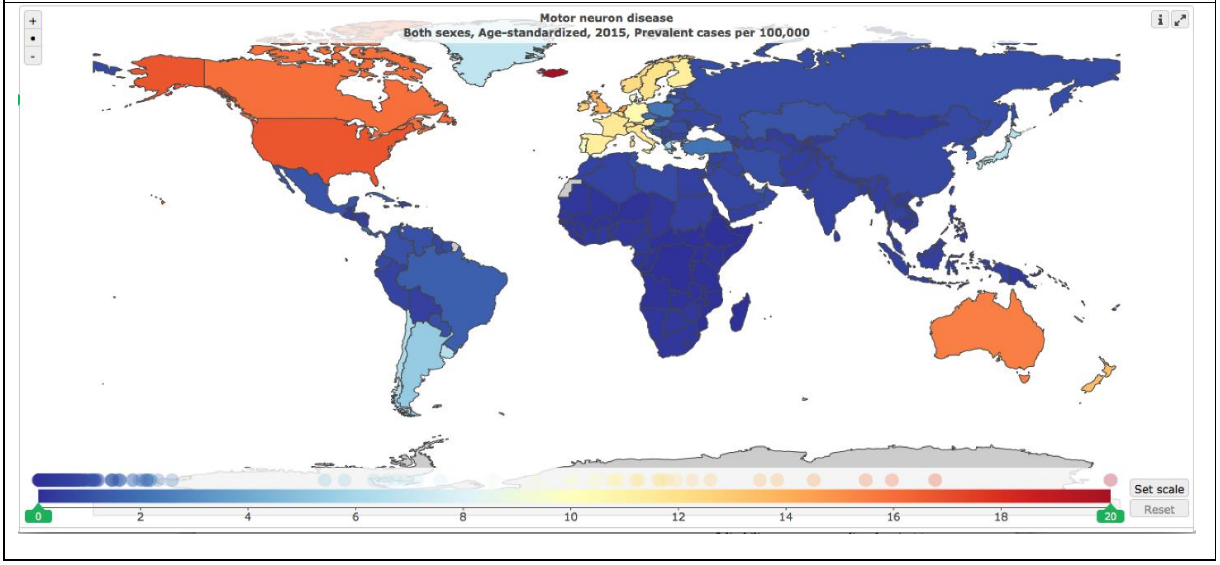
Motor neuron disease
Both sexes, Age-standardized, 2015, Prevalent cases per 100,000
Set scale
Reset
0 2 4 6 8 10 12 14 16 18 20

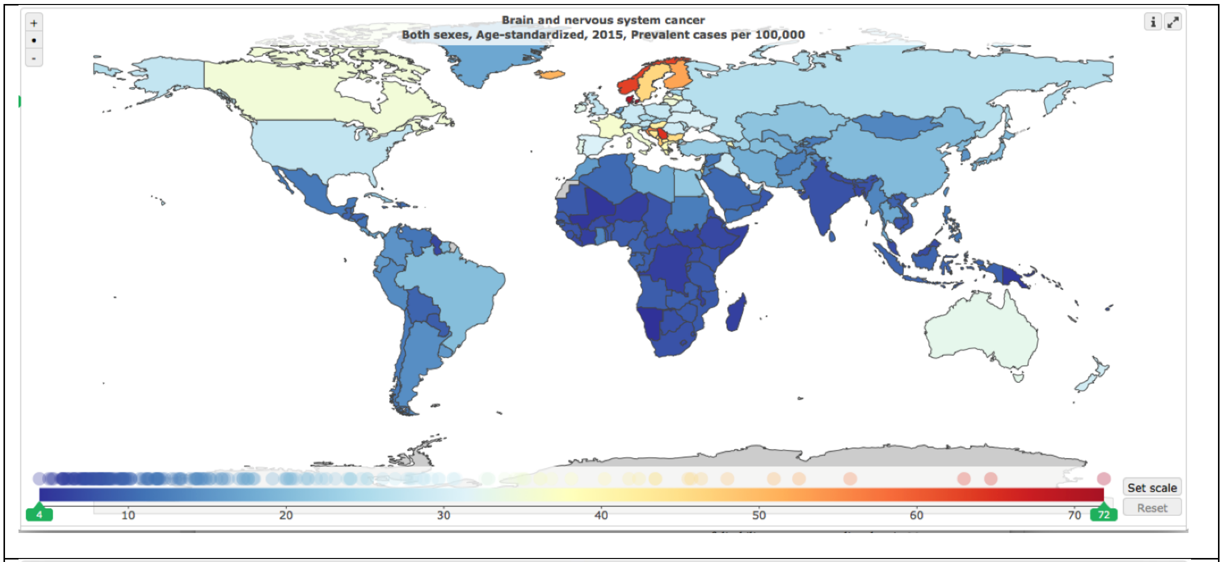
Brain and nervous system cancer
Both sexes, Age-standardized, 2015, Prevalent cases per 100,000
Set scale
Reset
4
10
20
30
40
50
60
70
72

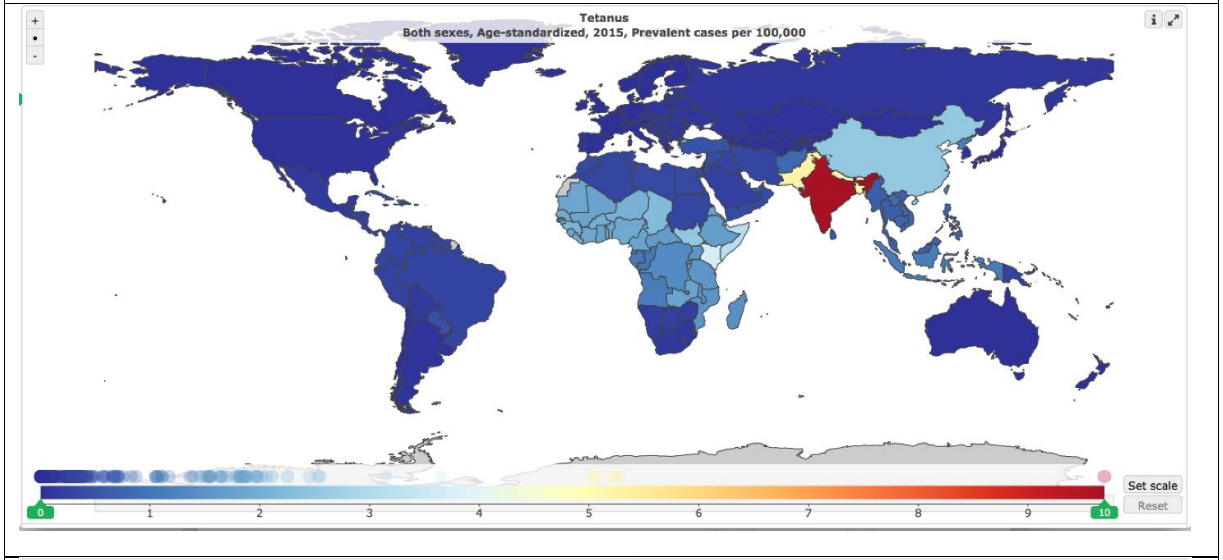
Tetanus
Both sexes, Age-standardized, 2015, Prevalent cases per 100,000
Set scale
Reset
0
1
2
3
4
5
6
7
8
9
10

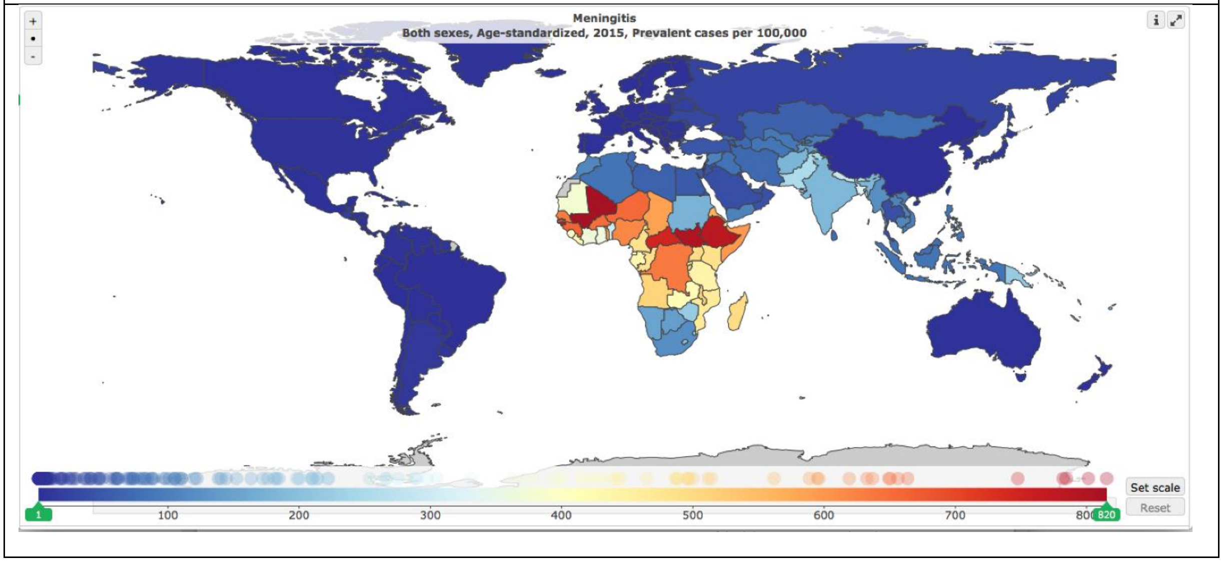
Meningitis
Both sexes, Age-standardized, 2015, Prevalent cases per 100,000
Set scale
Reset
1
100
200
300
400
500
600
700
800
820

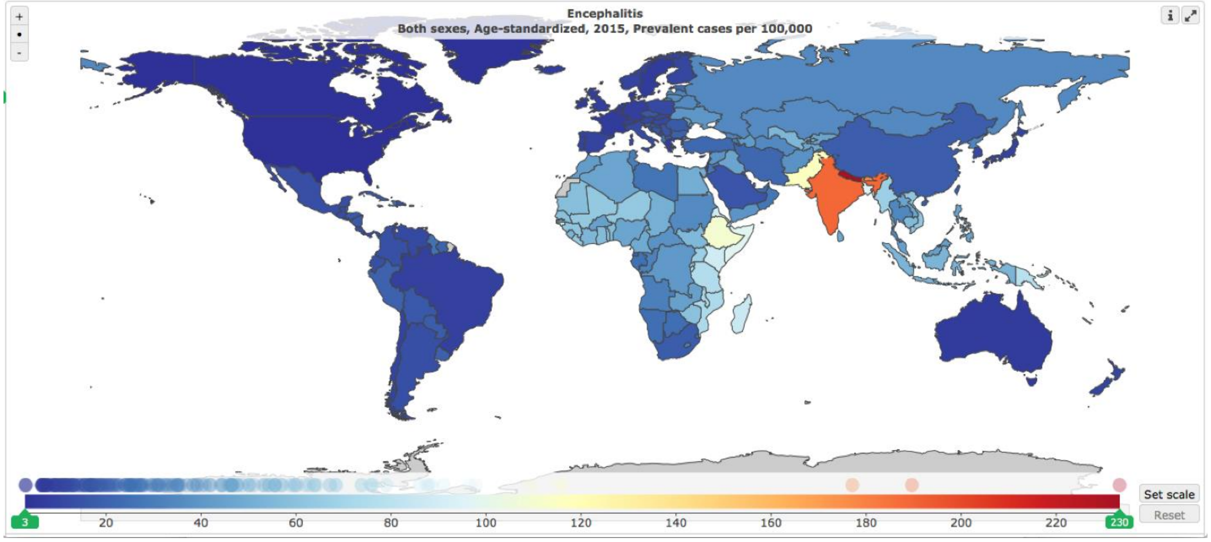
Encephalitis
Both sexes, Age-standardized, 2015, Prevalent cases per 100,000
3
20
40
60
80
100
120
140
160
180
200
220
230
Set scale
Reset

Appendix Figure 3. Geographical variations of the age-standardised DALY rates per 100,000 from individual neurological disorders both sexes, 2015

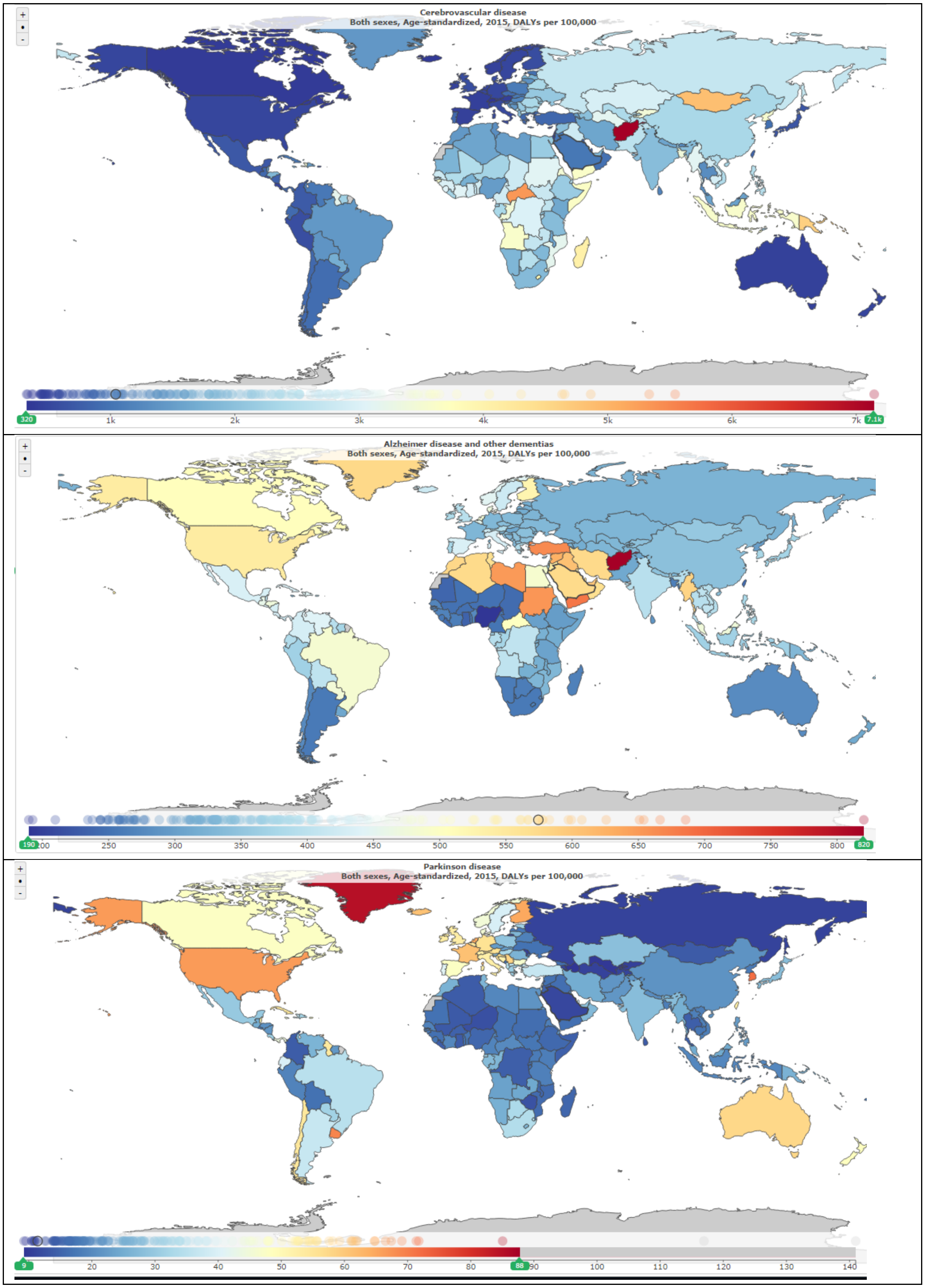

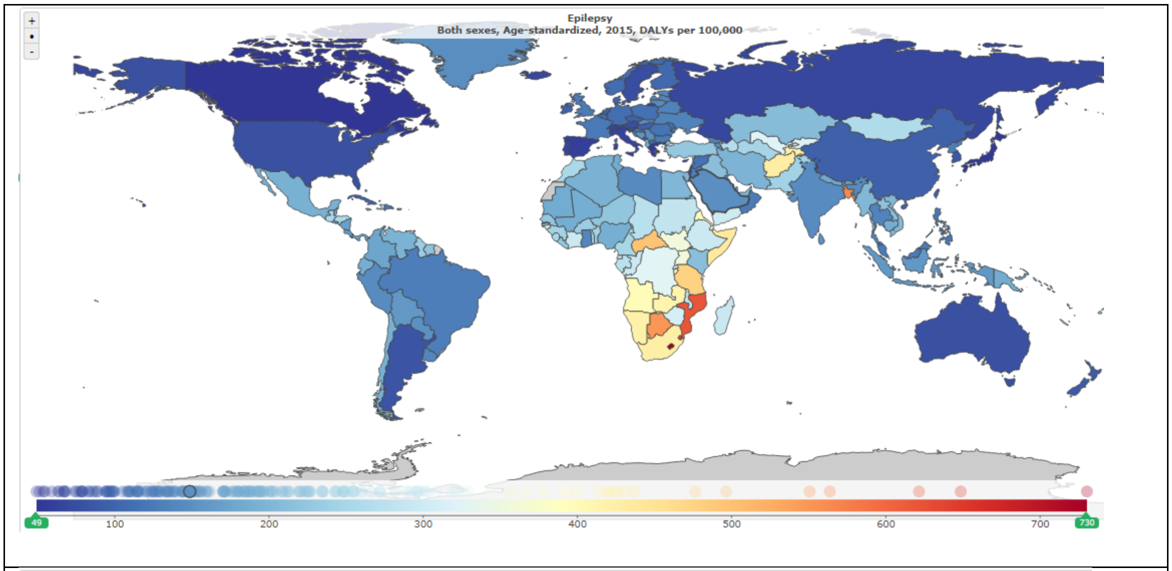
Epilepsy
Both sexes, Age-standardized, 2015, DALYs per 100,000
49
100
200
300
400
500
600
700
730

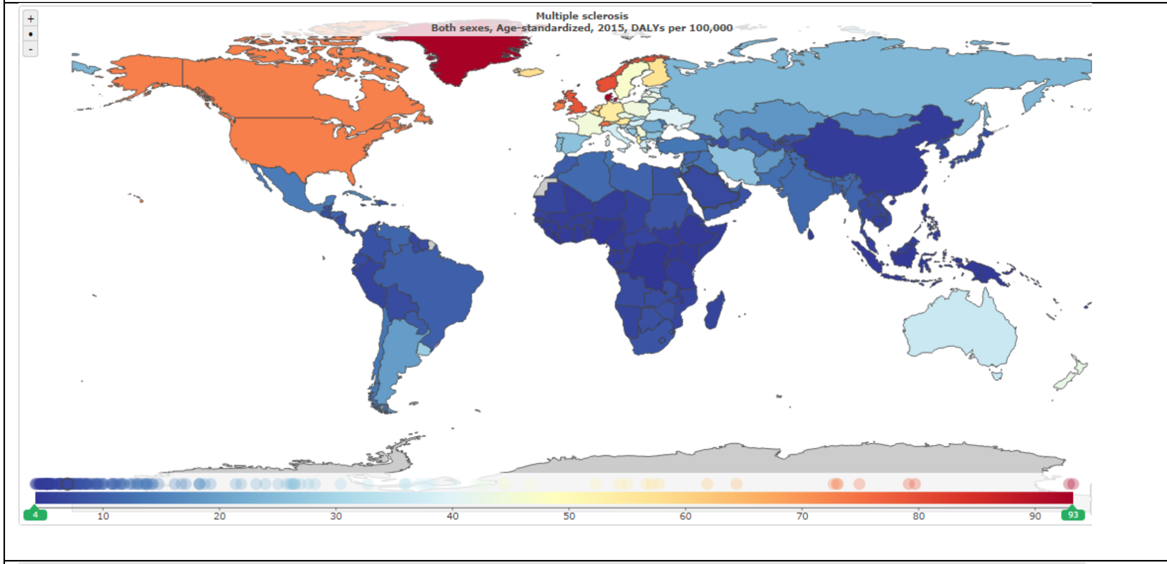
Multiple sclerosis
Both sexes, Age-standardized, 2015, DALYs per 100,000
4
10
20
30
40
50
60
70
80
90
93

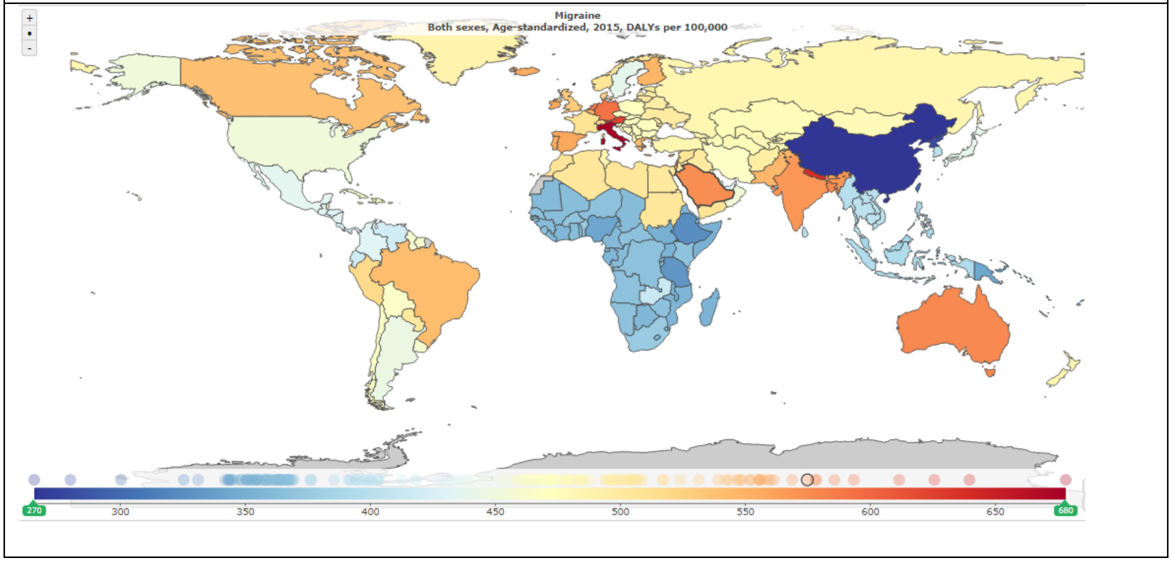
Migraine
Both sexes, Age-standardized, 2015, DALYs per 100,000
270
300
350
400
450
500
550
600
650
680

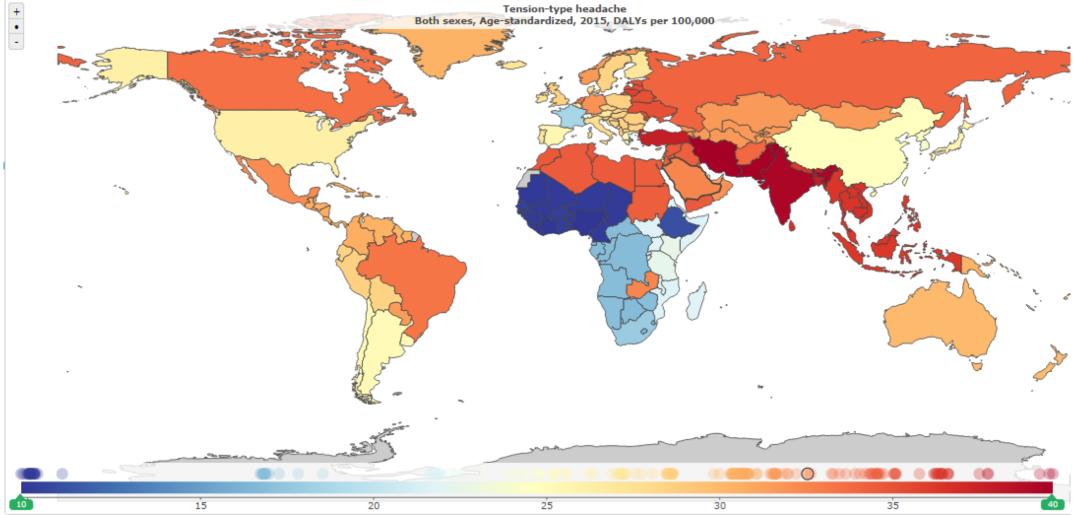
Tension-type headache
Both sexes, Age-standardized, 2015, DALYs per 100,000
10
15
20
25
30
35
40

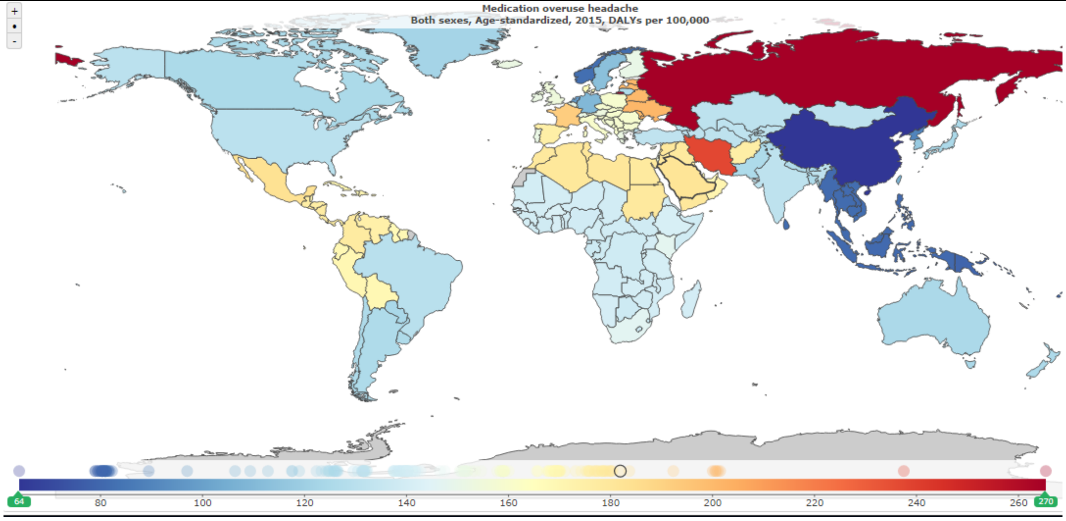
Medication overuse headache
Both sexes, Age-standardized, 2015, DALYs per 100,000
64
80
100
120
140
160
180
200
220
240
260
270

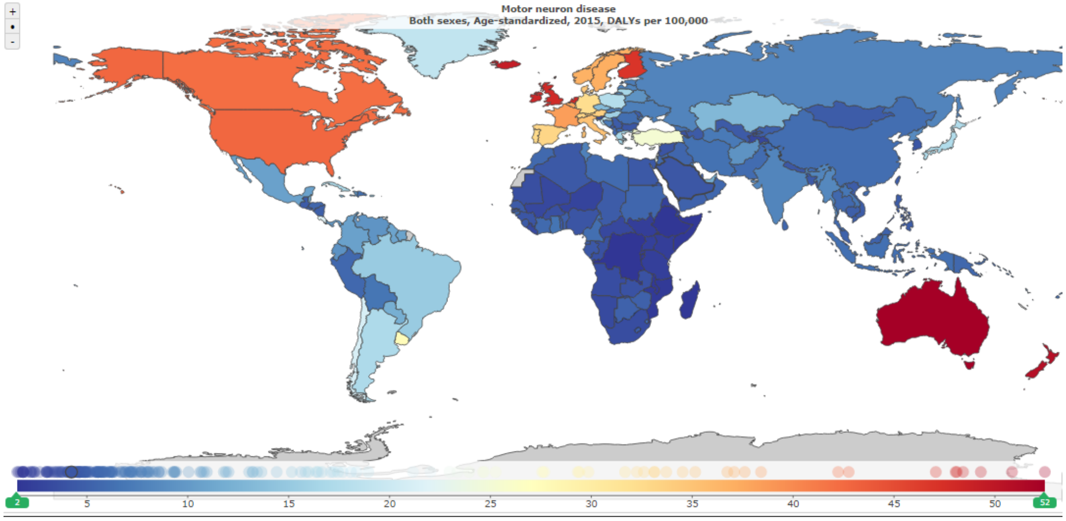
Motor neuron disease
Both sexes, Age-standardized, 2015, DALYs per 100,000
2
5
10
15
20
25
30
35
40
45
50
52

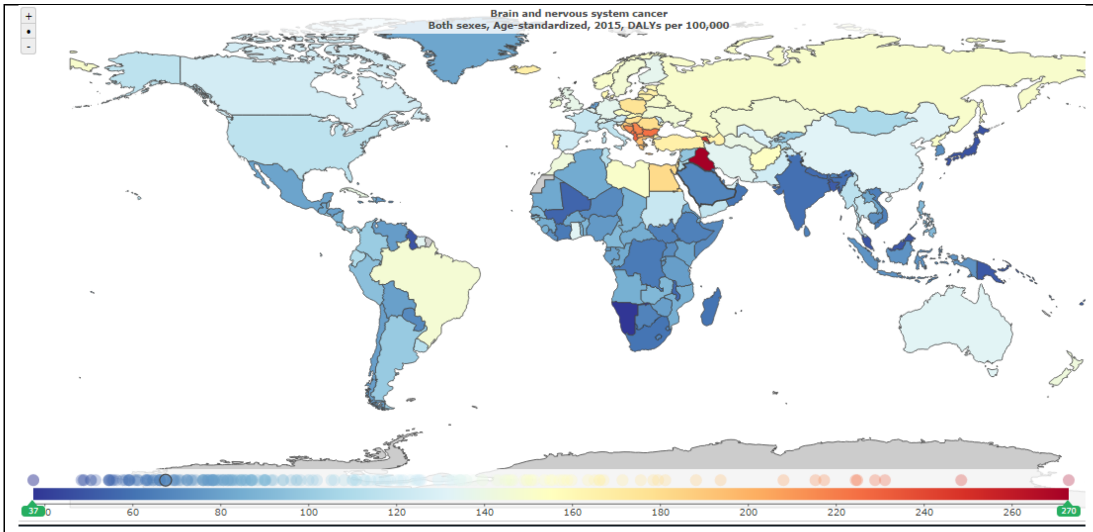
Brain and nervous system cancer
Both sexes, Age-standardized, 2015, DALYs per 100,000
37
60
80
100
120
140
160
180
200
220
240
260
270

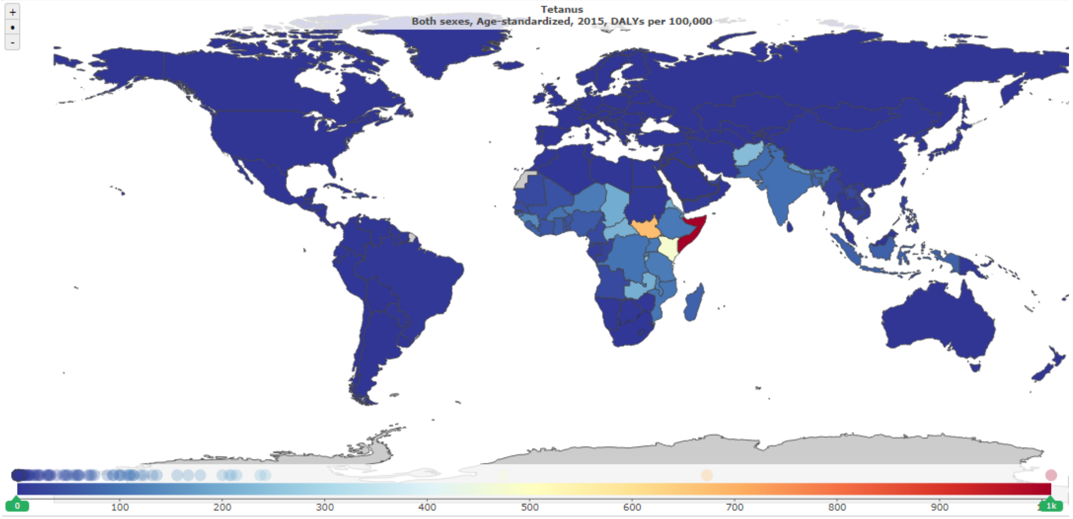
Tetanus
Both sexes, Age-standardized, 2015, DALYs per 100,000
0
100
200
300
400
500
600
700
800
900
1k

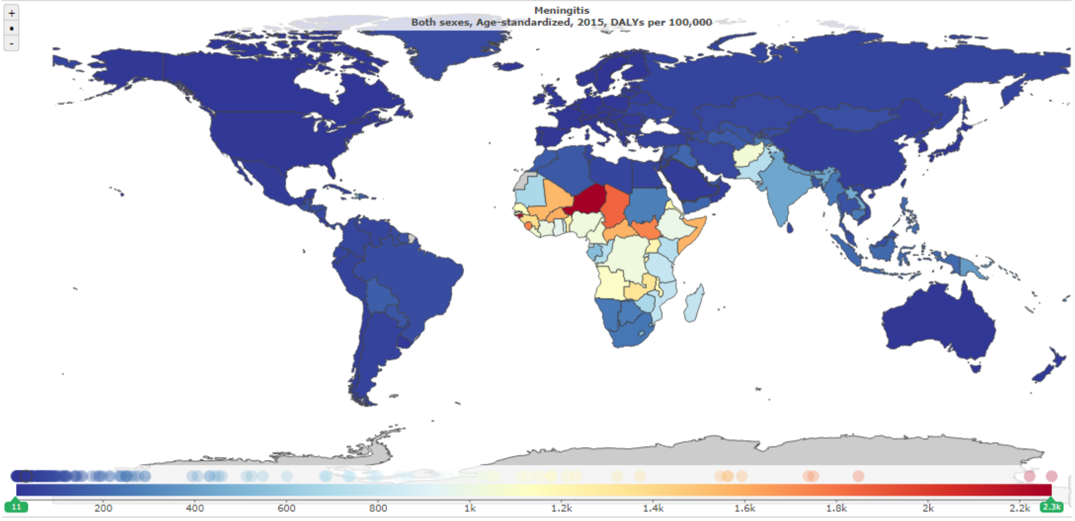
Meningitis
Both sexes, Age-standardized, 2015, DALYs per 100,000
11
200
400
600
800
1k
1.2k
1.4k
1.6k
1.8k
2k
2.2k
2.3k

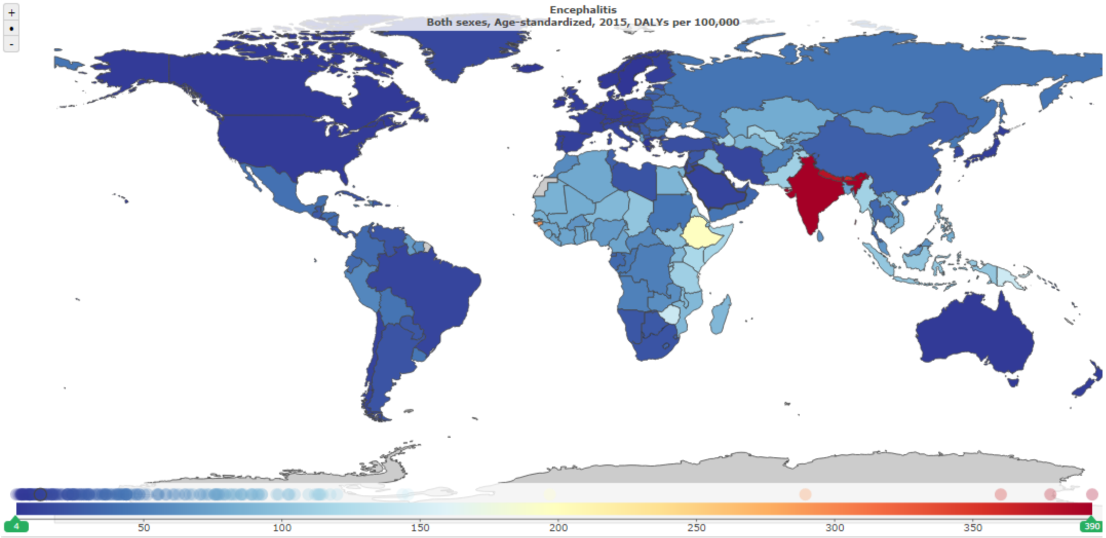
Encephalitis
Both sexes, Age-standardized, 2015, DALYs per 100,000
50
100
150
200
250
300
350
4
390

Appendix Figure 4. Geographical variations of the age-standardised death rates per 100,000 from individual neurological disorders, both sexes, 2015

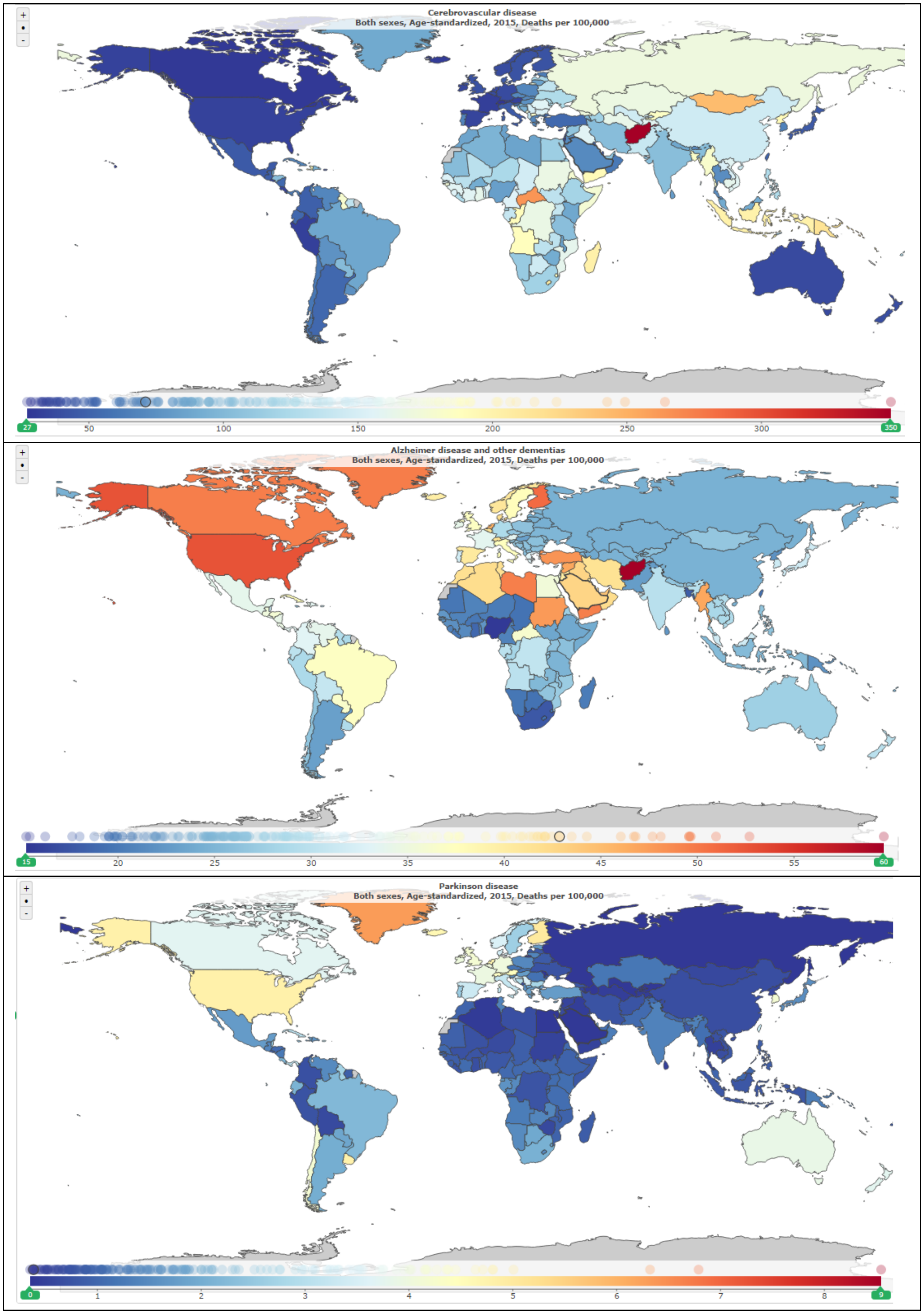

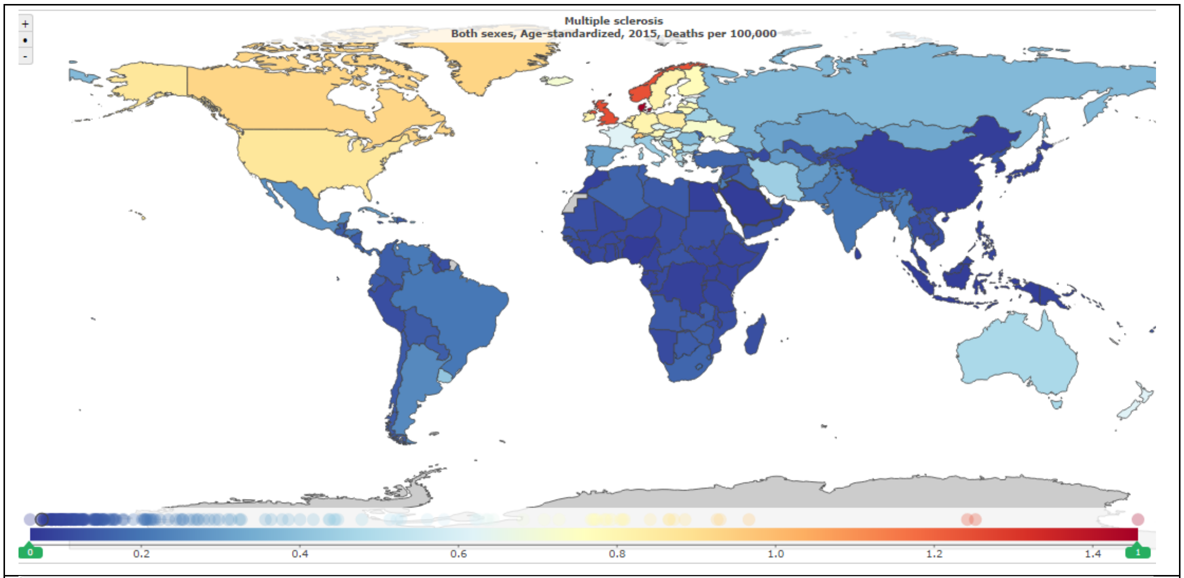
Multiple sclerosis
Both sexes, Age-standardized, 2015, Deaths per 100,000
0
0.2
0.4
0.6
0.8
1.0
1.2
1.4
1

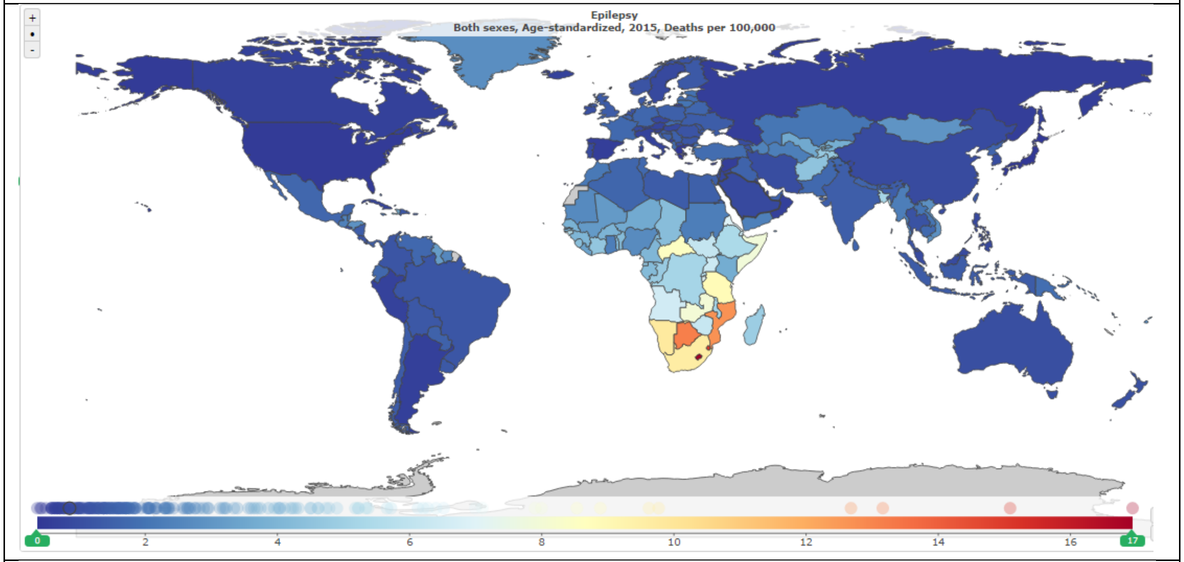
Epilepsy
Both sexes, Age-standardized, 2015, Deaths per 100,000
0
2
4
6
8
10
12
14
16
17

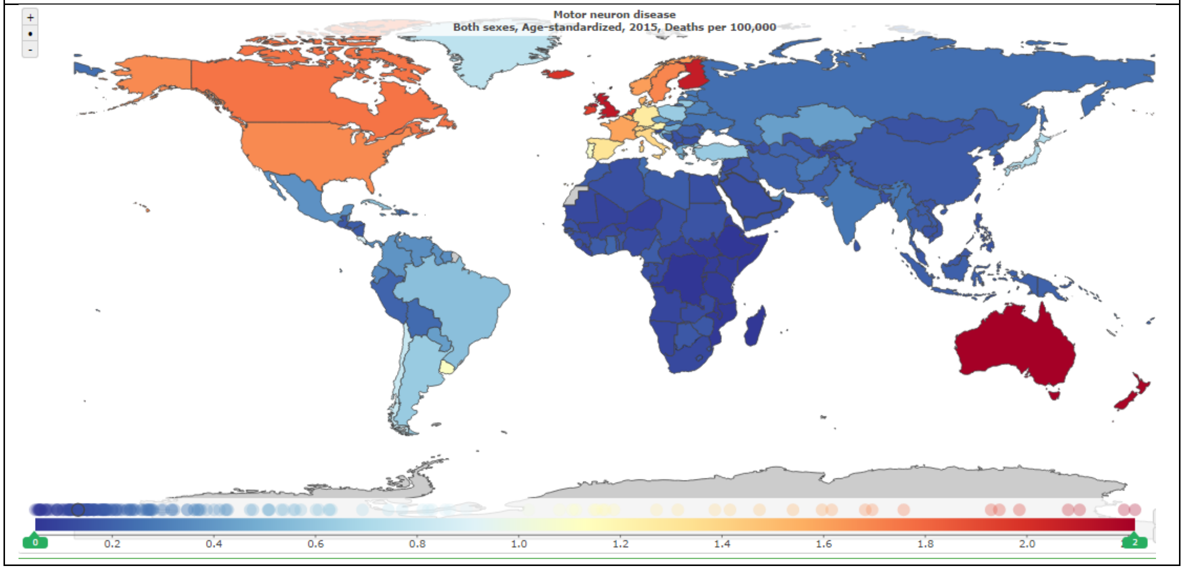
Motor neuron disease
Both sexes, Age-standardized, 2015, Deaths per 100,000
0
0.2
0.4
0.6
0.8
1.0
1.2
1.4
1.6
1.8
2.0
2

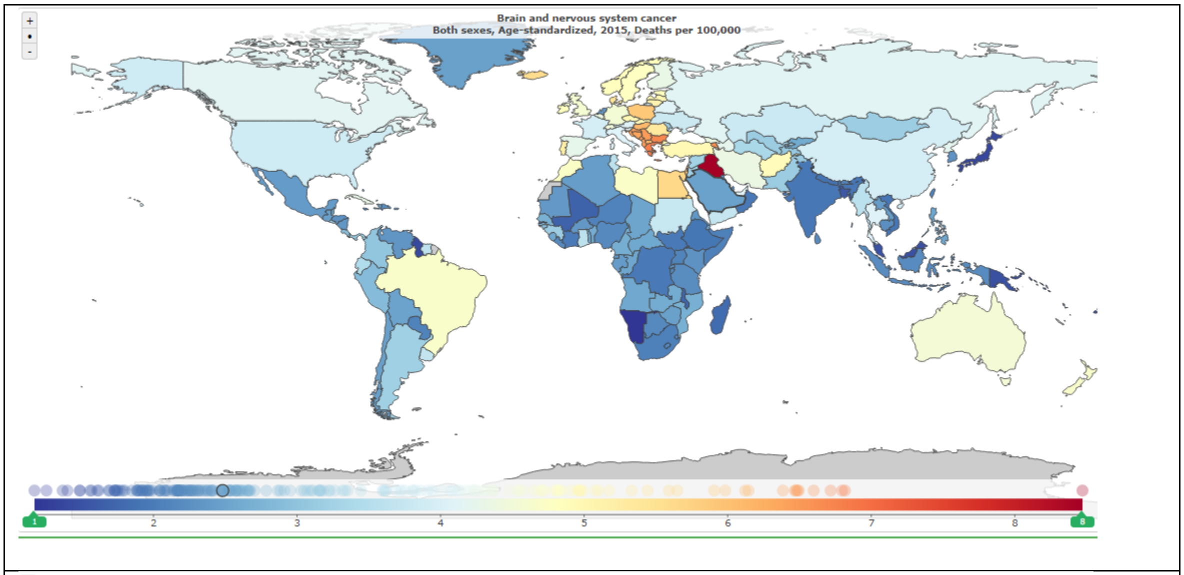
Brain and nervous system cancer
Both sexes, Age-standardized, 2015, Deaths per 100,000
1
2
3
4
5
6
7
8
8

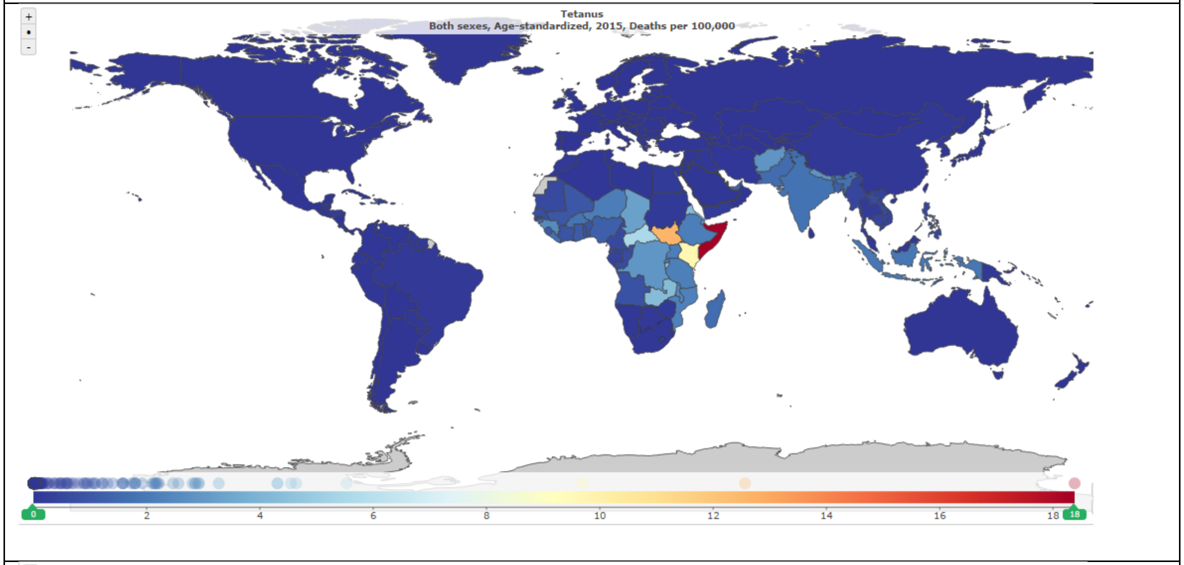
Tetanus
Both sexes, Age-standardized, 2015, Deaths per 100,000
0
2
4
6
8
10
12
14
16
18
18

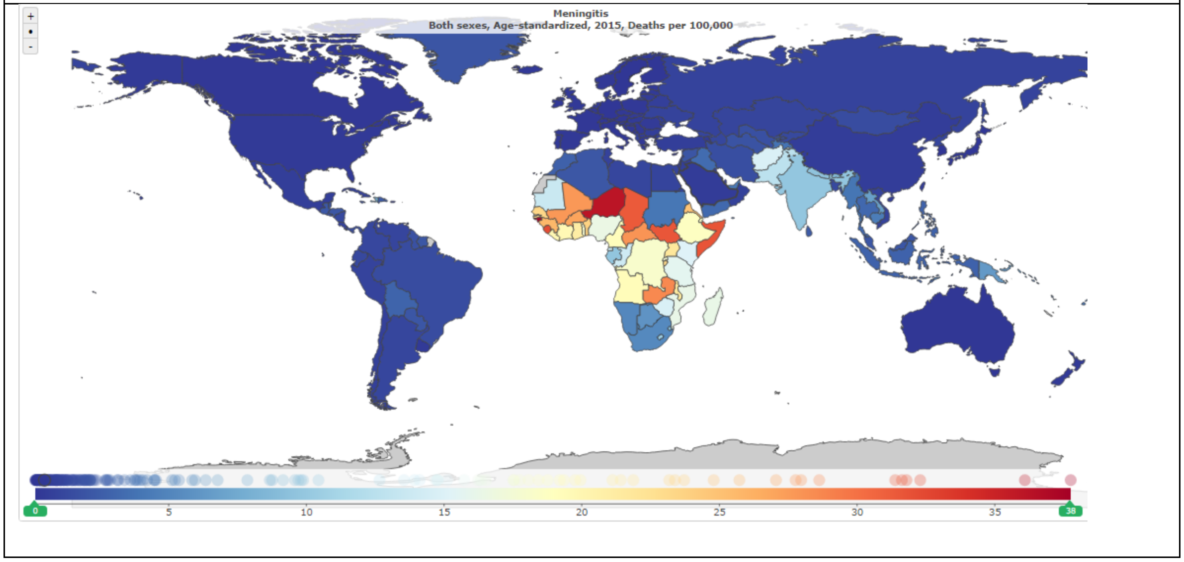
Meningitis
Both sexes, Age-standardized, 2015, Deaths per 100,000
0
5
10
15
20
25
30
35
38

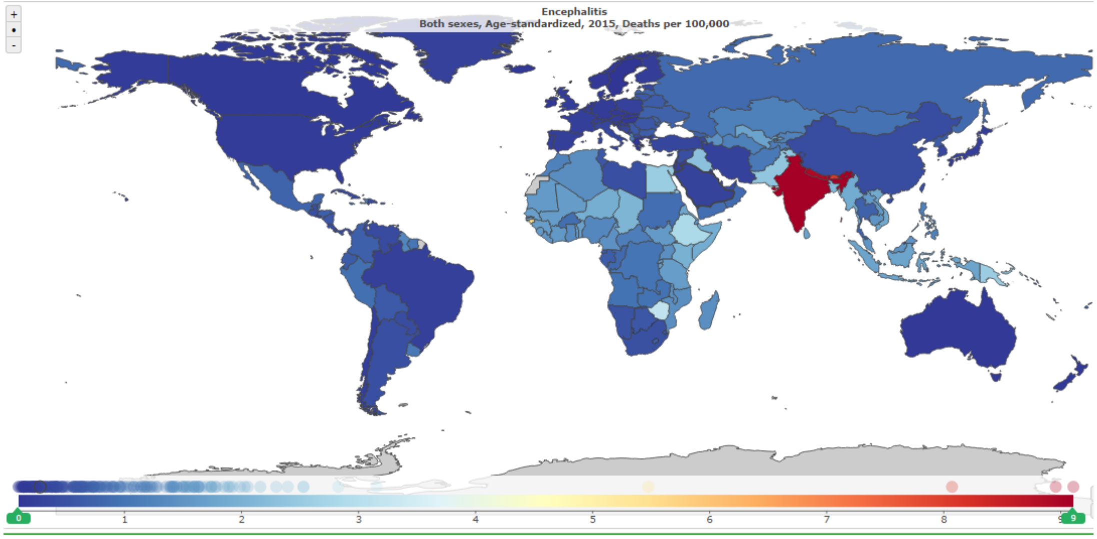
Encephalitis
Both sexes, Age-standardized, 2015, Deaths per 100,000
0
1
2
3
4
5
6
7
8
9

Appendix Figure 5. Expected relationship between age-standardised DALY rates per 100,000 for individual neurological disorders by GBD regions, by sex, 1990–2015

a. Cerebrovascular disease

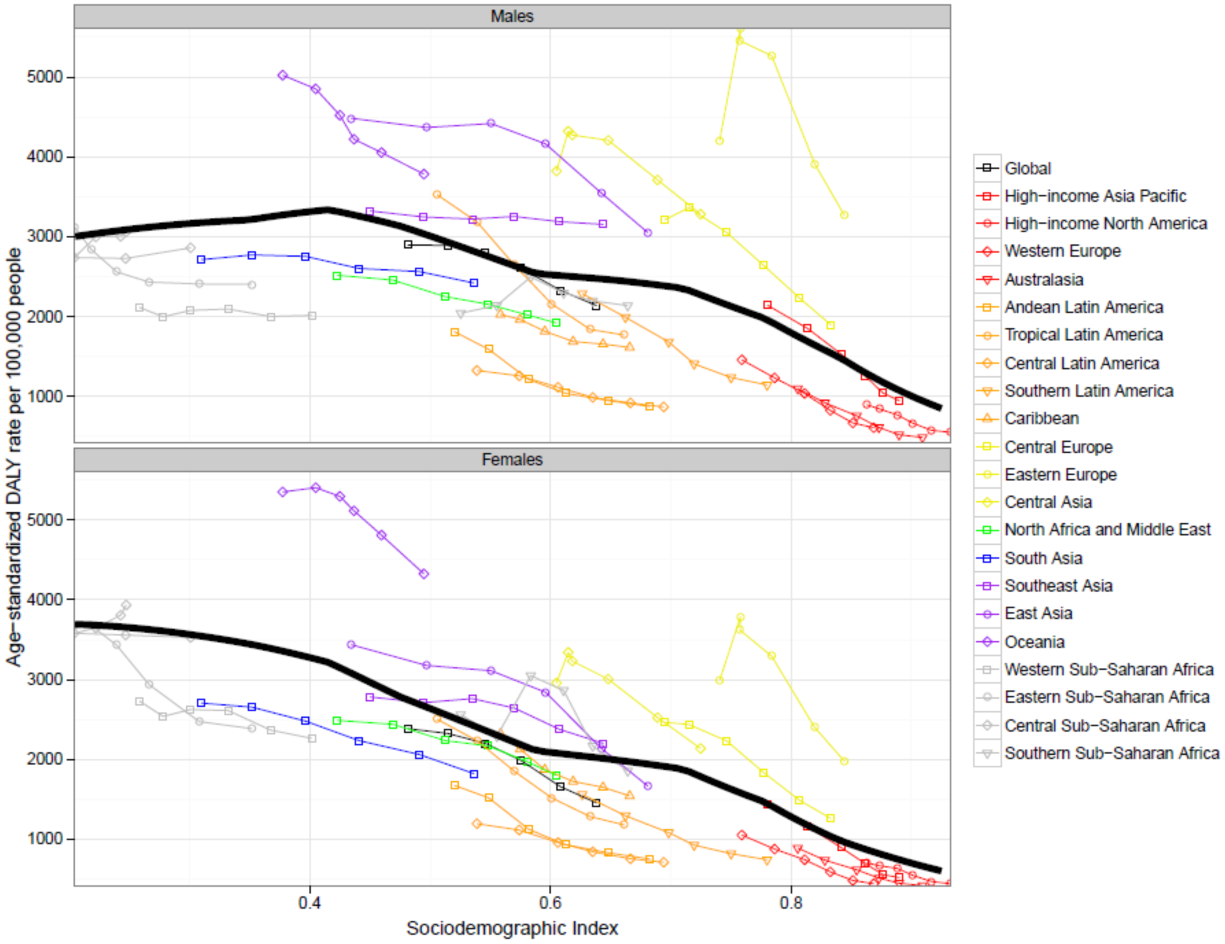

b. Alzheimer’s disease and other dementias

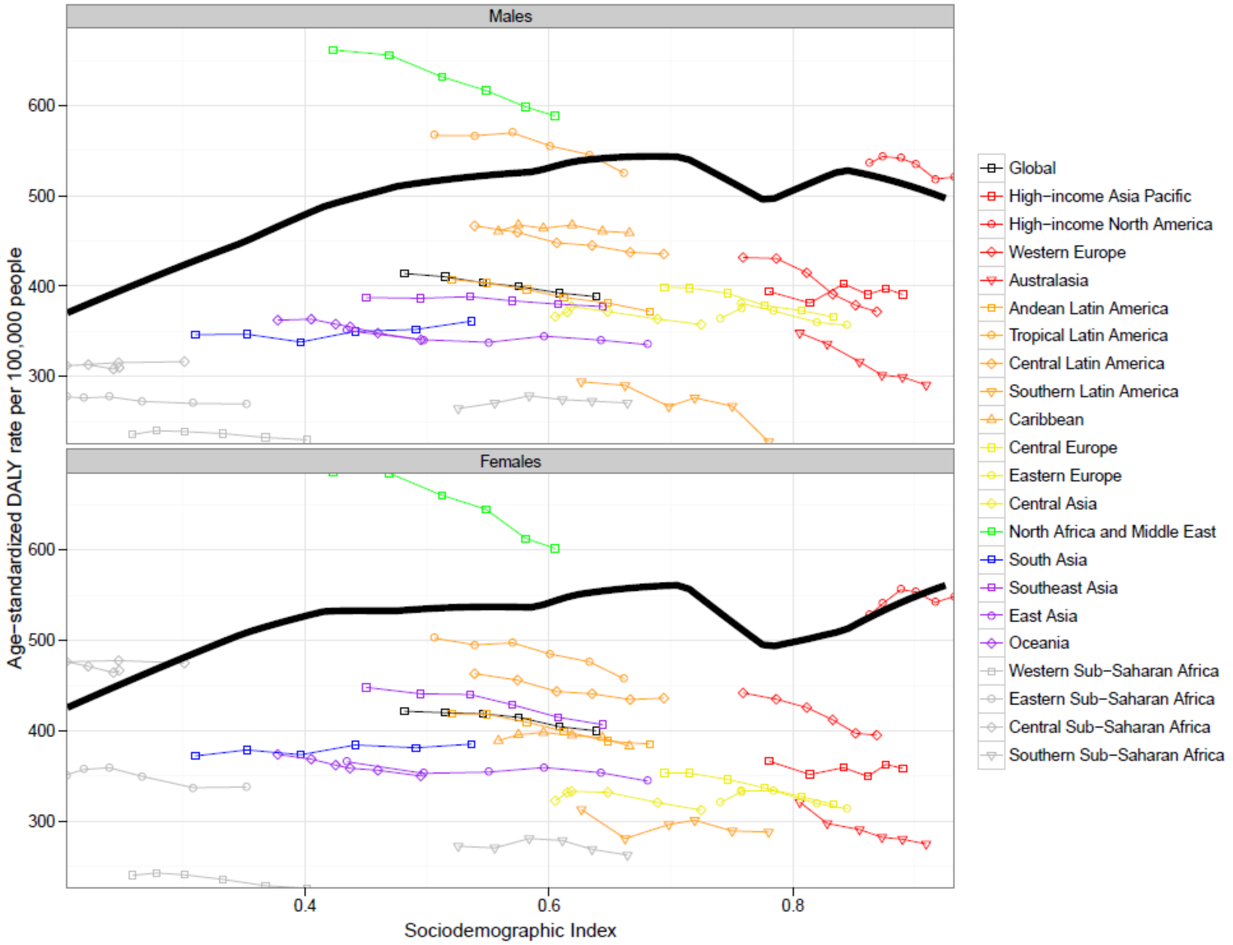

c. Migraine

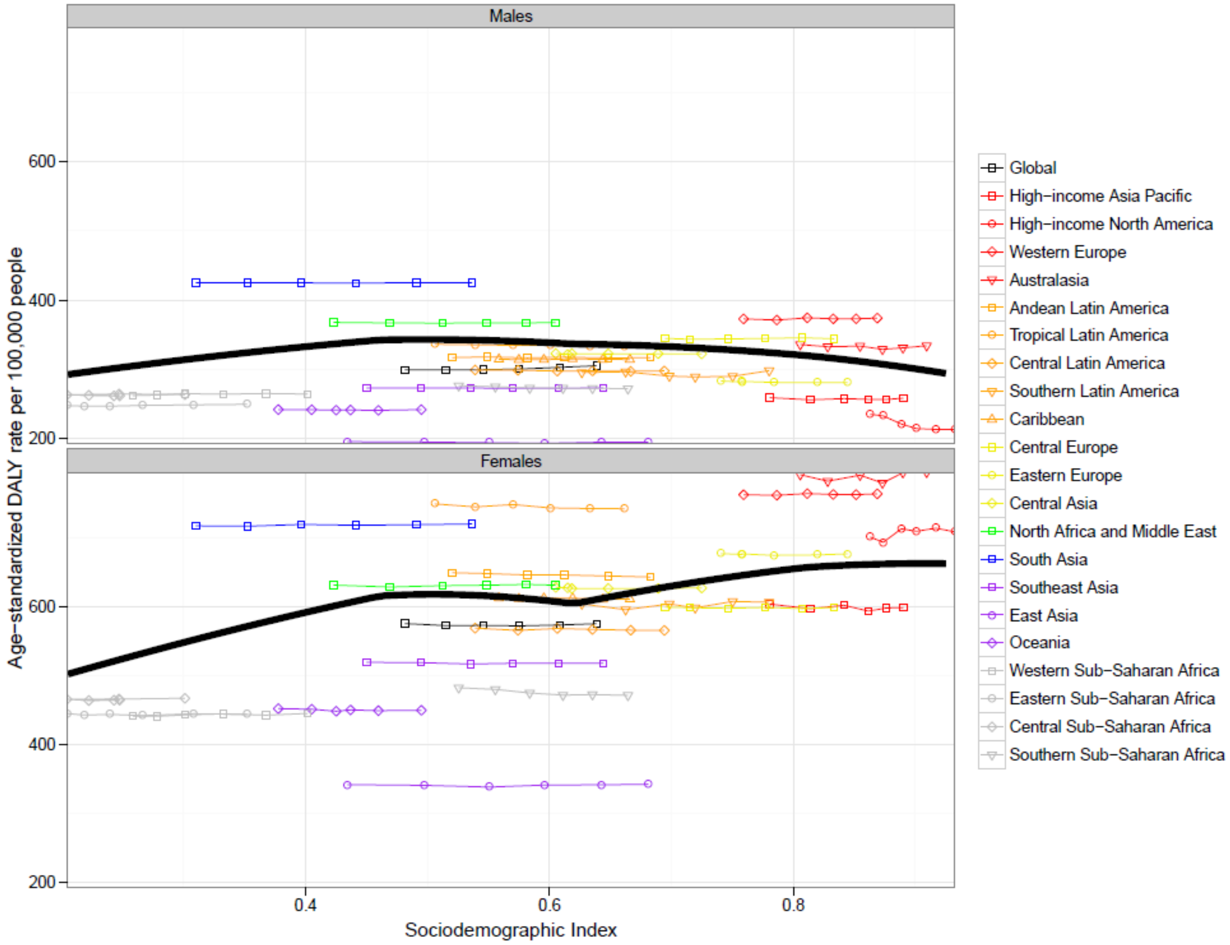

Males
Females
Age-standardized DALY rate per 100,000 people
600
400
200
0.4
0.6
0.8
Sociodemographic Index
Global
High-income Asia Pacific
High-income North America
Western Europe
Australasia
Andean Latin America
Tropical Latin America
Central Latin America
Southern Latin America
Caribbean
Central Europe
Eastern Europe
Central Asia
North Africa and Middle East
South Asia
Southeast Asia
East Asia
Oceania
Western Sub-Saharan Africa
Eastern Sub-Saharan Africa
Central Sub-Saharan Africa
Southern Sub-Saharan Africa

d. Epilepsy

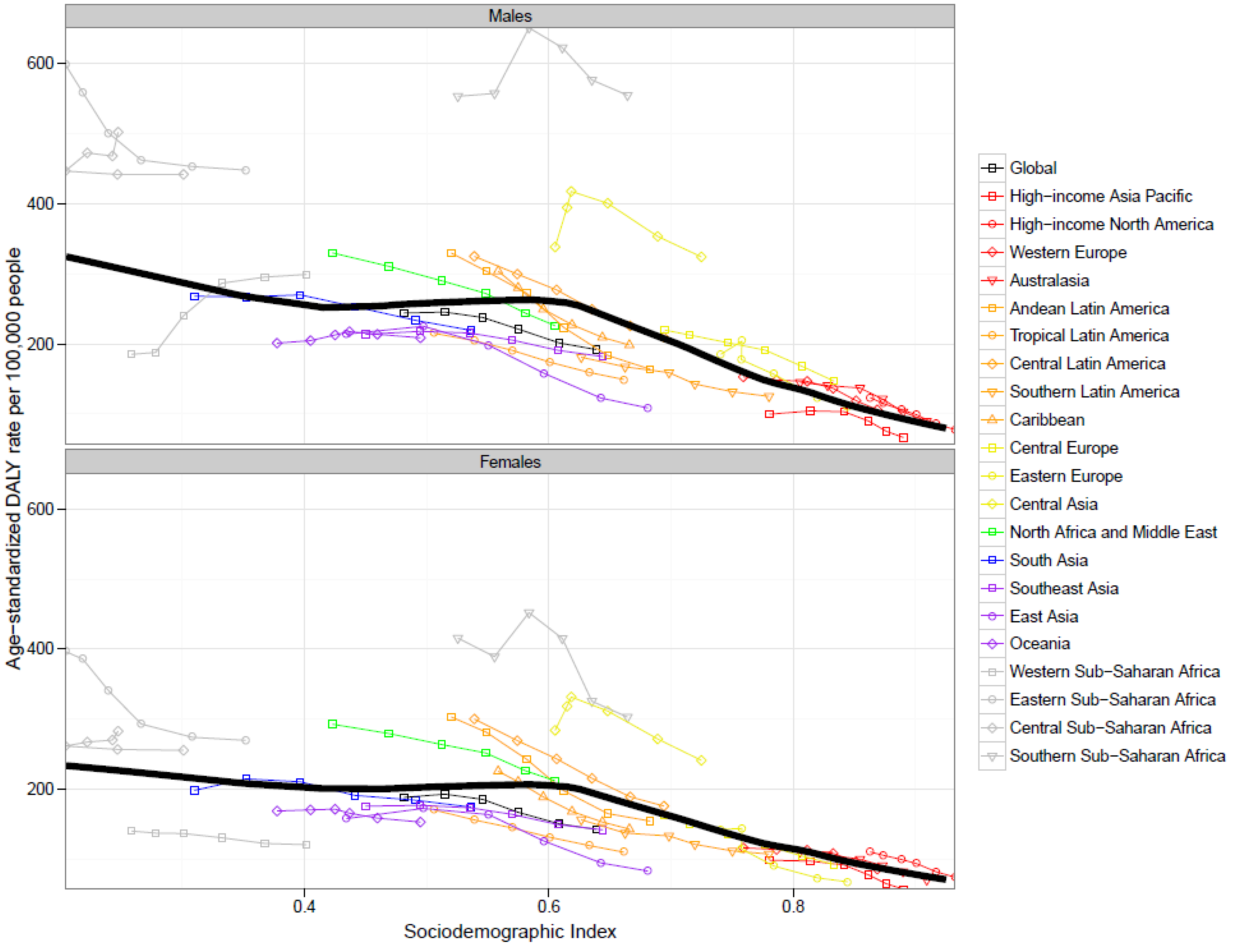

Males
Females
600
400
200
0.4
0.6
0.8
Age-standardized DALY rate per 100,000 people
Sociodemographic Index
Global
High-income Asia Pacific
High-income North America
Western Europe
Australasia
Andean Latin America
Tropical Latin America
Central Latin America
Southern Latin America
Caribbean
Central Europe
Eastern Europe
Central Asia
North Africa and Middle East
South Asia
Southeast Asia
East Asia
Oceania
Western Sub-Saharan Africa
Eastern Sub-Saharan Africa
Central Sub-Saharan Africa
Southern Sub-Saharan Africa

e. Meningitis

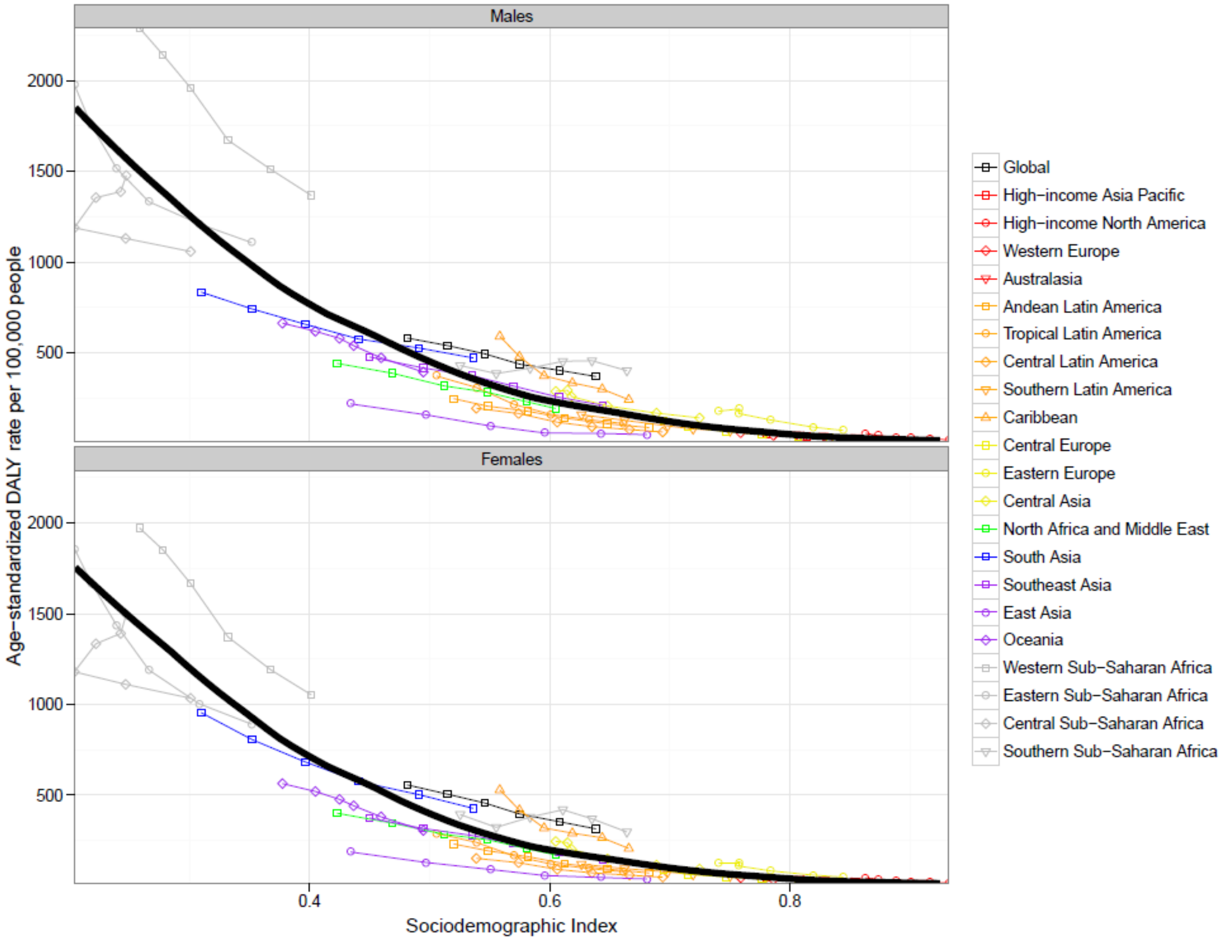

f. Parkinson's disease

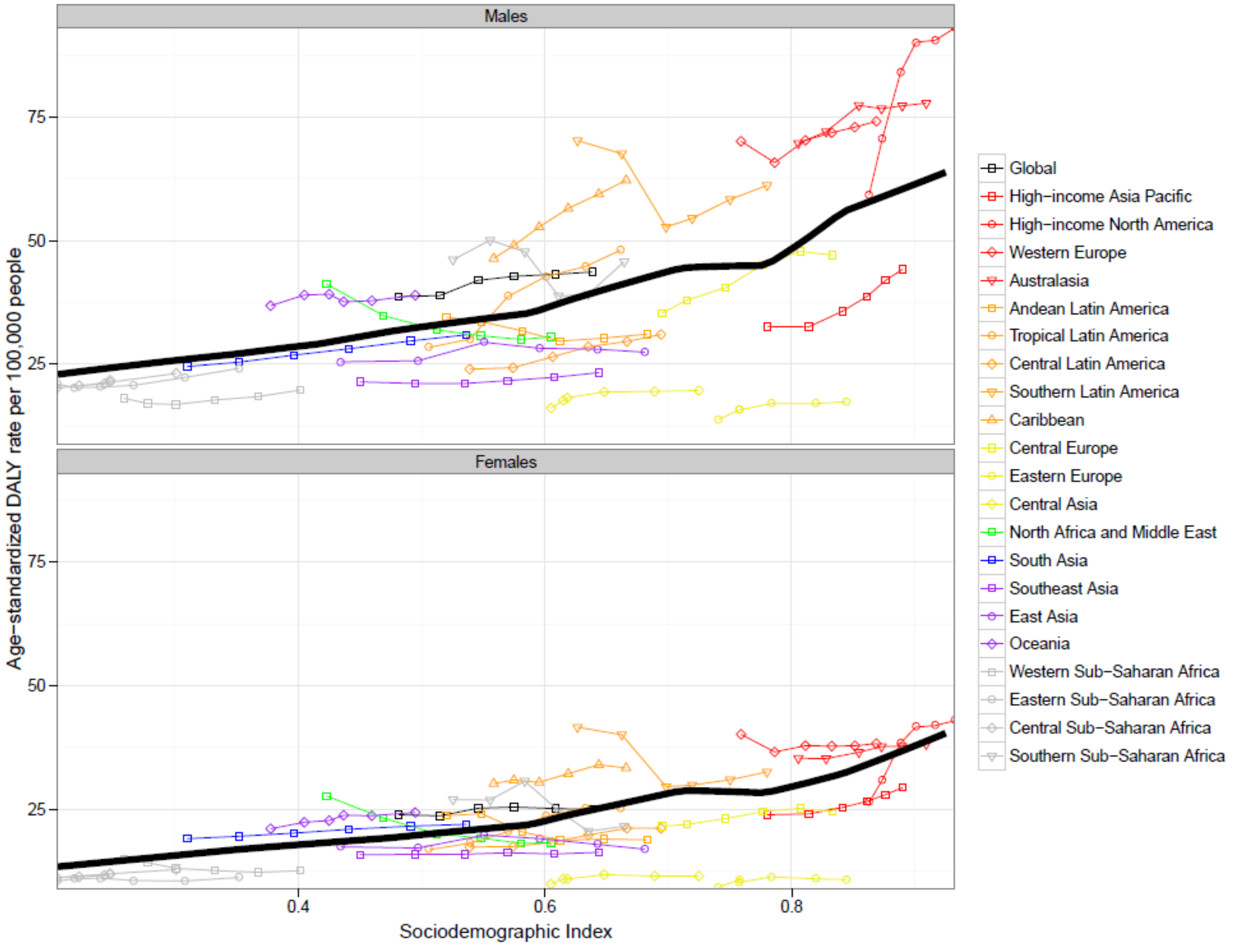

g. Multiple sclerosis

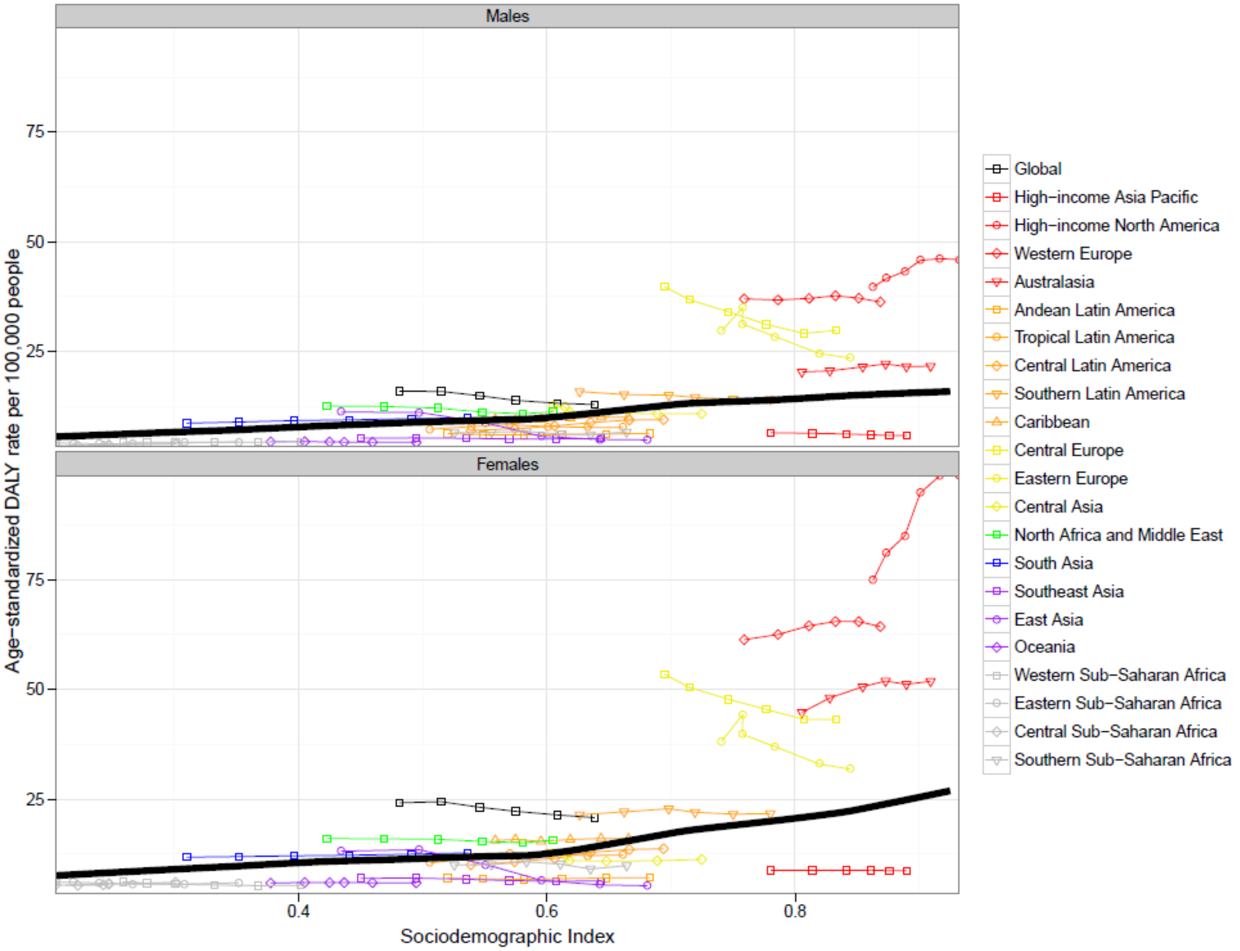

h. Motor-neuron disease

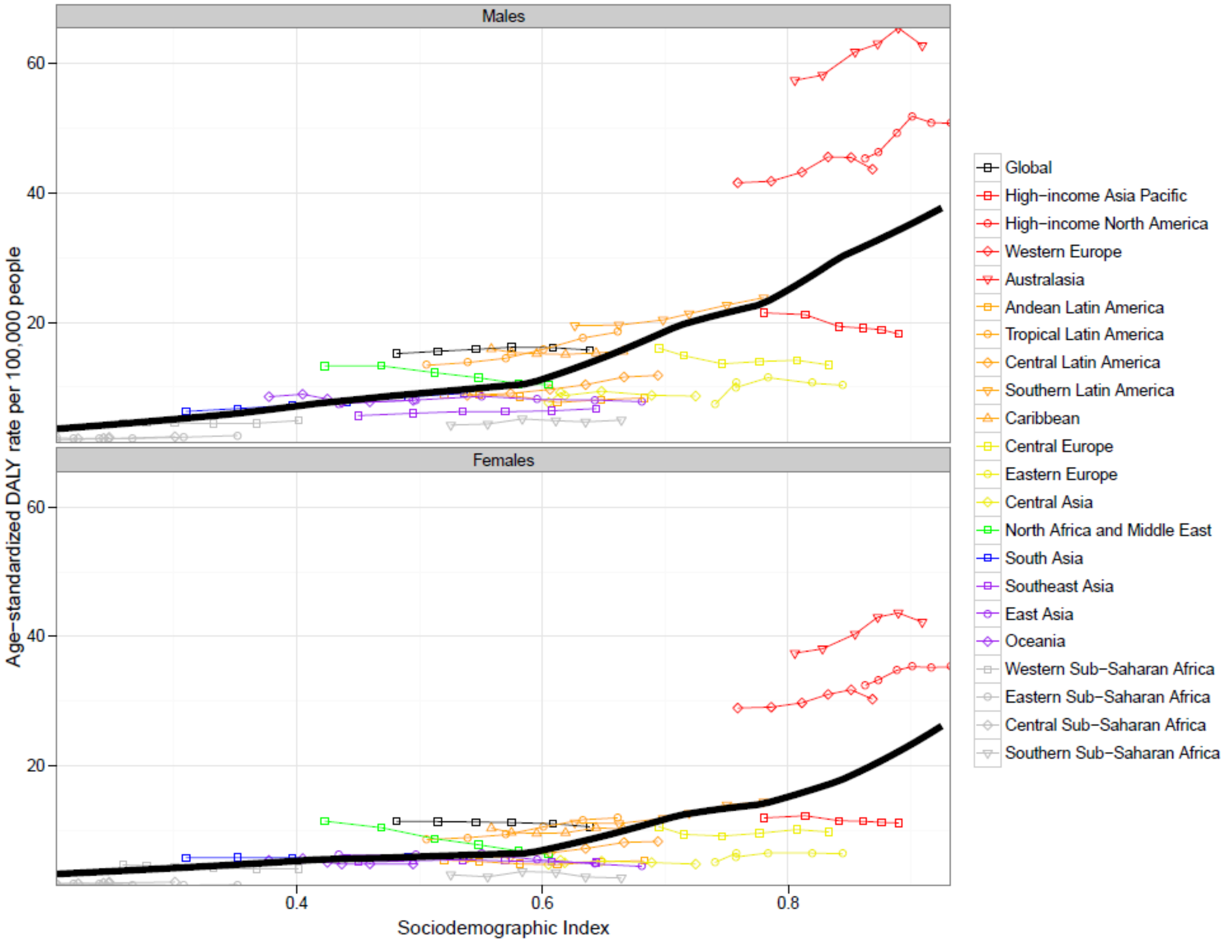

i. Tension-type headache

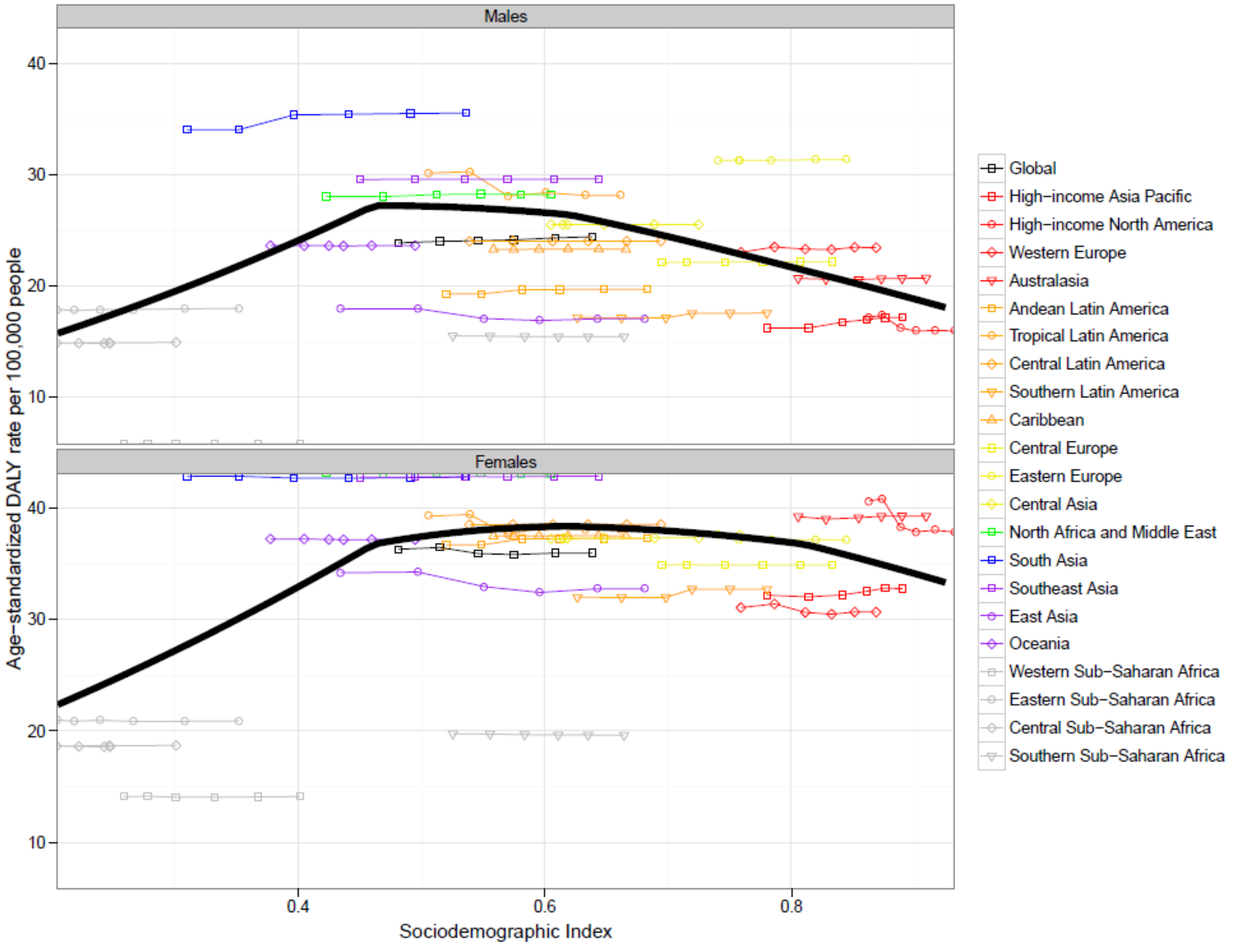

j. Medication overuse headache

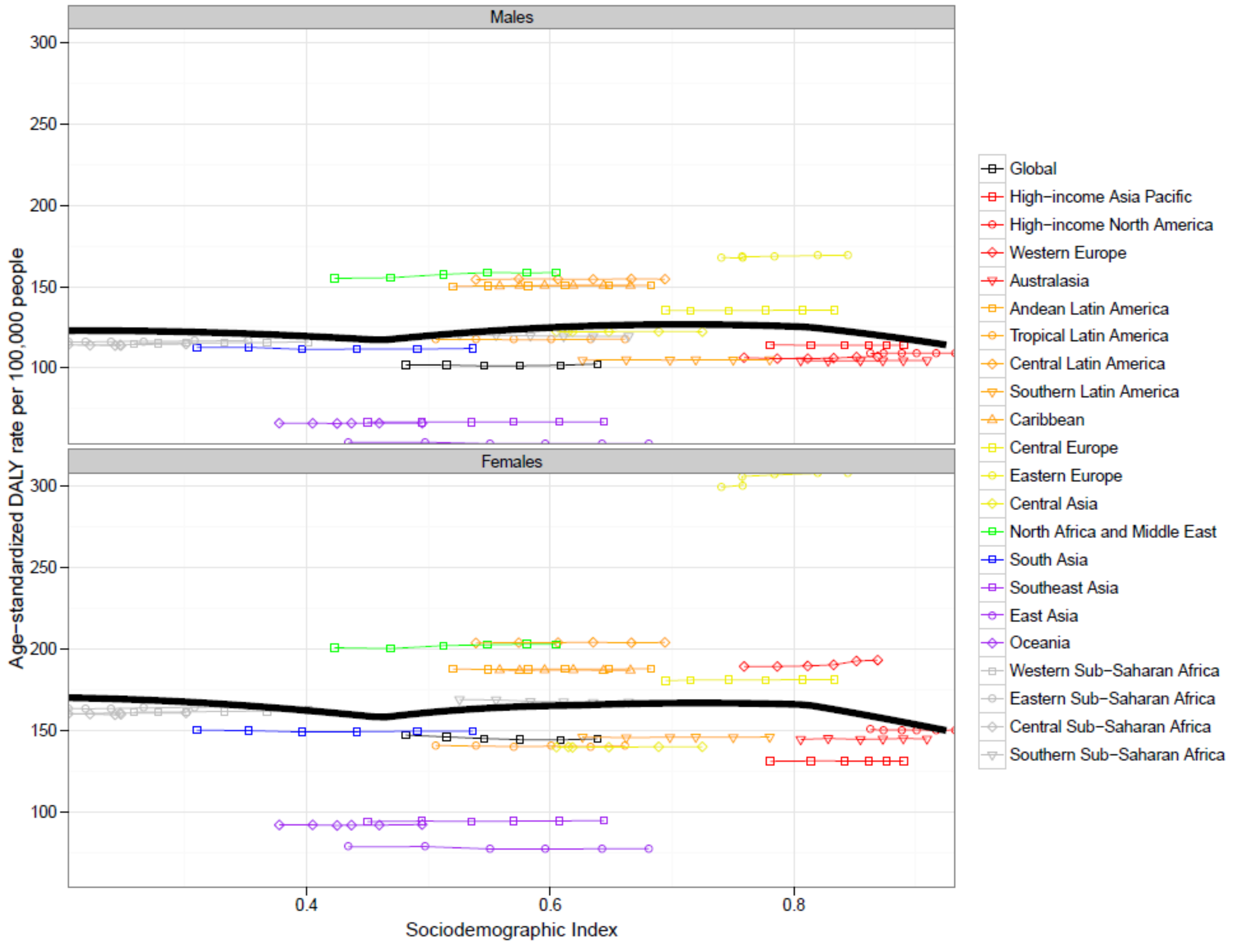

k. Brain and other nervous system cancer

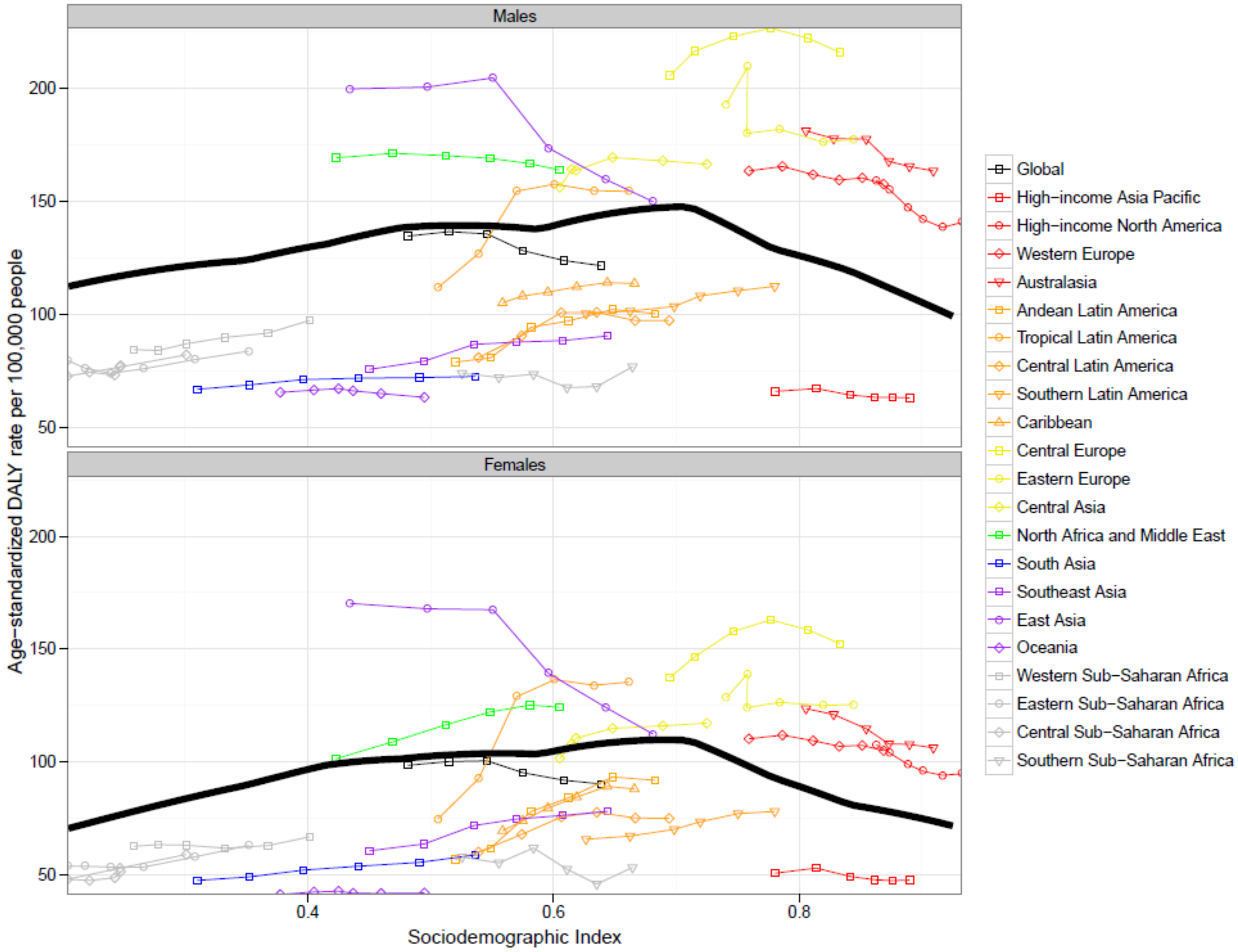

I. Encephalitis

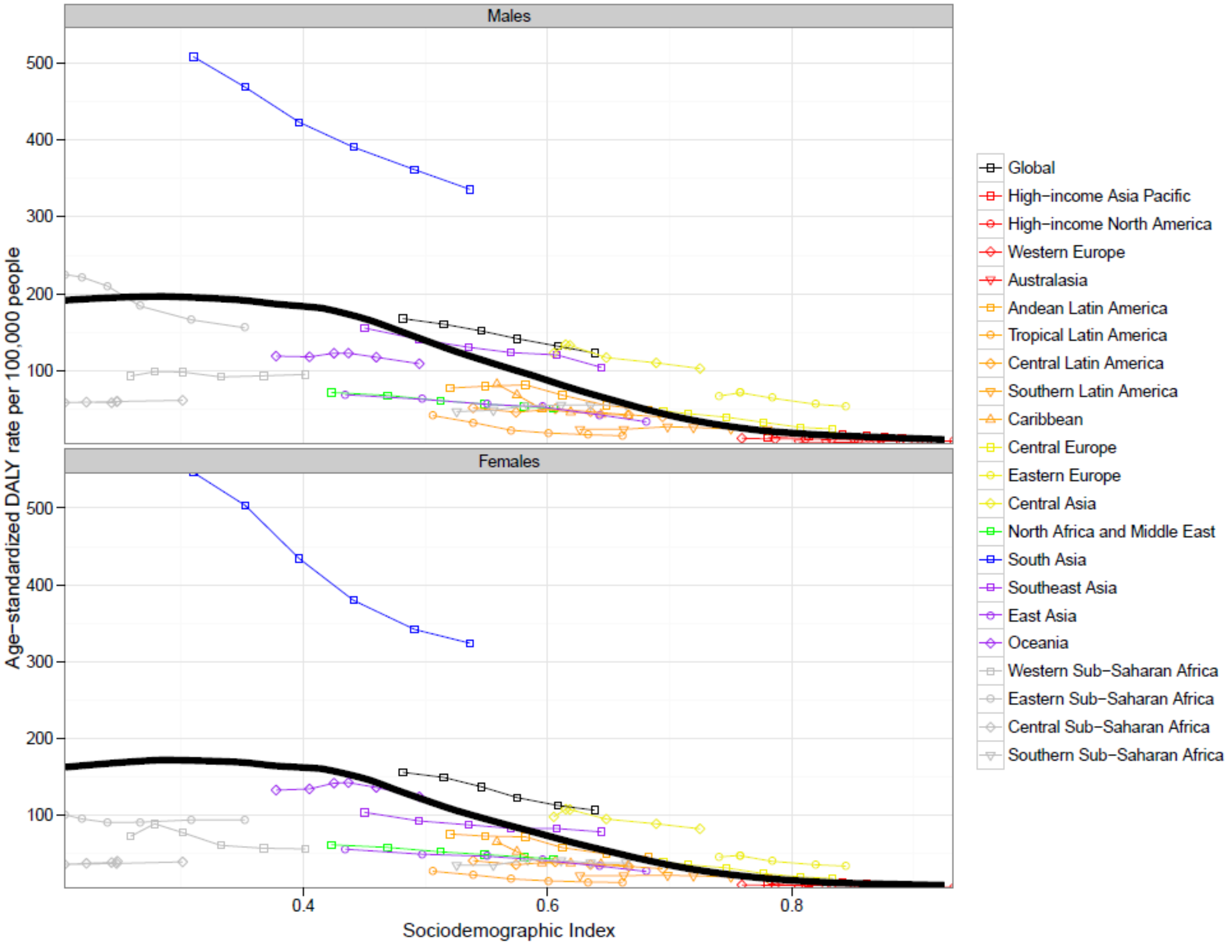

m. Tetanus

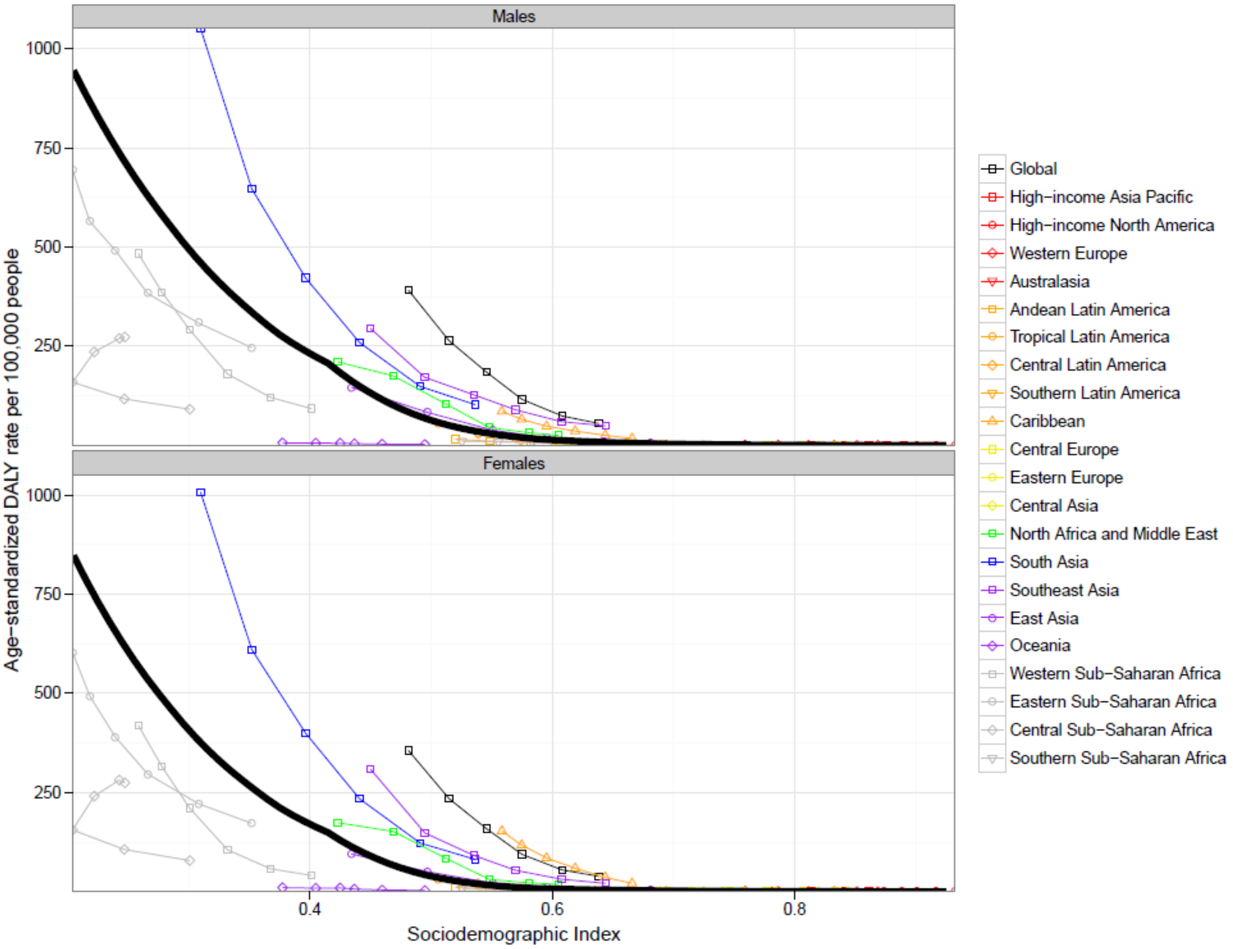

Males
Females
Age-standardized DALY rate per 100,000 people
1000
750
500
250
0.4
0.6
0.8
Sociodemographic Index
Global
High-income Asia Pacific
High-income North America
Western Europe
Australasia
Andean Latin America
Tropical Latin America
Central Latin America
Southern Latin America
Caribbean
Central Europe
Eastern Europe
Central Asia
North Africa and Middle East
South Asia
Southeast Asia
East Asia
Oceania
Western Sub-Saharan Africa
Eastern Sub-Saharan Africa
Central Sub-Saharan Africa
Southern Sub-Saharan Africa

n. Other neurological disorders

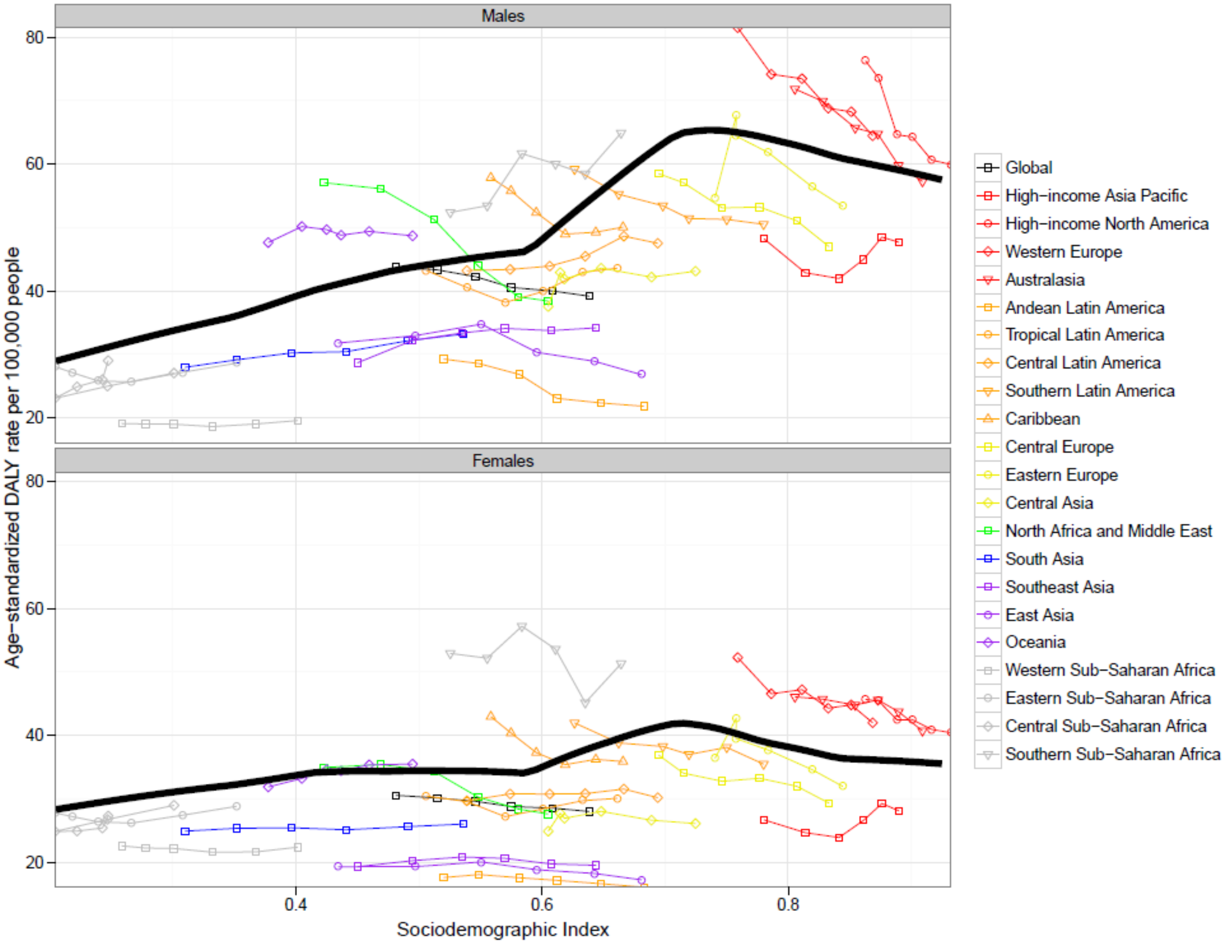

Supplement: Supplementary appendix [file mmc1.pdf]
